# Photogenerated-radical cyclopropylation of *in situ* generated iminiums mediated by NaI/PPh_3_: direct access to α-cyclopropyl tertiary alkylamines

**DOI:** 10.1039/d5sc06039g

**Published:** 2025-10-21

**Authors:** Ying Zhou, Shan Wang, Yi-Chuan Liu, Yan Liu, Fei Tan, Hongbo Dong, Jian Wang

**Affiliations:** a Antibiotics Research and Re-evaluation Key Laboratory of Sichuan Province, Sichuan Industrial Institute of Antibiotics, School of Pharmacy, Chengdu University Chengdu 610106 P. R. China wangjianchem@cdu.edu.cn; b Zhejiang Key Laboratory of Critical Care Medicine, The First Affiliated Hospital of Wenzhou Medical University Wenzhou 325000 China; c School of Chemistry and Chemical Engineering, Guangxi University Nanning 530004 China

## Abstract

Using cyclopropyl radicals to install cyclopropanes has been a fast-growing research field in recent years. Meanwhile, direct radical carbonyl alkylative amination has emerged as an ideal protocol for constructing α-branched tertiary amines. Based on the strategy of direct addition of cyclopropyl radicals to *in situ* generated iminium ions, we disclose a method for preparing diverse α-cyclopropyl tertiary alkylamines by photogenerated-radical cyclopropylation mediated by NaI/PPh_3_ using abundant feedstocks (aldehydes and amines) and easily procured cyclopropyl active esters. Importantly, NaI/PPh_3_ works as both the photoinitiator and sacrificial reductant in this reaction and hence gives an economical variant of carbonyl alkylative amination under mild reaction conditions. In addition, the electrochemical variant of this photogenerated-radical cyclopropylation was also investigated with the preliminary results.

## Abstract 2

Using cyclopropyl radicals to install cyclopropanes has been a fast-growing research field in recent years.

## Graphical abstract

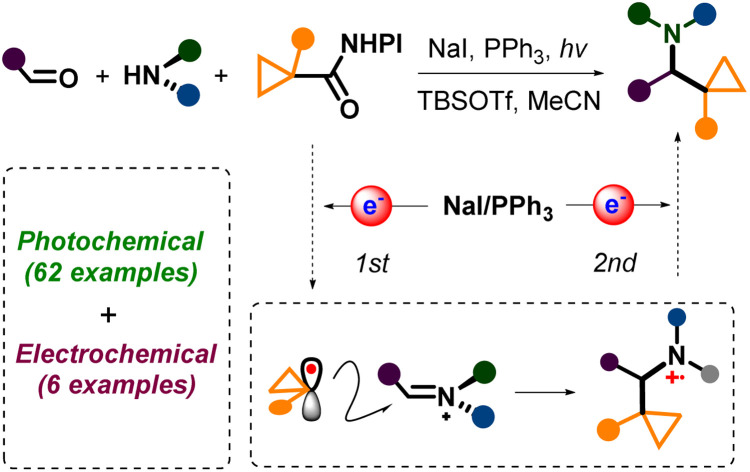

## Introduction

**Scheme 1:**
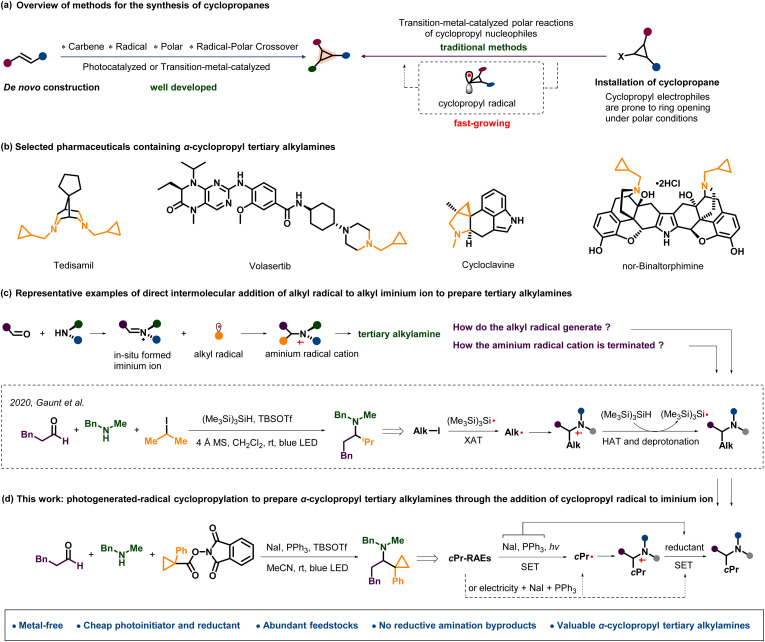
Background and summary of the present work.

The cyclopropyl motif can be frequently found in natural products and pharmaceuticals, and it usually possesses unique physicochemical and pharmacokinetic properties.^1^ As the smallest carbocycles, cyclopropanes have also been widely studied and applied in organic synthesis due to their high ring strain and unique bonding properties.^2^ Generally, two types of strategies for acquisition of the cyclopropyl unit have been summarized, namely *de novo* construction of cyclopropyl rings and installation of an existing cyclopropane (Scheme 1a).^3^*De novo* cyclopropane syntheses have made great achievements such as classical Simmons–Smith cyclopropanation, the Kulinkovich reaction, Corey–Chaykovsky cyclopropanation, and so on. Driven by the recent renaissance in photochemistry, a variety of new methods based on carbene transfer or radical reactions have been developed to construct cyclopropanes using the *de novo* strategy under relatively mild conditions.^4^ On the other hand, traditionally, the direct installation of an existing cyclopropane into target molecules has been focused on transition-metal-catalyzed cross-coupling reactions of cyclopropyl-containing substrates, especially the polar reactions of cyclopropyl nucleophiles.^3*a*,*f*^ It's known that two-electron chemistry at cyclopropyl electrophiles can easily lead to cyclopropyl ring opening. In contrast, cyclopropyl-radical-involved processes usually retain the cyclopropane ring.^5^ Accordingly, using cyclopropyl radicals to install cyclopropanes has been intensively investigated in recent years.^6^

α-Cyclopropyl tertiary alkylamines have appeared in many biologically active compounds and pharmaceuticals (Scheme 1b). But tertiary alkylamines possessing geminal alkyl and cyclopropyl groups at the α-positions of nitrogen are scarce due to the shortage of straightforward methods for their construction. Like other α-branched aliphatic amines, preparing branched α-cyclopropyl tertiary alkylamines *via* conventional two-component synthesis methods, such as carbonyl reductive amination,^7^*N*-alkylation of amines,^8^ transition metal-catalyzed C–N cross-coupling,^9^ imine addition,^10^ and hydroamination of alkenes,^11^ is often problematic. They may suffer from poor steric hindrance tolerance, overalkylation, high cost and chemical waste from metal catalysts, unstable organometallic reagents, requiring special auxiliary groups, and narrow availability of the starting materials. To address these challenges, some multicomponent reactions based on the strategy of the direct addition of carbon nucleophiles to *in situ* generated imines or iminium ions were developed, such as Strecker reactions,^12^ Mannich reactions,^13^ Petasis boron–Mannich reactions and aza–Morita–Baylis–Hillman reactions.^14^ However, these aforementioned multicomponent reactions usually require special nucleophiles with attenuated basicity, and a general method for the direct addition of alkyl groups to *in situ* generated imines and iminium ions, which could rapidly synthesize complex α-branched aliphatic amines, is highly desirable.^15^

In 2020, Gaunt and co-workers reported a radical carbonyl alkylative amination system for constructing structurally diverse α-branched tertiary amines from commercially available feedstocks under irradiation with blue light-emitting diodes (LEDs) (Scheme 1c).^16^ They used alkyl iodide to generate a neutral carbon-centred radical through a halogen atom transfer (XAT) step between a silyl radical and alkyl halide. Then, the alkyl radical added to the iminium intermediates *in situ* generated from secondary amines and aldehydes to form the aminium radical cation, which was rapidly terminated by the silane reagent through hydrogen-atom transfer (HAT). Most importantly, during this reaction, the novel elementary mechanistic step, adding an alkyl radical to positively charged iminium ions, provided great opportunities for the development of new alkyl amine synthetic methods.^17^ Following this precedent, the addition of carbamoyl and fuoromethyl radicals to iminium ions is successfully realized to prepare corresponding valuable amines.^18^ Meanwhile, different starting materials working as alkyl iminium ions or neutral alkyl imine sources, such as the combinations of primary amines with α-ketoesters or aldehydes and even secondary amides, were also reported to engage similar radical carbonyl alkylative amination.^19^ Furthermore, the Gaunt group recently disclosed another two new “higher-order” variants of carbonyl reductive amination, which involved the 2e^−^ addition process to iminium ions using alkyl zinc and 2-azinyl indium species, namely zinc-mediated carbonyl alkylative amination and carbonyl azinylative amination.^20^ Inspired by the fast-growing higher-order carbonyl reductive amination, we envisioned that cyclopropyl radicals could participate in the addition to iminium ions (Scheme 1d). This assumption enables us to efficiently prepare α-cyclopropyl tertiary alkylamines in a streamlined synthesis method. For this desired reaction manifold, the key points are how to generate the initial radical and how to terminate the aminium radical cation derived from the addition of the radical to iminium ion.

## Results and discussion

In our initial investigations, the easily procured cyclopropyl *N*-hydroxyphthalimide (NHPI) ester (3a) was first chosen as the cyclopropyl radical precursor, and *N*-methylbenzylamine (2a) and hydrocinnamaldehyde (1a) worked as the iminium ion sources. In 2019, the Fu group showed that the combination of sodium iodide and triphenylphosphine (NaI/PPh_3_) under blue light irradiation could reduce redox-active NHPI esters to generate alkyl radicals.^21^ Since then, numerous iodide/phosphine-mediated photoredox radical transformations have been reported, which have the advantage of having a more accessible and cost-effective catalyst system.^22^ Hence, we tried the first condition of the model reaction by using NaI/PPh_3_ as a photocatalyst and using tris(trimethylsilyl)silane ((Me_3_Si)_3_Si–H) as a reductant under blue light irradiation (Table 1, entry 1). The desired amine (4a) was obtained with a 45% isolated yield, and the reductive amination byproduct (5a) was also isolated in 12% yield. Surprisingly, without (Me_3_Si)_3_Si–H, in the presence of NaI/PPh_3_ and *tert*-butyldimethylsilyl trifluoromethanesulfonate (TBSOTf), the model reaction could also produce 4a and no 5a was detected. During this transformation, triphenylphosphine oxide (TPPO) was generated as the most conspicuous byproduct. Therefore, we conjectured that PPh_3_ worked not only as a photoinitiator but also as the terminal reductant. Crucially, as there is no reductive amination happening here, we inferred that it would be easy to get a high yield of this reaction through modification of the conditions. After screening of the reaction parameters, the optimized reaction conditions with a yield of 87% were identified to be using blue LED light to irradiate the stirred reaction mixture of 1a (0.4 mmol), 2a (0.4 mmol), 3a (0.2 mmol), NaI (0.4 mmol), PPh_3_ (0.4 mmol) and TBSOTf (0.4 mmol) in acetonitrile (1 mL) with cooling using a fan for 24 hours (Table 1, entry 2). The use of an excess amount of amine and aldehyde is necessary to achieve high yields, but unreactive amine and aldehyde can be efficiently recovered. Importantly, the one-pot strategy by directly using carboxylic acids as raw materials by their *in situ* activation without further purification and presynthesis of NHPI esters is also successful (Table 1, entry 3). Control experiments validated that light, NaI and PPh_3_ are indispensable to conduct this transformation successfully (entry 4–7). Reducing the amount of NaI or PPh_3_ would make the yield significantly decrease (entry 8 and 9). Other solvents such as ethyl acetate and dichloromethane resulted in lower yields (entry 10 and 11). When TBSOTf was replaced by acetic acid or trimethylsilyl trifluoromethanesulfonate (TMSOTf), the reaction worked but with lower yields (entry 12 and 13). Maintaining a high concentration of the reactive alkyl iminium ion, which captures the alkyl radical, is crucial to obtaining high yield. As such, reactions that used excess amounts of 3a over 1a and 2a gave lower yield than the standard conditions (entry 14). Except for 3a, two other cyclopropyl radical precursors *N*-hydroxytetrachlorophthalimide (TCNHPI) esters 6a and *N*-hydroxyphthalimide oxalate 7a were also examined under similar conditions (entry 14 and 15). More easily reduced redox active ester 6a (6a: *E*_p_ = −1.213 V *vs.* Fc^+^/Fc, 3a: *E*_p_ = −1.690 V *vs.* Fc^+^/Fc)^23^ generated the product 4a in a slightly higher yield (entry 15), while 7a could not produce the product at all (entry 16).^24^

**Table 1:** Optimization of reaction conditions^a^

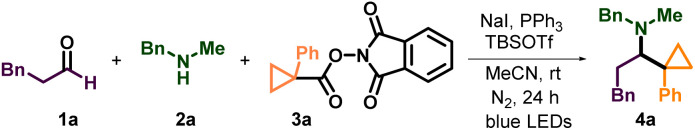		
Entry	Deviation from the standard conditions	Yield^b^ (%)
1^c^	With 2.0 equiv. (TMS)_3_SiH as a reductant	48 (12)
2	None	87
3^d^	One-pot method using carboxylic acid	67
4	In the dark	0
5	Without NaI	0
6	Without PPh_3_	0
7	In the dark, at 50 or 80 °C	0
8	NaI (0.25 equiv.)	45
9	PPh_3_ (0.25 equiv.)	14
10	In EA	48
11	In DCM	31
12	CH_3_COOH instead of TBSOTf	30
13	TMSOTf instead of TBSOTf	51
14^e^	1a/2a/3a = 1 : 1 : 2	55
15	6a instead of 3a	90
16	7a instead of 3a	0
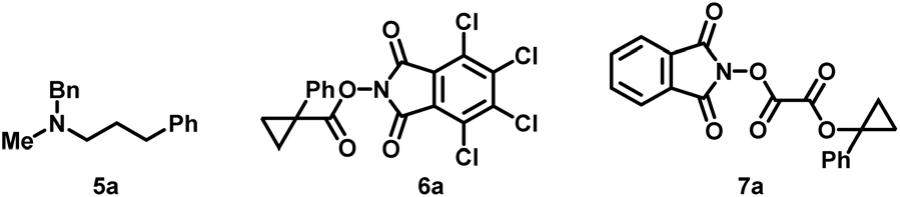		

a Reaction conditions: 1a (0.4 mmol, 2.0 equiv.), 2a (0.4 mmol, 2.0 equiv.), 3a (0.2 mmol, 1.0 equiv.), NaI (0.4 mmol, 2.0 equiv.), PPh_3_ (0.4 mmol, 2.0 equiv.), TBSOTf (0.4 mmol, 2.0 equiv.), MeCN (1 mL), rt, N_2_, 24 h, blue LEDs.

b Isolated yield.

c Yield of 5a is given in parentheses.

d (i) 1-Phenyl-1-cyclopropane-carboxylic acid (0.2 mmol), NHPI (0.22 mmol), 4-dimethylaminopyridine (5%), *N*,*N*-dicyclohexyl-carbodiimide (0.22 mmol), MeCN, 0 °C to rt, 2 h. (ii) Standard conditions without 3a.

e 1a (0.2 mmol, 1.0 equiv.).

**Scheme 2:**
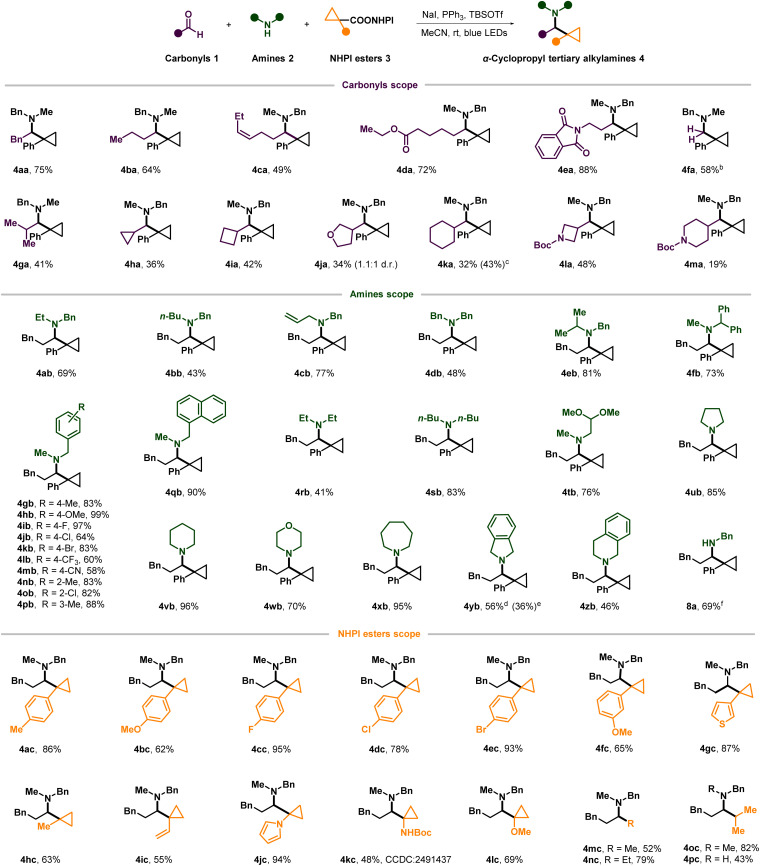
(a) 1 (0.4 mmol), 2 (0.4 mmol), 3 (0.2 mmol), NaI (0.4 mmol), PPh_3_ (0.4 mmol), TBSOTf (0.4 mmol), MeCN (1 mL), rt, N_2_, 24 h, blue LEDs. (b) Paraformaldehyde (2.0 mmol) was used as the formaldehyde source. (c) 1 (0.8 mmol), 2 (0.8 mmol), 3 (0.2 mmol), NaI (0.8 mmol), PPh_3_ (0.8 mmol), TBSOTf (0.8 mmol), MeCN (2 mL). (d) Amine·HCl (0.4 mmol) was freed beforehand using Et_3_N (0.4 mmol) in MeCN (1 mL). (e) Amine·HCl was used directly. (f) EtOAc instead of MeCN as solvent.

After optimization, we first explored the substrate scope of aldehydes in the benchmark reaction (Scheme 2). Various linear aldehydes, including those bearing carboxylic ester, phthalimide, alkenyl and aryl groups, reacted efficiently to give the desired amines in moderate to high yields (4aa–4ea). Formaldehyde also reacted with 2a and 3a to afford the unbranched amine 4fa using paraformaldehyde as a coupling partner. Branched aldehydes, including strained or unstrained saturated cyclic and heterocyclic rings, resulted in moderate to low yields, presumably due to the corresponding high steric hindrance in condensation or cyclopropyl radical addition and the lower reactivity of less electrophilic iminium ions (4ga–4ma).^25^ Unfortunately, other more sterically hindered carbonyls such as α-tertiary aldehydes, ketones and α-ketoesters were all totally unreactive in our reaction.

Subsequently, we turned to explore the scope of amines by varying the amine component while retaining hydrocinnam-aldehyde 1a and the redox-active NHPI ester 3a (Scheme 2). Secondary benzylamine derivatives containing the allyl, benzyl, linear or branched alkyl on nitrogen were all amenable to this transformation (4ab–4fb). However, when more sterically hindered tertiary-butyl is connected to the nitrogen center of benzylamine, corresponding photogenerated-radical cyclopropylation didn't work at all. *N*-methylbenzylamines bearing various substituents attached to the aromatic ring were also widely investigated, and all of them delivered the corresponding products in high yield, except for those which bear a strong electron-withdrawing group at the *para* position of arene (4gb–4pb). The *N*-methyl-1-naphthalenyl methanamine reacted smoothly and afforded the desired product with a yield of 90% (4qb). Besides benzylamine derivatives, dialkylated secondary amines also acted as good reaction partners (4rb–4xb), especially for the cyclic amine derivatives of varying ring sizes including pyrrolidine (4ub), piperidine (4vb), morpholine (4wb), and azepane (4xb). In contrast to the aforementioned cyclic amines, benzo-fused cyclic amines such as isoindoline (4yb) and 1,2,3,4-tetrahydroisoquinoline (4zb) showed relatively low activities and resulted in moderate yields (4yb and 4zb). It is needless to mention that amine hydrochloride salt can be used directly in this reaction but with a lower yield (36% of 4yb) compared to the approach where the amine hydrochloride salt was freed beforehand by stirring it with an equal equivalent of triethylamine (Et_3_N) (56% of yield). Last but not least, after a little modification of the standard reaction conditions by using ethyl acetate as solvent, primary benzylamines were also applicable to this multicomponent reaction to access complex secondary amines directly (8a).

Then, reactions with a variety of cyclopropyl redox-active NHPI esters coupled with compounds 1a and 2a were conducted to assess the applicability of this method (Scheme 2). Various functional groups on the benzene ring directly connected to the cyclopropyl were tolerated, and the yields of cyclopropyl alkylamines were generally high (4ac–4fc).

Noteworthily, although it was not easy to get high yields for tertiary alkylating agents during previously reported carbonyl alkylative amination, *a*-aryl cyclopropyl NHPI esters can react efficiently in our reaction to furnish α-tertiary cyclopropyl alkylamines in good yields. In addition, heteroaromatic variants (4gc and 4jc) also reacted smoothly during this photocatalyzed process in very high yields (87–94%). Apart from aryl, other groups including methyl and vinyl and even heteroatom groups at the *a*-position of carboxylic acid NHPI esters on the cyclopropane ring were also tolerated in moderate to high yields, which gave diverse cyclopropanes that enable subsequent elaboration (4hc–4lc). At last, the more common alkyl carboxylic acid NHPI esters could also provide desired tertiary or secondary amines (4mc-4pc) in good yield.

**Scheme 3:**
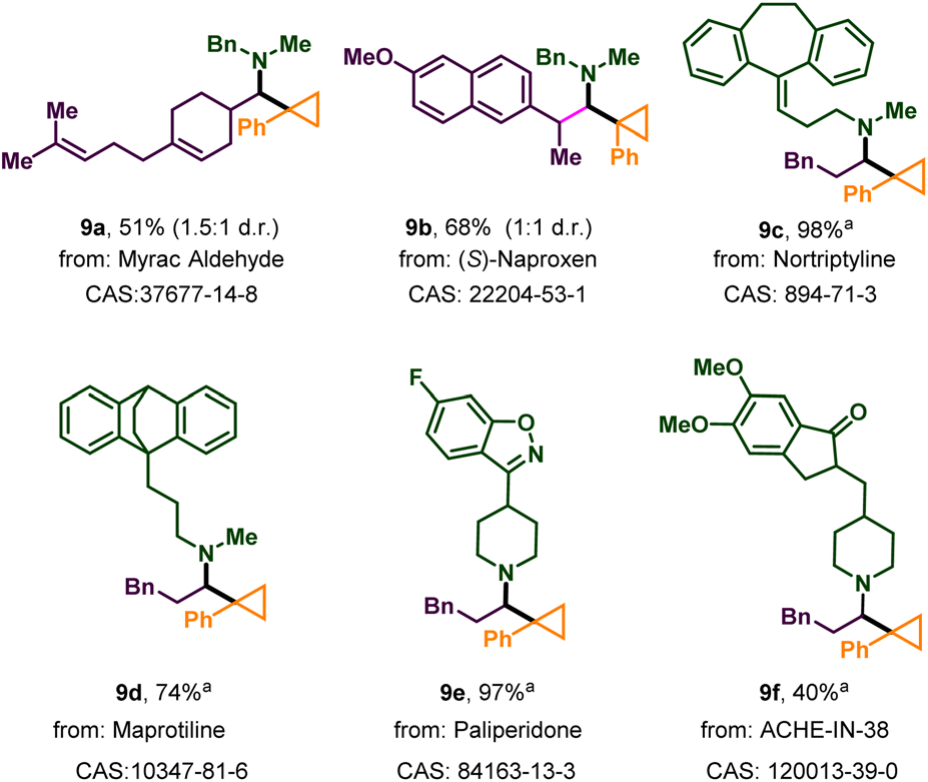
Application of the photogenerated-radical cyclopropylation in modification of natural products and drugs. Reactions were performed on a 0.20 mmol scale under standard conditions. Shown are isolated yields after chromatography. ^a^ Amine·HCl (0.4 mmol) was freed beforehand using Et_3_N (0.4 mmol) in MeCN (1 mL).

Next, the robustness of the protocol was demonstrated on complex natural products, drugs, or their derivatives (Scheme 3). Myracaldehyde and the aldehyde derived from naproxen reacted with 2a and 3a to afford the corresponding cyclopropylation products 9a and 9b in 51% and 68% yield respectively. Meanwhile, as a less sterically hindered secondary amine, the antidepressant nortriptyline gave the desired product 9c in almost quantitative yields (98%). Maprotiline with a similar structure also reacted efficiently (9d). The substructure of paliperidone (9e), which contains isoxazole, was also tolerated in our reaction in very high yield (97%). ACHE–IN–38, which can alleviate memory deficits in patients with Alzheimer's disease, can be transferred into complex α-cyclopropyl tertiary alkylamines (9f) by this method. In conclusion, all of these results showed the strong practicality of this protocol.

**Scheme 4:**
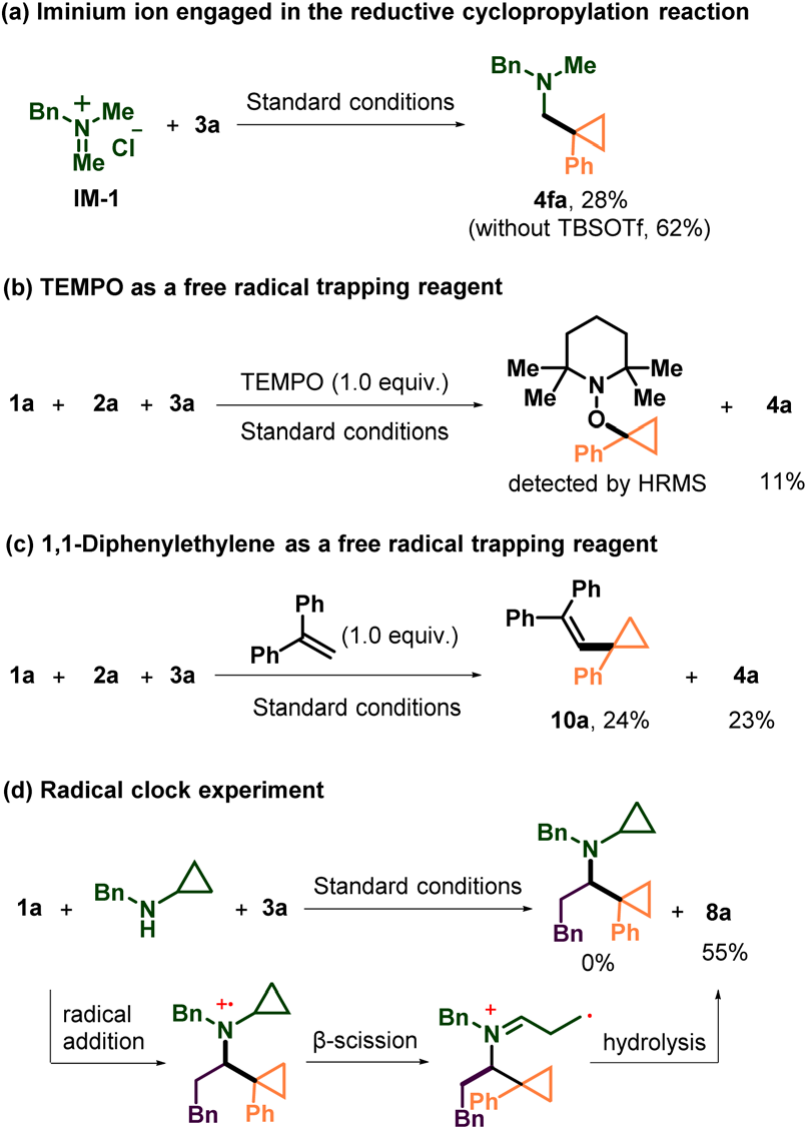
Radical capture experiments, and exploration of iminium ions in the reaction system.

To gain more information about the mechanism of the three-component photogenerated-radical cyclopropylation reaction, we carried out a series of mechanistic experiments. First, we used one prepared iminium salt (IM-1) to replace the corresponding amine and aldehyde to engage in this reaction (Scheme 4a). The product 4fa was isolated in 28% yield, and when TBSOTf was omitted, the yield of 4fa could raise to 62%. These results indicated the *in situ* formation of the iminium ion and its involvement in the subsequent reactions. It also indicated that TBSOTf might be important during the formation of the iminium ion from 1a and 2a but unnecessary in other fundamental reaction steps. Then, a radical scavenger experiment was carried out, and only 11% yield of 4a was obtained when one equivalent of (2,2,6,6 tetramethylpiperidin-1-yl)oxyl (TEMPO) was added into the model reaction under standard reaction conditions (Scheme 4b). More equivalents of TEMPO could totally inhibit the reaction (see the SI). Meanwhile, the TEMPO adduct of the cyclopropyl radical was detected using a high resolution mass spectrometer (HRMS). Next, we used 1,1-diphenylethylene to successfully couple with the cyclopropyl radical and generate 10a in 24% yield (Scheme 4c). In this system, the model reaction was not thoroughly suppressed and could also proceed in 23% yield. Generally, the results of these two radical capturing experiments indicated that carbon-centered radical generation from NHPI esters was highly possible, which was in accord with previous reports.^21^ To validate the existence of the aminium radical cation species during the reaction pathway, cyclopropylamine was used as the amine substrate under standard conditions (Scheme 4d). The reaction ultimately yielded only 8a, which could be explained by the fast ring opening of the cyclopropane structure of the aminium radical cation intermediates, proving that the step in which the radical was added to the iminium ion to form the aminium radical cation was feasible.

**Fig. 1:**
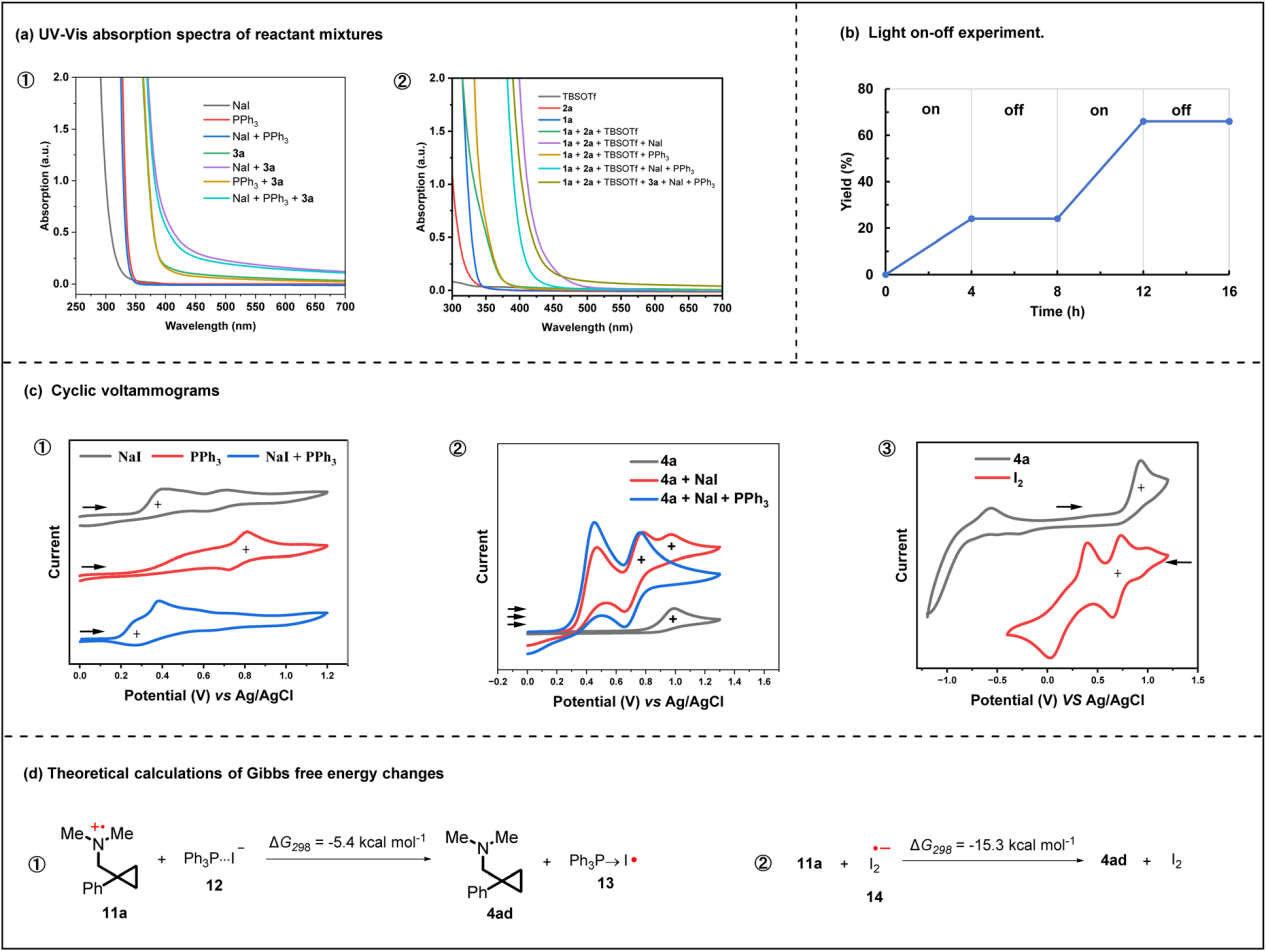
(a) UV-vis spectroscopic absorption experiments on NaI, PPh_3_, 1a, 2a, 3a, TBSOTf and their mixtures. (b) Light on/off experiments on model reactions. (c) Cyclic voltammetry studies. The black arrows indicate the initial scan direction. (d) Computational studies of the termination step of the aminium radical cation.

We then performed UV-visible (UV-vis) spectroscopic absorption experiments on various combinations of reaction components to inspect their possible interactions. An observable redshift of absorption onset occurred when NaI was mixed with redox-active ester (3a) (Fig. 1a-1). Meanwhile, NaI also induced the mixture of aldehyde (1a), amine (2a) and TBSOTf to present one comparatively larger redshift of absorption onset (Fig. 1a and 2). Without TBSOTf, the mixture of NaI, 1a and 2a still gave an obvious redshift of absorption onset compared with the mixture of 1a and 2a (Fig. S2-1). For all three groups of UV-vis experiments, addition of PPh_3_ induced a slight blueshift of absorption onset compared with the respective redshift system, but only PPh_3_ cannot have an effect on 3a, or the mixture of 1a and 2a. Consequently, the combination of NaI and PPh_3_ may activate both the redox-active ester and the mixture of amine and aldehyde under blue LED irradiation. In order to learn more about the effect of TBSOTf on our reaction, we also tested the UV-vis absorption of 3a in the presence of TBSOTf (Fig. S2-2). We found that TBSOTf would inhibit the redshift of 3a caused by NaI/PPh_3_, which meant that the photoactivation of 3a by NaI/PPh_3_ might be weakened by TBSOTf. This result may explain why the excess amount of TBSOTf to 1a and 2a will lead to a low yield (Table S1, entry 16). At last, the mixture of 1a, 2a, 3a and TBSOTf didn't show any interaction between them according to the UV-vis spectrum (Fig. S2-4).

Iodide has been reported to be capable of direct single-electron transfer (SET) reduction of carbocation, diazonium, and 1,2-diketones,^26^ and NaI/PPh_3_ may have an effect on the iminium ion during our reaction, but the process in which NaI/PPh_3_ reduces the iminium ion to an *a*-amino radical followed by final product formation through radical–radical cross-coupling (*a*-amino radical and cyclopropyl radical) is less possible because α-amino radicals from trialkylamines are readily oxidized (*E*_1/2_ = −1.03 V *vs.* SCE) and unstable under acidic conditions.^27^ The interaction between NaI and the mixture of 1a and 2a, leading to a decrease in the concentration of iminium ions, may be the reason why this reaction requires an excess amount of NaI, 1a and 2a to achieve high yield. In addition, the light on/off experiments showed that continuous irradiation was necessary for this transformation, and the radical chain mechanism is unlikely (Fig. 1b). From all these results, the two sequential steps of the mechanism, NaI/PPh_3_ initiating the radical generation from 3a under light irradiation and the generated cyclopropyl radical adding to the *in situ* generated iminium ions, are clear and proved now.

Next, cyclic voltammetry (CV) studies were performed on NaI (1 mM), PPh_3_ (1 mM), and their mixture (1 mM NaI and 1 mM PPh_3_) in acetonitrile (Fig. 1c-1). There was a noticeable increase in the current density and cathodic shift of the onset potential for the mixture of NaI and PPh_3_, which suggested that complexation of NaI and PPh_3_ in acetonitrile would form one more easily oxidizable species that should be PPh_3_⋯I^−^ (12) (the complexation of NaI and PPh_3_ is exergonic by 4.6 kcal mol^−1^ in acetonitrile).^21^ Therefore, even though PPh_3_ has not shown an obvious effect on 3a in UV-vis experiments, PPh_3_ still should engage in the initial reduction of 3a. Similarly, 12 may also reduce aminium radical cations 11a to yield the final product 4ad and PPh_3_ → I˙ (13) radical, which can be terminated by another 13, and these are supported by our computational studies (Fig. 1d-1, and see the SI). Additionally, the CV shows that the first oxidation wave of 4a can be suppressed by the mixture of NaI and PPh_3_, which means that NaI/PPh_3_ should be able to reduce 11a (Fig. 1c-2). On the other hand, CV studies revealed that the product 4a exhibited one irreversible oxidation wave with half-wave potentials *E*^ox^_1/2_ = +0.84 V (*versus* Ag/AgCl; Fig. 1c and 3), and iodine (I_2_) had the first reduction waves at *E*^red^_1/2_ = +0.74 V (*versus* Ag/AgCl; Fig. 1c and 3). These results indicated that aminium radical cations (11) were also able to be reduced by the anionic diiodide radical (I_2_˙^−^), which was in accord with the Maulide group's report.^28^ Meanwhile, the computational studies revealed that the single electron transfer from I_2_˙^−^ to aminium radical cations was a highly exergonic process (Δ*G*_298_(11a → 4ad) = −15.3 kcal mol^−1^; (Fig. 1d and 2). In our system, I_2_˙^−^ (14) may come from the reaction between 12 and 13 (see the SI). However, due to the large amounts of NaI and PPh_3_, we prefer that PPh_3_⋯I^−^ works as an electron donor for 11 in the secondary SET process of this transformation.

**Scheme 5:**
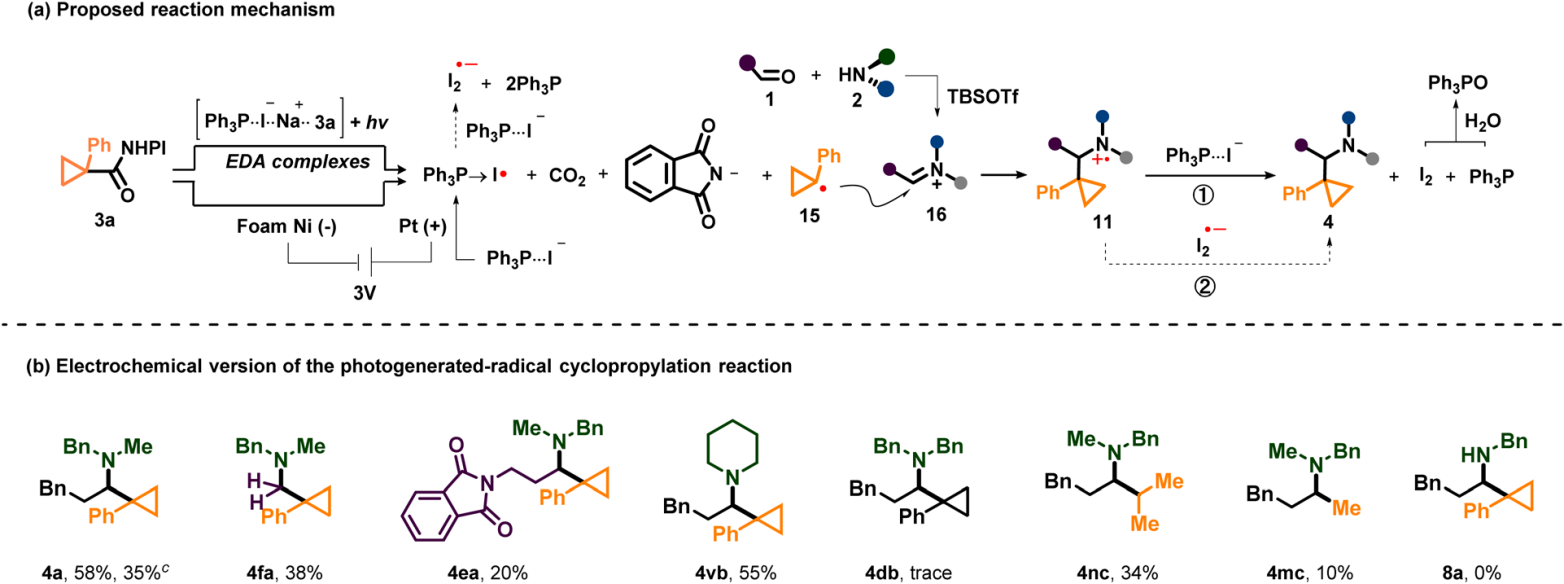
(a) Mechanistic proposal. (b) Preliminary results of the electrochemical version of the photogenerated-radical cyclopropylation. Reaction conditions: Pt plate anode, foam Ni cathode, 3.0 V, undivided cell, 1 (0.4 mmol, 2.0 equiv), 2 (0.4 mmol, 2.0 equiv.), 3 (0.2 mmol, 1.0 equiv.), NaI (0.4 mmol, 2.0 equiv.), PPh_3_ (0.4 mmol, 2.0 equiv), TBSOTf (0.4 mmol, 2.0 equiv), (*n*-Bu)_4_NBF_4_ (0.1 M), MeCN (4 mL), rt, N_2_, 1 h, and then 1 (0.2 mmol), 2 (0.2 mmol), PPh_3_ (0.2 mmol), TBSOTf (0.2 mmol) were added, 1 h. (c) Without electrolyte.

Taking into account all the results obtained from the aforementioned mechanistic studies and previous literature,^16,21,28^ we propose the mechanism outlined in Scheme 5a. Initially, visible-light irradiation of the transiently formed EDA complexes leads to electron transfer from iodide to the *N*-phthalimide moiety of 3a to further generate a cyclopropyl radical (15) through extrusion of carbon dioxide and the phthalimide anion. Then, the resulting 15 adds to the positively charged alkyl iminium ions (16) to give the amine radical cation 11, which soon undergoes a SET reduction with two possible electron donors including PPh3⋯I^−^ (12) and I_2_˙^−^ (14) to deliver the final product 4.

Due to its inherent tunability and scalability, we envisioned that electrochemistry could be applied to our reaction successfully. Based on the proposed reaction mechanism of this photochemical reaction, a large number of electrolysis conditions were screened (see the SI). Finally, 4 could be obtained in moderate yields under electrolysis conditions (6 examples, 10–58% yields) (Scheme 5b). Because NaI, PPh_3_ and TBSOTf were still indispensable to get products in the electrochemical reaction (electrolyte was not indispensable), we suggested that it had a similar mechanism to the photochemical reaction except that the active ester 3 was reduced at the cathode and PPh_3_⋯I^−^ (12) was oxidized to PPh_3_ → I˙ (13) at the anode.

## Conclusions

In summary, we have developed a general photochemical method of preparing diverse α-cyclopropyl tertiary alkylamines by radical cyclopropylation using abundant feedstocks (aldehydes and amines) and easily procured cyclopropyl active esters under mild and metal-free reaction conditions. Notably, this methodology used NaI/PPh_3_ as both the photoinitiator and electron donor, and no other traditional dye- or metal complex-based photoredox catalyst and expensive sacrificial reductant was needed. Meanwhile, a preliminary electrochemical version of this photogenerated-radical cyclopropylation was also developed. Mechanistic studies indicate that the reaction proceeds *via* the addition of the cyclopropyl radical, which is generated by visible-light-induced SET during the formation of EDA complexes upon aggregation of NaI/PPh_3_ and NHPI ester, to the *in situ* generated iminium ions from amines and aldehydes to obtain the aminium radical cations, which are finally terminated by reactive intermediates derived from NaI/PPh_3_ through the secondary SET.

## Author contributions

The manuscript was written through the contributions of all authors and all authors have given approval to the final version.

## Conflicts of interest

There are no conflicts to declare.

## Supplementary Material

SC-016-D5SC06039G-s001

## Data Availability

CCDC 2491437 contains the supplementary crystallographic data for this paper.^29^ All data associated with this work can be found in the supplementary information (SI). Supplementary information: experimental procedures and compound characterization. See DOI: https://doi.org/10.1039/d5sc06039g.
