# Supplementary material for: Photogenerated-radical cyclopropylation of in situ generated iminiums mediated by NaI/PPh3: direct access to α-cyclopropyl tertiary alkylamines
Source: Chem Sci. 2025 Oct 21;16(47):22647–55. doi: 10.1039/d5sc06039g (PMC12570270; doi:10.1039/d5sc06039g)
Supplement: SC-016-D5SC06039G-s001 [file SC-016-D5SC06039G-s001.pdf]

Supplementary Information (SI) for Chemical Science.
This journal is © The Royal Society of Chemistry 2025

Supporting Information

Photogenerated-radical cyclopropylation of *in-situ* generated iminiums mediated by NaI/PPh$_3$: direct access to *α*-cyclopropyl tertiary alkylamines

*Ying Zhou, Shan Wang, Yi-Chuan Liu, Yan Liu, Fei Tan, Hongbo Dong, and Jian Wang**

Supporting Information

Table of Contents

1. General Experimental Details S3

2. Photochemistry Reaction Optimizations S4

3. Electrochemistry Reaction Optimizations S5

4. General Synthetic Procedures S6

5. Radical Scavenger Experiment by TEMPO S8

6. UV-Vis Absorption Spectroscopic Measurements S9

7. Cyclic Voltammetry Measurements of **3a** and TBSOTf S9

8. Computational Studies S10

9. X-ray crystallographic data of **4kc** S20

10. Characteristic of the Obtained New Compounds S21

11. Copies of Product ^{1}H NMR and ^{13}C NMR S40

12. References S102

1. General Experimental Details

Unless otherwise noted, reagents were purchased from Aldrich Chemical Co. (Darmstadt, Germany), Adamas-beta (Shanghai, China), and Energy Chemical (Shanghai, China) and used without further purification. All known compounds are prepared according to the literature.[1-4] 1,3-dioxoisoindolin-2-yl 1-vinylcyclopropane-1-carboxylate (**3ic**) is preaperd by corresponding carboxylic acid. All reactions were run under an inert atmosphere (N_2) unless otherwise stated, with oven-dried glassware, using standard techniques. Irradiation of the reaction mixture was achieved using a 40 W Kessil A160WE LED-Tuna blue aquarium light (setup: max blue, max intensity). Clear glass vials (3 mL) with PTFE/silicon septum lined screw caps were used as the standard reaction vessel in photochemistry reactions. The instrument for electrolysis is a multichannel potentiostat (A-BF SS-3305D) (made in China). The dimension parameter of platinum plate electrode, reticulated vitreous carbon electrode and foam nickel electrode are 10 mm × 10 mm × 0.1 mm, 10 mm × 10 mm × 5 mm and 10 mm × 10 mm × 2 mm. Other metal electrodes all are 10 mm × 10 mm × 0.5 mm. Nuclear magnetic resonance (NMR) spectra were measured on a JNM-ECZ600R/S1 (JEOL, Tokyo, Japan) or AVANCFNEO400 (Bruker, Germany). with $CDCl_3$ as solvent and recorded in ppm relative to an internal tetramethylsilane standard. The coupling constants (*J*) were quoted in Hz. The following conventions were used for multiplicities: s, singlet; d, doublet; t, triplet; q, quartet; m, multiples. High-resolution mass spectra (HRMS) were recorded on a 6520 Q-TOF MS system (Agilent, Santa Clara, CA, USA) using an electrospray (ESI) ionization source. Cyclic voltammetry measurements were performed in dry MeCN on a CHI 610E electrochemical analyzer with a three-electrode cell, using 0.1 M Bu_4NPF_6 as supporting electrolyte, AgCl/Ag as reference electrode, platinum disk as working electrode, Pt wire as counter electrode, and a scan rate at 100 mV/s (the concentration of all the compounds were 1mM in MeCN). UV-Vis spectrum was measured by UV-2450 (A10834534597CS).

2. Photochemistry Reaction Optimizations

A 3 mL PTFE/Silicone-lined septa screw cap (PP) clear glass vial equipped with a magnetic stir bar was charged with phenylpropyl aldehyde (**1a**), *N*-methylbenzylamine (**2a**), MeCN, TBSOTf, NaI, PPh_3, and phenylcyclopropane active ester (**3a**). The vial was sealed and a needle was inserted through the septa and the contents evacuated/backfilled with N_2 (3 cycles). Then it was irradiated for indicated time with the blue LEDs. Upon completion, the reaction mixture was analyzed by TLC and directly loaded onto the prepared TLC glass plate using EtOAc/P.E. as an eluent to afford product **4a**.

Table S1. Reaction optimizations of photoinduced carbonyl aminative cyclopropylation.[a]

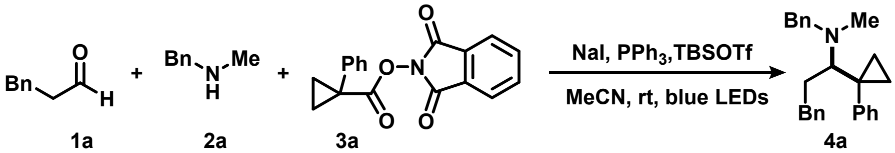

1a **2a** **3a** **4a**

Entry	Deviation from the standard conditions	Yield [%][b]
1	with 2.0 equiv. $(TMS)_3SiH$ as reductant	48 (12)[c]
2	none	87
3	one-pot method using carboxylic acid[d]	67
4	in the dark	0
5	without NaI	0
6	without PPh_3	0
7	NaI (0.25 equiv.)	45
8	PPh_3 (0.25 equiv.)	14
9	NaI (0.5 equiv.)	54
10	PPh_3 (0.5 equiv.)	64
11	in EA	48
12	in DCM	31
13	CH_3COOH instead of TBSOTf	30
14	TMSOTf instead of TBSOTf	51
15	TBSOTF (0.5equiv.)	35
16	TBSOTF (3.0equiv.)	26
17	**1a**/**2a**/**3a** = 1:1:2[e]	55
18	**6a** instead of **3a**	90
19	**7a** instead of **3a**	0
20	I_2 (0.01 equiv.) instead of NaI	20
21	I_2 (0.5 equiv.) and NaOH (0.5 equiv.) instead of NaI	45
22	I_2 (0.5 equiv.) and NaCl (0.5 equiv.) instead of NaI	33

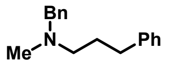

5a

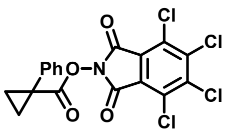

6a

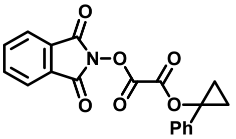

7a

[a] Reaction conditions: **1a** (0.4 mmol, 2.0 equiv.), **2a** (0.4 mmol, 2.0 equiv.), **3a** (0.2 mmol, 1.0 equiv.), NaI (0.4 mmol, 2.0 equiv.), PPh_3 (0.4 mmol, 2.0 equiv.), TBSOTf (0.4 mmol, 2.0 equiv.), MeCN (1 mL), rt, N_2, 24h, blue LEDs. [b] Isolated yield. [c] Yield of **5a** is given in parentheses. [d] (i) 1-Phenyl-1-cyclopropane-carboxylic acid (0.2 mmol), NHPI (0.22 mmol), 4-dimethylaminopyridine (5%), *N,N*-dicyclohexylcarbodiimide (0.22 mmol), MeCN, 0 ℃ to rt, 2h. (ii) standard conditions without **3a**. [e] **1a** (0.2 mmol, 1.0 equiv.).

3. Electrochemistry Reaction Optimizations

A 10 mL PTFE/Silicone-lined septa screw cap (PP) clear glass vial equipped with a magnetic stir bar and electrodes was charged with phenylpropyl aldehyde (**1a**), *N*-methylbenzylamine (**2a**), MeCN, TBSOTf, NaI, PPh_3, electrolyte and phenylcyclopropane active ester (**3a**). The vial was sealed and a needle was inserted through the rubber cap and the contents evacuated/backfilled with N_2 (3 cycles). Then the electrodes connected with the multichannel potentiostat and the reaction mixture was stirred and electrolyzed at a constant cell potential for 3h. Upon completion, the reaction mixture was analyzed by TLC and directly loaded onto the prepared TLC glass plate using EtOAc/P.E. as an eluent to afford product **4a**.

Table S2. Reaction optimizations of electrochemistry version of carbonyl aminative cyclopropylation.

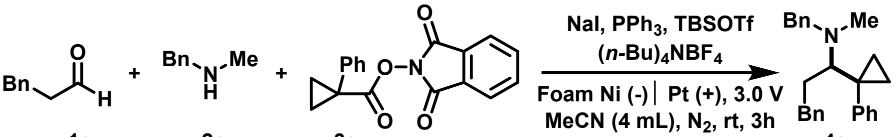

Entry	1a/2a/3a (mmol)	NaI (mmol)	PPh_3 (mmol)	Acid (mmol)	Slovent	Electrolyte	Cell potential	Electrode	Yield [%][a]
1	0.4/0.4/0.2	0.4	0.4	CH_3COOH (0.4)	MeCN (4 mL)	$(n\text{-Bu})_4NBF_4$ (0.4 mmol)	2.1 V	Foam Ni (-) | RVC (+)	23
2	0.4/0.4/0.2	0.4	0	CH_3COOH (0.4)	MeCN (4 mL)	$(n\text{-Bu})_4NBF_4$ (0.4 mmol)	2.1 V	Foam Ni (-) | RVC (+)	trace
3	0.4/0.4/0.2	0	0.4	CH_3COOH (0.4)	MeCN (4 mL)	$(n\text{-Bu})_4NBF_4$ (0.4 mmol)	2.1 V	Foam Ni (-) | RVC (+)	trace
4	0.4/0.4/0.2	0.4	0.4	TBSOTf (0.4)	MeCN (4 mL)	$(n\text{-Bu})_4NBF_4$ (0.4 mmol)	2.1 V	Foam Ni (-) | Pt (+)	19
5	0.4/0.4/0.2	0.4	0.4	TBSOTf (0.4)	MeCN (4 mL)	$(n\text{-Bu})_4NBF_4$ (0.4 mmol)	2.1 V	Pt (-) | Pt (+)	0
6	0.4/0.4/0.2	0.4	0.4	TBSOTf (0.4)	MeCN (4 mL)	$(n\text{-Bu})_4NBF_4$ (0.4 mmol)	2.1 V	RVC (-) | Pt (+)	0
7	0.4/0.4/0.2	0.4	0.4	TBSOTf (0.4)	MeCN (4 mL)	$(n\text{-Bu})_4NBF_4$ (0.4 mmol)	2.1 V	Fe (-) | Pt (+)	0
8	0.4/0.4/0.2	0.4	0.4	TBSOTf (0.4)	MeCN (4 mL)	$(n\text{-Bu})_4NBF_4$ (0.4 mmol)	2.1 V	Foam Ni (-) | Ni (+)	trace
9	0.4/0.4/0.2	0.4	0.4	TBSOTf (0.4)	MeCN (4 mL)	$(n\text{-Bu})_4NClO_4$ (0.4 mmol)	2.1 V	Foam Ni (-) | Pt (+)	21
10	0.4/0.4/0.2	0.4	0.4	TBSOTf (0.4)	MeCN (4 mL)	$(n\text{-Bu})_4NI$ (0.4 mmol)	2.1 V	Foam Ni (-) | Pt (+)	12
11	0.4/0.4/0.2	0.4	0.4	TBSOTf (0.4)	MeCN (4 mL)	$(n\text{-Bu})_4NPF_6$ (0.4 mmol)	2.1 V	Foam Ni (-) | Pt (+)	21
12	0.4/0.4/0.2	0.4	0.4	TBSOTf (0.4)	MeCN (4 mL)	None	2.1 V	Foam Ni (-) | Pt (+)	18
13	0.4/0.4/0.2	0	0.4	TBSOTf (0.4)	MeCN (4 mL)	$(n\text{-Bu})_4NBF_4$ (0.4 mmol)	2.1 V	Foam Ni (-) | Pt (+)	0
14	0.4/0.4/0.2	0.4	0	TBSOTf (0.4)	MeCN (4 mL)	$(n\text{-Bu})_4NBF_4$ (0.4 mmol)	2.1 V	Foam Ni (-) | Pt (+)	0
15	0.4/0.4/0.2	0.4	0.4	TBSOTf (0.4)	DCM (4 mL)	$(n\text{-Bu})_4NBF_4$ (0.4 mmol)	2.1 V	Foam Ni (-) | Pt (+)	0
16	0.4/0.4/0.2	0.4	0.4	TBSOTf (0.4)	DMA (4 mL)	$(n\text{-Bu})_4NBF_4$ (0.4 mmol)	2.1 V	Foam Ni (-) | Pt (+)	0
17	0.4/0.4/0.2	0.4	0.4	TBSOTf (0.4)	HFIP (4 mL)	$(n\text{-Bu})_4NBF_4$ (0.4 mmol)	2.1 V	Foam Ni (-) | Pt (+)	0
18	0.4/0.4/0.2	0.4	0.4	TBSOTf (0.4)	THF (4 mL)	$(n\text{-Bu})_4NBF_4$ (0.4 mmol)	2.1 V	Foam Ni (-) | Pt (+)	0
19	0.4/0.4/0.2	0.4	0.8	TBSOTf (0.4)	MeCN (4 mL)	$(n\text{-Bu})_4NBF_4$ (0.4 mmol)	2. 1 V	Foam Ni (-) | Pt (+)	18
20	0.4/0.4/0.2	0.4	0.4	TBSOTf (0.4)	MeCN (4 mL)	$(n\text{-Bu})_4NBF_4$ (0.4 mmol)	3 V	Foam Ni (-) | Pt (+)	25

21	0.4/0.4/0.2	0.4	0.4	TBSOTf (0.4)	MeCN (4 mL)	(n-Bu)$_4$NBF$_4$ (0.4 mmol)	5 V	Foam Ni (-) ∣ Pt (+)	0
22	0.4/0.4/0.2	0.4	0.4	TBSOTf (0.4)	MeCN (4 mL)	(n-Bu)$_4$NBF$_4$ (0.4 mmol)	1.5 V	Foam Ni (-) ∣ Pt (+)	14
23	0.4/0.4/0.2	0.4	0.4	TBSOTf (0.4)	MeCN (4 mL)	(n-Bu)$_4$NBF$_4$ (0.4 mmol)	4 V	Foam Ni (-) ∣ Pt (+)	0
24	0.4/0.4/0.2	0.4	0.4	TBSOTf (0.4)	MeCN (4 mL)	(n-Bu)$_4$NBF$_4$ (0.4 mmol)	3.5 V	Foam Ni (-) ∣ Pt (+)	14
25	0.4/0.4/0.2	0.4	0.8	TBSOTf (0.4)	MeCN (4 mL)	(n-Bu)$_4$NBF$_4$ (0.4 mmol)	3 V	Foam Ni (-) ∣ Pt (+)	10
26	0.2/0.2/0.4	0.4	0.4	TBSOTf (0.4)	MeCN (4 mL)	(n-Bu)$_4$NBF$_4$ (0.4 mmol)	3 V	Foam Ni (-) ∣ Pt (+)	21
27	0.4/0.4/0.2	0.4	PCy$_3$ (0.4)	TBSOTf (0.4)	MeCN (4 mL)	(n-(0.4 mmol)	3 V	Foam Ni (-) ∣ Pt (+)	0
28	1.0/1.0/0.2	0.4	0.4	TBSOTf (1.0)	MeCN (2 mL)	(n-Bu)$_4$NBF$_4$ (0.4 mmol)	3 V	Foam Ni (-) ∣ Pt (+)	30
29	0.4/0.4/0.2	0.4	0.4	TBSOTf (0.4)	MeCN (4 mL)	(n-Bu)$_4$NBF$_4$ (0.4 mmol)	1.5 V (24 h)	Foam Ni (-) ∣ Pt (+)	30
30	0.4/0.4/0.2	0.4	**P-1**	TBSOTf (0.4)	MeCN (4 mL)	(n-Bu)$_4$NBF$_4$ (0.4 mmol)	3 V	Foam Ni (-) ∣ Pt (+)	0
31	0.4/0.4/0.2	0.4	**P-2**	TBSOTf (0.4)	MeCN (4 mL)	(n-Bu)$_4$NBF$_4$ (0.4 mmol)	3 V	Foam Ni (-) ∣ Pt (+)	0
32	1.0/1.0/0.2	0.4	0.4	TBSOTf (1.0)	MeCN (4 mL)	(n-Bu)$_4$NBF$_4$ (0.4 mmol)	1.5 V (24 h)	Foam Ni (-) ∣ Pt (+)	30
33	0.4/0.4/0.2	0.2 + I_2(0.2)	0.4	TBSOTf (0.4)	MeCN (4 mL)	(n-Bu)$_4$NBF$_4$ (0.4 mmol)	3 V	Foam Ni (-) ∣ Pt (+)	0
34	0.4/0.4/0.2	0.8	0.4	TBSOTf (0.4)	MeCN (4 mL)	(n-Bu)$_4$NBF$_4$ (0.4 mmol)	3 V	Foam Ni (-) ∣ Pt (+)	<30
35	0.4/0.4/0.2	0.4 + CuI (0.04)	0.4	TBSOTf (0.4)	MeCN (4 mL)	(n-Bu)$_4$NBF$_4$ (0.4 mmol)	3 V	Foam Ni (-) ∣ Pt (+)	<30
36	0.4/0.4/0.2	0.4	0.4	TBSOTf (0.4)	MeCN (4 mL)	(n-Bu)$_4$NBF$_4$ (0.4 mmol)	3 V	Cu (-) ∣ Pt (+)	0
37	0.4/0.4/0.2	0.4	0.4	TBSOTf (0.4)	MeCN (2 mL)	(n-Bu)$_4$NBF$_4$ (0.4 mmol)	3 V	Foam Ni (-) ∣ Pt (+)	<30
38	0.4/0.4/0.2	0.4	0.4	TBSOTf (0.4)	MeCN (4 mL)	(n-Bu)$_4$NBF$_4$ (0.8 mmol)	3 V	Foam Ni (-) ∣ Pt (+)	<30
39	0.4/0.4/0.2	0.4	0.4	TBSOTf (0.4)	MeCN (4 mL)	(n-Bu)$_4$NBF$_4$ (0.2 mmol)	3 V	Foam Ni (-) ∣ Pt (+)	<30
40	0.6/0.6/0.2	0.4	0.4	TBSOTf (0.4)	MeCN (1 mL)	(n-Bu)$_4$NBF$_4$ (0.1 mmol)	1.5 V	Foam Ni (-) ∣ Pt (+)	<30
41	0.8/0.8/0.2	0.4	0.4	TBSOTf (0.8)	MeCN (8 mL)	(n-Bu)$_4$NBF$_4$ (0.8 mmol)	3 V	Foam Ni (-) ∣ Pt (+)	0
42[b]	0.4/0.4/0.2	0.4	0.4	TBSOTf (0.4)	MeCN (4 mL)	(n-Bu)$_4$NBF$_4$ (0.4 mmol)	3 V	Foam Ni (-) ∣ Pt (+)	58
43[b]	0.4/0.4/0.2	0.4	0.4	TBSOTf (0.4)	MeCN (4 mL)	None	3 V	Foam Ni (-) ∣ Pt (+)	35

[a] Isolated yield. [b] After 1h, **1a** (0.2 mmol), **2a** (0.2 mmol), PPh_3 (0.2 mmol) and TBSOTf (0.2 mmol) were added into the reaction mixture, then the electrochemistry reaction continued under N_2. **P-1**:Tris(2,4,6-Trimethoxyphenyl)Phosphine. **P-2**: Tris(4-Methoxy-phenyl)Phosphine

4. General Synthetic Procedures

4.1 General Procedure A: Photoinduced carbonyl aminative cyclopropylation

A 3 mL PTFE/Silicone-lined septa screw cap (PP) clear glass vial equipped with a magnetic stir bar was charged with amine (0.4 mmol), (If the amine is hydrochloride salt, it was freed beforehand by Et_3N (0.4 mmol) in MeCN (1mL)), aldehyde (0.4 mmol), MeCN (1 mL), PPh_3 (0.4 mmol), NaI (0.4 mmol), TBSOTf (0.4 mmol) and active ester (0.2 mmol). The vial was sealed and a needle was inserted through the septa and the contents evacuated/backfilled with N_2 (3 cycles). Then it was irradiated for 24h with the Kessil lamp with vigorous stirring. Upon completion, the reaction was quenched by $NaHCO_3$ (aq) and extracted by EA for three times. The combined organic solution was washed with

sodium chloride solution and dried over anhydrous sodium sulfate. After removal of the solvent, the residue was purified by flash column chromatography (silica gel) to afford the desired product.

4.2 General Procedure B: Electrochemistry reaction

A 10 mL PTFE/Silicone-lined septa screw cap (PP) clear glass vial equipped with a magnetic stir bar and electrodes (Foam Ni (-) ｜ Pt (+)) was charged with amine (0.4 mmol), aldehyde (0.4 mmol), MeCN (4 mL), PPh_3 (0.4 mmol), NaI (0.4 mmol), TBSOTf (0.4 mmol), (n-Bu)$_4$NBF$_4$ (0.1 M) and active ester (0.2 mmol). The vial was sealed and a needle was inserted through the rubber cap and the contents evacuated/backfilled with N_2 (3 cycles). Then the electrodes connected with the multichannel potentiostat and the reaction mixture was stirred and electrolyzed at a constant cell potential of 3.0 V for 1h. Then PPh_3 (0.2 mmol), aldehyde (0.2 mmol), amine (0.2 mmol), TBSOTf (0.2 mmol) were added, and the resulting mixture was stirred and electrolyzed at same cell potential for 1h again. Upon completion, the reaction was quenched by $NaHCO_3$(aq) and extracted by EA for three times. The combined organic solution was washed with sodium chloride solution and dried over anhydrous sodium sulfate. After removal of the solvent, the residue was purified by flash column chromatography (silica gel) to afford the desired product.

4.3 One-Pot Method by Directly Using Carboxylic Acids to Prepare 4a:

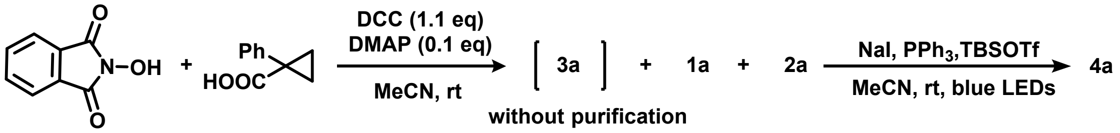

To a solution of N-hydroxyphthalimide (1.1 equiv.), 1-phenylcyclopropane-1-carboxylic acid (0.2 mmol, 1.0 equiv.) and 4-dimethylaminopyridine (DMAP) (0.1 equiv.) in MeCN (0.2 M) was added dicyclohexylcarbodiimide (DCC) (1.1 equiv.) at 0 °C. The resulting mixture was stirred at room temperature for 2 hours. Then, PPh_3 (0.4 mmol), NaI (0.4 mmol), phenylpropyl aldehyde (0.4 mmol), N-methylbenzylamine (0.4 mmol) and TBSOTf (0.4 mmol) was directly added, and the mixture was irradiated by blue LEDs and stirred at room temperature under N_2 atmosphere for 24 hours. Then, the reaction was quenched by $NaHCO_3$ (aq) and extracted by EA for three times. The combined organic solution was washed with sodium chloride solution and dried over anhydrous sodium sulfate. After removal of the solvent, the residue was purified by flash column chromatography (silica gel, PE/EA = 5:1), to afford the desired product **4a** (47 mg, 67%).

4.4 Synthesis of 1,3-dioxoisoindolin-2-yl 1-vinylcyclopropane-1-carboxylate (3ic):

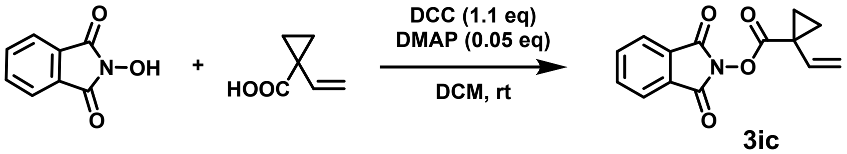

3ic

To a solution of N-hydroxyphthalimide (213 mg, 1.1 equiv.), 1-vinylcyclopropane-1-carboxylic acid (150 mg, 1.0 equiv.)[5] and 4-dimethylaminopyridine (DMAP) (14.6 mg, 0.1 equiv.) in DCM (0.2 M) was added dicyclohexylcarbodiimide (DCC) (269 mg, 1.1 equiv.) at 0 °C. The resulting mixture was stirred at room temperature and monitored by TLC technique. After the alkyl carboxylic acid was consumed, the mixture was filtered through a pad of silica gel and rinsed with additional DCM, filtrate was collected and concentrated under reduced pressure. After removal of the solvent, the residue was purified by flash column chromatography (silica gel, PE/EA = 5:1), to afford the desired product **3ic** (270 mg, 78%) as a white solid. mp 107.4-108.9 °C; ^{1}H NMR (400 MHz, $CDCl_3$): δ 7.88 (dd, J = 5.5, 3.1 Hz, 2H), 7.80-7.75 (m, 2H), 6.47 (dd, J = 17.2, 10.7 Hz, 1H), 5.15-5.08 (m, 2H), 1.78 (q, J =

4.3 Hz, 2H), 1.34 (q, 2H). ^{13}C NMR (101 MHz, $CDCl_3$): δ 170.25, 161.98, 134.74, 133.84, 128.96, 123.95, 114.47, 24.13, 19.31. HRMS(ESI): $[M+Na]^+$ calcd. for $C_{14}H_{11}NNaO_4^+$: m/z =280.0580; found, 280.0566.

5. Radical Scavenger Experiment by TEMPO

5.1 **Table S3**. Photoinduced model reaction in the presence of TEMPO[a]

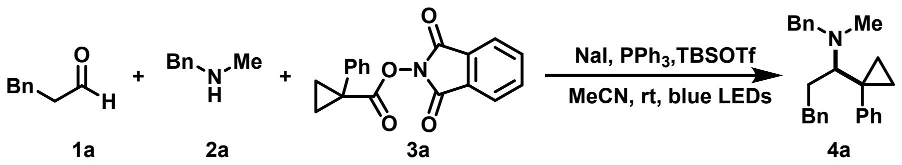

Entry	Addition of TEMPO (equiv.)	Yeild [%][b]
1	0.5	56
2	1.0	11
3	2.0	0

[a] Reaction conditions:**1a** (0.4 mmol, 2.0 equiv.), **2a** (0.4 mmol, 2.0 equiv.), **3a** (0.2 mmol, 1.0 equiv.), NaI (0.4 mmol, 2.0 equiv.), PPh_3 (0.4 mmol, 2.0 equiv.), TBSOTf (0.4 mmol, 2.0 equiv.), MeCN (1 mL), TEMPO, rt, N_2, 24h, blue LEDs. [b] Isolated yield.

5.2 Mass spectrometry for detection of reaction intermediates

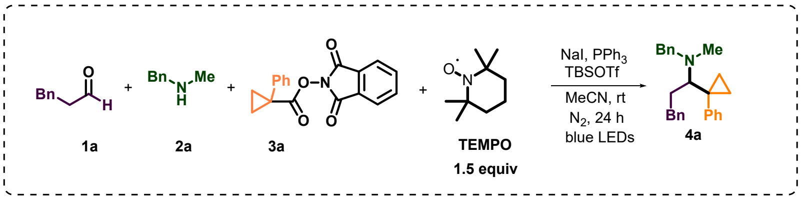

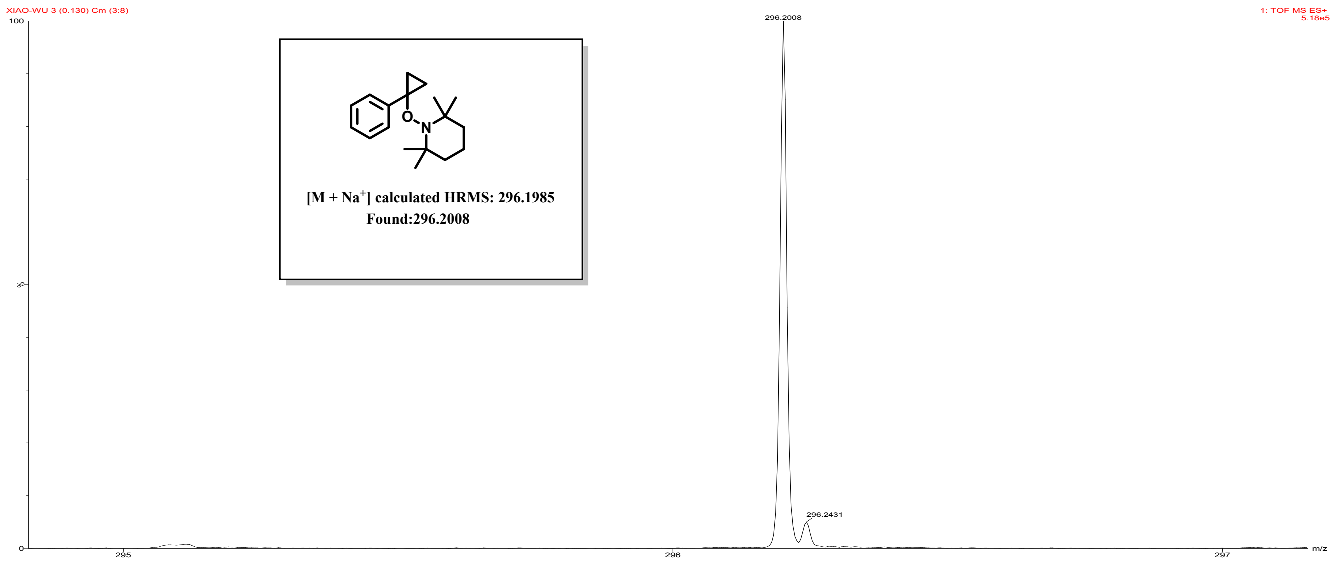

/

Figure S1: Mass spectrometry for detection of reaction intermediates

6. UV-Vis Absorption Spectroscopic Measurements

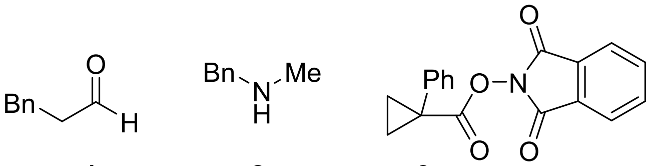

1a **2a** **3a**

Stock solutions of **1a** (0.1 M), **2a** (0.1 M), **3a** (0.05 M), PPh_3 (0.1 M), NaI (0.1 M) and TBSOTf (0.1 M) were prepared in the presence of air using MeCN as solvent.

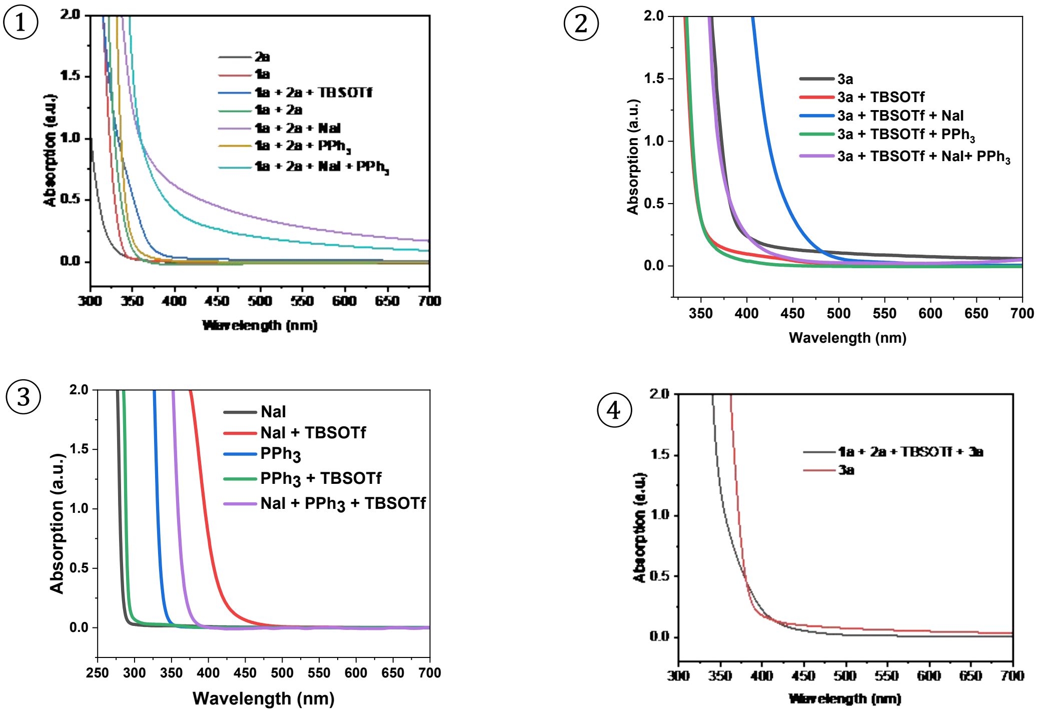

Figure S2: UV/vis absorption spectra of the combination between different starting materials recorded in MeCN as solvent

7. Cyclic Voltammetry Measurements

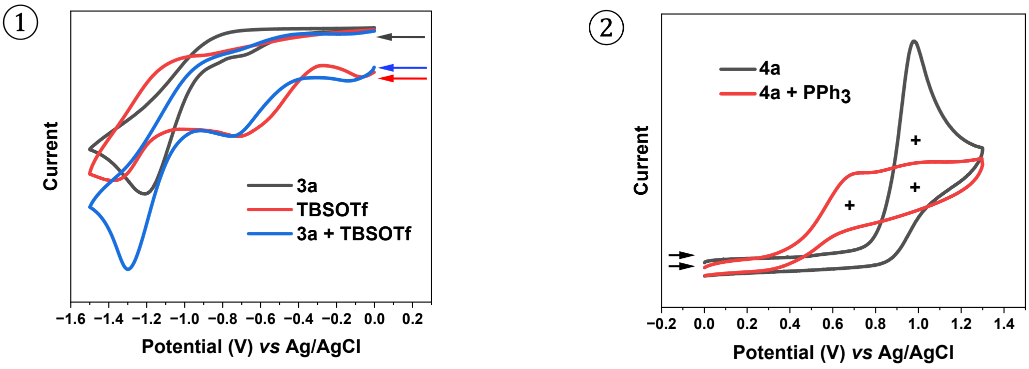

8. Computational studies

Density functional theory (DFT) [1] calculations were performed using using *Gaussian 16* [2] and the *Avogadro* [3] was used to generate input geometries and visualize output structures. Geometry optimizations and frequency calculations were performed using the M06-2X functional [4] and an atom-pairwise dispersion correction (D3) [5,6] with def2-SVP basis set [7,8]. The single point energy were calculated with def2-TZVP basis set [9,10]. For comparative purpose and to model solvation effect, the calculations were carried out in MeCN as solvent at the same level of theory, by applying the most commonly used the polarizable continuum SMD [11,12] model with the refined parameters for iodine (SMD18) [13]. All stationary points were characterized as minima or transitions states based on normal vibrational mode analysis. The Gibbs free energies are calculated at 298.15 K and concentration correction to 0.01 M performed using the Shermo program.[14] The zero-point energy (ZPE) correction factor of 0.97 obtained at the M06-2X/def2-TZVP level was approximately adopted as the correction factor for the M06-2X/def2-SVP calculations.[15]

[1] R. G. Parr, Y. Weitao, Density-Functional Theory of Atoms and Molecules, vol. 16 of International Series of Monographs on Chemistry (*Oxford Univ. Press,* **1989**).

[2] M. J. Frisch, G. W. Trucks, H. B. Schlegel, G. E. Scuseria, M. A. Robb, J. R. Cheeseman, G. Scalmani, V. Barone, G. A. Petersson, H. Nakatsuji, X. Li, M. Caricato, A. V. Marenich, J. Bloino, B. G. Janesko, R. Gomperts, B. Mennucci, H. P. Hratchian, J. V. Ortiz, A. F. Izmaylov, J. L. Sonnenberg, D. Williams-Young, F. Ding, F. Lipparini, F. Egidi, J. Goings, B. Peng, A. Petrone, T. Henderson, D. Ranasinghe, V. G. Zakrzewski, J. Gao, N. Rega, G. Zheng, W. Liang, M. Hada, M. Ehara, K. Toyota, R. Fukuda, J. Hasegawa, M. Ishida, T. Nakajima, Y. Honda, O. Kitao, H. Nakai, T. Vreven, K. Throssell, J. A. Montgomery, Jr., J. E. Peralta, F. Ogliaro, M. J. Bearpark, J. J. Heyd, E. N. Brothers, K. N. Kudin, V. N. Staroverov, T. A. Keith, R. Kobayashi, J. Normand, K. Raghavachari, A. P. Rendell, J. C. Burant, S. S. Iyengar, J. Tomasi, M. Cossi, J. M. Millam, M. Klene, C. Adamo, R. Cammi, J. W. Ochterski, R. L. Martin, K. Morokuma, O. Farkas, J. B. Foresman, and D. J. Fox, Gaussian, Inc., Wallingford CT, 2016.

[3] M. D. Hanwell, D. E. Curtis, D. C. Lonie, T. Vandermeersch, E. Zurek, G. R. Hutchison, Avogadro: An advanced semantic chemical editor, visualization, and analysis platform. *J Cheminform*, **2012**, *4*, 17.

[4] Y. Zhao, D. G. Truhlar, The M06 suite of density functionals for main group thermochemistry, thermochemical kinetics, noncovalent interactions, excited states, and transition elements: two new functionals and systematic testing of four M06-class functionals and 12 other functionals. *Theor. Chem. Acc.* **2008**, *120*, 215–241.

[5] S. Grimme, S. Ehrlich, L. Goerigk, Effect of the damping function in dispersion corrected density functional theory. *J. Comput. Chem.* **2011**, *32*, 1456–1465.

[6] S. Grimme, J. Antony, S. Ehrlich, H. Krieg, A consistent and accurate ab initio parametrization of density functional dispersion correction (DFT-D) for the 94 elements H-Pu. *J. Chem. Phys.* **2010,** *132*, 154104.

[7] A. Schaefer, H. Horn, R. Ahlrichs. Fully optimized contracted Gaussian-basis sets for atoms Li to Kr. *J. Chem. Phys.* **1992**, *97*, 2571–2577.

[8] A. Schaefer, C. Huber, R. Ahlrichs. Fully optimized contracted Gaussian-basis sets of triple zeta valence quality for atoms Li to Kr. *J. Chem. Phys.* **1994**, *100*, 5829–5835.

[9] R. A. Weigend, Balanced basis sets of split valence, triple zeta valence and quadruple zeta valence quality for H to Rn: Design and assessment of accuracy. *Phys. Chem. Chem. Phys.* **2005**, *7*, 3297–3305.

[10] F. Weigend, Accurate Coulomb-fitting basis sets for H to Rn. *Phys. Chem. Chem. Phys.* **2006**, *8*, 1057-1065.

[11] E. Cancès, B. Mennucci, J. Tomasi, A new integral equation formalism for the polarizable continuum model: Theoretical background and applications to isotropic and anisotropic dielectrics. *J. Chem. Phys.* **1997**, *107*, 3032–3041

[12] A. V: Marenich, C. J. Cramer, D. G: Truhlar, Universal solvation model based on solute electron density and on a continuum model of the solvent defined by the bulk dielectric constant and atomic surface tensions. *J. Phys. Chem. B* **2009**, *113*, 6378–6396.

[13] E. Engelade, N. Schulz, F. Heinen, S. M. Huber, D. G: Truhlar, Refined SMD parameters for bromine and iodine accurately model halogen-bonding interactions in solution. *Chem. Eur. J.* **2018**, *24*, 15983–15987.

[14] T. Lu, Q. Chen, Shermo: A general code for calculating molecular thermodynamic properties. *Comput. Theor. Chem.* **2021**, *1200*, 113249

[15] I. M. Alecu, J. Zheng, Y. Zhao, D. G. Truhlar, Computational thermochemistry: Scale factor databases and scale factors for vibrational frequencies obtained from electronic model chemistries. *J. Chem. Theory Comput.* **2010**, *6*, 2872–2887.

Table S4. Cartesian coordinates, energy, enthalpy, and optimized structure of compounds

Compound	Sturcture	U(0) (Hartree)	H(T) (Hartree)	G(T) (Hartree)
**12**	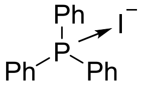	-1333.8200576	-1333.8007446	-1333.8669592

Cartesian Coordinates (CH_3CN)	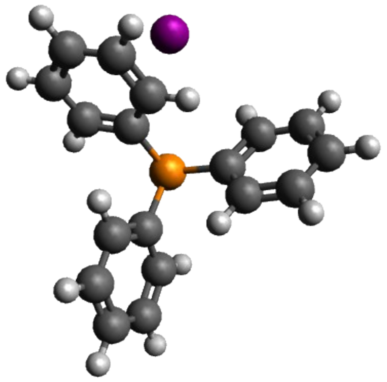

P	0.84141500	-0.01152200	-0.82830700
I	-3.55466000	-0.00646000	-0.78697000
C	0.98876100	1.63018600	-0.00051700
C	2.07924100	2.01374700	0.79093400
C	-0.07091900	2.53092700	-0.18191100
C	2.10423400	3.27166800	1.39554200
H	2.91579400	1.32784300	0.94212800
C	-0.05037000	3.78317700	0.43235000
H	-0.92323300	2.24574700	-0.80515100
C	1.03935100	4.15665200	1.22061000
H	2.96023500	3.55954200	2.00884000
H	-0.88472200	4.47191200	0.28761100
H	1.06054200	5.13924900	1.69552600
C	2.57834700	-0.62753400	-0.73153500
C	3.06861200	-1.44942700	0.29129400
C	3.44622200	-0.23399100	-1.76103000
C	4.40219100	-1.86355800	0.28602100
H	2.40973000	-1.76796800	1.10204900
C	4.78072600	-0.63800900	-1.75948300
H	3.07226600	0.39671500	-2.57251400
C	5.26083800	-1.45718600	-0.73573100
H	4.77162500	-2.50490600	1.08849700
H	5.44512300	-0.32009900	-2.56515300
H	6.30293400	-1.78175900	-0.73702200
C	0.01345600	-1.01969800	0.47289600
C	-0.34271200	-0.53468700	1.73595100
C	-0.33326400	-2.33463300	0.12608800
C	-1.03462300	-1.35034800	2.63482700
H	-0.09069500	0.48797500	2.02427300
C	-1.01028800	-3.15187600	1.02850300
H	-0.07726900	-2.71975200	-0.86529700
C	-1.36818000	-2.65813600	2.28539600
H	-1.31351200	-0.95762400	3.61449200

H	-1.27219300	-4.17310400	0.74546900
H	-1.91062000	-3.29219300	2.98902600

Compound	Sturcture	U(0) (Hartree)	H(T) (Hartree)	G(T) (Hartree)
**13**	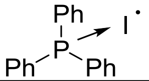	-1333.6387964	-1333.6202621	-1333.6845113

Cartesian Coordinates (CH_3CN)	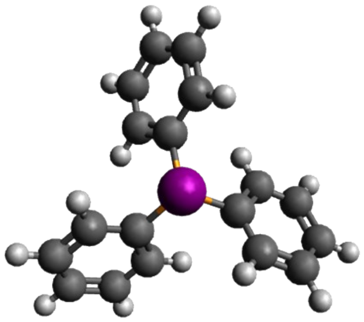

P	-0.09245300	0.00225100	-0.00082000
I	2.87453200	-0.13835800	-0.05000500
C	-0.67577300	1.63276000	-0.53574900
C	-1.66306700	2.33959400	0.16297200
C	-0.09587400	2.18809700	-1.68614500
C	-2.07279900	3.59120900	-0.29696200
H	-2.11649400	1.91385300	1.06046000
C	-0.51683800	3.43481300	-2.14302400
H	0.68575300	1.64343400	-2.22240200
C	-1.50345700	4.13759800	-1.44767700
H	-2.84382600	4.13933700	0.24690400
H	-0.06692600	3.86302100	-3.04010700
H	-1.82714800	5.11732100	-1.80324800
C	-0.73013600	-0.28972500	1.67021300
C	-1.77621200	-1.18514600	1.92732700
C	-0.12836900	0.40935500	2.72757700
C	-2.22180200	-1.36962600	3.23644900
H	-2.24735200	-1.73412400	1.10945500
C	-0.58527900	0.22548600	4.03034800
H	0.69798300	1.09715000	2.52909600
C	-1.63036600	-0.66548400	4.28564900
H	-3.03864500	-2.06548500	3.43463600
H	-0.11864100	0.77361200	4.85033900
H	-1.98247000	-0.81333400	5.30802200
C	-0.82765700	-1.23782900	-1.09881700
C	-1.83392900	-0.91989900	-2.01998800
C	-0.34400000	-2.55215200	-1.01288500
C	-2.35943500	-1.91794400	-2.84102000
H	-2.21216000	0.10173800	-2.09401300
C	-0.88046800	-3.54403900	-1.83026800
H	0.45231600	-2.79719300	-0.30500000
C	-1.88670200	-3.22711300	-2.74586600
H	-3.14543700	-1.66920500	-3.55593700
H	-0.50607100	-4.56637400	-1.75821300
H	-2.30128700	-4.00427100	-3.39021600

Compound	Sturcture	U(0) (Hartree)	H(T) (Hartree)	G(T) (Hartree)
**14-2**	I^-	-297.8168203	-297.8144599	-297.8336686

Cartesian Coordinates (CH_3CN)	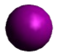

I	0.00000000	0.00000000	0.00000000

Compound	Sturcture	U(0) (Hartree)	H(T) (Hartree)	G(T) (Hartree)
**14-1**	I_2	-595.2799858	-595.2761625	-595.3056828

Cartesian Coordinates (CH_3CN)	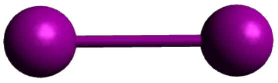

I	0.00000000	0.00000000	1.33093300
I	0.00000000	0.00000000	-1.33093300

Compound	Sturcture	U(0) (Hartree)	H(T) (Hartree)	G(T) (Hartree)
**14**	$I_2^{-\bullet}$	-595.4453646	-595.4413547	-595.4724040

Cartesian Coordinates (CH_3CN)	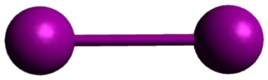

I	0.00000000	0.00000000	1.60156800
I	0.00000000	0.00000000	-1.60156800

Compound	Sturcture	U(0) (Hartree)	H(T) (Hartree)	G(T) (Hartree)
**17**	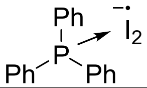	-1631.4557232	-1631.4345020	-1631.5064379

Cartesian Coordinates (CH_3CN)	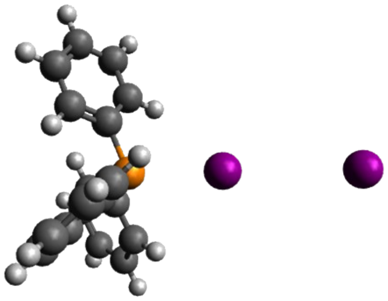

P	1.61925900	-0.00012000	-0.00146000
C	2.31143700	-0.52144800	-1.59705400
C	3.34195200	-1.46468300	-1.69818300
C	1.76745700	0.04812100	-2.75818100
C	3.82994800	-1.82514000	-2.95465900
H	3.76824000	-1.91520100	-0.79946700
C	2.26616200	-0.31040200	-4.00844800
H	0.95259300	0.77308900	-2.68162900
C	3.29611300	-1.24865400	-4.10757200
H	4.63425700	-2.55879800	-3.03032000
H	1.84328800	0.13787000	-4.90893400
H	3.68103000	-1.53378400	-5.08820400

C	2.28655900	-1.12323400	1.25983800
C	1.72791200	-2.40716900	1.34816800
C	3.31501100	-0.74690600	2.13311200
C	2.21030800	-3.31238200	2.29077400
H	0.91434400	-2.69694400	0.67766500
C	3.78661200	-1.65630300	3.08032500
H	3.75235000	0.25188500	2.07294100
C	3.23833700	-2.93691200	3.15852100
H	1.77628700	-4.31154500	2.35373000
H	4.58950200	-1.36122600	3.75792500
H	3.61065400	-3.64489800	3.90097700
C	2.28496300	1.65180600	0.35274400
C	1.71378300	2.36762100	1.41569500
C	3.32228000	2.22179300	-0.39622100
C	2.19180500	3.63601600	1.73677200
H	0.89374600	1.92987800	1.99131900
C	3.78949600	3.49640500	-0.07391100
H	3.76977200	1.67215000	-1.22693100
C	3.22828700	4.20196500	0.99093500
H	1.74775900	4.18800100	2.56670800
H	4.59905900	3.93742100	-0.65783900
H	3.59693800	5.19877100	1.23907300
I	-1.35772000	-0.00782400	-0.01937900
I	-5.58321000	-0.00138000	-0.00290400

Compound	Sturcture	U(0) (Hartree)	H(T) (Hartree)	G(T) (Hartree)
**17-1**	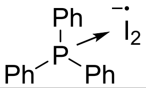	-1631.4595375	-1631.4384426	-1631.5091552

Cartesian Coordinates (CH_3CN)

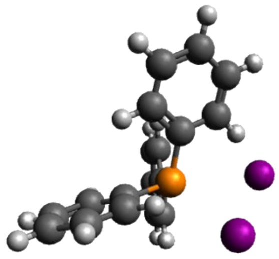

C	2.72510800	0.19648900	0.65567900
C	3.11137600	1.11581100	1.63937900
C	3.65819400	-0.72036500	0.14757300
C	4.42748900	1.12120300	2.10238200
H	2.38985900	1.82976300	2.04170400
C	4.97230400	-0.70169500	0.60837400
H	3.35323100	-1.44807000	-0.60927800
C	5.35702300	0.21717700	1.58780800
H	4.72658200	1.83942800	2.86751600
H	5.69663300	-1.41260500	0.20765100
H	6.38534900	0.22546400	1.95314900
C	-0.00512200	1.20342800	1.02895000
C	-0.37778800	0.74406800	2.30098100
C	-0.47706300	2.43655600	0.56212500

C	-1.20584500	1.52363000	3.10484600
H	-0.02624100	-0.22851200	2.65589600
C	-1.31292800	3.20685300	1.37033500
H	-0.19682800	2.79580200	-0.43014500
C	-1.67770000	2.75252300	2.63791800
H	-1.49543500	1.16438800	4.09361900
H	-1.68177900	4.16648100	1.00451800
H	-2.33654500	3.35640500	3.26432000
C	1.04653200	0.78292300	-1.67157600
C	-0.11782500	0.62729800	-2.43945000
C	2.16829700	1.41810400	-2.21961400
C	-0.16224600	1.12379300	-3.73998800
H	-0.99119800	0.12377600	-2.01415800
C	2.11799000	1.90324400	-3.52676900
H	3.07801900	1.54123500	-1.62851700
C	0.95594500	1.75907400	-4.28535400
H	-1.07145900	1.00745200	-4.33191500
H	2.99200600	2.39996100	-3.95126900
H	0.92131200	2.14112200	-5.30706900
P	1.03150600	0.11922100	0.01482900
I	0.10413600	-2.70276600	0.01786800
I	-3.65958100	-0.04348100	-0.00315300

**Compound**	**Sturcture**	**U(0) (Hartree)**	**H(T) (Hartree)**	**G(T) (Hartree)**
**18-1**	PPh_3	-1036.0019069	-1035.9858830	-1036.0429290

Cartesian Coordinates (CH_3CN)

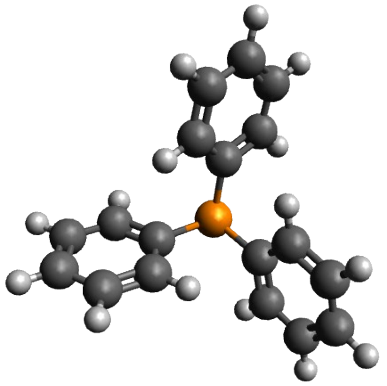

P	-0.00006300	-0.00035000	-1.26218400
C	1.34004200	-0.96361200	-0.43917500
C	1.64476400	-2.21844100	-0.98768300
C	2.05462800	-0.52050600	0.68076600
C	2.62942100	-3.02327100	-0.41623000
H	1.10312300	-2.57006200	-1.87038400
C	3.04912900	-1.32218300	1.24510400
H	1.83522100	0.45423200	1.12201000
C	3.33586300	-2.57456000	0.70148300
H	2.85203800	-3.99974800	-0.85040900
H	3.59984300	-0.96533100	2.11749600
H	4.11264600	-3.19956200	1.14576800
C	0.16474600	1.64212600	-0.44020900
C	-0.59214200	2.04984600	0.66517700
C	1.11457600	2.52434500	-0.97696800

C	-0.39578800	3.31341000	1.22678000
H	-1.33893800	1.38009400	1.09714700
C	1.31892000	3.78032800	-0.40759200
H	1.70261600	2.22280200	-1.84848000
C	0.56095100	4.17844200	0.69560300
H	-0.99294800	3.62005700	2.08764000
H	2.06576800	4.45385800	-0.83202300
H	0.71353800	5.16452300	1.13800000
C	-1.50431200	-0.67928800	-0.43945700
C	-2.74315700	-0.31964400	-0.99085900
C	-1.47798900	-1.51634400	0.68300800
C	-3.93256600	-0.77013400	-0.41973300
H	-2.77641400	0.32260900	-1.87548700
C	-2.66952200	-1.97713700	1.24694500
H	-0.52437300	-1.81123700	1.12639900
C	-3.89734700	-1.60281400	0.70058100
H	-4.88939100	-0.47732100	-0.85598700
H	-2.63598400	-2.62993500	2.12126500
H	-4.82710200	-1.96310500	1.14456000

Compound	Sturcture	U(0) (Hartree)	H(T) (Hartree)	G(T) (Hartree)
**18**	$^{\bullet+}$PPh$_3$	-1035.7996673	-1035.7836029	-1035.8415030

Cartesian Coordinates (CH_3CN)

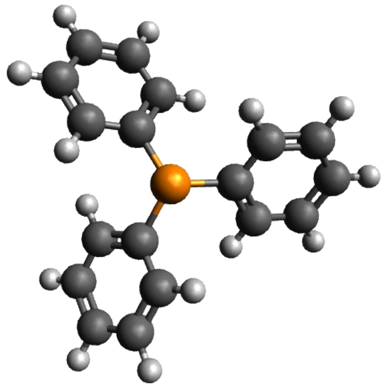

P	0.00032600	0.00006300	-0.65088600
C	-0.32884000	1.69820700	-0.22822100
C	0.53026300	2.68819800	-0.73908600
C	-1.43265800	2.04784700	0.56768300
C	0.29205900	4.02280300	-0.43129300
H	1.37684900	2.41222500	-1.37217500
C	-1.65908100	3.38888200	0.86286000
H	-2.09788100	1.27974500	0.96585900
C	-0.80080500	4.37224900	0.36648100
H	0.95704900	4.79443600	-0.82101800
H	-2.50823700	3.66616800	1.48871300
H	-0.98597700	5.42160200	0.60170700
C	-1.30643500	-1.13342900	-0.22708100
C	-1.06231200	-2.25294600	0.58605600
C	-2.58861600	-0.89463600	-0.75443200
C	-2.11107500	-3.11887200	0.88128100
H	-0.06776500	-2.43673600	0.99634500
C	-3.62590100	-1.76760100	-0.44666600

H	-2.76844800	-0.03244200	-1.40089800
C	-3.38714600	-2.87770800	0.36788700
H	-1.93077500	-3.98430700	1.52015600
H	-4.62296900	-1.58593700	-0.84964200
H	-4.20376100	-3.56230000	0.60290400
C	1.63566200	-0.56453400	-0.22823500
C	2.06588500	-1.79890900	-0.74856000
C	2.48772900	0.21063300	0.57637500
C	3.34094300	-2.26072400	-0.44249300
H	1.40467300	-2.38965800	-1.38693800
C	3.76231000	-0.26475700	0.86995900
H	2.15301200	1.16660200	0.98275700
C	4.18749300	-1.49461100	0.36336300
H	3.67876400	-3.21865200	-0.83963600
H	4.42479200	0.32728300	1.50263000
H	5.18893800	-1.85951600	0.59726500

Compound	Sturcture	U(0) (Hartree)	H(T) (Hartree)	G(T) (Hartree)
**11a**	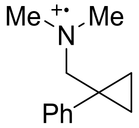	-521.7391468	-521.7251049	-521.7782087

Cartesian Coordinates (CH_3CN)

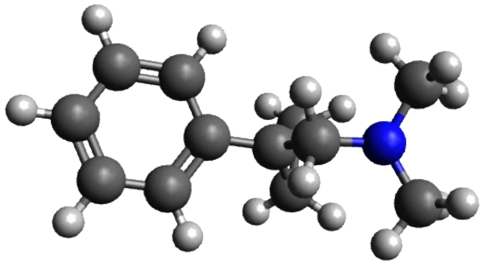

C	0.35769400	0.17051000	0.43893300
C	0.86553500	-0.25269500	1.78585400
C	0.82193900	1.20683800	1.41725600
H	1.84566700	-0.72850100	1.85927900
H	0.12457400	-0.62537400	2.49601600
H	0.04976900	1.82832700	1.87522600
H	1.77107200	1.71691900	1.24204200
C	-1.11295100	0.05794600	0.14668900
C	-1.76244800	-1.17758900	0.25450800
C	-1.83754300	1.17437500	-0.28562400
C	-3.11764700	-1.29275200	-0.05430800
H	-1.19965000	-2.05228900	0.59025800
C	-3.19322700	1.06048000	-0.59530300
H	-1.33387700	2.14031000	-0.37273200
C	-3.83543300	-0.17328800	-0.47957500
H	-3.61620800	-2.25914100	0.04004600
H	-3.75046400	1.93892400	-0.92577000
H	-4.89662600	-0.26238200	-0.71897100
C	3.40280800	1.00702600	-0.71597600
H	2.78470300	1.88234900	-0.93519500
H	3.96595500	1.13870100	0.22253900

H	4.14101000	0.84325600	-1.52069100	
N	2.59049700	-0.17120700	-0.60989000	
C	3.23247700	-1.43249400	-0.36754400	
H	2.51403700	-2.14515800	0.05305600	
H	3.58745700	-1.81763400	-1.34242900	
H	4.10052000	-1.28766000	0.28786800	
C	1.17011600	-0.12595100	-0.83948600	
H	0.96667500	0.65865800	-1.58186300	
H	0.86399500	-1.10529700	-1.23199000	
**Compound**	**Sturcture**	**U(0) (Hartree)**	**H(T) (Hartree)**	**G(T) (Hartree)**
**4ad**	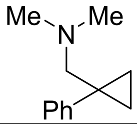	-521.9324394	-521.9196194	-521.9692859
Cartesian Coordinates (CH_3CN)	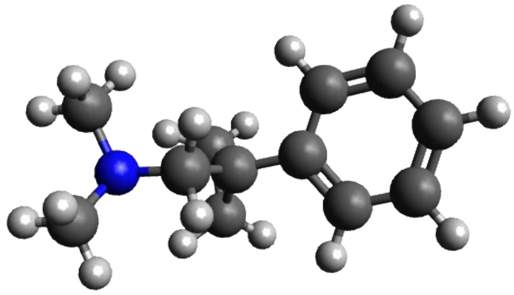			
C	0.32929400	-0.31748700	0.49253400	
C	0.71176900	-1.50063300	1.32874700	
C	0.78137300	-0.11504000	1.91036900	
H	1.66351300	-1.98008200	1.08767800	
H	-0.08244000	-2.16645400	1.67367800	
H	0.02314500	0.16939900	2.64363900	
H	1.77616600	0.30135600	2.07398200	
C	-1.12045800	-0.10424000	0.16221100	
C	-1.89223800	-1.13378900	-0.38974700	
C	-1.70794400	1.15604800	0.33748400	
C	-3.22266100	-0.91330700	-0.75094100	
H	-1.44379200	-2.12032700	-0.53187400	
C	-3.03707100	1.37991600	-0.02112500	
H	-1.11300000	1.96671200	0.76676200	
C	-3.79862100	0.34423200	-0.56679100	
H	-3.81217900	-1.72799900	-1.17604100	
H	-3.48172500	2.36588000	0.12773000	
H	-4.83972000	0.51715700	-0.84565200	
C	3.11843600	1.42669000	0.00958100	
H	3.14523800	2.08545800	-0.88509000	
H	2.45103500	1.89247900	0.74791800	
H	4.13139900	1.39313500	0.43816200	
N	2.66336300	0.08783400	-0.31001800	
C	3.52099200	-0.53608800	-1.29744600	
H	3.20995500	-1.57628400	-1.46985000	
H	3.51106500	-0.00580800	-2.27382800	
H	4.55943400	-0.54685500	-0.93412400	
C	1.25575400	0.05850200	-0.66844500	

H	0.92980300	1.03315200	-1.08479600
H	1.09681200	-0.68458100	-1.46675000

Table S5. Gibbs free energy change of the proposed process

1	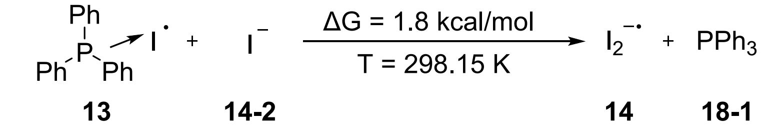
2	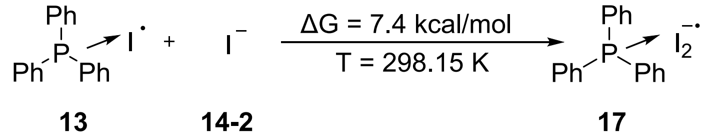
3	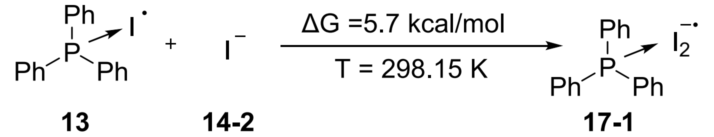
4	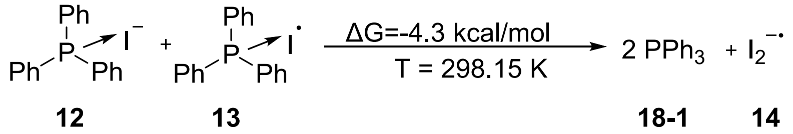
5	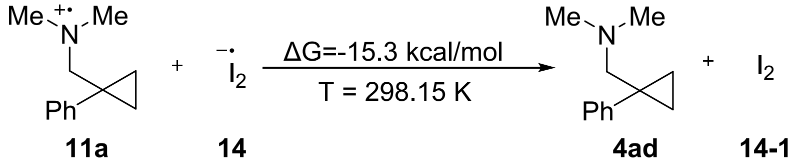
6	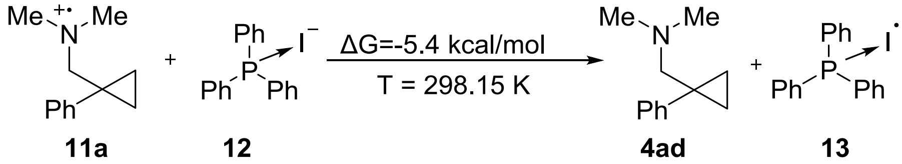
7	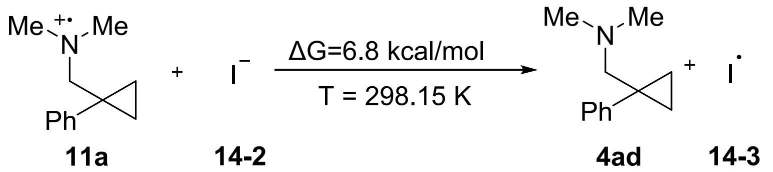
8	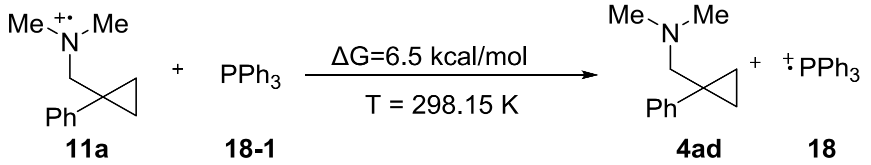
9	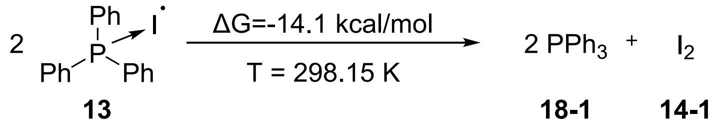

9. X-ray crystallographic data of 4kc

Single crystals of C25H34N2O2 [mo_WJ_250925_a] (CCDC 2491437) were obtained by slow solvent evaporation from dichloromethane solution. A suitable crystal was selected and measured on a Bruker APEX-IICCD diffractometer. The crystal was kept at 180.0 K during data collection. Using Olex2 [1], the structure was solved with the SHELXT [2] structure solution program using Intrinsic Phasing and refined with the SHELXL [3] refinement package using Least Squares minimisation.

[1] Dolomanov, O.V., Bourhis, L.J., Gildea, R.J, Howard, J.A.K. & Puschmann, H. (2009), J. Appl. Cryst. 42, 339-341.
[2] Sheldrick, G.M. (2015). Acta Cryst. A71, 3-8.
[3] Sheldrick, G.M. (2015). Acta Cryst. C71, 3-8.

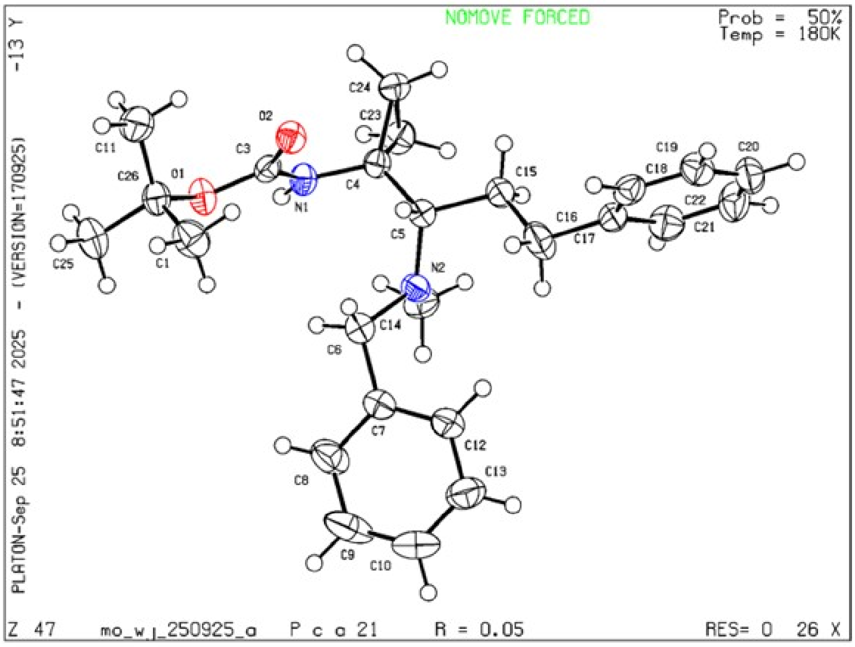

Table S4. Crystal data and structure refinement for mo_WJ_250925_a.

Identification code	mo_WJ_250925_a
Empirical formula	$C_{25}H_{34}N_2O_2$
Formula weight	394.54
Temperature/K	180.0
Crystal system	orthorhombic
Space group	$Pca2_1$
a/Å	25.759(10)
b/Å	9.319(3)
c/Å	9.352(3)
α /°	90
β /°	90
γ /°	90
Volume/Å^3	2245.0(13)
Z	4
ρ_{calc}g/cm^3	1.167
μ /mm^{-1}	0.074
F(000)	856.0
Crystal size/mm^3	0.39 × 0.04 × 0.03
Radiation	MoKα (λ = 0.71073)

2Θ range for data collection/°	4.37 to 49.996
Index ranges	-30 ≤ h ≤ 30, -11 ≤ k ≤ 11, -11 ≤ l ≤ 10
Reflections collected	18530
Independent reflections	3806 [R_{int} = 0.1206, R_{sigma} = 0.0753]
Data/restraints/parameters	3806/1/266
Goodness-of-fit on F^2	1.073
Final R indexes [I>=2σ (I)]	R_1 = 0.0458, wR_2 = 0.0944
Final R indexes [all data]	R_1 = 0.0604, wR_2 = 0.1055
Largest diff. peak/hole / e Å^{-3}	0.16/-0.17
Flack parameter	0.4(10)

10. Characteristic of the Obtained New Compounds

N-benzyl-*N*-methyl-3-phenyl-1-(1-phenylcyclopropyl)propan-1-amine (**4a**)

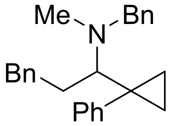

The titled compound was synthesized following the general procedure A. EtOAc/*n*-hexane (1:5); colorless oil (61 mg, 86%); ^{1}H NMR (600 MHz, $CDCl_3$): δ 7.47 (d, *J* = 7.4 Hz, 2H), 7.30 (t, *J* = 7.5 Hz, 2H), 7.29-7.24 (m, 6H), 7.24-7.18 (m, 5H), 3.72-3.63 (m, 2H), 2.87-2.81 (m, 1H), 2.75-2.68 (m, 1H), 2.32 (dd, *J* = 9.3, 4.5 Hz, 1H), 2.15 (s, 3H), 2.14-2.06 (m, 1H), 1.99-1.93 (m, 1H), 0.98-0.94 (m, 1H), 0.88-0.85 (m, 1H), 0.69-0.64 (m, 1H), 0.60-0.56 (m, 1H). ^{13}C NMR (151 MHz, $CDCl_3$): δ 144.74, 142.94, 141.01, 131.50, 128.52, 128.43, 128.31, 128.15, 127.80, 126.56, 126.36, 125.79, 70.45, 58.74, 37.57, 34.44, 32.18, 28.88, 13.12, 10.74. HRMS (ESI): $[M+H]^+$ calcd. for $C_{26}H_{30}N^+$: m/z = 356.2373; found, 356.2381.

N-benzyl-*N*-methyl-2-phenyl-1-(1-phenylcyclopropyl)ethan-1-amine (**4aa**)

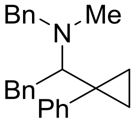

The titled compound was synthesized following the general procedure A. EtOAc/*n*-hexane (1:5); colorless oil (52 mg, 75%); ^{1}H NMR (400 MHz, $CDCl_3$): δ 7.54-7.49 (m, 2H), 7.34-7.25 (m, 4H), 7.25-7.12 (m, 7H), 6.94 (dd, *J* = 7.3, 2.3 Hz, 2H), 3.61-3.50 (m, 2H), 3.08-2.93 (m, 2H), 2.78 (dd, *J* = 8.8, 5.1 Hz, 1H), 2.14 (s, 3H), 0.94-0.84 (m, 2H), 0.77-0.71 (m, 1H), 0.70-0.63 (m, 1H). ^{13}C NMR (101 MHz, $CDCl_3$): δ 144.77, 141.54, 140.71, 131.33, 129.23, 128.31, 128.22, 127.93, 127.85, 126.44, 126.38, 125.78, 70.81, 60.27, 37.89, 36.25, 27.97, 12.99, 10.88. HRMS (ESI): $[M+H]^+$ calcd. for $C_{25}H_{28}N^+$: m/z = 342.2216; found, 342.2225.

N-benzyl-*N*-methyl-1-(1-phenylcyclopropyl)butan-1-amine (**4ba)**

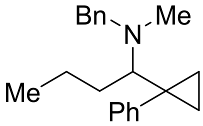

The titled compound was synthesized following the general procedure A. EtOAc/*n*-hexane (1:10); colorless oil (37 mg, 64%); ^{1}H NMR (400 MHz, $CDCl_3$): δ 7.49 (d, *J* = 7.3 Hz, 2H), 7.32-7.28 (m, 4H),

7.27-7.20 (m, 4H), 3.73-3.59 (m, 2H), 2.32 (dd, J = 8.6, 4.6 Hz, 1H), 2.13 (s, 3H), 1.82-1.72 (m, 1H), 1.64-1.46 (m, 3H), 1.00 (d, J = 7.2 Hz, 1H), 0.96-0.92 (m, 3H), 0.90-0.85 (m, 1H), 0.75-0.66 (m, 2H). ^{13}C NMR (101 MHz, CDCl$_3$): δ 145.02, 141.21, 131.41, 128.33, 128.07, 127.70, 126.48, 126.21, 70.62, 59.04, 37.74, 32.10, 29.01, 21.52, 14.45, 12.81, 11.32. HRMS (ESI): [M+H]$^+$ calcd. for $C_{21}H_{28}N^+$: m/z = 294.2216; found, 294.2225.

(*Z*)-*N*-benzyl-*N*-methyl-1-(1-phenylcyclopropyl)hept-4-en-1-amine (**4ca**)

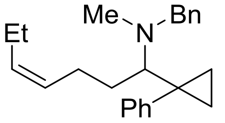

The titled compound was synthesized following the general procedure A. EtOAc/*n*-hexane (1:5); colorless oil (32 mg, 49%); ^{1}H NMR (600 MHz, CDCl$_3$): δ 7.47 (d, J = 7.3 Hz, 2H), 7.27 (t, J = 7.4 Hz, 4H), 7.25-7.19 (m, 4H), 5.45-5.36 (m, 2H), 3.64 (dd, 2H), 2.31 (dd, J = 9.0, 4.7 Hz, 1H), 2.28-2.14 (m, 2H), 2.13 (s, 3H), 2.11-2.06 (m, 2H), 1.87-1.80 (m, 1H), 1.70-1.63 (m, 1H), 0.99 (t, J = 7.6 Hz, 3H), 0.97-0.94 (m, 1H), 0.90-0.86 (m, 1H), 0.71-0.67 (m, 1H), 0.66-0.62 (m, 1H). ^{13}C NMR (151 MHz, CDCl$_3$): δ 144.94, 141.16, 132.05, 131.50, 129.28, 128.35, 128.15, 127.82, 126.57, 126.36, 70.51, 58.87, 37.81, 30.13, 28.97, 25.82, 20.78, 14.60, 13.01, 11.25. HRMS (ESI): [M+H]$^+$ calcd. for $C_{24}H_{32}N^+$: m/z = 334.2529; found, 334.2541.

Ethyl 6-(benzyl(methyl)amino)-6-(1-phenylcyclopropyl)hexanoate (**4da**)

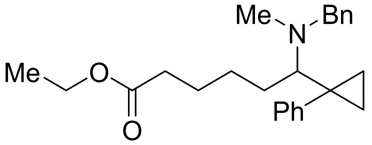

The titled compound was synthesized following the general procedure A. EtOAc/*n*-hexane (1:10); colorless oil (55 mg, 72%); ^{1}H NMR (600 MHz, CDCl$_3$): δ 7.43 (d, J = 7.1 Hz, 2H), 7.34-7.23 (m, 4H), 7.20-7.17 (m, 4H), 4.15-4.10 (m, 2H), 3.64-3.56 (m, 2H), 2.32 (t, J = 7.5 Hz, 2H), 2.24 (dd, J = 8.9, 4.9 Hz, 1H), 2.07 (s, 3H), 1.80-1.73 (m, 1H), 1.70-1.57 (m, 4H), 1.53-1.41 (m, 2H), 1.25 (t, J = 7.1 Hz, 3H), 0.95-0.90 (m, 1H), 0.85-0.80 (m, 1H), 0.68-0.64 (m, 1H), 0.63-0.58 (m, 1H). ^{13}C NMR (151 MHz, CDCl$_3$): δ 173.92, 144.78, 141.02, 131.43, 128.28, 128.09, 127.75, 126.52, 126.30, 70.67, 60.30, 58.44, 37.68, 34.48, 29.58, 28.92, 28.16, 25.29, 14.35, 13.58, 10.86. HRMS (ESI): [M+H]$^+$ calcd. for $C_{25}H_{34}NO_2^+$: m/z = 380.2584; found, 380.2595.

2-(3-(benzyl(methyl)amino)-3-(1-phenylcyclopropyl)propyl)isoindoline-1,3-dione (**4ea**)

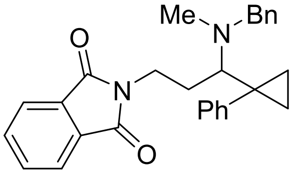

The titled compound was synthesized following the general procedure A. EtOAc/*n*-hexane (1:5); colorless oil (75 mg, 88%); ^{1}H NMR (400 MHz, CDCl$_3$): δ 7.73 (dd, J = 5.5, 3.1 Hz, 2H), 7.60 (dd, J = 5.5, 3.0 Hz, 2H), 7.34 (d, J = 7.5 Hz, 2H), 7.17-7.10 (m, 6H), 7.11-7.04 (m, 2H), 3.81-3.63 (m, 2H), 3.62-3.52 (m, 2H), 2.32 (dd, J = 9.4, 4.7 Hz, 1H), 2.02 (s, 4H), 1.93-1.81 (m, 1H), 0.88-0.76 (m, 2H), 0.58-0.47 (m, 2H). ^{13}C NMR (151 MHz, CDCl$_3$): δ 168.56, 144.39, 140.60, 133.99, 132.35, 131.53, 128.45, 128.21, 127.95, 126.67, 126.55, 123.29, 68.66, 58.87, 37.29, 37.18, 28.93, 28.54, 13.11, 10.52. HRMS (ESI): [M+H]$^+$ calcd. for $C_{28}H_{29}N_2O_2^+$: m/z = 425.2224; found, 425.2236.

N-benzyl-1-cyclohexyl-*N*-methyl-1-(1-phenylcyclopropyl)methanamine (**4fa**)

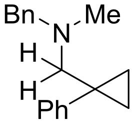

The titled compound was synthesized following the general procedure A paraformaldehyde (2.0 mmol) was used as carbonyl compound. EtOAc/*n*-hexane (1:5); colorless oil (29 mg, 58%); ^{1}H NMR (600 MHz, $CDCl_3$): δ 7.33 (dd, *J* = 8.2, 1.5 Hz, 2H), 7.29 (dd, *J* = 8.5, 6.8 Hz, 2H), 7.24-7.17 (m, 4H), 7.11-7.08 (m, 2H), 3.50 (s, 2H), 2.58 (s, 2H), 2.21 (s, 3H), 0.91-0.87 (m, 2H), 0.74-0.70 (m, 2H). ^{13}C NMR (151 MHz, $CDCl_3$): δ 144.55, 139.52, 128.85, 128.82, 128.11, 128.00, 126.78, 125.93, 66.24, 62.39, 42.81, 23.69, 11.87. HRMS (ESI): $[M+H]^+$ calcd. for $C_{18}H_{22}N^+$: m/z = 252.1747; found, 252.1752.

N-benzyl-*N*, 2-dimethyl-1-(1-phenylcyclopropyl)propan-1-amine (**4ga**)

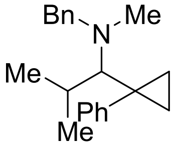

The titled compound was synthesized following the general procedure A. EtOAc/*n*-hexane (1:5); colorless oil (25 mg, 41%); ^{1}H NMR (400 MHz, $CDCl_3$): δ 7.56-7.49 (m, 2H), 7.35-7.27 (m, 4H), 7.25-7.17 (m, 4H), 3.87 (s, 2H), 2.24-2.11 (m, 1H), 2.02 (s, 3H), 1.84 (d, *J* = 10.3 Hz, 1H), 1.32 (d, *J* = 6.6 Hz, 3H), 1.12-0.97 (m, 4H), 0.89-0.84(m, 1H), 0.79-0.74 (m, 1H), 0.72-0.67 (m, 1H). ^{13}C NMR (101 MHz, $CDCl_3$): δ 144.99, 141.40, 131.64, 128.09, 128.07, 127.65, 126.39, 126.20, 78.07, 60.64, 37.24, 30.63, 27.20, 23.01, 22.13, 13.52, 12.40. HRMS (ESI): $[M+H]^+$ calcd. for $C_{21}H_{28}N^+$: m/z = 294.2216; found, 294.2223.

N-benzyl-1-cyclopropyl-*N*-methyl-1-(1-phenylcyclopropyl)methanamine (**4ha**)

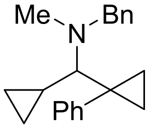

The titled compound was synthesized following the general procedure A. EtOAc/*n*-hexane (1:5); colorless oil (21 mg, 36%); ^{1}H NMR (400 MHz, $CDCl_3$): δ 7.50-7.46 (m, 2H), 7.32-7.18 (m, 6H), 7.16-7.10 (m, 2H), 3.82-3.68 (m, 2H), 2.28 (s, 3H), 1.69 (d, *J* = 10.0 Hz, 1H), 1.02-0.85 (m, 3H), 0.85-0.75 (m, 2H), 0.65-0.54 (m, 2H), 0.38-0.25 (m, 2H). ^{13}C NMR (151 MHz, $CDCl_3$): δ 145.58, 141.20, 131.03, 128.49, 128.10, 127.60, 126.50, 126.08,74.82, 60.01, 39.26, 31.55, 29.59, 11.24, 10.59, 4.83, 3.60. HRMS (ESI): [M+H]+ calcd. for $C_{21}H_{26}N^+$: m/z = 292.2060; found, 292.2069.

N-benzyl-1-cyclobutyl-*N*-methyl-1-(1-phenylcyclopropyl)methanamine (**4ia**)

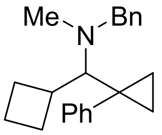

The titled compound was synthesized following the general procedure A. EtOAc/*n*-hexane (1:5); colorless oil (26 mg, 42%); ^{1}H NMR (400 MHz, $CDCl_3$): δ 7.50-7.46 (m, 2H), 7.34-7.27 (m, 6H), 7.25-7.20 (m, 2H), 3.64-3.68 (m, 2H), 2.86-2.72 (m, 1H), 2.38-2.26 (m, 1H), 2.13 (s, 3H), 2.07-1.98 (m, 3H), 1.99-1.73 (m, 3H), 0.90-0.75 (m, 3H), 0.68-0.60 (m, 1H). ^{13}C NMR (151 MHz, $CDCl_3$): δ 145.08, 141.47, 131.68, 128.27, 128.11, 127.70, 126.50, 126.28, 59.55, 38.59, 38.55, 30.10, 29.25, 27.50, 19.14, 10.97, 10.87. HRMS (ESI): $[M+H]^+$ calcd. for $C_{22}H_{28}N^+$: m/z = 306.2216; found, 306.2220.

N-benzyl-*N*-methyl-1-(1-phenylcyclopropyl)-1-(tetrahydrofuran-3-yl)methanamine (**4ja**)

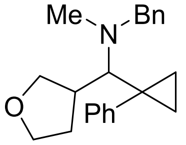

The titled compound was synthesized following the general procedure A. EtOAc/*n*-hexane (1:10); colorless oil (22mg, 34%, 1.1:1 d.r.); ^{1}H NMR (600 MHz, $CDCl_3$): δ 7.36 (dd, *J* = 8.5, 7.1 Hz, 2H), 7.17-7.10 (m, 5H), 7.10-7.04 (m, 3H), 4.20 (t, *J* = 7.6 Hz, 0.5H), 3.87-3.80 (m, 1H), 3.79-3.71 (m, 1H), 3.68-3.58 (m, 2.5H), 3.49-3.53 (m, 1H), 2.59-2.64 (m, 1H), 2.28-2.32 (m, 0.4H), 1.99 (d, *J* = 6.1 Hz, 2H), 1.96 (d, *J* = 9.6 Hz, 2H), 1.93-1.87 (m, 0.6H), 1.82-1.70 (m, 1H), 0.98-0.91 (m, 1H), 0.83-0.73 (m, 1H), 0.61-0.48 (m, 1H), 0.47-0.41 (m, 1H). ^{13}C NMR (151 MHz, $CDCl_3$): δ 144.44, 144.16, 140.81, 140.56, 131.85, 131.73, 128.20, 127.96, 127.89, 126.68, 126.65, 126.62, 126.61, 73.64, 72.95, 72.47, 68.70, 67.82, 59.29, 59.23, 43.01, 42.46, 37.99, 37.87, 32.63, 31.56, 28.47, 27.67, 12.62, 12.01, 11.91, 11.15. HRMS (ESI): $[M+H]^+$ calcd. for $C_{22}H_{28}N^+$: m/z = 322.2165; found, 322.2170.

N-benzyl-1-cyclohexyl-*N*-methyl-1-(1-phenylcyclopropyl)methanamine (**4ka**)

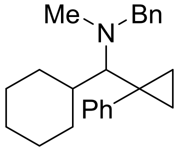

The titled compound was synthesized following the general procedure A. EtOAc/*n*-hexane (1:5); colorless oil (21 mg, 32%); ^{1}H NMR (400 MHz, $CDCl_3$): δ 7.50 (dd, *J* = 7.4, 1.7 Hz, 2H), 7.35-7.27 (m, 4H), 7.27-7.16 (m, 4H), 3.87 (s, 2H), 2.62-2.57 (m, 1H), 1.98 (s, 3H), 1.93 (d, *J* = 10.3 Hz, 1H), 1.88-1.81 (m, 2H), 1.78-1.67 (m, 2H), 1.43-1.07 (m, 5H), 1.06-0.94 (m, 2H), 0.85-0.81 (m, 1H), 0.76-0.71 (m, 1H), 0.69-0.64 (m, 1H). ^{13}C NMR (101 MHz, $CDCl_3$): δ 145.13, 141.50, 131.66, 128.19, 128.13, 127.75, 126.45, 126.19, 76.57,61.56, 40.26, 37.10, 33.17, 31.88, 26.88, 26.78, 26.71, 26.62, 13.66, 11.36. HRMS (ESI): $[M+H]^+$ calcd. for $C_{24}H_{32}N^+$: m/z = 334.2529; found, 334.2538.

tert-Butyl 3-((benzyl(methyl)amino)(1-phenylcyclopropyl)methyl)azetidine-1-carboxylate (**4la**)

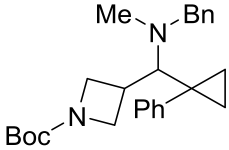

The titled compound was synthesized following the general procedure A. EtOAc/*n*-hexane (1:5); colorless oil (40 mg, 48%); ^{1}H NMR (400 MHz, $CDCl_3$): δ 7.40 (d, 2H), 7.34-7.27 (m, 4H), 7.27-7.20 (m, 4H), 4.16 (t, *J* = 8.4 Hz, 1H), 3.99-3.89 (m, 3H), 3.65-3.69 (m, 2H), 3.03-2.89 (m, 1H), 2.68 (d, *J* = 11.4 Hz, 1H), 2.14 (s, 3H), 1.49 (s, 9H), 0.95-0.90 (m, 1H), 0.89-0.84 (m, 2H), 0.77-0.71 (m, 1H). ^{13}C NMR (101 MHz, $CDCl_3$): δ 155.88, 143.37, 140.00, 131.41, 128.19, 128.07, 127.94, 126.72, 126.65, 79.33, 73.16, 59.87, 37.96, 31.37, 28.48, 26.74, 10.87, 10.59. HRMS (ESI): $[M+H]^+$ calcd. for $C_{26}H_{35}N_2O_2^+$: m/z = 407.2693; found, 407.2702.

tert-Butyl 4-((benzyl(methyl)amino)(1-phenylcyclopropyl)methyl)piperidine-1-carboxylate (**4ma**)

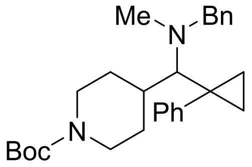

The titled compound was synthesized following the general procedure A. EtOAc/*n*-hexane (1:5); colorless oil (16 mg, 19%); ^{1}H NMR (400 MHz, $CDCl_3$): δ 7.44-7.40 (m, 2H), 7.25 (d, *J* = 2.0 Hz, 2H), 7.24 (s, 2H), 7.22 (s, 2H), 7.20-7.14 (m, 2H), 4.26-3.99 (m, 2H), 3.84 (d, *J* = 4.5 Hz, 2H), 2.82-2.70 (m, 1H), 2.66-2.55 (m, 1H), 2.47 (d, *J* = 13.2 Hz, 1H), 1.94 (s, 3H), 1.91 (s, 1H), 1.88-1.84 (m, 1H), 1.43 (s, 10H), 1.32-1.24 (m, 1H), 1.16 (dd, *J* = 10.2, 3.3 Hz, 1H), 1.06-0.99 (m, 1H), 0.85-0.77 (m, 1H), 0.69-0.58 (m, 2H). ^{13}C NMR (151 MHz, $CDCl_3$): δ 154.93, 144.61, 140.89, 131.55, 128.15, 128.06, 127.85, 126.55, 126.39, 79.23, 75.54, 60.49, 38.61, 36.94, 32.23, 30.92, 28.52, 26.27, 13.23, 11.29. HRMS (ESI): $[M+H]^+$ calcd. for $C_{27}H_{29}N_2^+$: m/z = 435.3006; found, 435.3018.

N-benzyl-*N*-methyl-1-(4-(4-methylpent-3-en-1-yl)cyclohex-3-en-1-yl)-1-(1-phenylcyclopropyl) methanamine (**9a**)

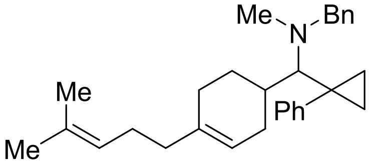

The titled compound was synthesized following the general procedure A. EtOAc/*n*-hexane (1:10); colorless oil (42 mg, 51%, 1.5:1 d.r.); ^{1}H NMR (400 MHz, $CDCl_3$): δ 7.41 (dd, *J* = 8.0, 6.2 Hz, 2H), 7.22 (t, *J* = 5.6 Hz, 4H), 7.19-7.07 (m, 4H), 5.42-5.32 (m, 1H), 5.13-5.00 (m, 1H), 3.83-3.80 (m, 1H), 3.76 (s, 1H), 2.67-2.50 (m, 1H), 2.21-2.07 (m, 2H), 2.01 (m, 4H), 1.94 (d, *J* = 6.1 Hz, 3H), 1.92-1.84 (m, 3H), 1.83-1.71 (m, 1H), 1.66-1.57 (m, 3H), 1.56-1.45 (m, 3H), 1.37-1.27 (m, 1H), 0.99 (tt, *J* = 9.5, 5.1 Hz, 1H), 0.85-0.74 (m, 1H), 0.71-0.53 (m, 2H). ^{13}C NMR (151 MHz, $CDCl_3$): δ 144.96, 144.94, 141.33, 141.24, 137.86, 137.17, 131.70, 131.67, 131.54, 131.47, 131.35, 131.34, 128.11, 128.10, 128.07, 128.05, 127.73, 127.71, 126.43, 126.38, 126.22, 126.18, 124.54, 124.49, 120.93, 120.84, 120.17, 119.96, 76.54, 76.46, 74.63, 74.38, 60.77, 60.65, 60.35, 60.28, 38.17, 37.56, 37.50, 37.40, 36.97, 36.72, 36.36, 36.28, 35.72, 35.11, 34.54, 31.77, 31.33, 29.09, 28.78, 28.07, 27.17, 26.88, 26.60, 26.53, 26.43, 25.77, 25.75, 25.74, 25.01, 17.79, 17.75, 17.72, 17.69, 13.68, 13.10, 12.98, 11.60, 11.55, 11.19, 11.16. HRMS (ESI): $[M+H]^+$ calcd. for $C_{30}H40N^+$: m/z = 414.3155; found, 414.3163.

N-benzyl-2-(6-methoxynaphthalen-2-yl)-*N*-methyl-1-(1-phenylcyclopropyl)propan-1-amine (**9b**)

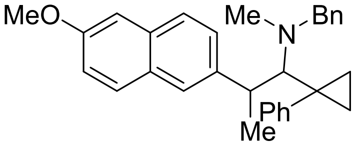

The titled compound was synthesized following the general procedure A. EtOAc/*n*-hexane (1:10); colorless oil (59 mg, 68%, 1:1 d.r.); ^{1}H NMR (400 MHz, $CDCl_3$): δ 7.75 (d, *J* = 8.7 Hz, 1H), 7.73-7.68 (m, 3H), 7.66-7.62 (m, 2H), 7.58 (d, *J* = 8.9 Hz, 1H), 7.48 (d, *J* = 1.7 Hz, 1H), 7.47-7.43 (m, 2H), 7.40 (d, *J* = 2.8 Hz, 1H), 7.39-7.31 (m, 7H), 7.28 (d, *J* = 1.6 Hz, 1H), 7.24-7.22 (m, 1H), 7.21 (d, *J* = 1.9 Hz, 1H), 7.19 (dd, *J* = 4.4, 1.9 Hz, 2H), 7.15 (dd, *J* = 5.2, 2.3 Hz, 3H), 7.10 (dd, *J* = 8.9, 2.5 Hz, 1H), 7.03-6.98 (m, 1H), 6.93 (dd, *J* = 8.1, 6.5 Hz, 2H), 6.69-6.64 (m, 2H), 4.08-3.98 (m, 2H), 3.94 (d, *J* = 4.0 Hz, 6H), 3.77-3.69 (m, 1H), 3.52 (d, *J* = 14.2 Hz, 1H), 3.46-3.30 (m, 2H), 2.89 (d, *J* = 10.5 Hz, 1H), 2.63 (d, *J* = 11.0 Hz, 1H), 2.24 (s, 3H), 1.89 (s, 3H), 1.66 (d, *J* = 6.8 Hz, 3H), 1.34 (d, *J* = 6.9 Hz, 3H), 1.16-1.10 (m, 1H), 1.00 (m, 2H), 0.87-0.81 (m, 1H), 0.81-0.74 (m, 2H), 0.28-0.15 (m, 2H). ^{13}C NMR (151 MHz, $CDCl_3$): δ 157.38, 157.14, 145.57, 144.63, 142.76, 142.49, 141.20, 140.97, 133.26, 133.22, 131.90, 131.87, 129.25, 129.18, 129.02, 128.27, 127.94, 127.92, 127.71, 127.59, 127.35, 126.96, 126.76, 126.65, 126.59, 126.51, 126.17, 124.58, 124.11, 118.74, 118.40, 105.81, 105.74, 76.38, 75.27, 60.92, 60.20, 55.40, 43.14, 42.66, 37.78, 37.35, 31.55, 30.32, 28.53, 27.66, 23.84, 22.98, 13.76, 12.61, 12.32, 11.74. HRMS (ESI): $[M+H]^+$ calcd. for $C_{31}H_{34}NO^+$: m/z = 436.2635; found, 436.2641.

N-benzyl-*N*-ethyl-3-phenyl-1-(1-phenylcyclopropyl)propan-1-amine (**4ab**)

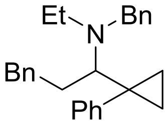

The titled compound was synthesized following the general procedure A. EtOAc/*n*-hexane (1:15); colorless oil (51 mg, 69%); ^{1}H NMR (600 MHz, $CDCl_3$): δ 7.43-7.39 (m, 2H), 7.27 (d, *J* = 7.9 Hz, 3H), 7.25 (d, *J* = 1.6 Hz, 2H), 7.24-7.19 (m, 3H), 7.19-7.13 (m, 5H), 3.65 (dd, *J* = 114.9, 14.7 Hz, 2H), 2.83-2.74 (m, 1H), 2.67-2.59 (m, 1H), 2.40-2.34 (m, 2H), 2.29 (dd, *J* = 8.5, 5.2 Hz, 1H), 2.03-1.94 (m, 1H), 1.92-1.85 (m, 1H), 0.90 (t, *J* = 7.0 Hz, 3H), 0.88-0.83 (m, 1H), 0.73-0.67 (m, 1H), 0.62-0.57 (m, 1H), 0.56-0.51 (m, 1H). ^{13}C NMR (151 MHz, $CDCl_3$): δ 144.86, 143.09, 141.82, 131.59, 128.51, 128.46, 128.38, 128.09, 127.78, 126.47, 126.43, 125.80, 66.23, 54.96, 44.10, 34.63, 31.62, 29.05, 14.87, 12.67, 11.52. HRMS(ESI): $[M+H]^+$ calcd. for $C_{27}H_{32}N^+$: m/z = 370.2529; found, 370.2538.

N-benzyl-*N*-(3-phenyl-1-(1-phenylcyclopropyl)propyl)butan-1-amine (**4bb**)

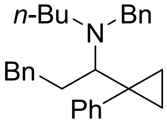

The titled compound was synthesized following the general procedure A. EtOAc/*n*-hexane (1:5); colorless oil (34 mg, 43%); ^{1}H NMR (600 MHz, $CDCl_3$): δ 7.45-7.41 (m, 2H), 7.30-7.26 (m, 5H), 7.25-7.21 (m, 3H), 7.21-7.15 (m, 5H), 3.62 (dd, *J* = 109.3, 14.6 Hz, 2H), 2.85-2.79 (m, 1H), 2.69-2.61 (m, 1H), 2.37-2.23 (m, 3H), 2.04-1.95 (m, 1H), 1.93-1.85 (m, 1H), 1.37-1.27 (m, 2H), 1.18-1.05 (m, 2H), 0.92-0.86 (m, 1H), 0.77 (t, *J* = 7.4 Hz, 3H), 0.74-0.69 (m, 1H), 0.65-0.59 (m, 1H), 0.58-0.53 (m, 1H). ^{13}C NMR (151 MHz, $CDCl_3$): δ 144.90, 143.08, 141.78, 131.56, 128.51, 128.45, 128.06, 127.78, 127.65, 126.45, 126.43, 125.80, 66.32, 55.40, 50.35, 35.11, 31.93, 31.74, 29.00, 20.55, 14.22, 12.60, 11.61. HRMS (ESI): $[M+H]^+$ calcd. for $C_{29}H_{36}N^+$: m/z = 398.2842; found, 398.2850.

N-benzyl-*N*-(3-phenyl-1-(1-phenylcyclopropyl)propyl)prop-2-en-1-amine (**4cb**)

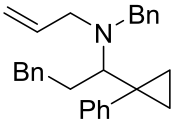

The titled compound was synthesized following the general procedure A. EtOAc/*n*-hexane (1:5); colorless oil (59 mg, 77%); ^{1}H NMR (600 MHz, $CDCl_3$): δ 7.44 (d, *J* = 7.5 Hz, 2H), 7.28 (dd, *J* = 14.3, 7.0 Hz, 4H), 7.24 (d, *J* = 7.8 Hz, 3H), 7.19 (dd, *J* = 14.0, 7.1 Hz, 6H), 5.79-5.70 (m, 1H), 5.02 (dd, *J* = 21.4, 13.7 Hz, 2H), 3.65 (dd, *J* = 110.1, 14.5 Hz, 2H), 3.04-2.93 (m, 2H), 2.86-2.79 (m, 1H), 2.70-2.62 (m, 1H), 2.34 (dd, *J* = 8.5, 5.3 Hz, 1H), 2.06-1.97 (m, 1H), 1.94-1.86 (m, 1H), 0.90-0.86 (m, 1H), 0.72-0.67 (m, 1H), 0.65-0.60 (m, 1H), 0.57-0.52 (m, 1H). ^{13}C NMR (151 MHz, $CDCl_3$): δ 144.60, 142.95, 141.16, 138.26, 131.59, 128.47, 128.45, 128.10, 127.81, 126.54, 126.51, 125.81, 116.05, 65.40, 54.42, 53.16, 34.51, 31.81, 28.85, 12.52, 10.97. HRMS (ESI): $[M+H]^+$ calcd. for $C_{28}H_{32}N^+$: m/z = 382.2529; found, 382.2536.

N, *N*-dibenzyl-3-phenyl-1-(1-phenylcyclopropyl)propan-1-amine (**4db**)

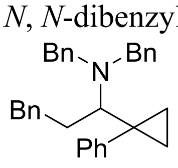

The titled compound was synthesized following the general procedure A. *n*-hexane; colorless oil (41 mg, 48%); ^{1}H NMR (600 MHz, $CDCl_3$): δ 7.51 (d, 2H), 7.34-7.27 (m, 12H), 7.26-7.18 (m, 6H), 3.62 (dd, *J* = 104.2, 14.2 Hz, 4H), 2.95-2.89 (m, 1H), 2.76-2.69 (m, 1H), 2.38 (t, *J* = 7.9, 5.6, 2.1 Hz, 1H), 2.21-2.13 (m, 1H), 1.99-1.91 (m, 1H), 0.97-0.92 (m, 1H), 0.70-0.63 (m, 2H), 0.56-0.51 (m, 1H). ^{13}C NMR (151 MHz, $CDCl_3$): δ 144.86, 142.94, 140.85, 131.66, 128.69, 128.57, 128.23, 127.95, 126.72, 126.63,

125.92, 64.70, 54.36, 34.76, 31.30, 28.49, 12.38, 11.91. HRMS (ESI): $[M+H]^+$ calcd. for $C_{32}H_{34}N^+$: m/z = 432.2686; found, 432.2692.

N-benzyl-*N*-isopropyl-3-phenyl-1-(1-phenylcyclopropyl)propan-1-amine (**4eb**)

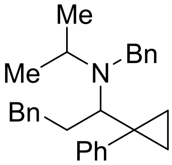

The titled compound was synthesized following the general procedure A. EtOAc/*n*-hexane (1:5); colorless oil (62 mg, 81%); ^{1}H NMR (600 MHz, $CDCl_3$): δ 7.42 (d, 2H), 7.27 (t, J = 7.6 Hz, 3H), 7.24 (d, J = 4.5 Hz, 3H), 7.20-7.13 (m, 7H), 3.49 (s, 2H), 2.89-2.85 (m, 1H), 2.78-2.71 (m, 1H), 2.70-2.63 (m, 1H), 2.47 (dd, J = 7.6, 4.7 Hz, 1H), 1.93-1.81 (m, 2H), 1.00 (d, J = 6.7 Hz, 3H), 0.90 (d, J = 6.7 Hz, 3H), 0.89-0.85 (m, 1H), 0.80-0.74 (m, 2H), 0.72-0.68 (m, 1H). ^{13}C NMR (151 MHz, $CDCl_3$): δ 145.13, 142.92, 142.42, 131.23, 128.49, 128.43, 128.06, 128.01, 127.77, 126.37, 126.24, 125.85, 63.13, 50.39, 48.81, 35.32, 33.29, 30.51, 22.57, 17.93, 13.34, 10.99. HRMS (ESI): $[M+H]^+$ calcd. for $C_{28}H_{34}N^+$: m/z = 384.2686; found, 384.2694.

N-benzhydryl-*N*-methyl-3-phenyl-1-(1-phenylcyclopropyl)propan-1-amine (**4fb**)

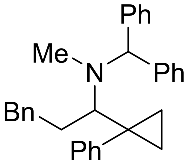

The titled compound was synthesized following the general procedure A. *n*-hexane; colorless oil (63 mg, 73%); ^{1}H NMR (600 MHz, $CDCl_3$): δ 7.46 (dd, J = 8.0, 1.3 Hz, 2H), 7.38 (dd, J = 8.1, 1.3 Hz, 2H), 7.34 (m, 4H), 7.29-7.27 (m, 2H), 7.25-7.23 (m, 1H), 7.22-7.18 (m, 5H), 7.18-7.13 (m, 4H), 4.87 (s, 1H), 2.78 (m, 1H), 2.63 (t, J = 8.6 Hz, 2H), 2.02 (s, 3H), 2.01-1.95 (m, 1H), 1.65-1.57 (m, 1H), 0.83-0.80 (m, 1H), 0.79-0.76 (m, 1H), 0.74-0.70 (m, 2H). ^{13}C NMR (151 MHz, $CDCl_3$): δ 145.82, 144.16, 143.72, 142.93, 131.60, 128.50, 128.44, 128.37, 128.34, 128.00, 127.66, 126.77, 126.66, 126.29, 125.87, 74.39, 63.81, 35.21, 34.22, 29.35, 28.65, 11.04, 10.65. HRMS (ESI): $[M+H]^+$ calcd. for $C_{32}H_{34}N^+$: m/z = 432.2686; found, 432.2698 .

N-phenethyl-1-phenyl-*N*-(1-(p-tolyl)propan-2-yl)cyclopropan-1-amine (**4gb**)

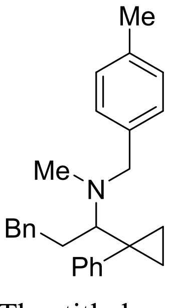

The titled compound was synthesized following the general procedure A. EtOAc/*n*-hexane (1:10); colorless oil (61mg, 83%); ^{1}H NMR (600 MHz, $CDCl_3$): δ 7.49-7.45 (m, 2H), 7.31 (t, J = 7.6 Hz, 2H), 7.28-7.24 (m, 2H), 7.24-7.18 (m, 4H), 7.15 (d, J = 7.8 Hz, 2H), 7.10 (d, J = 7.8 Hz, 2H), 3.63 (dd, 2H), 2.84 (m, 1H), 2.71 (m, 1H), 2.34 (s, 3H), 2.33-2.30 (m, 1H), 2.14 (s, 3H), 2.12-2.06 (m, 1H), 1.99-1.91 (m, 1H), 1.01-0.94 (m, 1H), 0.89-0.85 (m, 1H), 0.70-0.65 (m, 1H), 0.60-0.56 (m, 1H). ^{13}C NMR (151 MHz, $CDCl_3$): δ 144.80, 143.00, 137.91, 136.05, 131.50, 128.85, 128.53, 128.43, 128.29, 127.79, 126.33, 125.78, 70.83, 58.94, 37.46, 34.46, 32.17, 28.90, 21.16, 13.10, 11.24. HRMS (ESI): $[M+H]^+$ calcd. for $C_{27}H_{32}N^+$: m/z = 370.2529; found, 370.2535.

N-(4-methoxybenzyl)-*N*-methyl-3-phenyl-1-(1-phenylcyclopropyl)propan-1-amine (**4hb**)

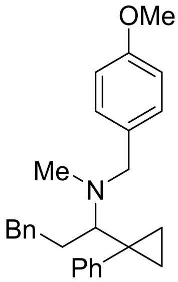

The titled compound was synthesized following the general procedure A. EtOAc/*n*-hexane (1:5); colorless oil (76 mg, 99%); ^{1}H NMR (600 MHz, $CDCl_3$): δ 7.49 (d, *J* = 6.6 Hz, 2H), 7.33 (t, *J* = 7.6 Hz, 2H), 7.30-7.21 (m, 6H), 7.18 (d, *J* = 8.6 Hz, 2H), 6.85 (d, *J* = 8.5 Hz, 2H), 3.81 (s, 3H), 3.65 (dd, 2H), 2.88-2.82 (m, 1H), 2.77-2.69 (m, 1H), 2.33 (dd, *J* = 9.2, 4.5 Hz, 1H), 2.15 (s, 3H), 2.13-2.08 (m, 1H), 2.01-1.93 (m, 1H), 0.99-0.95 (m, 1H), 0.89-8.85 (m, 1H), 0.71-0.67 (m, 1H), 0.63-0.58 (m, 1H). ^{13}C NMR (151 MHz, $CDCl_3$): δ 158.93, 144.89, 143.04, 133.54, 131.56, 129.47, 128.59, 128.51, 127.85, 126.40, 125.86, 113.58, 70.83, 58.30, 55.36, 37.41, 35.11, 32.14, 28.96, 13.13, 10.92. HRMS (ESI): $[M+H]^+$ calcd. for $C_{27}H_{32}NO^+$: m/z = 386.2478; found, 386.2486.

N-(4-fluorobenzyl)-*N*-methyl-3-phenyl-1-(2-phenylcyclopropyl)propan-1-amine (**4ib**)

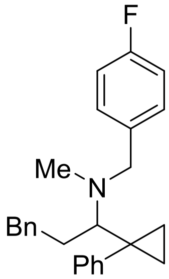

The titled compound was synthesized following the general procedure A. EtOAc/*n*-hexane (1:10); colorless oil (72 mg, 97%); ^{1}H NMR (600 MHz, $CDCl_3$): δ 7.49-7.45 (m, 2H), 7.35-7.30 (m, 1H), 7.28 (t, *J* = 7.4 Hz, 2H), 7.25-7.22 (m, 4H), 7.21-7.17 (m, 2H), 7.00-6.94 (m, 2H), 3.68-3.58 (m, 2H), 2.88-2.80 (m, 1H), 2.76-2.68 (m, 1H), 2.34 (dd, *J* = 9.2, 4.6 Hz, 1H), 2.15 (s, 3H), 2.14-2.05 (m, 1H), 2.01-1.92 (m, 1H), 1.00-0.94 (m, 1H), 0.89-0.83 (m, 1H), 0.73-0.67 (m, 1H), 0.64-0.59 (m, 1H). ^{13}C NMR (151 MHz, $CDCl_3$): δ 162.60, 160.98, 144.79, 142.88, 136.62, 136.60, 131.51, 129.74, 129.68, 128.57, 128.54, 127.90, 126.47, 125.92, 115.00, 114.86, 70.31, 58.06, 37.49, 34.53, 31.99, 28.91, 13.07, 11.22. ^{19}F NMR (376 MHz, $CDCl_3$): δ −117.8 (s). HRMS (ESI): $[M+H]^+$ calcd. for $C_{26}H_{29}FN^+$: m/z = 374.2276; found, 374.2288.

N-(4-chlorobenzyl)-*N*-methyl-3-phenyl-1-(1-phenylcyclopropyl)propan-1-amine (**4jb**)

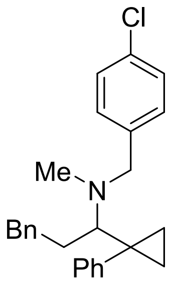

The titled compound was synthesized following the general procedure A. EtOAc/*n*-hexane (1:10); colorless oil (49mg, 64%); ^{1}H NMR (600 MHz, $CDCl_3$): δ 7.43-7.40 (m, 2H), 7.27 (t, *J* = 7.5 Hz, 2H),

7.24-7.15 (m, 8H), 7.11 (d, J = 8.1 Hz, 2H), 3.57 (dd, J = 14.3 Hz, 2H), 2.81-2.73 (m, 1H), 2.70-2.62 (m, 1H), 2.28 (dd, 1H), 2.10 (s, 3H), 2.08-1.99 (m, 1H), 1.95-1.86 (m, 1H), 0.95-0.88 (m, 1H), 0.84-0.78 (m, 1H), 0.68-0.62 (m, 1H), 0.59-0.52 (m, 1H). ^{13}C NMR (151 MHz, $CDCl_3$): δ 144.67, 142.75, 139.51, 132.05, 131.41, 129.55, 128.48, 128.25, 127.84, 126.42, 125.86, 71.13, 57.99, 37.53, 33.34, 31.89, 28.02, 12.98, 9.79. HRMS (ESI): $[M+H]^+$ calcd. for $C_{26}H_{29}ClN^+$: m/z = 390.1983; found, 390.1992.

N-(4-bromobenzyl)-*N*-methyl-3-phenyl-1-(1-phenylcyclopropyl)propan-1-amine (**4kb**)

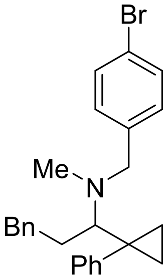

The titled compound was synthesized following the general procedure A. EtOAc/*n*-hexane (1:10); colorless oil (71 mg, 83%); ^{1}H NMR (600 MHz, $CDCl_3$): δ 7.46-7.43 (m, 2H), 7.40-7.37 (m, 2H), 7.33-7.29 (m, 2H), 7.28-7.24 (m, 2H), 7.23-7.19 (m, 4H), 7.11-7.08 (m, 2H), 3.59 (dd, J = 14.3 Hz, 2H), 2.85-2.77 (m, 1H), 2.74-2.66 (m, 1H), 2.32 (dd, J = 9.2, 4.6 Hz, 1H), 2.14 (s, 3H), 2.12-2.02 (m, 1H), 1.98-1.89 (m, 1H), 0.98-0.92 (m, 1H), 0.87-0.81 (m, 1H), 0.72-0.66 (m, 1H), 0.62-0.56 (m, 1H). ^{13}C NMR (151 MHz, $CDCl_3$): δ 144.72, 142.79, 140.10, 131.46, 131.25, 130.01, 128.53, 127.91, 126.48, 125.92, 120.20, 70.36, 58.08, 37.61, 34.50, 31.94, 28.87, 13.04, 11.13. HRMS (ESI): $[M+H]^+$ calcd. for $C_{26}H_{29}BrN^+$: m/z = 434.1478; found, 434.1481.

N-methyl-3-phenyl-1-(1-phenylcyclopropyl)-*N*-(4-(trifluoromethyl)benzyl)propan-1-amine (**4lb**)

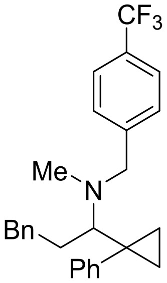

The titled compound was synthesized following the general procedure A. EtOAc/*n*-hexane (1:10); colorless oil (51 mg, 60%); ^{1}H NMR (600 MHz, $CDCl_3$): δ 7.50 (d, J = 8.0 Hz, 2H), 7.44 (d, J = 7.5 Hz, 2H), 7.30 (t, J = 7.8 Hz, 4H), 7.26 (t, J = 7.8 Hz, 2H), 7.21 (dd, J = 7.7, 5.3 Hz, 4H), 3.67 (m, 2H), 2.84-2.74 (m, 1H), 2.74-2.66 (m, 1H), 2.34 (dd, J = 9.2, 4.6 Hz, 1H), 2.16 (s, 3H), 2.10-2.04 (m, 1H), 1.97-1.90 (m, 1H), 0.97-0.93 (m, 1H), 0.87-0.83 (m, 1H), 0.71-0.67 (m, 1H), 0.58-0.62 (m, 1H). ^{13}C NMR (151 MHz, $CDCl_3$): δ 145.33, 144.65, 142.70, 131.43, 128.96, 128.53, 128.38, 127.93, 126.53, 125.95, 125.10, 123.58, 100.74, 70.52, 58.75, 38.42, 34.48, 31.45, 28.38, 13.49, 11.06. ^{19}F NMR (376 MHz, $CDCl_3$): δ −62.7 (s). HRMS (ESI): $[M+H]^+$ calcd. for $C_{27}H_{29}F_3N^+$: m/z =424.2247; found, 424.2253.

4-(2-(phenethyl(1-phenylcyclopropyl)amino)propyl)benzonitrile (**4mb**)

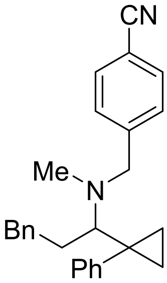

The titled compound was synthesized following the general procedure A. EtOAc/*n*-hexane (1:15); colorless oil (44 mg,58%); ^{1}H NMR (600 MHz, $CDCl_3$): δ 7.32 (d, 2H), 7.23-7.20 (m, 2H), 7.11-7.03 (m, 6H), 7.02-6.97 (m, 4H), 3.44 (m, 2H), 2.62-2.55 (m, 1H), 2.52-2.46 (m, 1H), 2.16-2.12 (m, 1H), 1.96 (s, 3H), 1.89-1.79 (m, 1H), 1.77-1.68 (m, 1H), 0.77-0.71 (m, 1H), 0.65-0.59 (m, 1H), 0.53-0.47 (m, 1H), 0.43-0.37 (m, 1H). ^{13}C NMR (151 MHz, $CDCl_3$): δ 146.92, 144.52, 142.50, 132.04, 131.33, 128.68, 128.52, 128.44, 127.92, 126.54, 125.96, 119.26, 110.26, 70.45, 58.15, 37.87, 34.44, 31.69, 28.75, 12.89, 10.54. HRMS (ESI): $[M+H]^+$ calcd. for $C_{27}H_{29}N_2^+$: m/z = 381.2325; found, 381.2333.

N-phenethyl-1-phenyl-*N*-(1-(o-tolyl)propan-2-yl)cyclopropan-1-amine (**4nb**)

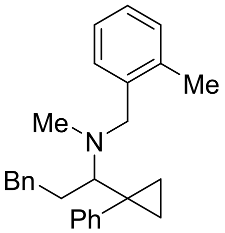

The titled compound was synthesized following the general procedure A. EtOAc/*n*-hexane (1:10); colorless oil (61 mg, 83%); ^{1}H NMR (600 MHz, $CDCl_3$): δ 7.48 (d, *J* = 7.2 Hz, 2H), 7.35-7.26 (m, 5H), 7.25-7.20 (m, 4H), 7.18-7.11 (m, 3H), 3.64 (m, 2H), 2.86-2.79 (m, 1H), 2.76-2.70 (m, 1H), 2.33 (dd, *J* = 9.0, 4.8 Hz, 1H), 2.22 (s, 3H), 2.15 (s, 3H), 2.14-2.07 (m, 1H), 2.01-1.94 (m, 1H), 0.99-0.91 (m, 1H), 0.82-0.76 (m, 1H), 0.72-0.66 (m, 1H), 0.63-0.57 (m, 1H). ^{13}C NMR (151 MHz, $CDCl_3$): δ 144.83, 143.03, 138.54, 137.07, 131.64, 130.14, 128.76, 128.58, 128.51, 127.86, 126.55, 126.45, 125.87, 125.60, 70.28, 56.96, 37.41, 34.70, 31.52, 29.02, 19.60, 13.17, 10.93. HRMS (ESI): $[M+H]^+$ calcd. for $C_{27}H_{32}N^+$: m/z = 370.2529; found, 370.2541.

N-(2-chlorobenzyl)-*N*-methyl-3-phenyl-1-(1-phenylcyclopropyl)propan-1-amine (**4ob**)

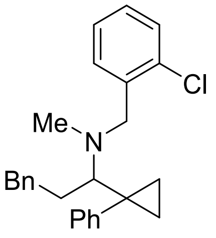

The titled compound was synthesized following the general procedure A. EtOAc/*n*-hexane (1:10); colorless oil (63 mg, 82%); ^{1}H NMR (600 MHz, $CDCl_3$): δ 7.07 (d, *J* = 7.5 Hz, 2H), 7.02 (d, *J* = 7.6 Hz, 1H), 6.92-6.88 (m, 3H), 6.87-6.85 (m, 2H), 6.83-6.78 (m, 5H), 6.74 (t, *J* = 7.6, 1.7 Hz, 1H), 3.39 -3.29 (m, 2H), 2.45-2.37 (m, 1H), 2.33-2.25 (m, 1H), 1.95 (dd, *J* = 9.4, 4.5 Hz, 1H), 1.81 (s, 3H), 1.72-1.63 (m, 1H), 1.61-1.52 (m, 1H), 0.60-0.54 (m, 1H), 0.48-0.42 (m, 1H), 0.31-0.25 (m, 1H), 0.22-0.16 (m, 1H). ^{13}C NMR (151 MHz, $CDCl_3$): δ 144.58, 142.91, 138.20, 133.85, 131.55, 129.69, 129.26, 128.54, 128.46, 127.90, 127.56, 126.58, 126.48, 125.83, 70.79, 55.46, 37.59, 34.49, 32.32, 28.99, 13.18, 11.14. HRMS (ESI): $[M+H]^+$ calcd. for $C_{26}H_{29}ClN^+$: m/z = 390.1983; found, 390.1898.

N-methyl-*N*-(3-methylbenzyl)-3-phenyl-1-(1-phenylcyclopropyl)propan-1-amine (**4pb**)

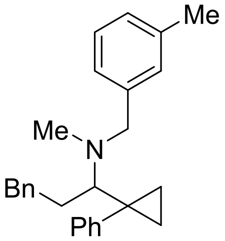

The titled compound was synthesized following the general procedure A. EtOAc/*n*-hexane (1:10); colorless oil (65 mg, 88%); ^{1}H NMR (400 MHz, $CDCl_3$): δ 7.52-7.47 (m, 2H), 7.36-7.26 (m, 5H), 7.25-7.17 (m, 4H), 7.11-7.02 (m, 3H), 3.66 (m, 2H), 2.92-2.67 (m, 2H), 2.40-2.34 (m, 1H), 2.35 (s, 3H), 2.18 (s, 3H), 2.16-2.05 (m, 1H), 2.02-1.92 (m, 1H), 1.01-0.95 (m, 1H), 0.92-0.86 (m, 1H), 0.73-0.66 (m, 1H), 0.64-0.58 (m, 1H). ^{13}C NMR (101 MHz, $CDCl_3$): δ 144.82, 142.97, 140.94, 137.62, 131.47, 129.08, 128.51, 128.42, 128.03, 127.78, 127.29, 126.33, 125.78, 125.37, 77.10, 70.95, 58.12, 37.60, 35.13, 31.60, 29.24, 21.53, 13.08, 11.13. HRMS (ESI): $[M+H]^+$ calcd. for $C_{27}H_{32}N^+$: m/z = 370.2529; found, 370.2540.

N-(1-(naphthalen-1-yl)propan-2-yl)-*N*-phenethyl-1-phenylcyclopropan-1-amine (**4qb**)

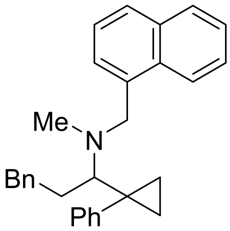

The titled compound was synthesized following the general procedure A. EtOAc/*n*-hexane (1:10); colorless oil (73 mg, 90%); ^{1}H NMR (600 MHz, $CDCl_3$): δ 7.85 (d, *J* = 8.2 Hz, 1H), 7.71 (d, *J* = 8.0 Hz, 1H), 7.61 (d, *J* = 8.1 Hz, 1H), 7.39-7.25 (m, 7H), 7.22-7.05 (m, 7H), 4.06-3.90 (m, 2H), 2.72-2.64 (m, 1H), 2.62-2.54 (m, 1H), 2.33 (dd, *J* = 9.2, 4.6 Hz, 1H), 2.03 (s, 3H), 2.02-1.96 (m, 4H), 1.90-1.83 (m, 1H), 0.85-0.79 (m, 1H), 0.71-0.65 (m, 1H), 0.59-0.53 (m, 1H), 0.50-0.45 (m, 1H). ^{13}C NMR (151 MHz, $CDCl_3$): δ 144.84, 142.98, 136.03, 133.86, 132.38, 131.66, 128.73, 128.56, 128.48, 127.90, 127.40, 126.46, 126.16, 125.84, 125.52, 125.50, 125.35, 124.50, 70.46, 56.98, 36.75, 34.66, 32.10, 29.45, 13.31, 10.76. HRMS (ESI): $[M+H]^+$ calcd. for $C_{30}H_{32}N^+$: m/z = 406.2529; found, 406.2537.

N, *N*-diethyl-3-phenyl-1-(1-phenylcyclopropyl)propan-1-amine (**4rb**)

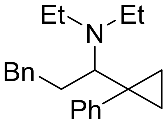

The titled compound was synthesized following the general procedure A. EtOAc/*n*-hexane (1:5); colorless oil (25 mg, 41%); ^{1}H NMR (600 MHz, $CDCl_3$): δ 7.42 (d, *J* = 7.5 Hz, 2H), 7.29 (t, *J* = 7.5 Hz, 2H), 7.26-7.22 (m, 2H), 7.22-7.16 (m, 4H), 2.79-2.65 (m, 2H), 2.43 (m, 4H), 2.30 (t, *J* = 7.0 Hz, 1H), 1.92-1.86 (m, 2H), 0.93 (t, *J* = 7.1 Hz, 6H), 0.89-0.85 (m, 1H), 0.83-0.78 (m, 1H), 0.65 (t, *J* = 7.4 Hz, 2H). ^{13}C NMR (151 MHz, $CDCl_3$): δ 145.01, 143.12, 131.46, 128.46, 128.37, 127.65, 126.24, 125.71, 67.28, 44.29, 34.46, 32.02, 29.61, 15.37, 12.32, 11.69. HRMS (ESI): $[M+H]^+$ calcd. for $C_{22}H_{30}N^+$: m/z = 308.2373; found, 308.2383.

N-butyl-*N*-(3-phenyl-1-(1-phenylcyclopropyl)propyl)butan-1-amine (**4sb**)

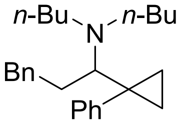

The titled compound was synthesized following the general procedure A. EtOAc/*n*-hexane (1:10); colorless oil (60 mg, 83%); ^{1}H NMR (600 MHz, $CDCl_3$): δ 7.38-7.34 (m, 2H), 7.24 (t, *J* = 7.5 Hz, 2H), 7.20-7.11 (m, 6H), 2.74-2.59 (m, 2H), 2.31-2.18 (m, 5H), 1.87-1.80 (m, 2H), 1.27-1.21 (m, 4H), 1.16-1.08 (m, 4H), 0.84-0.77 (m, 7H), 0.76-0.72 (m, 1H), 0.60 (t, *J* = 7.4 Hz, 2H). ^{13}C NMR (151 MHz, $CDCl_3$): δ 145.09, 143.18, 131.43, 128.46, 128.37, 127.63, 126.22, 125.70, 67.56, 51.37, 34.66, 32.29, 31.91, 29.59, 20.56, 14.25, 12.29, 11.70. HRMS (ESI): $[M+H]^+$ calcd. for $C_{26}H_{38}N^+$: m/z = 364.2999; found, 364.3007.

N-(2,2-dimethoxyethyl)-*N*-methyl-3-phenyl-1-(1-phenylcyclopropyl)propan-1-amine (**4tb**)

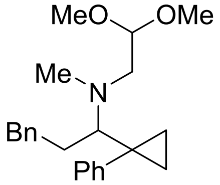

The titled compound was synthesized following the general procedure A. EtOAc/*n*-hexane (1:5); colorless oil (53 mg, 76%); ^{1}H NMR (600 MHz, $CDCl_3$) δ 7.33 (d, *J* = 7.5 Hz, 2H), 7.22 (t, *J* = 7.4 Hz, 2H), 7.18-7.13 (m, 4H), 7.13-7.08 (m, 2H), 4.20 (t, *J* = 5.2 Hz, 1H), 3.22 (d, *J* = 5.0 Hz, 6H), 2.77-2.70 (m, 1H), 2.58-2.51 (m, 2H), 2.47 (dd, *J* = 14.0, 5.2 Hz, 1H), 2.26 (s, 3H), 2.19 (dd, *J* = 9.7, 4.5 Hz, 1H), 1.92-1.78 (m, 2H), 0.87-0.79 (m, 2H), 0.57-0.53 (m, 1H), 0.50-0.46 (m, 1H). ^{13}C NMR (151 MHz, $CDCl_3$): δ 144.63, 143.08, 131.51, 128.55, 128.41, 127.85, 126.44, 125.75, 104.58, 71.57, 56.85, 53.95, 53.10, 38.85, 34.10, 32.55, 28.89, 13.07, 10.75. HRMS (ESI): $[M+H]^+$ calcd. for $C_{23}H_{32}NO_2^+$: m/z = 354.2428; found, 354.2433.

1-(3-phenyl-1-(1-phenylcyclopropyl)propyl)pyrrolidine (**4ub**)

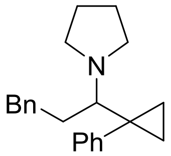

The titled compound was synthesized following the general procedure A. EtOAc/*n*-hexane (1:5); colorless oil (51 mg, 85%); ^{1}H NMR (400 MHz, $CDCl_3$): δ 7.47 (d, *J* = 7.3 Hz, 2H), 7.31-7.25 (m, 4H), 7.23-7.16 (m, 4H), 2.92-2.86 (m, 3H), 2.70-2.68 (m, 1H), 2.61-2.52 (m, 2H), 2.00-1.94 (m, 2H), 1.87 (s, 1H), 1.77-1.70 (m, 4H), 0.91-0.87 (m, 3H), 0.65-0.63 (m, 1H). ^{13}C NMR (151 MHz, $CDCl_3$): δ 143.59, 143.01, 130.86, 128.47, 128.43, 127.84, 126.31, 125.38, 71.25, 51.54, 34.80, 34.32, 27.66, 23.73, 14.90, 11.28. HRMS (ESI): $[M+H]^+$ calcd. for $C_{22}H_{28}N^+$: m/z = 306.2216; found, 306.2223.

1-(3-phenyl-1-(1-phenylcyclopropyl)propyl)piperidine (**4vb**)

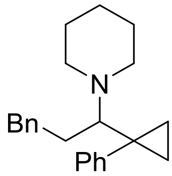

The titled compound was synthesized following the general procedure A. EtOAc/*n*-hexane (1:5); colorless oil (61 mg, 96%); ^{1}H NMR (600 MHz, $CDCl_3$): δ 7.42 (d, *J* = 7.6 Hz, 2H), 7.29-7.25 (m, 2H), 7.22 (t, *J* = 7.5 Hz, 2H), 7.18 (dd, *J* = 13.6, 7.3 Hz, 4H), 2.74-2.57 (m, 4H), 2.44-2.39 (m, 2H), 2.07-1.98 (m, 2H), 1.93-1.83 (m, 1H), 1.44 (t, *J* = 5.7 Hz, 4H), 1.40-1.37 (m, 2H), 0.93-0.87 (m, 1H), 0.87-0.81 (m, 1H), 0.63-0.57 (m, 1H), 0.55-0.49 (m, 1H). ^{13}C NMR (151 MHz, $CDCl_3$): δ 144.81, 143.04, 131.40, 128.55, 128.34, 127.63, 126.15, 125.69, 72.02, 51.04, 34.38, 32. 20, 28.85, 27.09, 25.20, 13.28, 11.54. HRMS (ESI): $[M+H]^+$ calcd. for $C_{23}H_{30}N^+$: m/z = 320.2373; found, 320.2382.

4-(3-phenyl-1-(1-phenylcyclopropyl)propyl)morpholine (**4wb**)

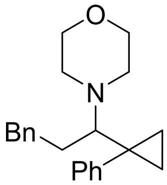

The titled compound was synthesized following the general procedure A. EtOAc/*n*-hexane (1:10); colorless oil (45 mg, 70%); ^{1}H NMR (600 MHz, $CDCl_3$): δ 7.44 (d, J = 7.5 Hz, 2H), 7.29 (t, J = 7.5 Hz, 2H), 7.26-7.23 (m, 2H), 7.21-7.16 (m, 4H), 3.60 (t, J = 4.7 Hz, 4H), 2.73-2.70 (m, 4H), 2.51-2.46 (m, 2H), 2.06-2.00 (m, 1H), 1.99-1.93 (m, 2H), 0.98-0.95 (m, 1H), 0.86-0.82 (m, 1H), 0.69-0.65 (m, 1H), 0.54-0.51 (m, 1H). ^{13}C NMR (151 MHz, $CDCl_3$): δ 144.06, 142.56, 131.20, 128.47, 128.41, 127.82, 126.37, 125.85, 71.77, 67.86, 50.31, 34.20, 32.00, 28.36, 13.88, 11.45. HRMS (ESI): $[M+H]^+$ calcd. for $C_{22}H_{28}NO^+$: m/z = 322.2165; found, 322.2171.

1-(3-phenyl-1-(1-phenylcyclopropyl)propyl)azepane (**4xb**)

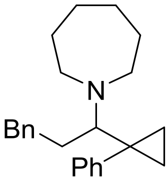

The titled compound was synthesized following the general procedure A. EtOAc/n-hexane (1:5); colorless oil (63 mg, 95%); ^{1}H NMR (400 MHz, $CDCl_3$): δ 7.29 (d, J = 7.1 Hz, 2H), 7.23-7.17 (m, 2H), 7.15 (d, J = 7.9 Hz, 2H), 7.12 (s, 2H), 7.11-7.06 (m, 2H), 2.81-2.71 (m, 1H), 2.69-2.62 (m, 2H), 2.60-2.48 (m, 1H), 2.48-2.38 (m, 2H), 2.20 (dd, J = 8.6, 5.5 Hz, 1H), 1.89-1.76 (m, 2H), 1.51-1.33 (m, 8H), 0.75 (t, J = 7.4 Hz, 2H), 0.54-0.46 (m, 1H), 0.46-0.38 (m, 1H). ^{13}C NMR (151 MHz, $CDCl_3$): δ 145.20, 143.21, 131.42, 128.50, 128.28, 127.61, 126.12, 125.58, 72.25, 51.81, 34.09, 32.93, 30.89, 29.61, 27.15, 12.79, 10.53. HRMS (ESI): $[M+H]^+$ calcd. for $C_{22}H_{28}NO^+$: m/z = 334.2529; found, 334.2533.

2-(3-phenyl-1-(1-phenylcyclopropyl)propyl)isoindoline (**4yb**)

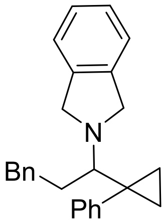

The titled compound was synthesized following the general procedure A, amine·HCl was used. EtOAc/*n*-hexane (1:10); colorless oil (39 mg, 56%); ^{1}H NMR (600 MHz, $CDCl_3$): δ 7.45-7.41 (m, 2H), 7.29 (t, J = 7.5 Hz, 2H), 7.25-7.15 (m, 10H), 4.33 (d, J = 11.2 Hz, 2H), 3.94 (d, J = 11.2 Hz, 2H), 2.90-2.82 (m, 1H), 2.79-2.72 (m, 1H), 2.27 (t, J = 6.8 Hz, 1H), 2.13-1.99 (m, 2H), 1.01-0.94 (m, 2H), 0.90-0.85 (m, 1H), 0.67-0.62 (m, 1H). ^{13}C NMR (151 MHz, $CDCl_3$): δ 143.36, 142.78, 140.28, 131.11, 128.52, 128.48, 127.93, 126.50, 126.41, 125.88, 122.35, 70.09, 55.97, 34.30, 34.09, 27.67, 14.78, 10.75. HRMS (ESI): $[M+H]^+$ calcd. for $C_{26}H_{28}N^+$: m/z = 354.2216; found, 354.2222.

2-(3-phenyl-1-(1-phenylcyclopropyl)propyl)-1,2,3,4-tetrahydroisoquinoline (**4zb**)

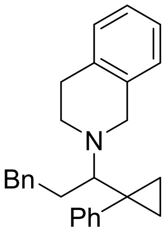

The titled compound was synthesized following the general procedure A. EtOAc/*n*-hexane (1:10); colorless oil (33 mg, 46%); ^{1}H NMR (600 MHz, $CDCl_3$): δ 7.32 (d, *J* = 7.5 Hz, 2H), 7.16 (t, *J* = 7.5 Hz, 2H), 7.13-7.02 (m, 6H), 7.00-6.96 (m, 2H), 6.96-6.91 (m, 1H), 6.88-6.84 (m, 1H), 4.00 (d, *J* = 14.9 Hz, 1H), 3.66 (d, *J* = 14.9 Hz, 1H), 2.70-2.65 (m, 1H), 2.64-2.52 (m, 4H), 2.45-2.38 (m, 1H), 2.14-2.08 (m, 1H), 2.08-1.99 (m, 1H), 1.95-1.86 (m, 1H), 0.91-0.85 (m, 1H), 0.82 (dt, *J* = 9.9, 5.1 Hz, 1H), 0.55 (dt, *J* = 9.6, 4.9 Hz, 1H), 0.46 (dt, *J* = 9.9, 5.1 Hz, 1H). ^{13}C NMR (151 MHz, $CDCl_3$): δ 144.25, 142.85, 136.39, 135.47, 131.48, 128.95, 128.55, 128.43, 127.83, 126.54, 126.46, 125.81, 125.79, 125.39, 71.30, 53.63, 45.55, 34.23, 32.20, 30.52, 28.68, 13.80, 11.53. HRMS (ESI): $[M+H]^+$ calcd. for $C_{26}H_{29}ClN^+$: m/z = 368.2373; found, 368.2375.

N-benzyl-3-phenyl-1-(1-phenylcyclopropyl)propan-1-amine (**8a**)

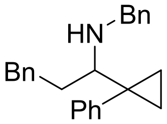

The titled compound was synthesized following the general procedure A, solvent in EA. EtOAc/*n*-hexane (1:5); colorless oil (47 mg, 69%); ^{1}H NMR (600 MHz, $CDCl_3$): δ 7.47-7.41 (m, 4H), 7.37 (t, *J* = 5.6 Hz, 2H), 7.32 (t, *J* = 5.5 Hz, 4H), 7.25 (d, *J* = 4.5 Hz, 2H), 7.22-7.15 (m, 3H), 4.26 (dd, *J* = 13.3, 4.0 Hz, 1H), 3.96 (dd, *J* = 13.3, 3.0 Hz, 1H), 2.88-2.81 (m, 1H), 2.74-2.66 (m, 1H), 2.08-2.01 (m, 1H), 1.99-1.91 (m, 1H), 1.60-1.51 (m, 1H), 1.44 (s, 1H), 0.98-0.92 (m, 1H), 0.88-0.77 (m, 2H), 0.55-0.50 (m, 1H). ^{13}C NMR (101 MHz, $CDCl_3$): δ 142.78, 142.10, 141.19, 131.25, 128.50, 128.39, 128.34, 128.16, 128.00, 126.87, 126.65, 125.68, 64.83, 51.91, 36.37, 33.39, 29.03, 13.51, 8.99. HRMS (ESI): $[M+H]^+$ calcd. for $C_{25}H_{28}N^+$: m/z = 342.2216; found, 342.2248.

N-(3-(10,11-dihydro-5H-dibenzo[*a*,*d*][7]annulen-5-ylidene)propyl)-*N*-methyl-3-phenyl-1-(1-phenylcyclopropyl)propan-1-amine (**9c**)

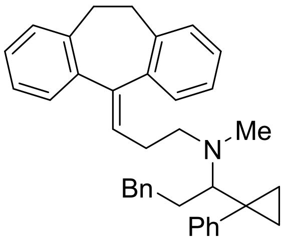

The titled compound was synthesized following the general procedure A, amine·HCl used. EtOAc/*n*-hexane (1:5); colorless oil (97 mg, 98%); ^{1}H NMR (600 MHz, $CDCl_3$): δ 7.38 (d, *J* = 7.3 Hz, 2H), 7.26 (t, *J* = 7.4 Hz, 3H), 7.19 (d, *J* = 7.7 Hz, 5H), 7.18-7.11 (m, 7H), 7.06-7.02 (m, 1H), 5.81 (t, *J* = 7.4 Hz, 1H), 3.45-3.24 (m, 2H), 2.96 (t, *J* = 13.7 Hz, 1H), 2.84-2.51 (m, 4H), 2.51-2.40 (m, 1H), 2.20 (d, *J* = 7.5 Hz, 2H), 2.18-2.09 (m, 4H), 1.93 (d, *J* = 49.9 Hz, 2H), 0.90-0.87 (m, 1H), 0.83-0.80 (m, 1H), 0.64-0.60 (m, 1H), 0.55-0.52 (m, 1H). ^{13}C NMR (151 MHz, $CDCl_3$): δ 144.67, 143.08, 142.93, 141.61, 140.33, 139.46, 137.14, 131.46, 130.35, 130.05, 128.70, 128.51, 128.40, 128.07, 127.77, 127.41, 127.03, 126.35, 126.10, 125.78, 71.15, 54.36, 53.93, 38.03, 34.33, 33.89, 32.20, 29.10, 28.92, 13.14, 11.53. HRMS (ESI): $[M+H]^+$ calcd. for $C_{37}H_{40}N^+$: m/z = 498.3155; found, 498.3161.

N-(3-(9,10-ethanoanthracen-9(10*H*)-yl)propyl)-*N*-methyl-3-phenyl-1-(1-phenylcyclopropyl)propan-1-amine (**9d**)

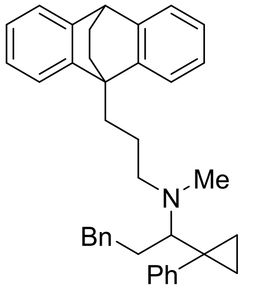

The titled compound was synthesized following the general procedure A, amine·HCl was used. EtOAc/*n*-hexane (1:1); colorless oil (75 mg, 74%); ^{1}H NMR (400 MHz, $CDCl_3$): δ 7.40 (dd, J = 7.5, 3.7 Hz, 2H), 7.22-7.09 (m, 12H), 7.04-6.96 (m, 4H), 4.18 (d, J = 3.3 Hz, 1H), 2.79-2.58 (m, 4H), 2.24 (dd, J = 26.2, 6.6 Hz, 6H), 2.04-1.95 (m, 1H), 1.93-1.86 (m, 1H), 1.80-1.69 (m, 4H), 1.49-1.42 (m, 2H), 0.94-0.80 (m, 2H), 0.65-0.51 (m, 2H). ^{13}C NMR (151 MHz, $CDCl_3$): δ 145.84, 145.76, 145.16, 144.82, 142.94, 131.52, 128.54, 128.48, 127.85, 126.39, 125.84, 125.33, 125.23, 123.40, 123.36, 121.56, 121.50, 71.45, 56.10, 44.91, 44.67, 37.97, 34.58, 32.02, 29.76, 29.29, 28.75,27.80,24.16,13.13,10.88. HRMS (ESI): $[M+H]^+$ calcd. for $C_{30}H_{32}FN_2O^+$: m/z = 512.3312; found, 512.3320.

(6-fluoro-3-(1-(3-phenyl-1-(1-phenylcyclopropyl)propyl)piperidin-4-yl)benzo[*d*]isoxazole (**9e**)

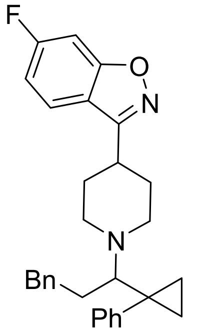

The titled compound was synthesized following the general procedure A, amine·HCl was used. EtOAc/*n*-hexane (1:5); colorless oil (88 mg, 97%); ^{1}H NMR (600 MHz, $CDCl_3$): δ 7.56 (dd, J = 8.7, 5.1 Hz, 1H), 7.36 (d, J = 7.5 Hz, 2H), 7.20 (t, J = 7.5 Hz, 2H), 7.15 (d, J = 9.5 Hz, 3H), 7.11 (dd, J = 12.1, 5.2 Hz, 4H), 6.98-6.92 (m, 1H), 2.94-2.84 (m, 1H), 2.80-2.71 (m, 3H), 2.70-2.61 (m, 1H), 2.61-2.54 (m, 1H), 2.34-2.27 (m, 1H), 2.05-1.94 (m, 2H), 1.90-1.81 (m, 3H), 1.77 (dd, J = 12.4, 3.7 Hz, 2H), 0.88-0.83 (m, 1H), 0.80-0.75 (m, 1H), 0.57-0.53 (m, 1H), 0.47-0.43 (m, 1H). ^{13}C NMR (151 MHz, $CDCl_3$): δ 164.17 (d, J = 251.0 Hz), 163.90 (d, J = 13.7 Hz), 161.58, 144.58, 142.78, 131.40, 128.55, 128.45, 127.81, 126.38, 125.84, 122.72 (d, J = 11.2 Hz), 117.51, 112.32 (d, J = 26.2 Hz), 97.48 (d, J = 26.3 Hz), 71.71, 51.08, 48.64, 35.24, 34.26, 32.07, 31.82, 31.55, 28.99, 13.34, 11.41. ^{19}F NMR (376 MHz, $CDCl_3$): δ −110.6 (s). HRMS (ESI): $[M+H]^+$ calcd. for $C_{30}H_{32}FN_2O^+$: m/z = 455.2493; found, 455.2499.

5,6-dimethoxy-2-((1-(3-phenyl-1-(1-phenylcyclopropyl)propyl)piperidin-4-yl)methyl)-2,3-dihydro-1*H*-inden-1-one (**9f**)

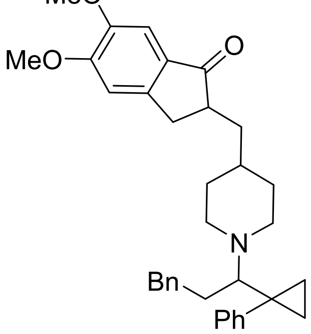

The titled compound was synthesized following the general procedure A, amine·HCl was used. EtOAc/*n*-hexane (1:5); colorless oil (41 mg, 40%); ^{1}H NMR (400 MHz, $CDCl_3$): δ 7.44 (d, *J* = 7.5 Hz, 2H), 7.29 (t, *J* = 7.4 Hz, 2H), 7.25-7.16 (m, 6H), 6.86 (s, 1H), 3.97 (s, 3H), 3.91 (s, 3H), 3.23 (dd, *J* = 17.5, 8.0 Hz, 1H), 2.74-2.66 (m, 7H), 2.26-2.20 (m, 1H), 2.07-2.03 (m, 2H), 1.94-1.84 (m, 2H), 1.64 (dd, *J* = 31.6, 12.1 Hz, 2H), 1.54-1.37 (m, 2H), 1.27-1.06 (m, 2H), 0.98-0.81 (m, 2H), 0.58 (d, *J* = 34.5 Hz, 2H). ^{13}C NMR (101 MHz, $CDCl_3$): δ 208.04, 155.45, 149.43, 148.86, 144.62, 142.88, 131.31, 129.37, 128.47, 128.29, 127.62, 126.13, 125.66, 107.38, 104.40, 71.57, 56.22, 56.11, 51.19, 51.10, 49.18, 49.02, 45.47, 39.01, 35.08, 34.27, 33.38, 32.19, 32.14, 14.22, 13.29, 11.46. HRMS (ESI): $[M+H]^+$ calcd. for $C_{35}H42NO_3^+$: m/z = 524.3159; found, 524.3166.

N-benzyl-*N*-methyl-3-phenyl-1-(1-(p-tolyl)cyclopropyl)propan-1-amine (**4ac**)

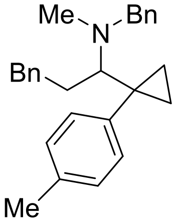

The titled compound was synthesized following the general procedure A. EtOAc/*n*-hexane (1:10); colorless oil (63 mg, 86%); ^{1}H NMR (600 MHz, $CDCl_3$): δ 7.19 (d, *J* = 7.8 Hz, 2H), 7.15-7.08 (m, 6H), 7.04 (dd, *J* = 11.7, 5.3 Hz, 4H), 6.90 (d, *J* = 7.7 Hz, 2H), 3.56-3.46 (m, 2H), 2.70-2.64 (m, 1H), 2.58-2.51 (m, 1H), 2.16 (s, 3H), 2.10 (dd, *J* = 9.3, 4.6 Hz, 1H), 1.98 (s, 3H), 1.96-1.88 (m, 1H), 1.83-1.76 (m, 1H), 0.80-0.75 (m, 1H), 0.70-0.65 (m, 1H), 0.49-0.43 (m, 1H), 0.40-0.35 (m, 1H). ^{13}C NMR (151 MHz, $CDCl_3$): δ 143.07, 141.70, 141.13, 135.98, 131.46, 128.60, 128.58, 128.50, 128.43, 128.22, 126.63, 125.85, 70.62, 58.77, 37.74, 34.48, 32.37, 28.46, 21.21, 13.28, 11.46. HRMS (ESI): $[M+H]^+$ calcd. for $C_{27}H_{32}N^+$: m/z = 370.2529; found, 370.2539.

N-benzyl-1-(1-(4-methoxyphenyl)cyclopropyl)-*N*-methyl-3-phenylpropan-1-amine (**4bc**)

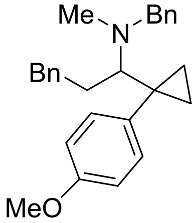

The titled compound was synthesized following the general procedure A. EtOAc/*n*-hexane (1:5); colorless oil (48 mg, 62%); ^{1}H NMR (600 MHz, $CDCl_3$): δ 7.32 (d, *J* = 8.4 Hz, 2H), 7.27-7.21 (m, 6H), 7.19-7.13 (m, 4H), 6.75 (d, 2H), 3.74 (s, 3H), 3.62 (dd, 2H), 2.82-2.74 (m, 1H), 2.69-2.61 (m, 1H), 2.18 (dd, *J* = 9.4, 4.5 Hz, 1H), 2.10 (s, 3H), 2.08-1.98 (m, 1H), 1.94-1.85 (m, 1H), 0.90-0.84 (m, 1H), 0.81-0.75 (m, 1H), 0.58-0.52 (m, 1H), 0.50-0.44 (m, 1H). ^{13}C NMR (151 MHz, $CDCl_3$): δ 158.18, 143.06, 141.13, 136.70, 132.51, 128.57, 128.49, 128.38, 128.21, 126.61, 125.83, 113.19, 70.61, 58.73, 55.34, 37.74, 34.44, 32.37, 28.09, 13.40, 11.50. HRMS (ESI): $[M+H]^+$ calcd. for $C_{27}H_{32}NO^+$: m/z = 386.2478; found, 386.2481.

N-benzyl-1-(1-(4-fluorophenyl)cyclopropyl)-*N*-methyl-3-phenylpropan-1-amine (**4cc**)

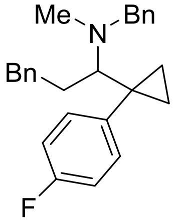

The titled compound was synthesized following the general procedure A. EtOAc/*n*-hexane (1:10); colorless oil (71 mg, 95%); ^{1}H NMR (600 MHz, $CDCl_3$): δ 7.42-7.38 (m, 2H), 7.32-7.26 (m, 4H), 7.25-

7.18 (m, 6H), 6.94 (t, J = 8.7 Hz, 2H), 3.71-3.61 (m, 2H), 2.85-2.77 (m, 1H), 2.73-2.65 (m, 1H), 2.28 (dd, J = 9.1, 4.7 Hz, 1H), 2.14 (s, 3H), 2.09-2.00 (m, 1H), 1.94-1.86 (m, 1H), 0.93-0.86 (m, 1H), 0.85-0.82 (m, 1H), 0.66-0.60 (m, 1H), 0.60-0.54 (m, 1H). ^{13}C NMR (151 MHz, $CDCl_3$): δ 162.34, 160.72, 142.77, 140.78, 140.37, 132.86, 132.81, 128.49, 128.32, 128.21, 127.13, 125.88, 114.63, 114.49, 70.19, 58.94, 37.60, 34.44, 32.01, 28.24, 12.64, 10.90. ^{19}F NMR (376 MHz, $CDCl_3$): δ −116.6 (s). HRMS (ESI): $[M+H]^+$ calcd. for $C_{26}H_{29}N^+$: m/z = 374.2279; found, 374.2284 .

N-benzyl-1-(1-(4-chlorophenyl)cyclopropyl)-*N*-methyl-3-phenylpropan-1-amine (**4dc**)

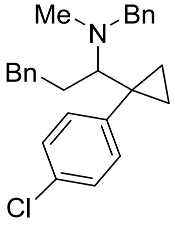

The titled compound was synthesized following the general procedure A. EtOAc/*n*-hexane (1:10); colorless oil (61 mg, 78%); ^{1}H NMR (600 MHz, $CDCl_3$): δ 7.40 (d, J = 8.1 Hz, 2H), 7.34-7.28 (m, 4H), 7.27-7.21 (m, 8H), 3.74-3.64 (m, 2H), 2.88-2.80 (m, 1H), 2.75-2.67 (m, 1H), 2.32 (dd, J = 9.2, 4.6 Hz, 1H), 2.16 (s, 3H), 2.13-2.03 (m, 1H), 1.95-1.87 (m, 1H), 0.95-0.84 (m, 2H), 0.68-0.58 (m, 2H). ^{13}C NMR (151 MHz, $CDCl_3$): δ 143.43, 142.78, 140.81, 132.78, 132.12, 128.55, 128.38, 128.26, 128.00, 126.74, 125.95, 70.16, 59.15, 37.68, 34.55, 32.04, 28.53, 13.18, 11.35. HRMS (ESI): $[M+H]^+$ calcd. for $C_{26}H_{29}ClN^+$: m/z = 390.1983; found, 390.1989.

N-benzyl-1-(1-(4-bromophenyl)cyclopropyl)-*N*-methyl-3-phenylpropan-1-amine (**4ec**)

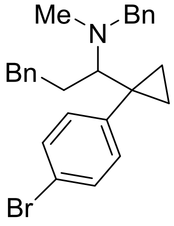

The titled compound was synthesized following the general procedure A. EtOAc/*n*-hexane (1:10); colorless oil (80 mg, 93%); ^{1}H NMR (600 MHz, $CDCl_3$): δ 7.35 (d, J = 8.2 Hz, 2H), 7.31-7.23 (m, 6H), 7.21-7.16 (m, 6H), 3.63 (dd, 2H), 2.82-2.76 (m, 1H), 2.70-2.62 (m, 1H), 2.27 (dd, J = 9.2, 4.6 Hz, 1H), 2.11 (s, 3H), 2.07-1.98 (m, 1H), 1.90-1.81 (m, 1H), 0.86-0.82 (m, 2H), 0.60-0.56 (m, 2H). ^{13}C NMR (151 MHz, $CDCl_3$): δ 143.92, 142.71, 140.72, 138.63, 133.12, 130.89, 128.48, 128.32, 128.20, 126.68, 125.89, 120.15, 70.05, 59.10, 37.61, 34.49, 31.96, 28.53, 13.07, 11.25. HRMS (ESI): $[M+H]^+$ calcd. for $C_{26}H_{29}BrN^+$: m/z = 434.1478; found, 434.1480.

N-benzyl-1-(1-(3-methoxyphenyl)cyclopropyl)-*N*-methyl-3-phenylpropan-1-amine (**4fc**)

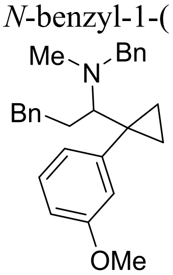

The titled compound was synthesized following the general procedure A. EtOAc/*n*-hexane (1:5); colorless oil (50 mg, 65%); ^{1}H NMR (600 MHz, $CDCl_3$): δ 6.95-6.89 (m, 6H), 6.87-6.79 (m, 5H), 6.72-6.65 (m, 2H), 6.39 (dd, J = 8.2, 2.6 Hz, 1H), 3.41 (s, 3H), 3.31 (dd, 2H), 2.51-2.43 (m, 1H), 2.40-2.31 (m, 1H), 1.95 (dd, J = 9.2, 4.6 Hz, 1H), 1.80 (s, 3H), 1.78-1.71 (m, 1H), 1.62-1.54 (m, 1H), 0.61-0.55 (m, 1H), 0.51-0.45 (m, 1H), 0.34-0.28 (m, 1H), 0.24-0.18 (m, 1H). ^{13}C NMR (151 MHz, $CDCl_3$): δ 159.10, 146.46, 142.95, 141.06, 128.74, 128.57, 128.49, 128.38, 128.20, 126.62, 125.85, 123.94, 117.46,

111.55, 70.43, 58.74, 55.29, 37.71, 34.49, 32.13, 28.98, 13.24, 11.04. HRMS (ESI): $[M+H]^+$ calcd. for $C_{27}H_{32}NO^+$: m/z = 386.2478; found, 386.2482.

N-benzyl-*N*-methyl-3-phenyl-1-(1-(thiophen-3-yl)cyclopropyl)propan-1-amine (**4gc**)

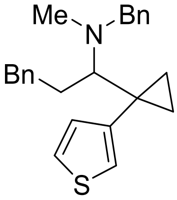

The titled compound was synthesized following the general procedure A. EtOAc/*n*-hexane (1:5); colorless oil (62 mg, 87%); ^{1}H NMR (600 MHz, $CDCl_3$): δ 7.30 (d, *J* = 5.6 Hz, 6H), 7.24-7.18 (m, 6H), 7.14-7.10 (m, 1H), 3.75-3.57 (m, 2H), 2.88-2.80 (m, 1H), 2.78-2.70 (m, 1H), 2.18 (s, 3H), 2.16-2.11 (m, 2H), 2.05-1.98 (m, 1H), 1.02-0.96 (m, 1H), 0.90-0.84 (m, 1H), 0.71-0.65 (m, 1H), 0.59-0.53 (m, 1H). ^{13}C NMR (151 MHz, $CDCl_3$): δ 145.36, 142.94, 140.98, 129.81, 128.56, 128.50, 128.45, 128.26, 126.68, 125.88, 124.52, 123.16, 70.57, 58.49, 38.15, 34.50, 33.02, 23.66, 14.45, 12.02. HRMS (ESI): $[M+H]^+$ calcd. for $C_{24}H_{28}NS^+$: m/z = 362.1937; found, 362.1944.

N-benzyl-*N*-methyl-3-phenyl-1-(1-vinylcyclopropyl)propan-1-amine (**4ic**)

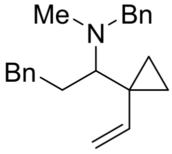

The titled compound was synthesized following the general procedure A. EtOAc/*n*-hexane (1:10); colorless oil (33 mg, 55%); ^{1}H NMR (600 MHz, $CDCl_3$): δ 7.32 (d, *J* = 7.1 Hz, 1H), 7.33-7.27 (m, 2H), 7.25 (d, *J* = 1.9 Hz, 1H), 7.22-7.14 (m, 5H), 6.34 (dd, *J* = 17.2, 10.6 Hz, 1H), 4.96-4.88 (m, 2H), 3.83 (d, *J* = 13.5 Hz, 1H), 3.61 (d, *J* = 13.5 Hz, 1H), 2.79-2.70 (m, 2H), 2.25 (s, 3H), 2.14-2.05 (m, 1H), 1.96-1.90 (m, 2H), 0.89-0.86 (m, 1H), 0.74-0.70 (m, 1H), 0.55-0.51 (m, 1H), 0.41-0.38 (m, 1H). ^{13}C NMR (151 MHz, $CDCl_3$): δ 143.02, 140.94, 128.64, 128.56, 128.51, 128.45, 128.28, 126.74, 125.81, 111.75, 70.00, 58.46, 39.47, 33.93, 32.59, 25.28, 14.25, 13.97. HRMS (ESI): $[M+H]^+$ calcd. for $C_{22}H_{27}N^+$: m/z = 306.2216; found, 306.2224.

1-(1-(1*H*-pyrrol-1-yl)cyclopropyl)-*N*-benzyl-*N*-methyl-3-phenylpropan-1-amine (**4jc**)

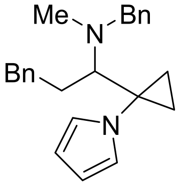

The titled compound was synthesized following the general procedure A. EtOAc/*n*-hexane (1:5); colorless oil (65 mg, 94%); ^{1}H NMR (600 MHz, $CDCl_3$): δ 7.30-7.25 (m, 6H), 7.23-7.17 (m, 4H), 6.80 (t, *J* = 2.2 Hz, 2H), 6.06 (t, *J* = 2.2 Hz, 2H), 3.64 (dd, 2H), 2.84-2.76 (m, 1H), 2.69-2.61 (m, 1H), 2.32 (dd, *J* = 9.7, 4.6 Hz, 1H), 2.16-2.09 (m, 1H), 2.08 (s, 3H), 1.94-1.85 (m, 1H), 1.22-1.15 (m, 1H), 0.98-0.91 (m, 1H), 0.90-0.84 (m, 1H), 0.69-0.63 (m, 1H). ^{13}C NMR (151 MHz, $CDCl_3$): δ 142.49, 140.72, 128.54, 128.52, 128.42, 128.27, 126.77, 125.98, 122.36, 109.42, 67.74, 58.01, 42.54, 36.48, 33.99, 31.58, 14.05, 11.95. HRMS (ESI): $[M+H]^+$ calcd. for $C_{24}H_{29}N_2^+$: m/z = 345.2325; found, 345.2334.

tert-butyl (1-(1-(benzyl(methyl)amino)-3-phenylpropyl)cyclopropyl)carbamate (**4kc**)

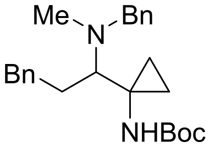

The titled compound was synthesized following the general procedure A. EtOAc/*n*-hexane (1:2); white solid (38 mg, 48%). mp 153.5-156.7 °C; ^{1}H NMR (400 MHz, $CDCl_3$): δ 7.38-7.16 (m, 10H), 4.95 (s, 1H), 3.92 (d, *J* = 13.8 Hz, 1H), 3.78 (d, *J* = 13.7 Hz, 1H), 2.91-2.81 (m, 1H), 2.81-2.72 (m, 1H), 2.57 (dd, *J* = 8.7, 4.9 Hz, 1H), 2.36 (s, 3H), 1.97-1.85 (m, 1H), 1.73-1.62 (m, 1H), 1.45 (s, 9H), 0.95 (s, 1H), 0.86-0.71 (m, 2H), 0.67-0.57 (m, 1H). ^{13}C NMR (151 MHz, $CDCl_3$): δ 155.35, 142.88, 140.85, 128.56, 128.47, 128.44, 128.33, 126.80, 125.82, 79.21, 66.82, 58.99, 37.51, 35.01, 33.99, 30.72, 28.58, 13.40, 12.00. HRMS (ESI): $[M+H]^+$ calcd. for $C_{25}H_{35}N_2O_2^+$: m/z = 395.2693; found, 395.2701.

N-benzyl-1-(1-methoxycyclopropyl)-*N*-methyl-3-phenylpropan-1-amine (**4lc**)

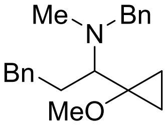

The titled compound was synthesized following the general procedure A. EtOAc/*n*-hexane (1:5); colorless oil (43 mg, 69%); ^{1}H NMR (600 MHz, $CDCl_3$): δ 7.40 (d, *J* = 7.5 Hz, 2H), 7.35-7.28 (m, 4H), 7.25-7.18 (m, 4H), 3.99 (d, *J* = 13.7 Hz, 1H), 3.83 (d, *J* = 13.8 Hz, 1H), 3.28 (s, 3H), 2.99-2.92 (m, 1H), 2.83 (dd, *J* = 9.6, 4.3 Hz, 1H), 2.76-2.68 (m, 1H), 2.39 (s, 3H), 1.90-1.80 (m, 1H), 1.60 (d, *J* = 56.2 Hz, 1H), 0.85-0.78 (m, 1H), 0.76-0.69 (m, 1H), 0.62-0.56 (m, 1H), 0.56-0.50 (m, 1H). ^{13}C NMR (151 MHz, $CDCl_3$): δ 142.89, 141.21, 128.56, 128.45, 128.45, 128.25, 126.67, 125.81, 63.79, 63.55, 59.19, 54.74, 37.80, 34.02, 31.06, 10.56, 10.05. HRMS (ESI): $[M+H]^+$ calcd. for $C_{21}H_{28}NO^+$: m/z = 310.2165; found, 310.2189.

11. Copies of Product ^{1}H NMR and ^{13}C NMR

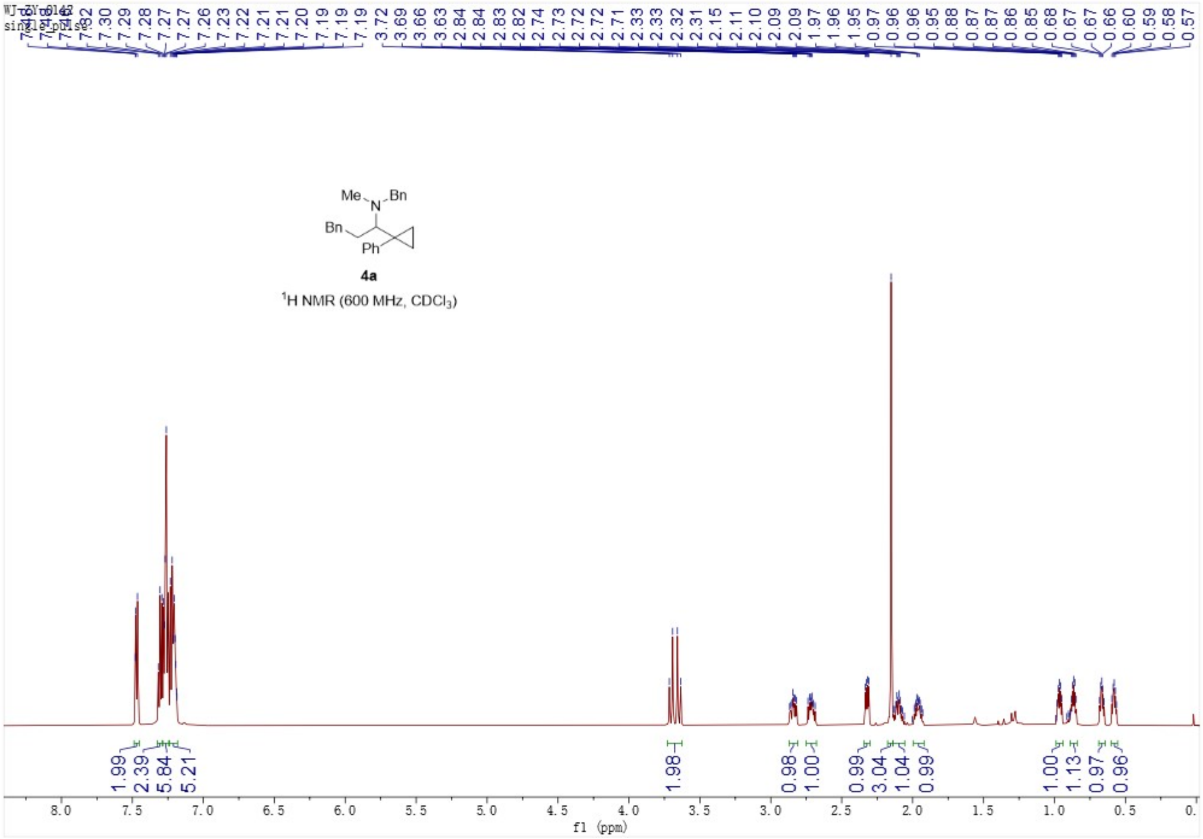

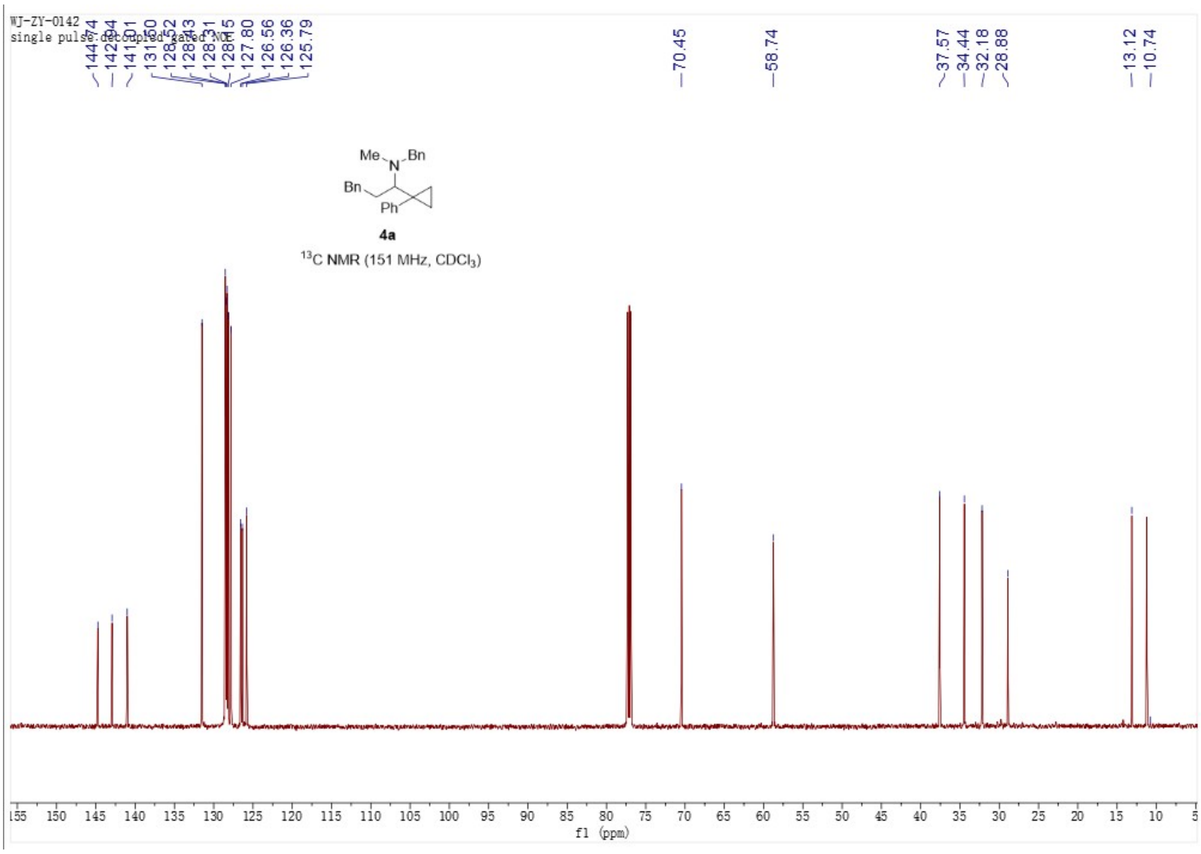

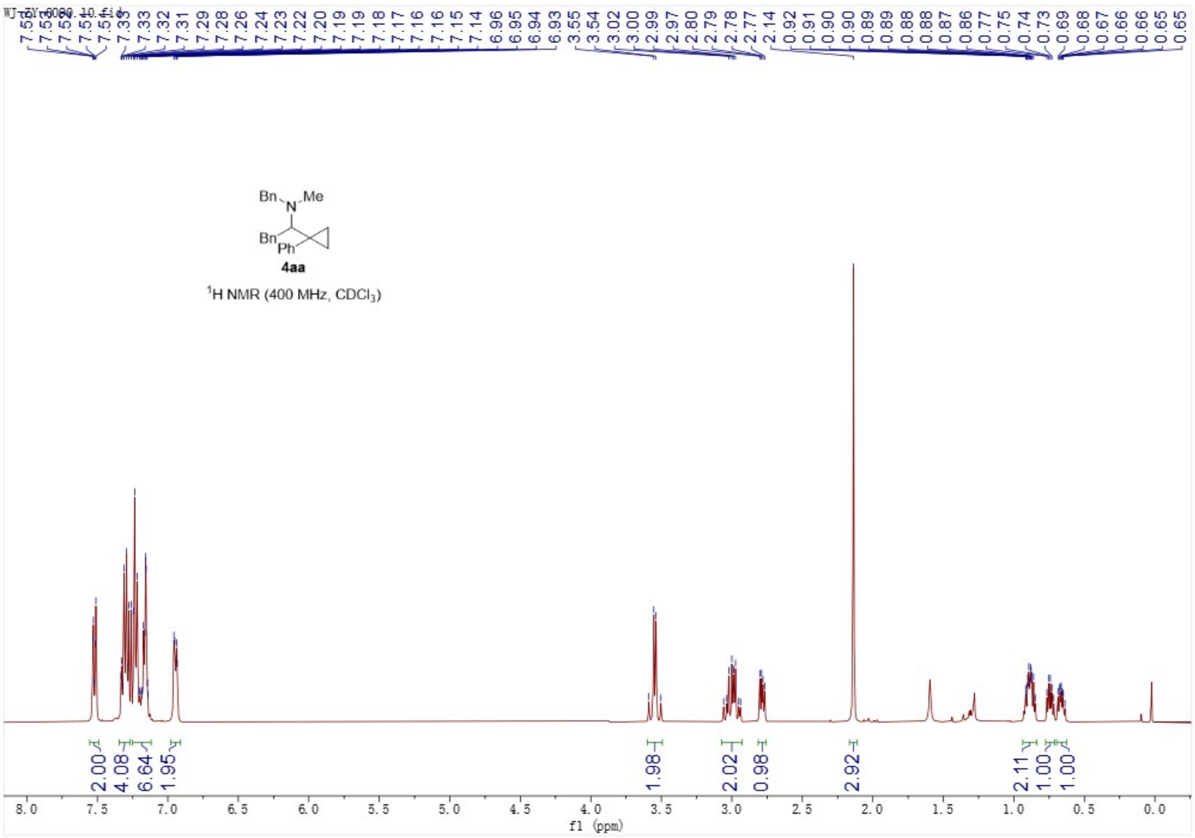

4aa
^{1}H NMR (400 MHz, CDCl$_3$)
f1 (ppm)

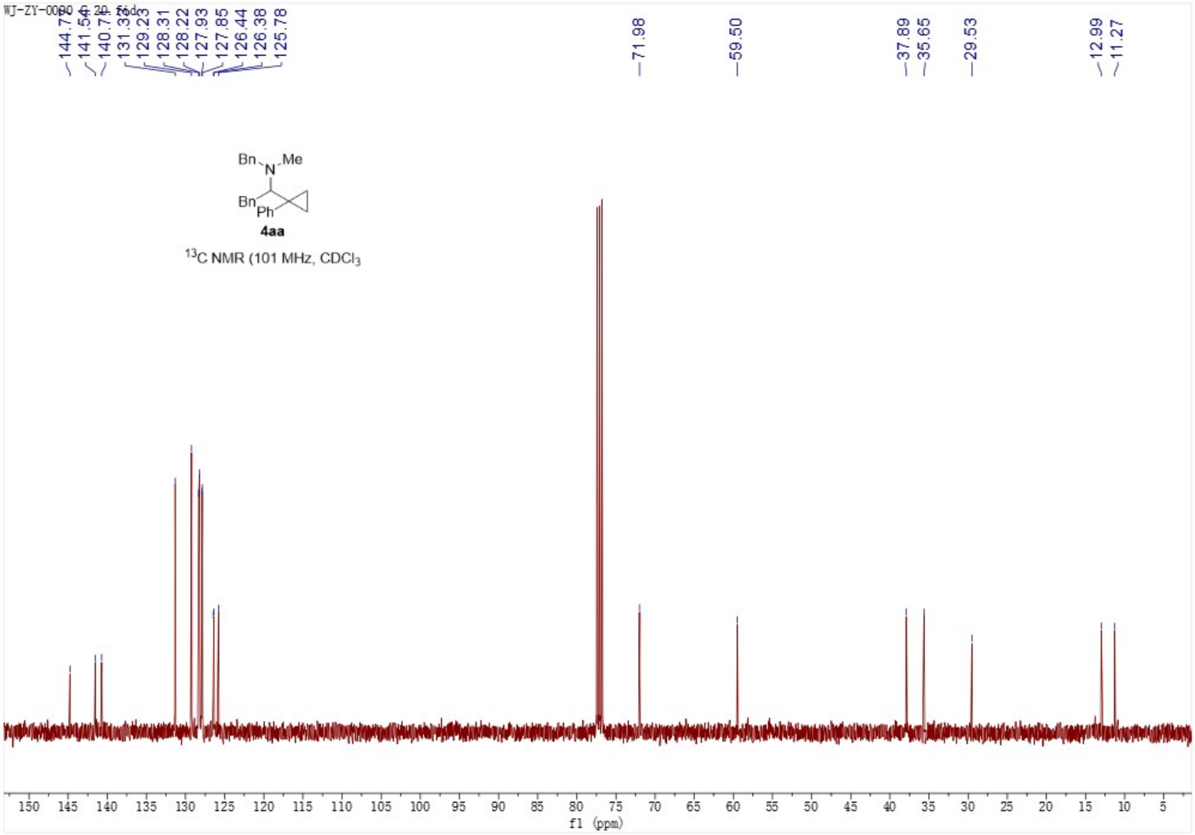

4aa
^{13}C NMR (101 MHz, CDCl$_3$
f1 (ppm)

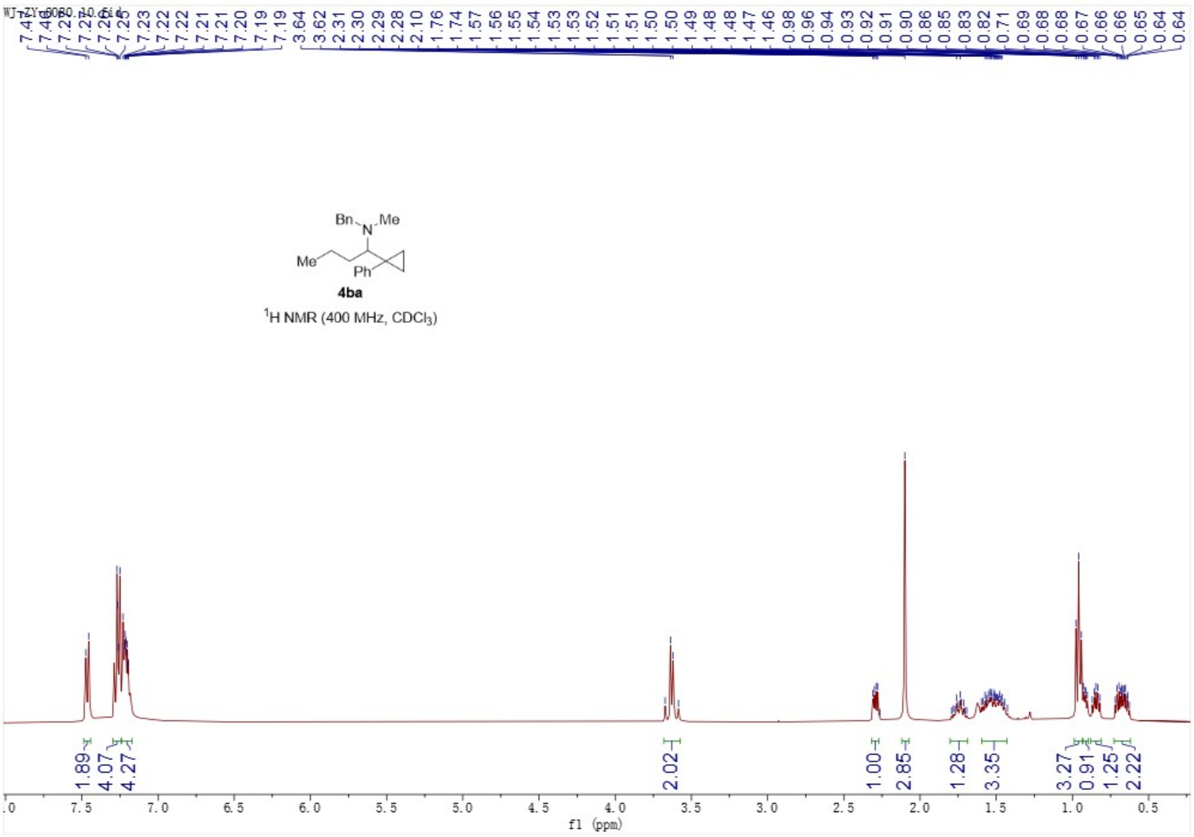

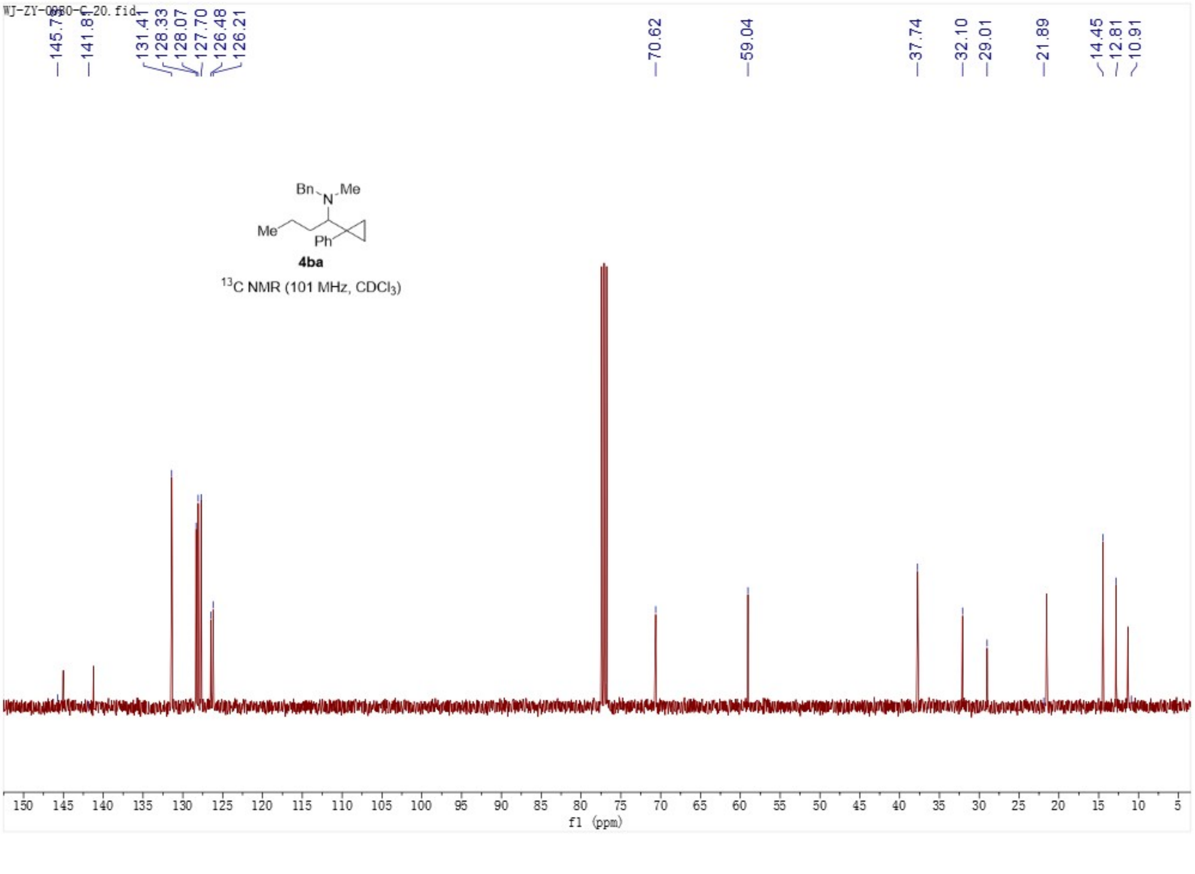

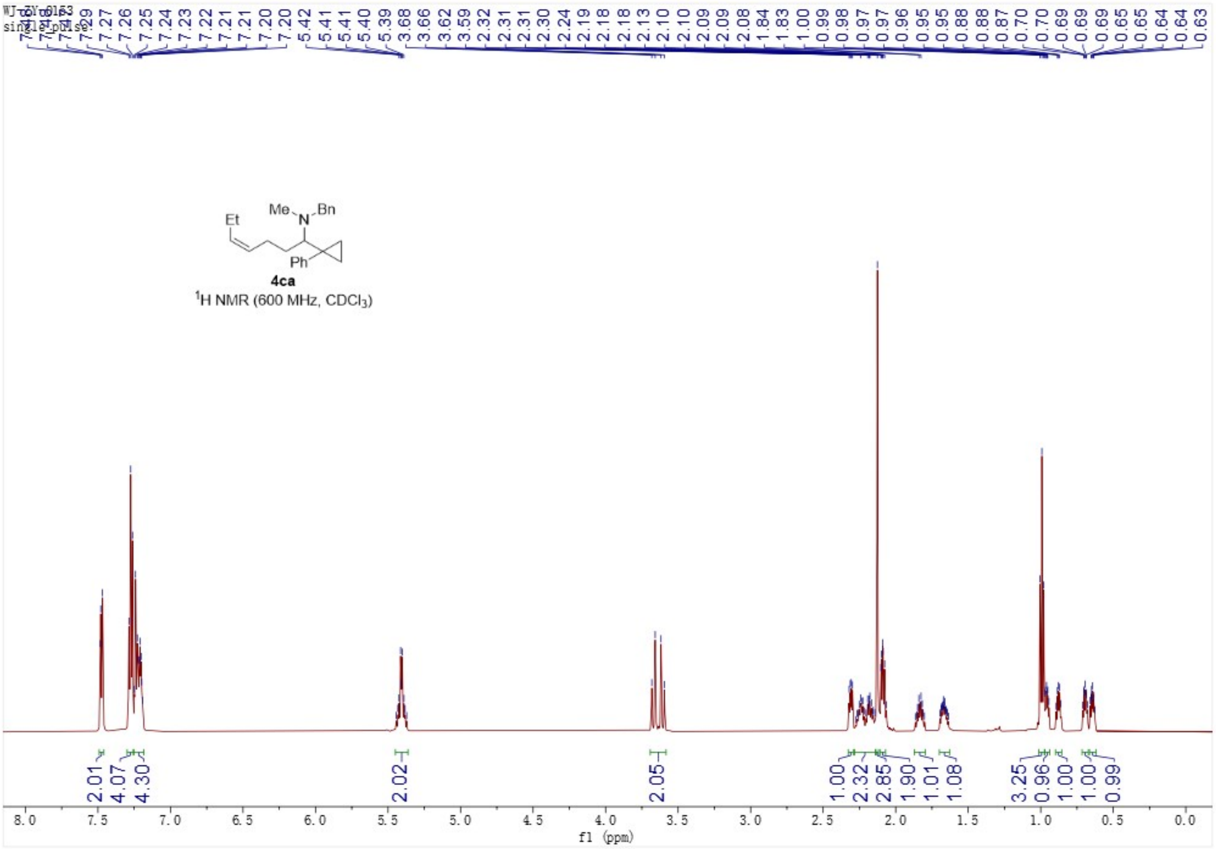

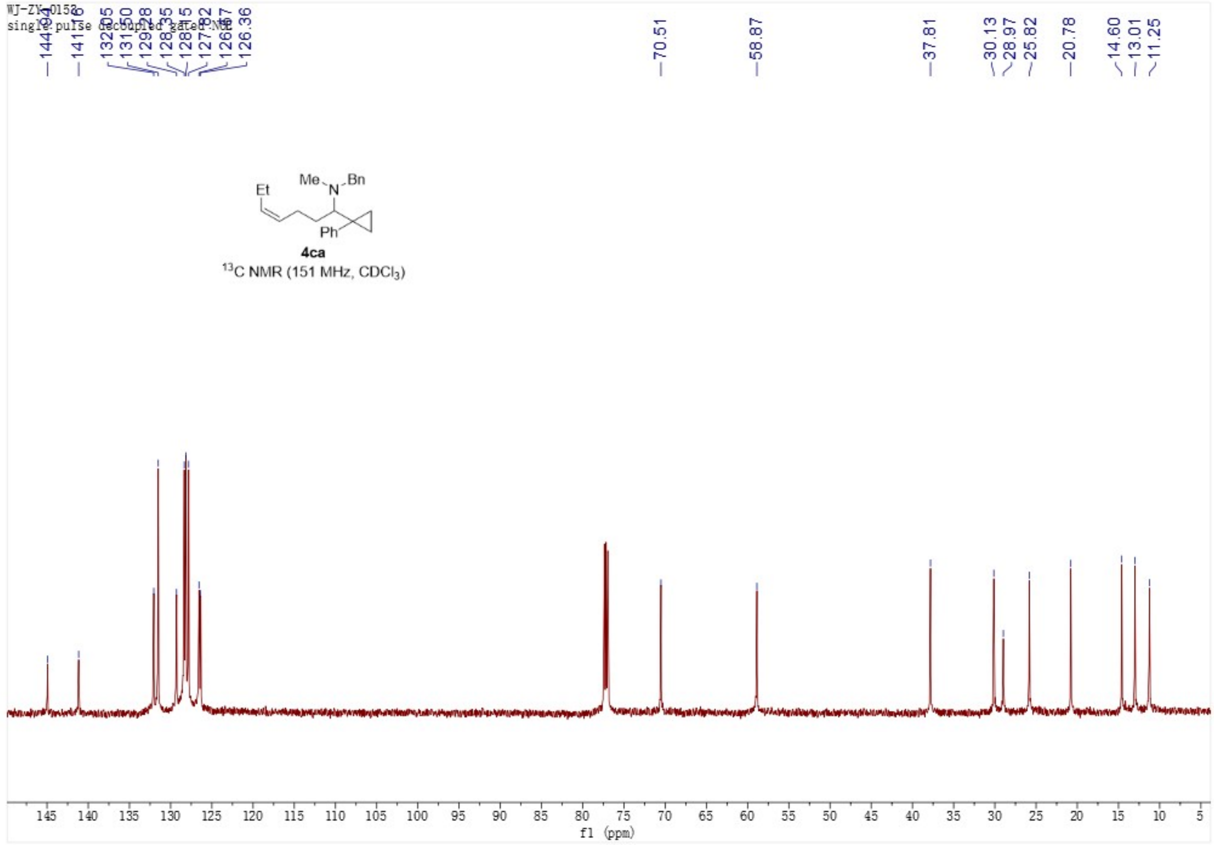

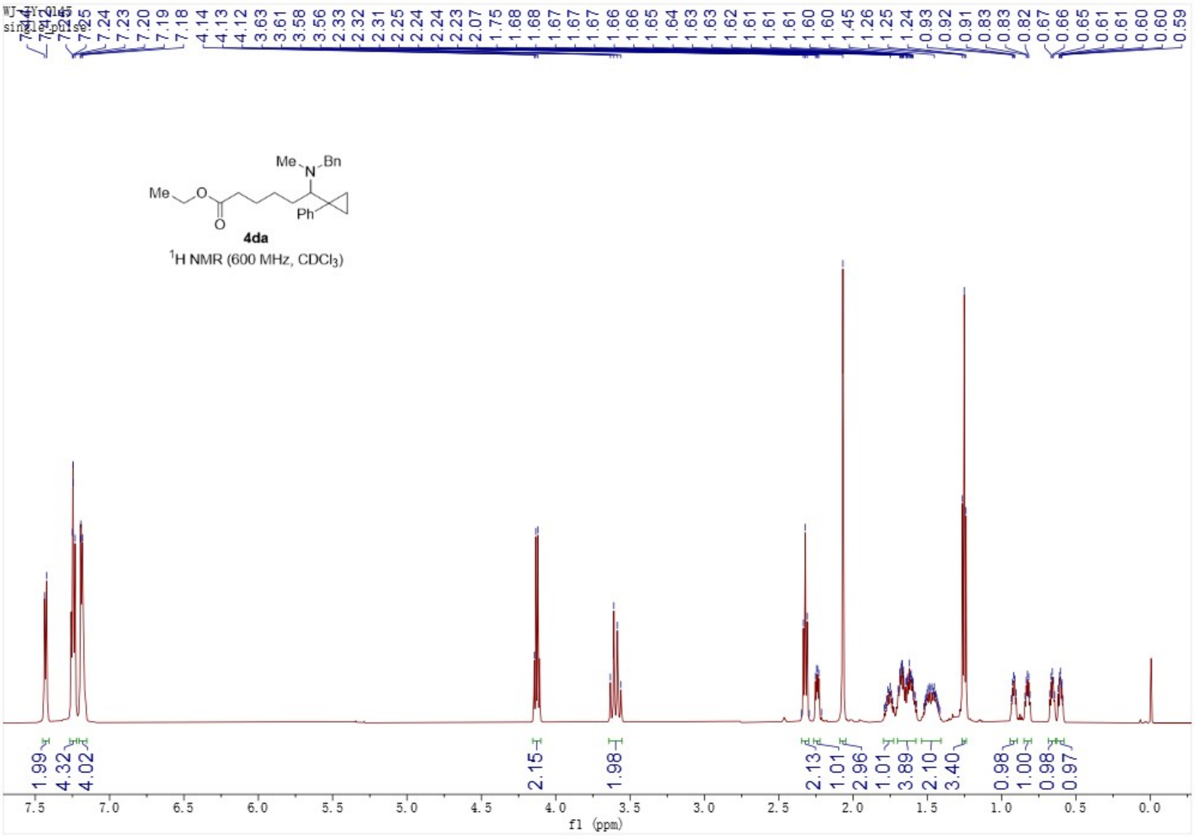
WJ-ZY-0145
single pulse
4da
^{1}H NMR (600 MHz, CDCl$_3$)
f1 (ppm)

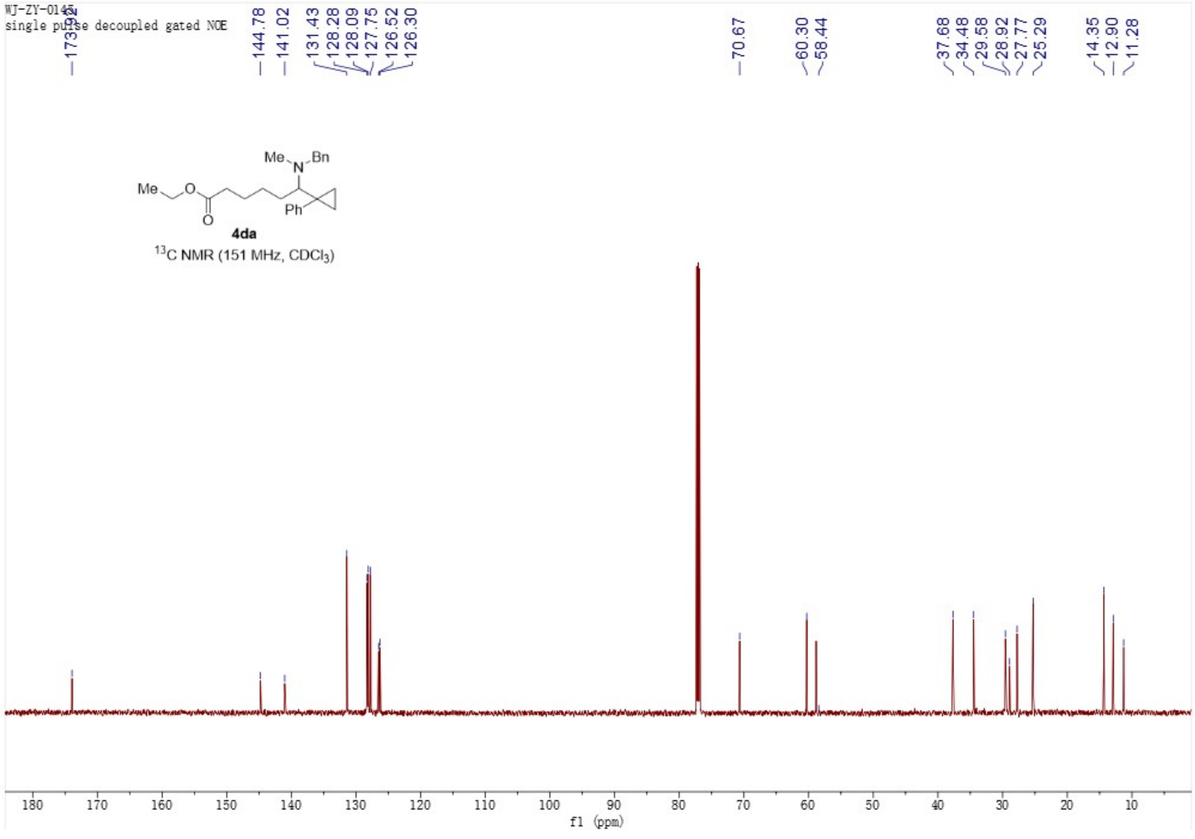
WJ-ZY-0145
single pulse decoupled gated NOE
4da
^{13}C NMR (151 MHz, CDCl$_3$)
f1 (ppm)

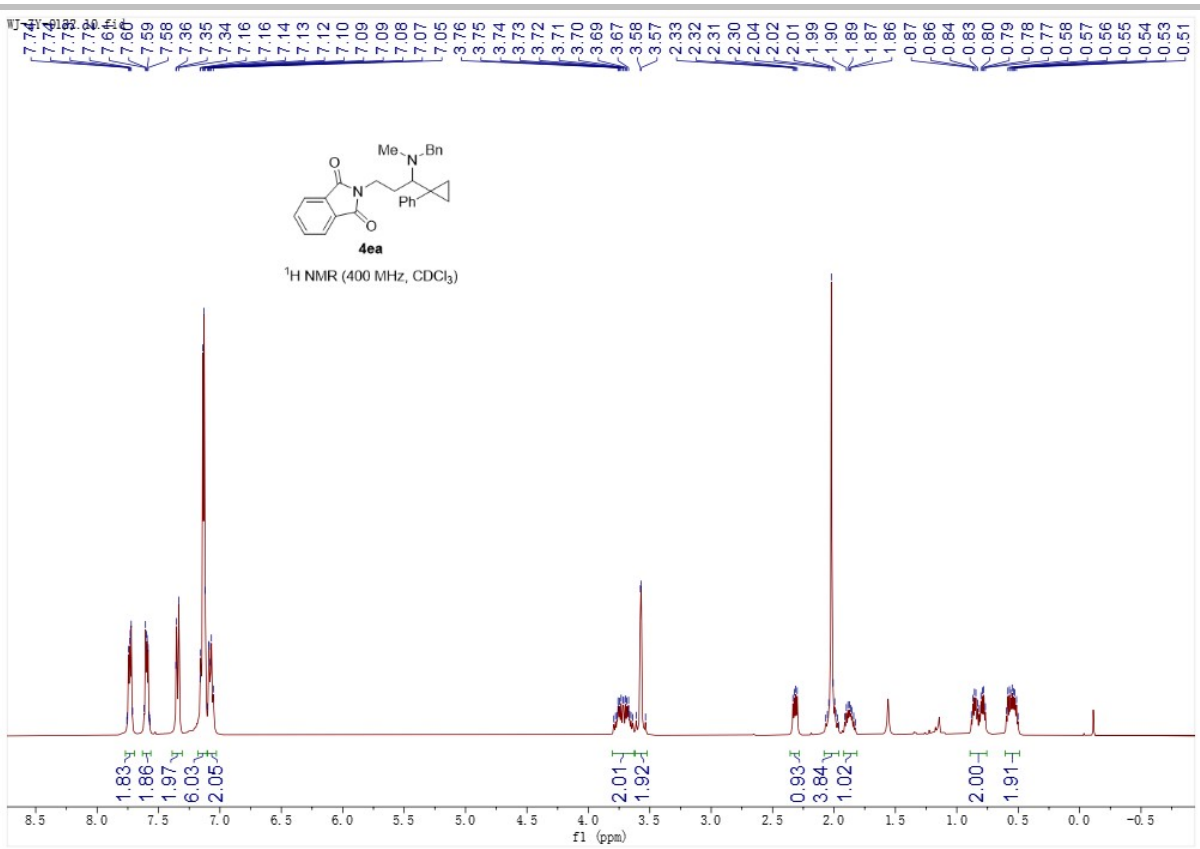

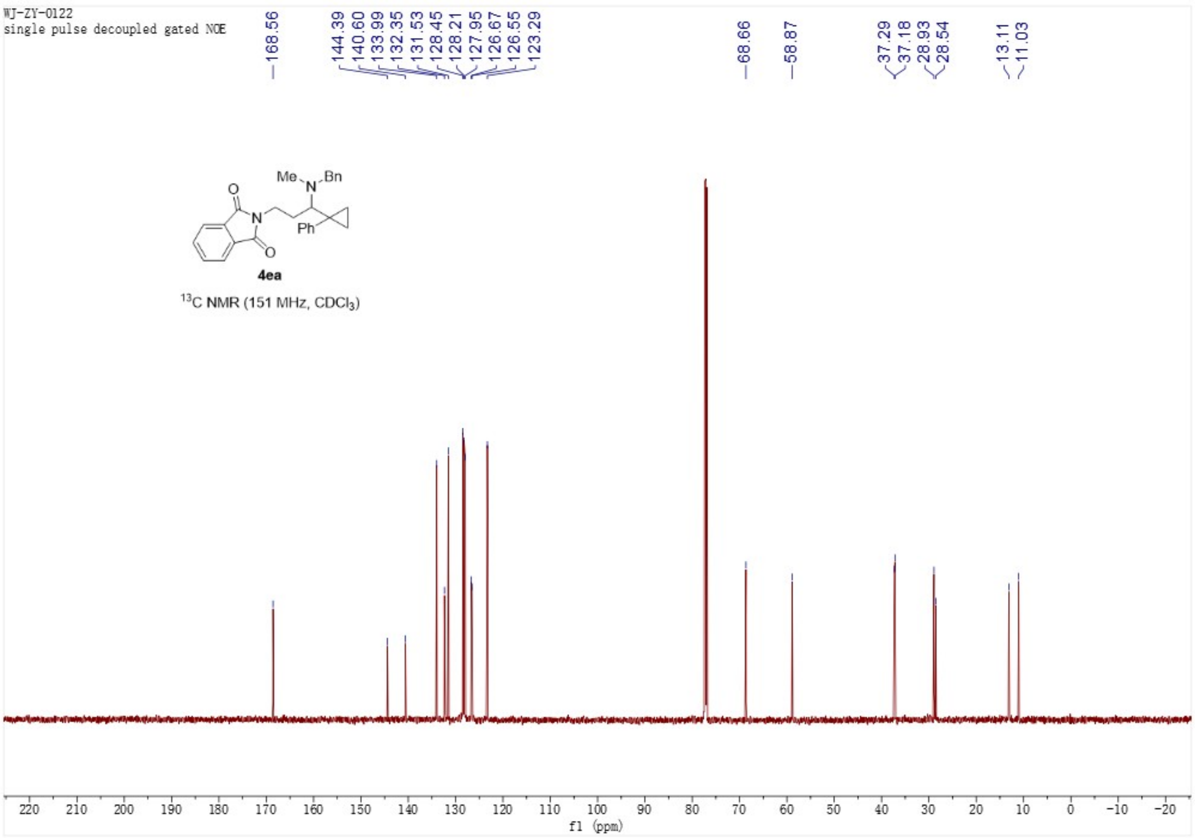

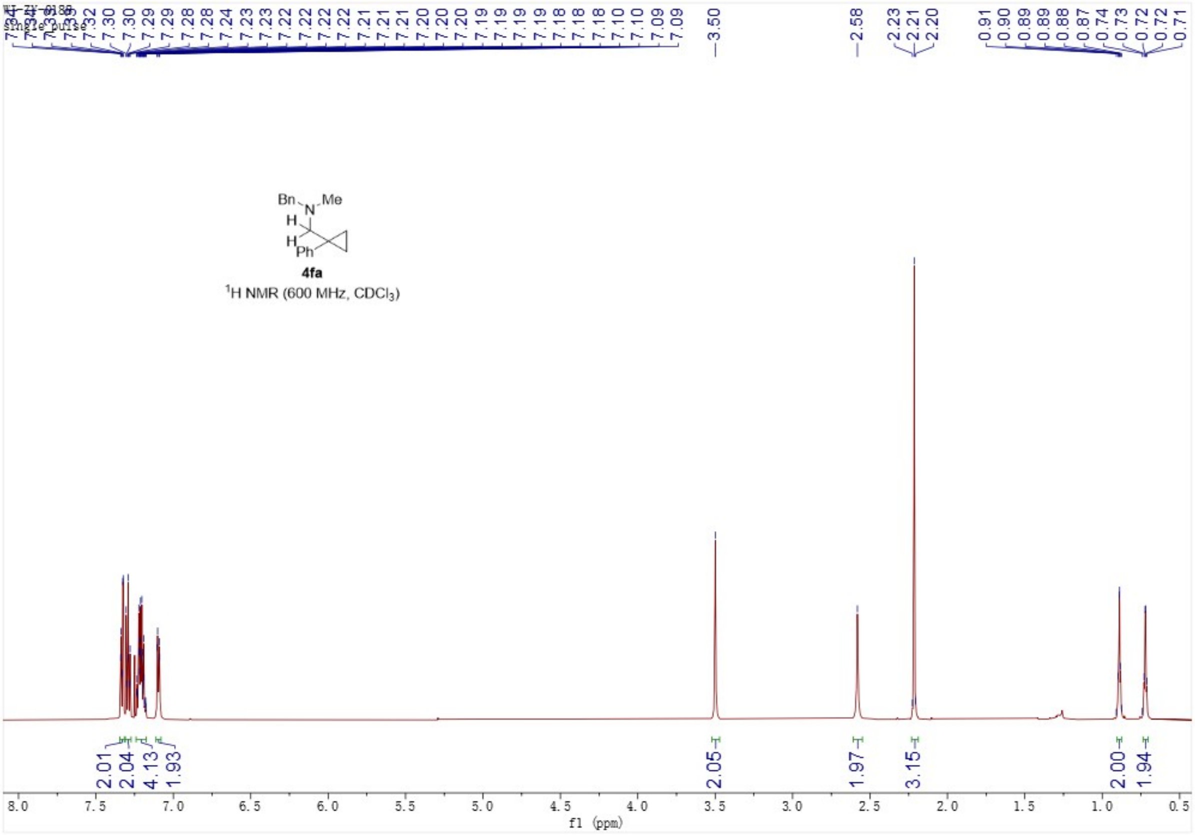

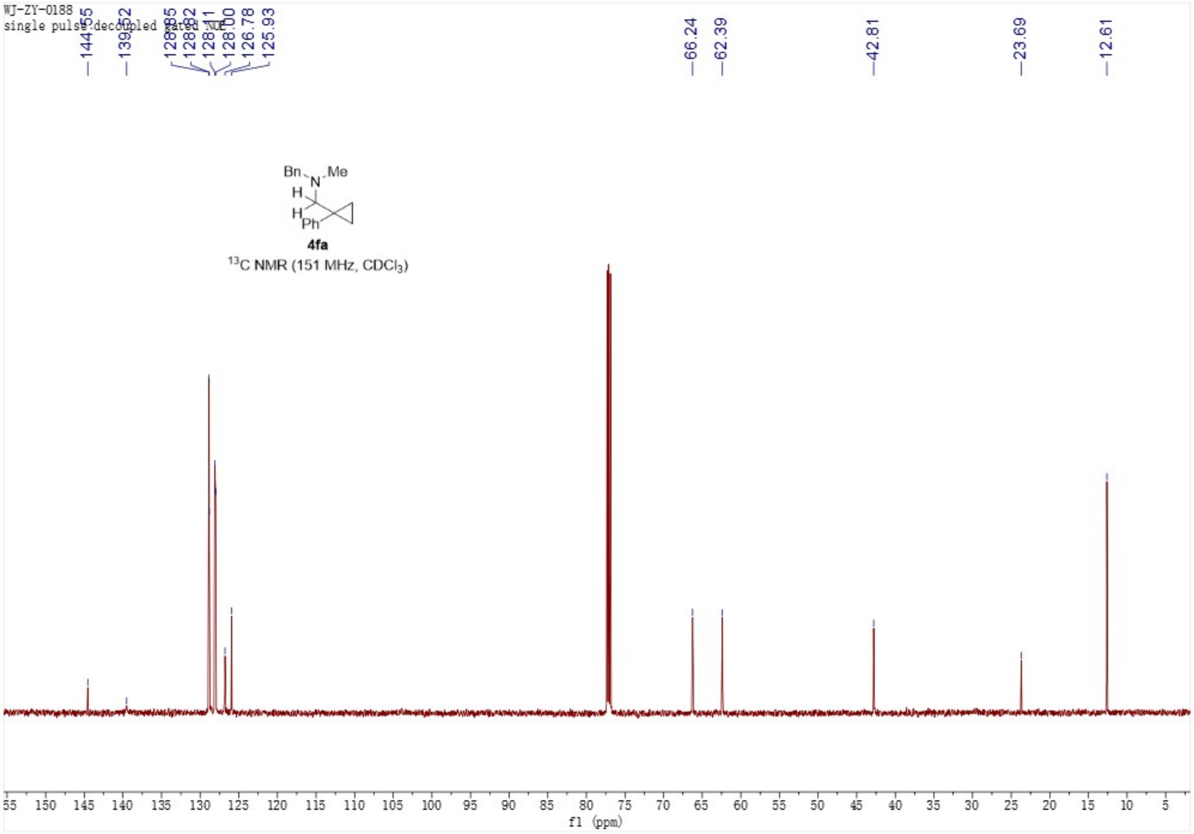

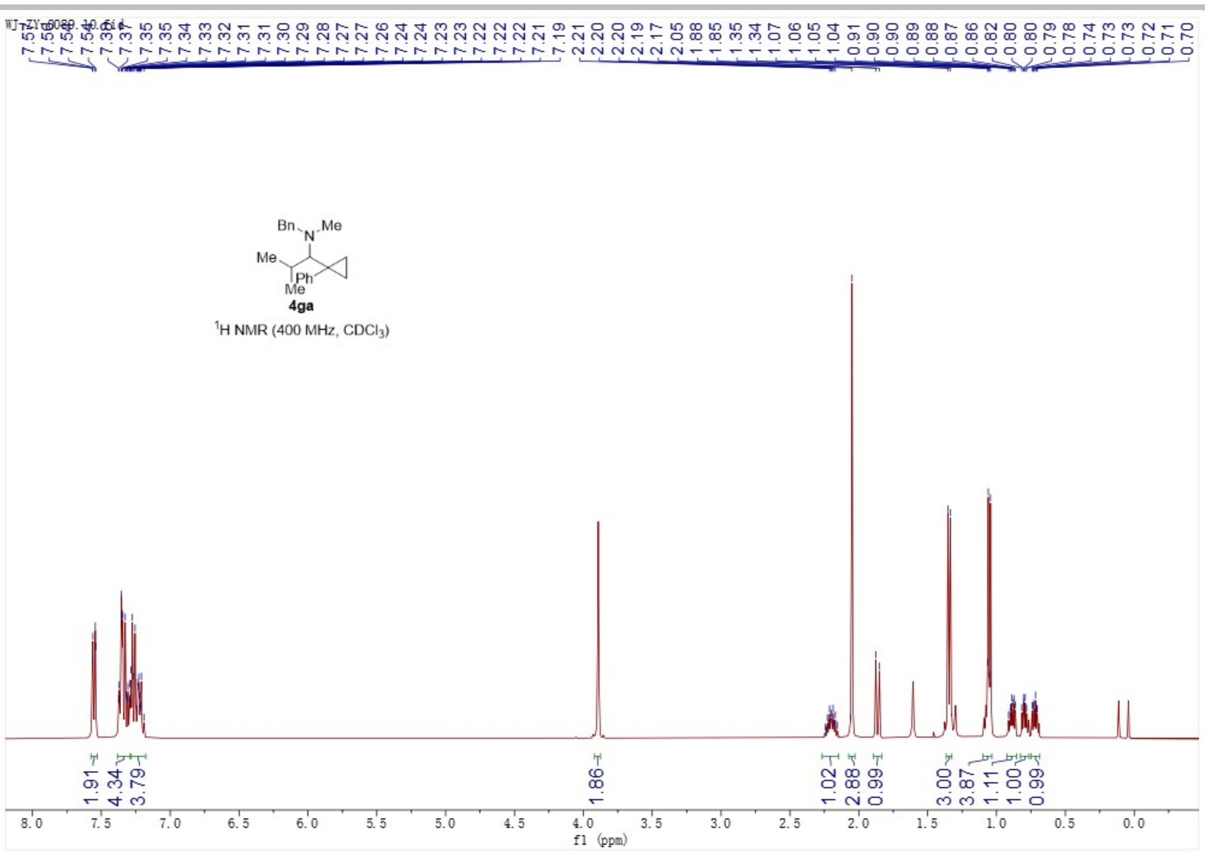
Bn N Me
Me Ph Me
4ga
^{1}H NMR (400 MHz, CDCl$_3$)
fl (ppm)

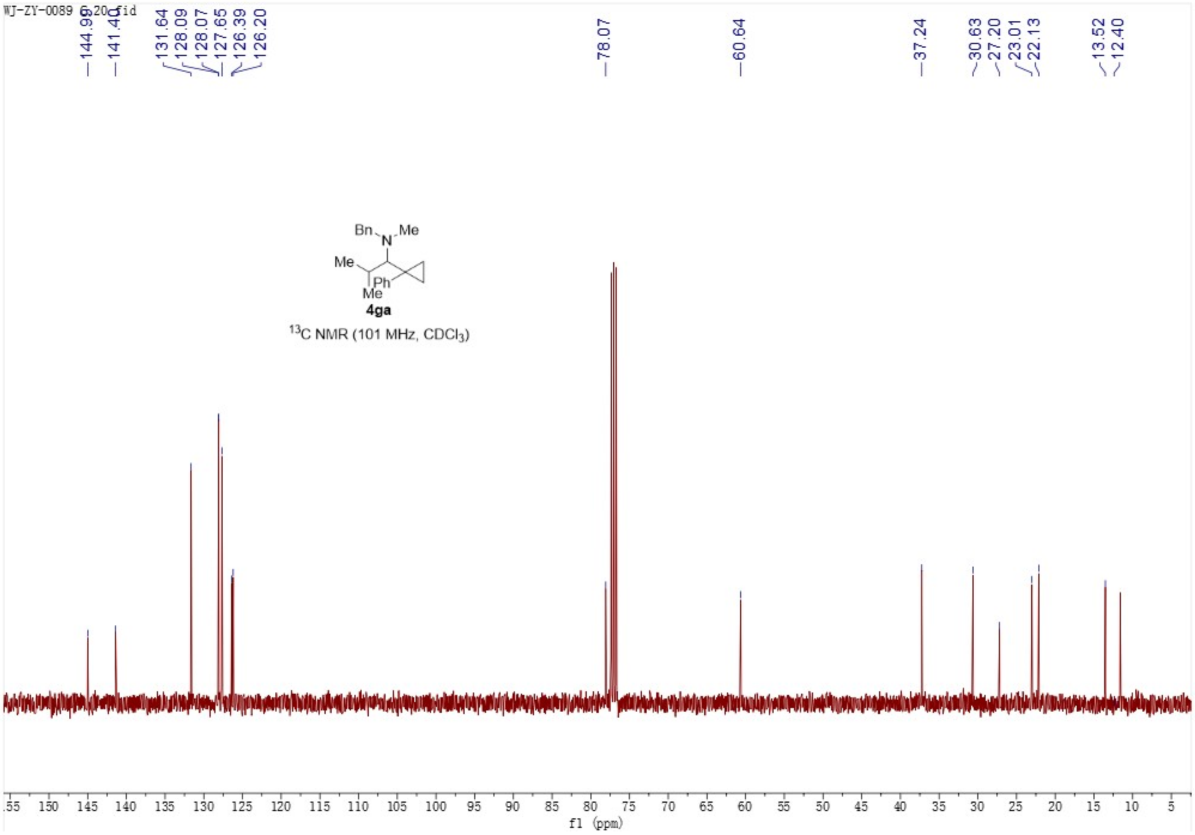
Bn N Me
Me Ph Me
4ga
^{13}C NMR (101 MHz, CDCl$_3$)
fl (ppm)

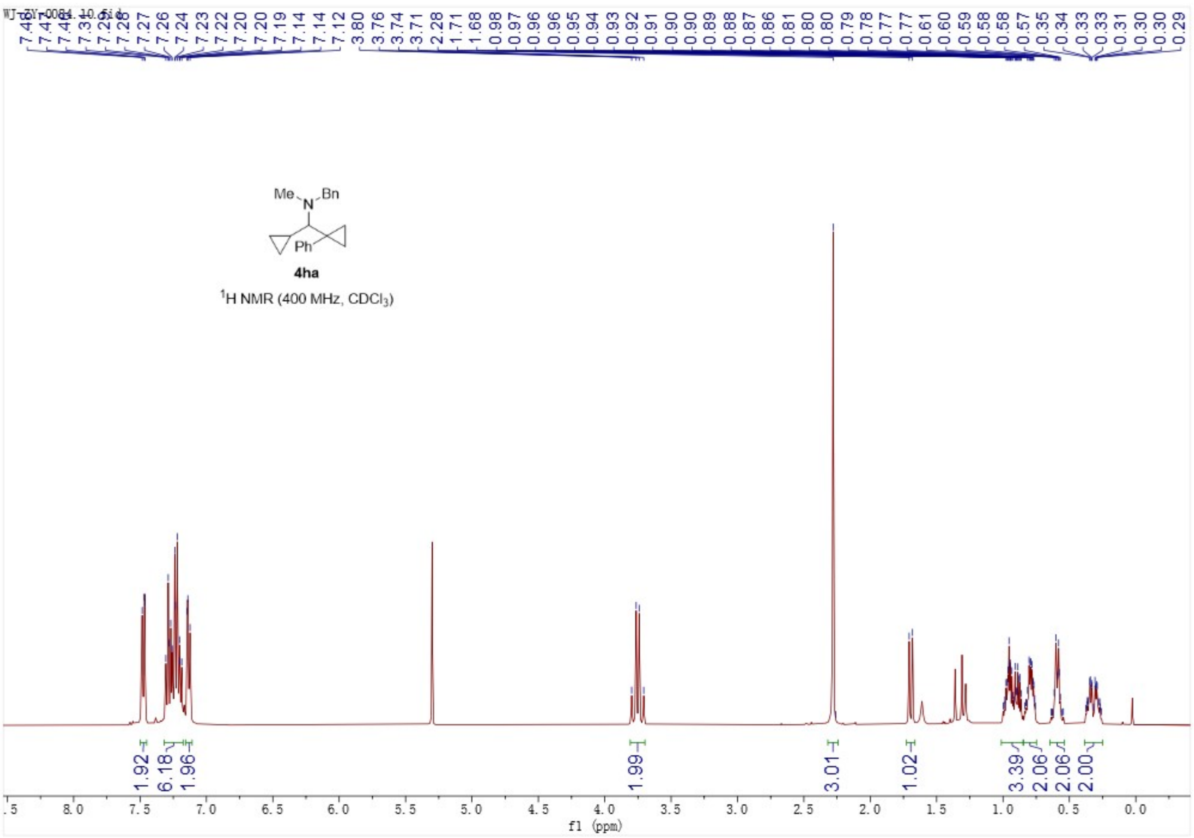
WJ-ZY-0084.10.fid
Me
N
Bn
Ph
4ha
^{1}H NMR (400 MHz, CDCl$_3$)
f1 (ppm)

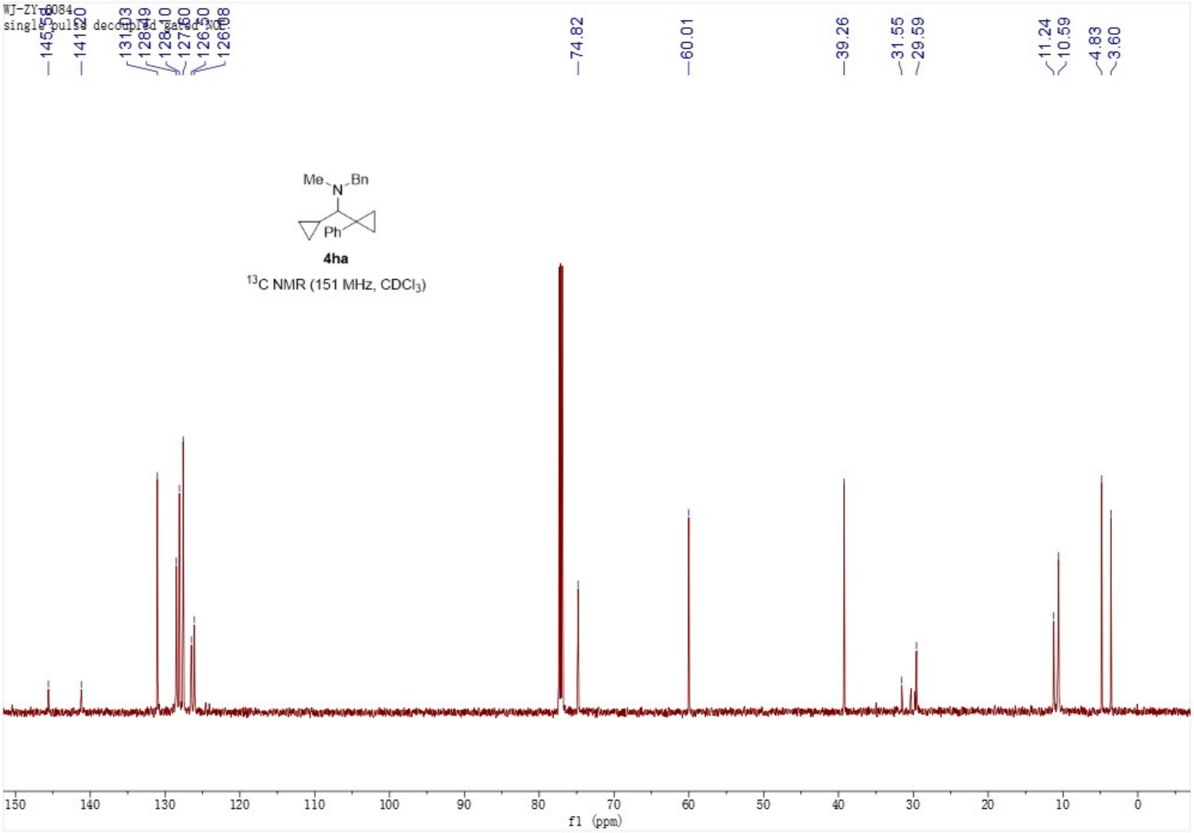
WJ-ZY-0084
single pulse decoupled gated NOE
Me
N
Bn
Ph
4ha
^{13}C NMR (151 MHz, CDCl$_3$)
f1 (ppm)

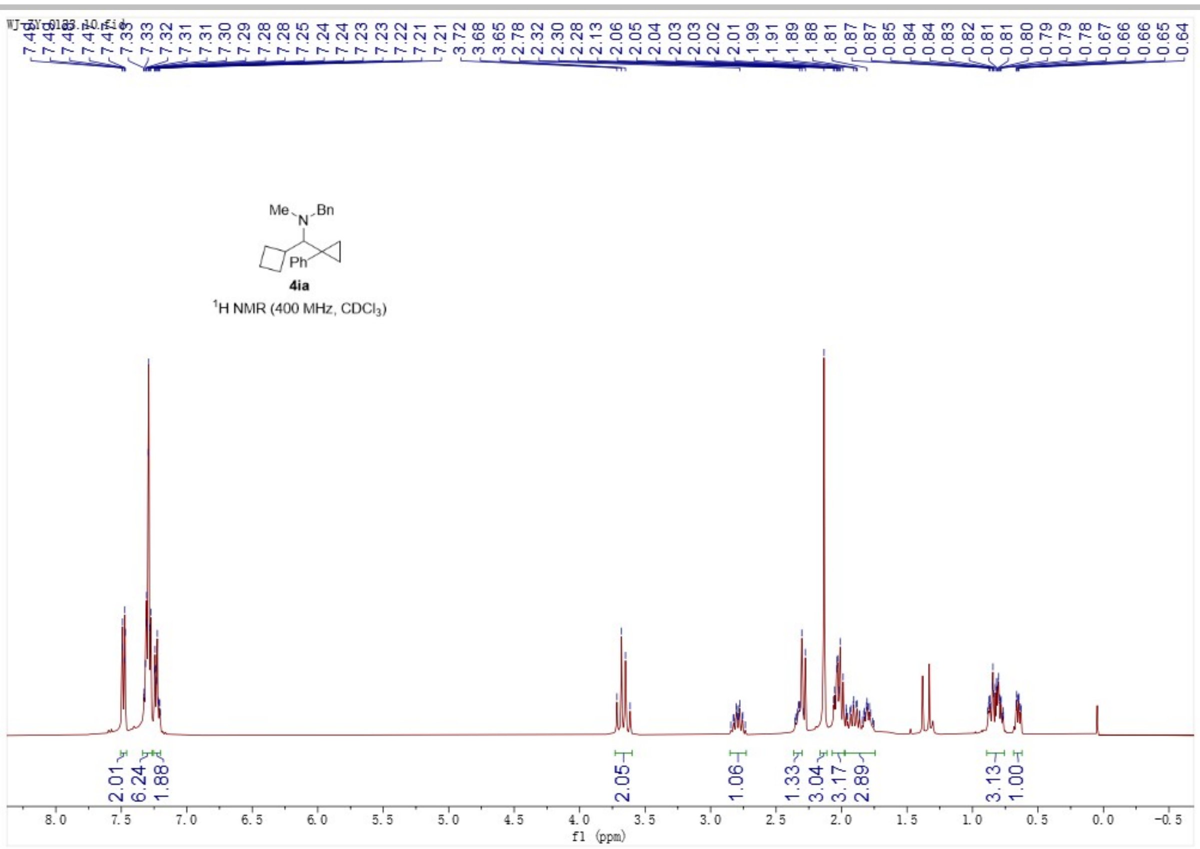

WJ-ZY-0123.10.fid
Me
N
Bn
Ph
4ia
1H NMR (400 MHz, CDCl3)
f1 (ppm)

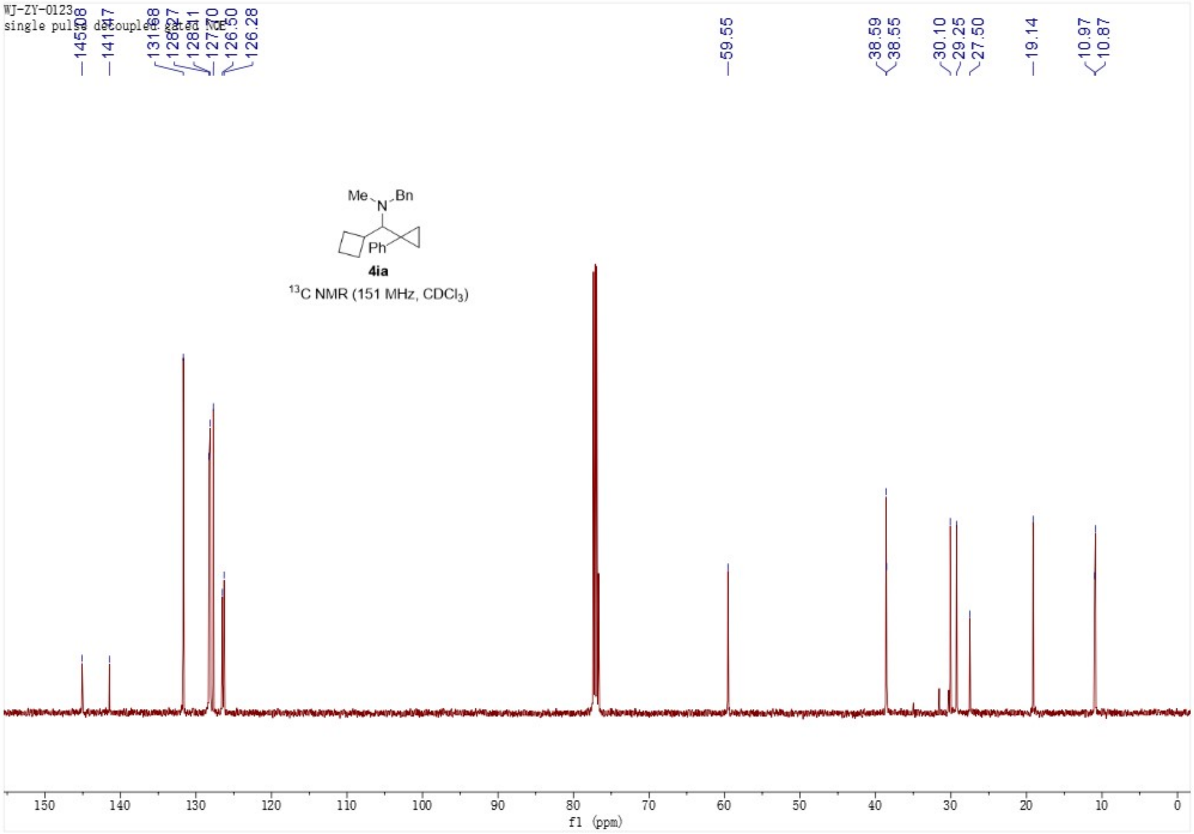

WJ-ZY-0123
single pulse decoupled gated NOE
Me
N
Bn
Ph
4ia
13C NMR (151 MHz, CDCl3)
f1 (ppm)

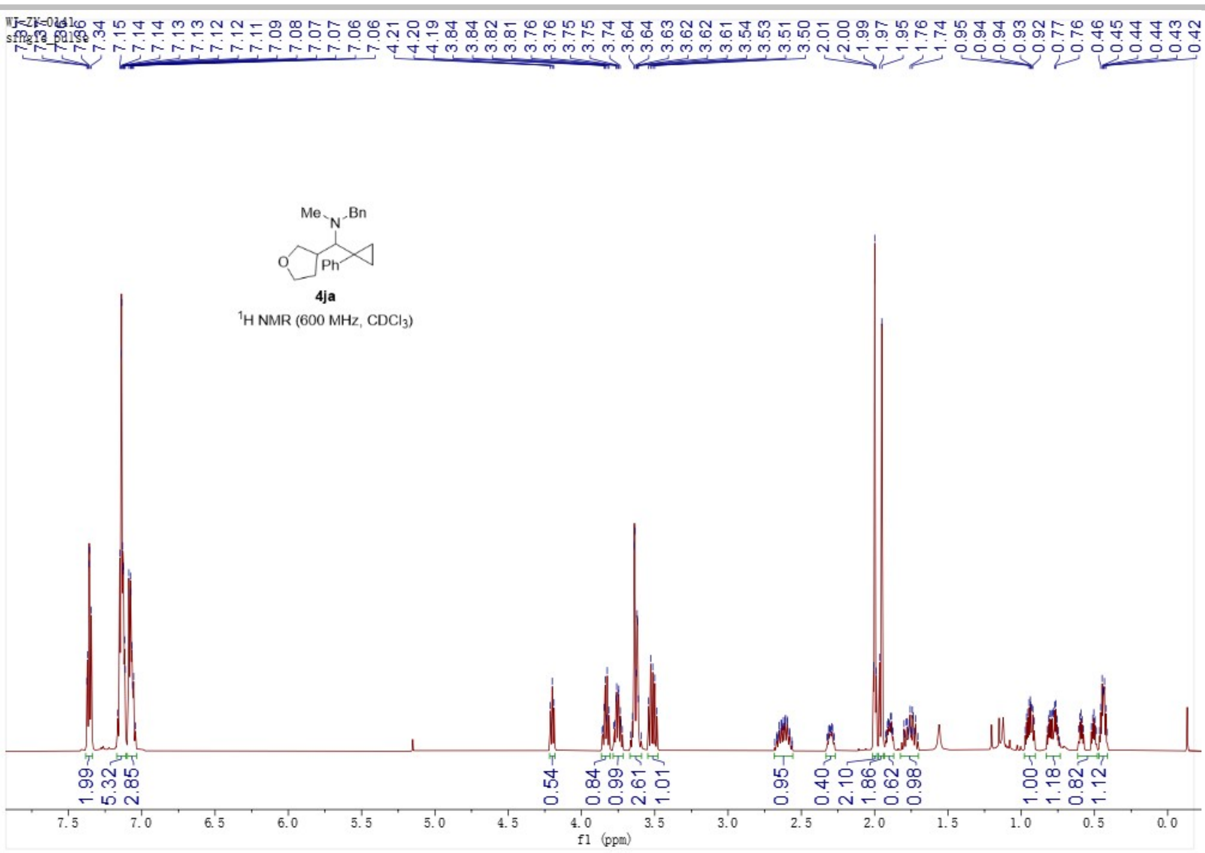
4ja
1H NMR (600 MHz, CDCl3)
f1 (ppm)

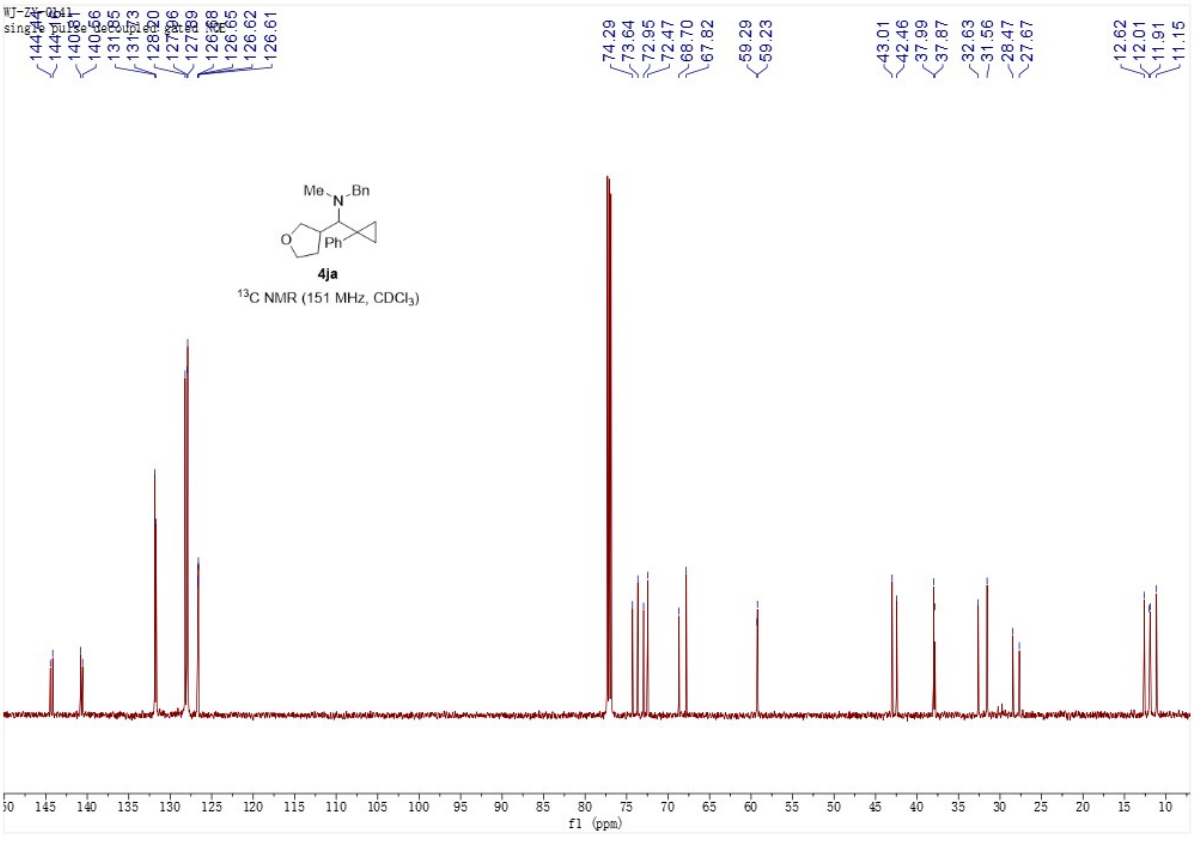
4ja
13C NMR (151 MHz, CDCl3)
f1 (ppm)

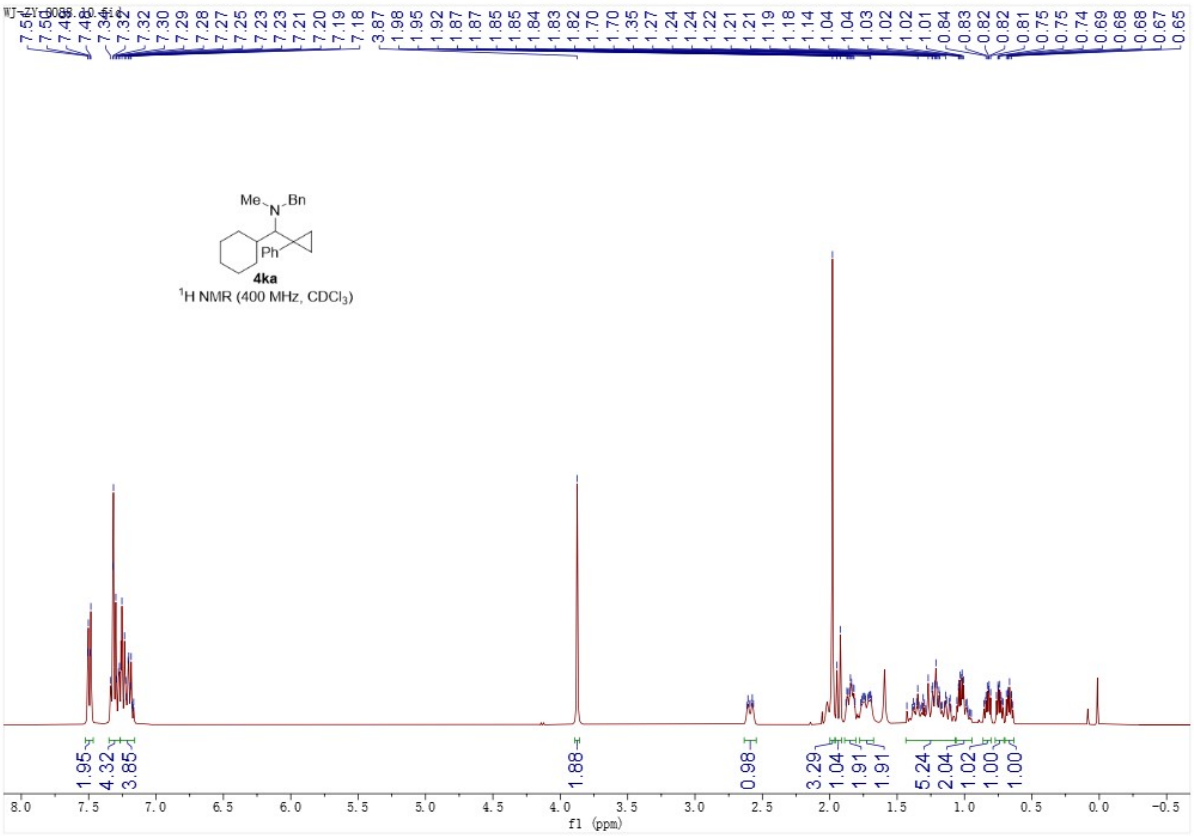
4ka
1H NMR (400 MHz, CDCl3)
f1 (ppm)

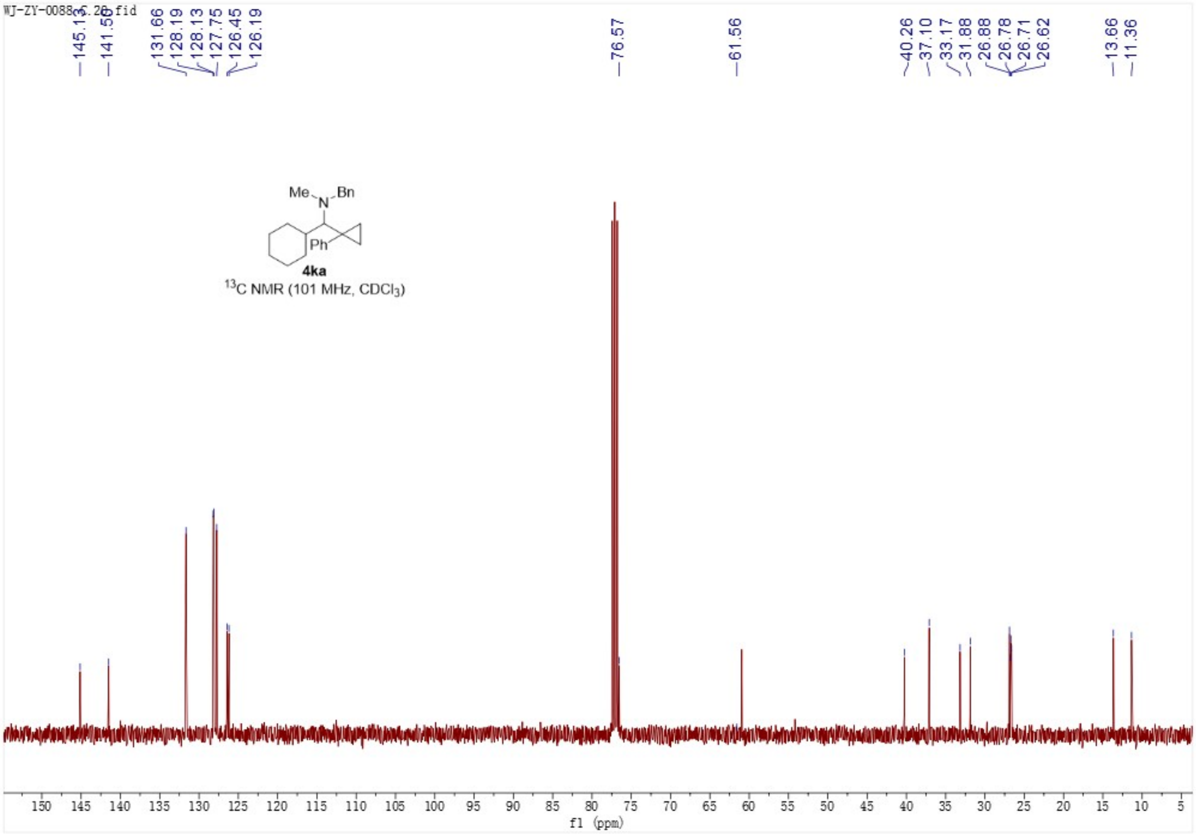
4ka
13C NMR (101 MHz, CDCl3)
f1 (ppm)

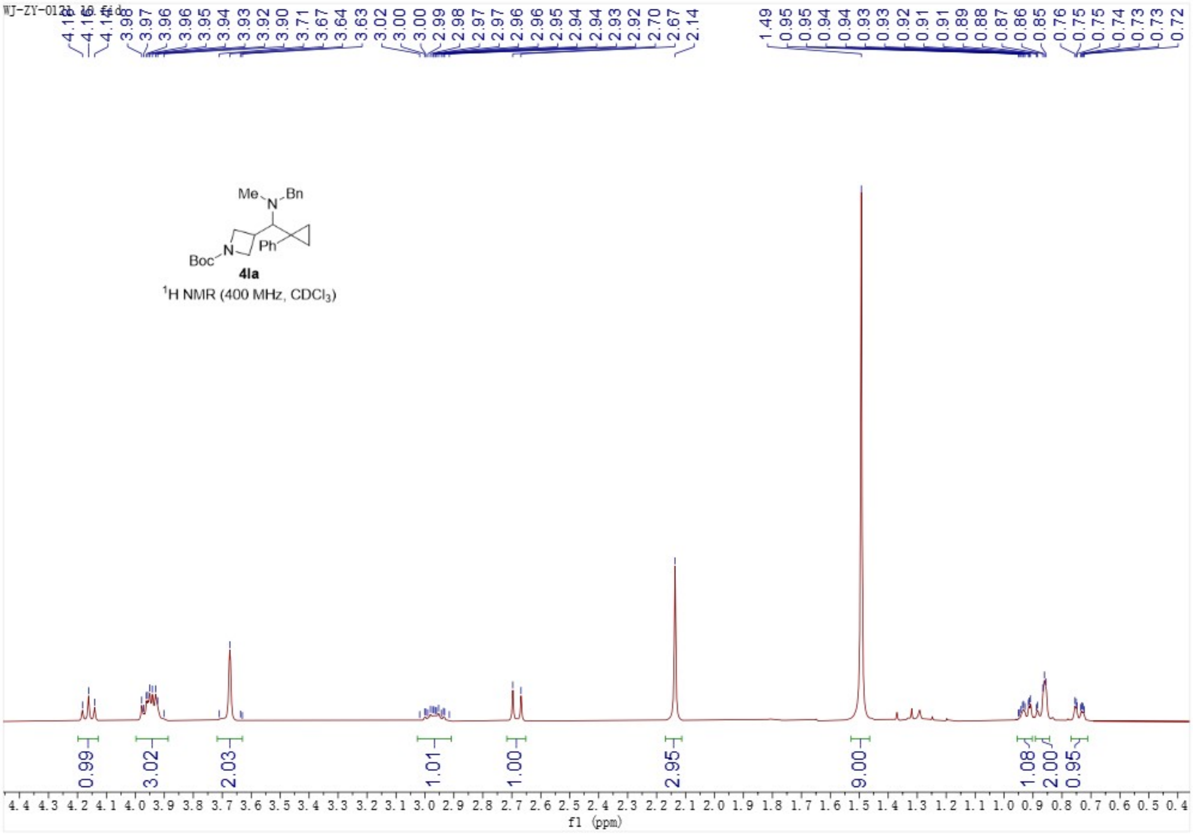

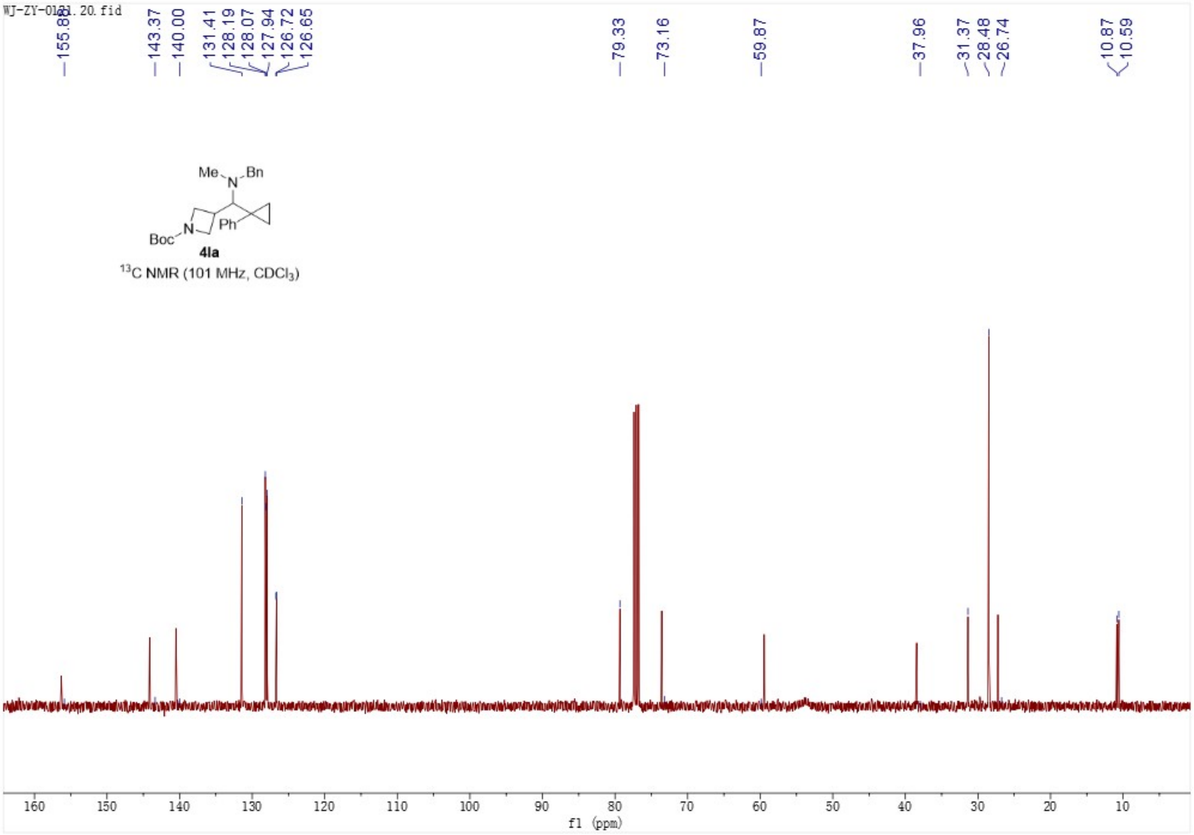

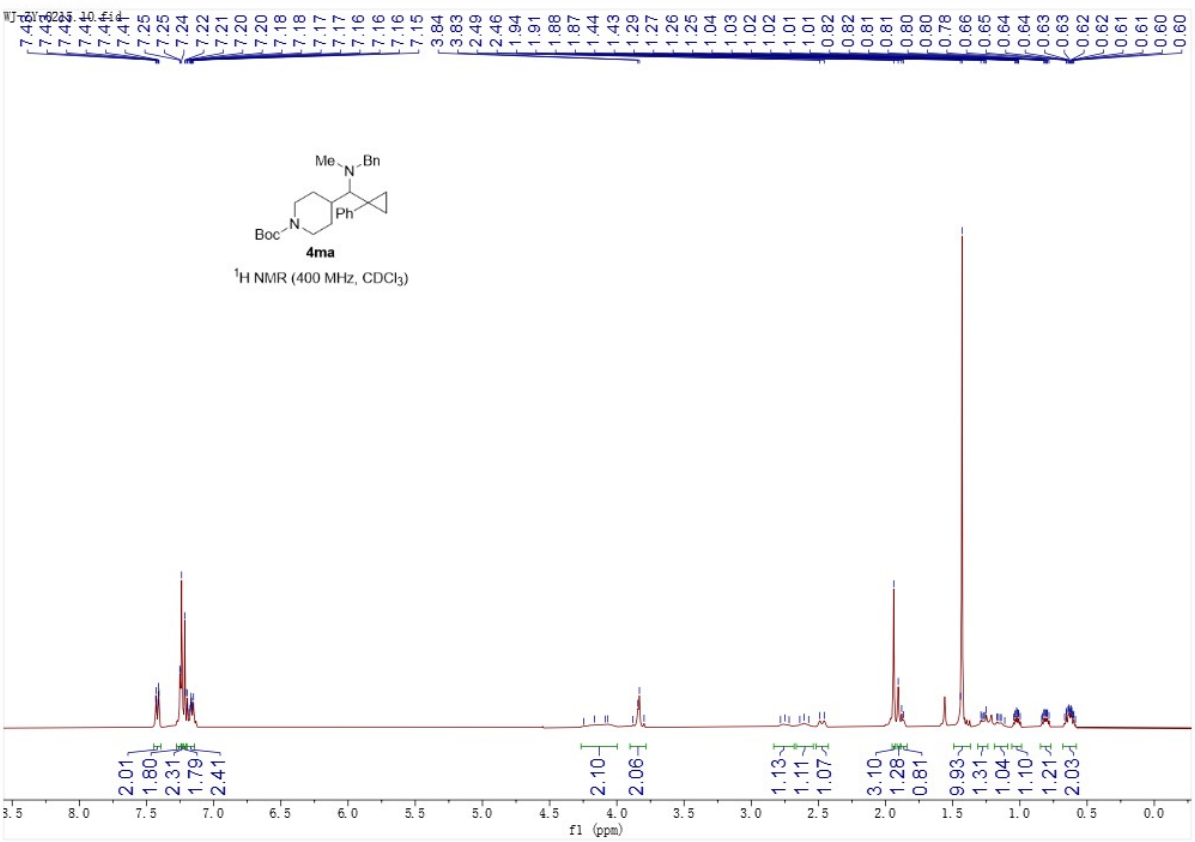
Me
N
Bn
Ph
Boc
N
4ma
^{1}H NMR (400 MHz, CDCl3)
f1 (ppm)

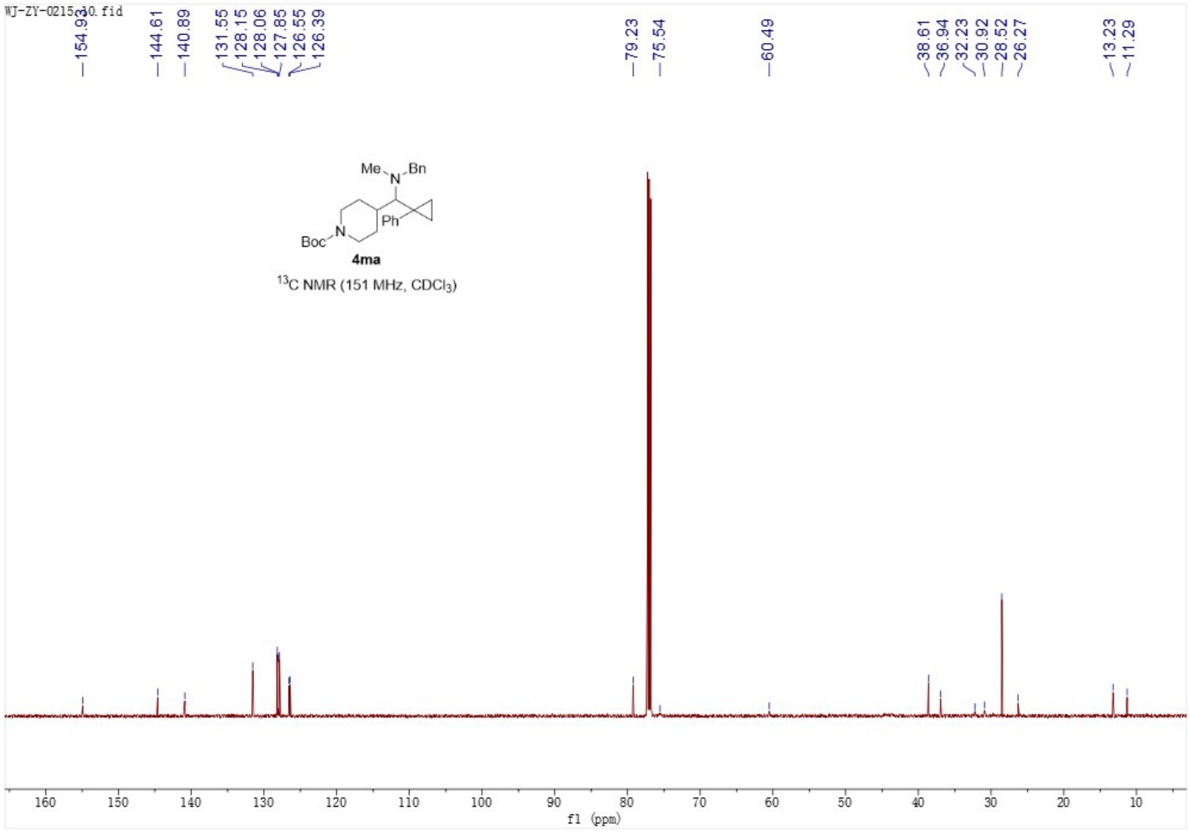
Me
N
Bn
Ph
Boc
N
4ma
^{13}C NMR (151 MHz, CDCl3)
154.9
144.61
140.89
131.55
128.15
128.06
127.85
126.55
126.39
79.23
75.54
60.49
38.61
36.94
32.23
30.92
28.52
26.27
13.23
11.29
f1 (ppm)

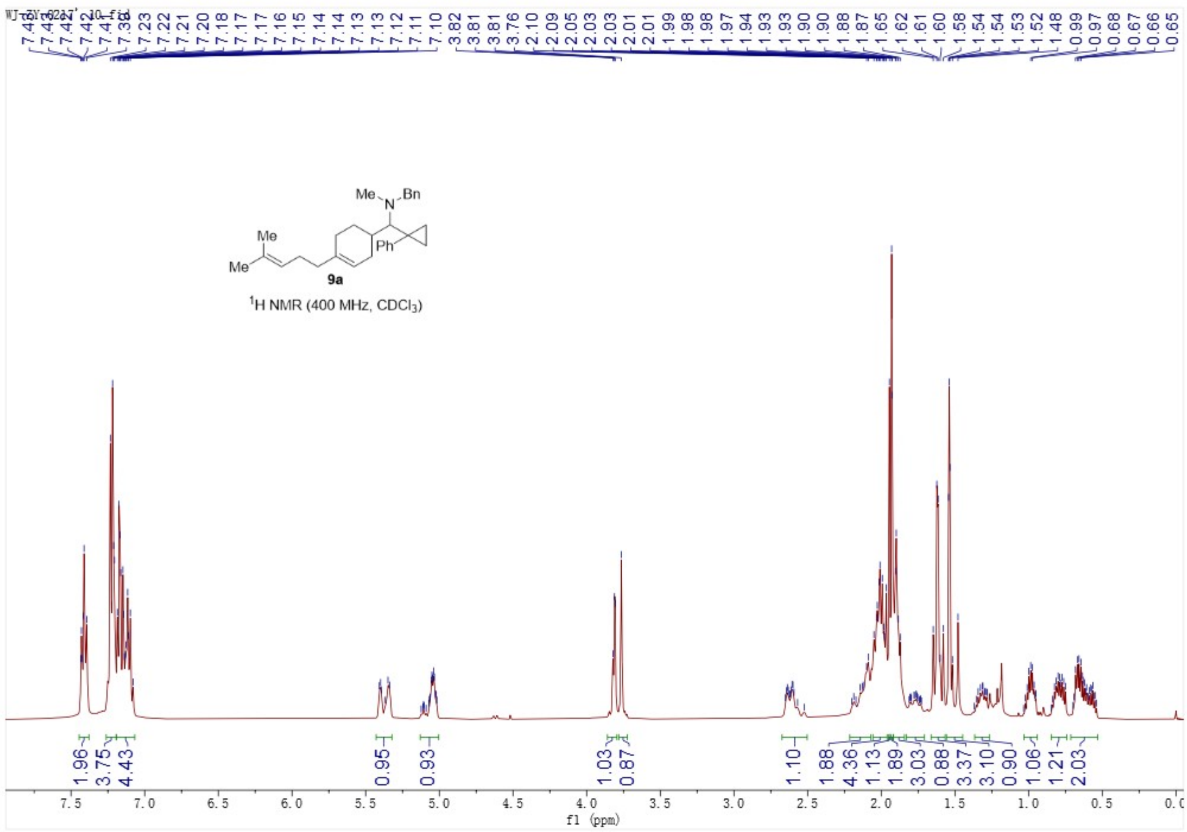

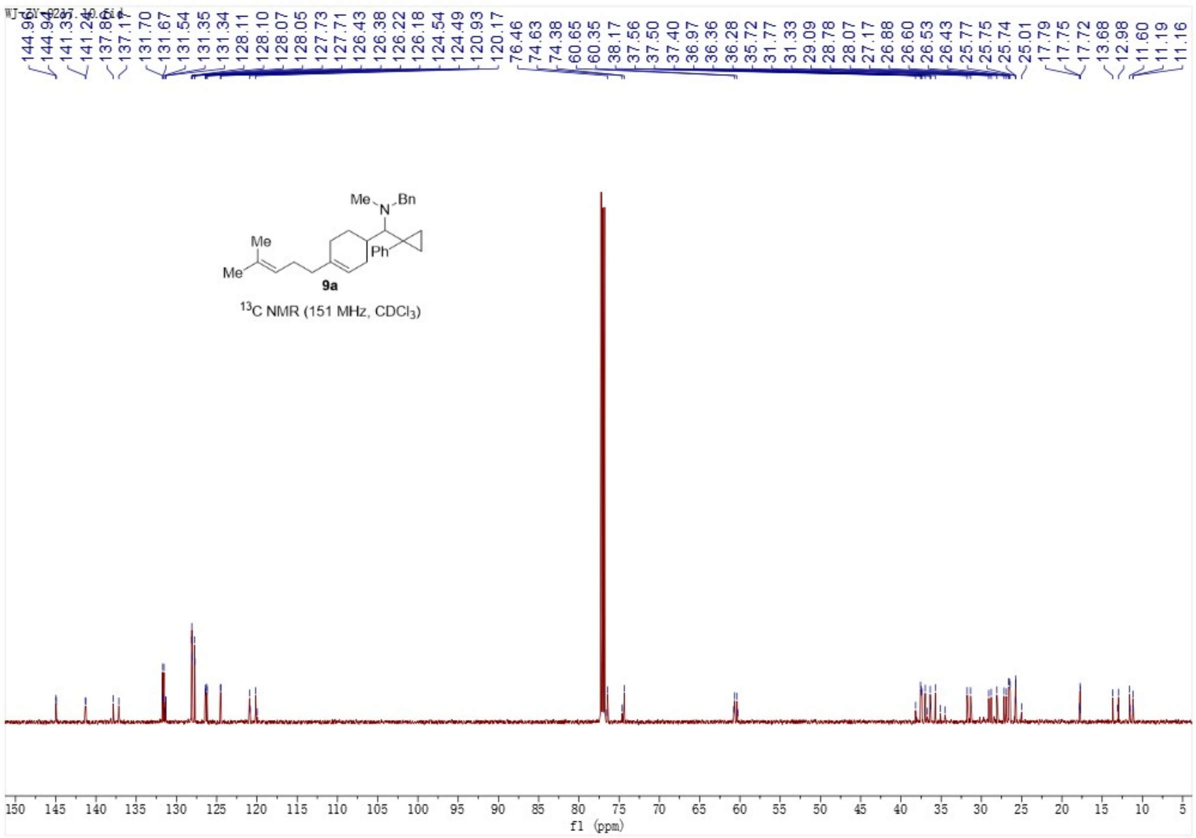

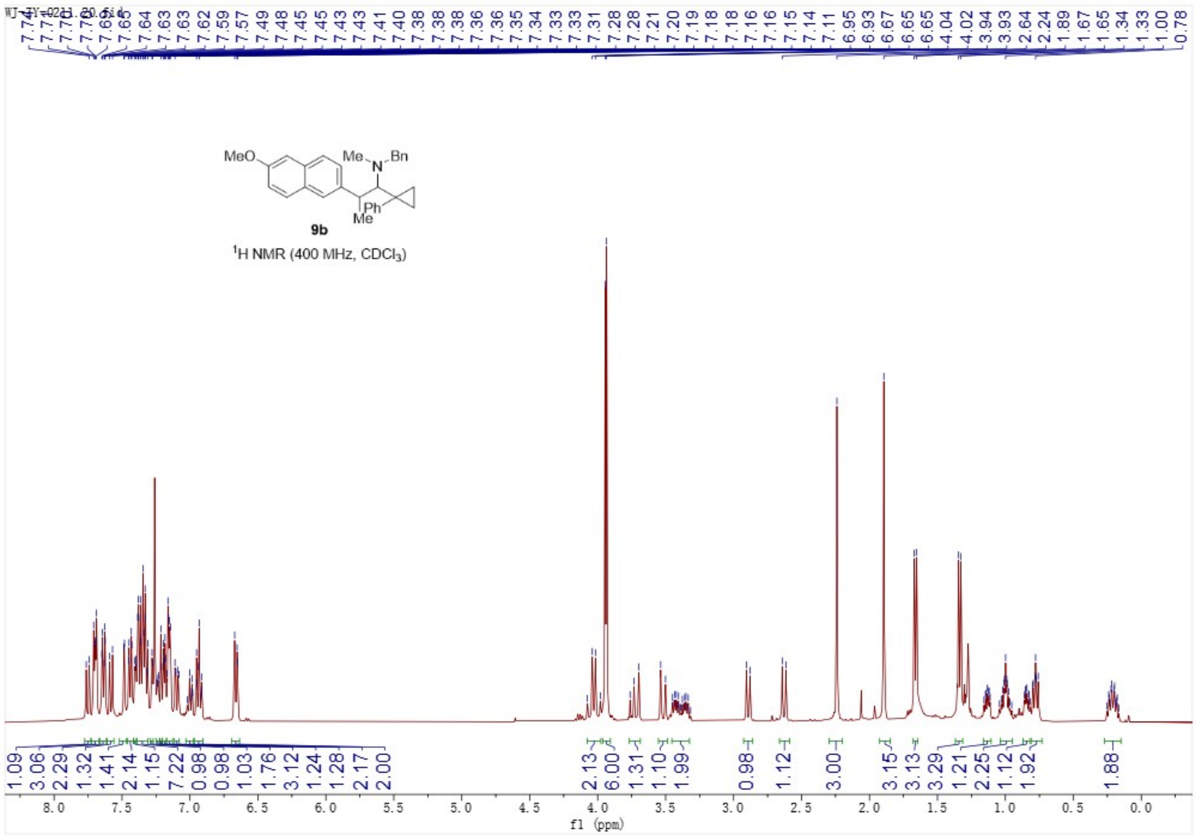

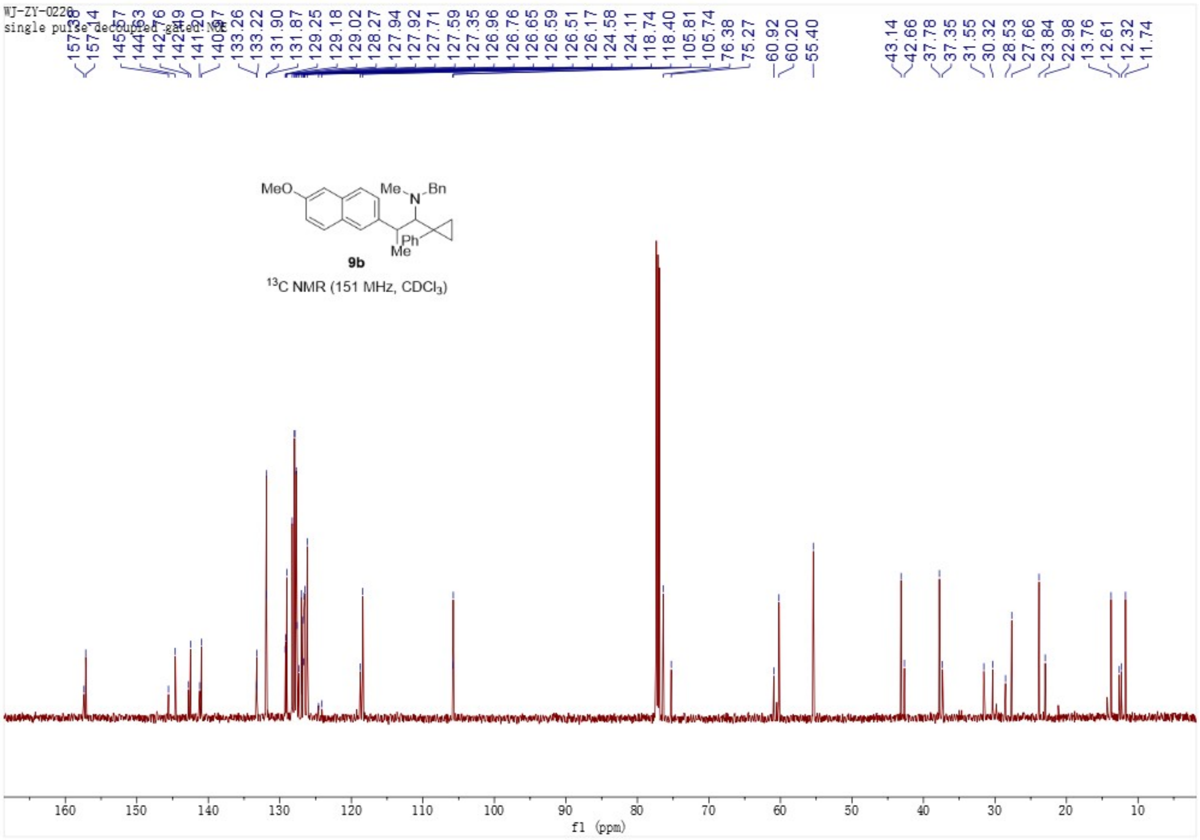

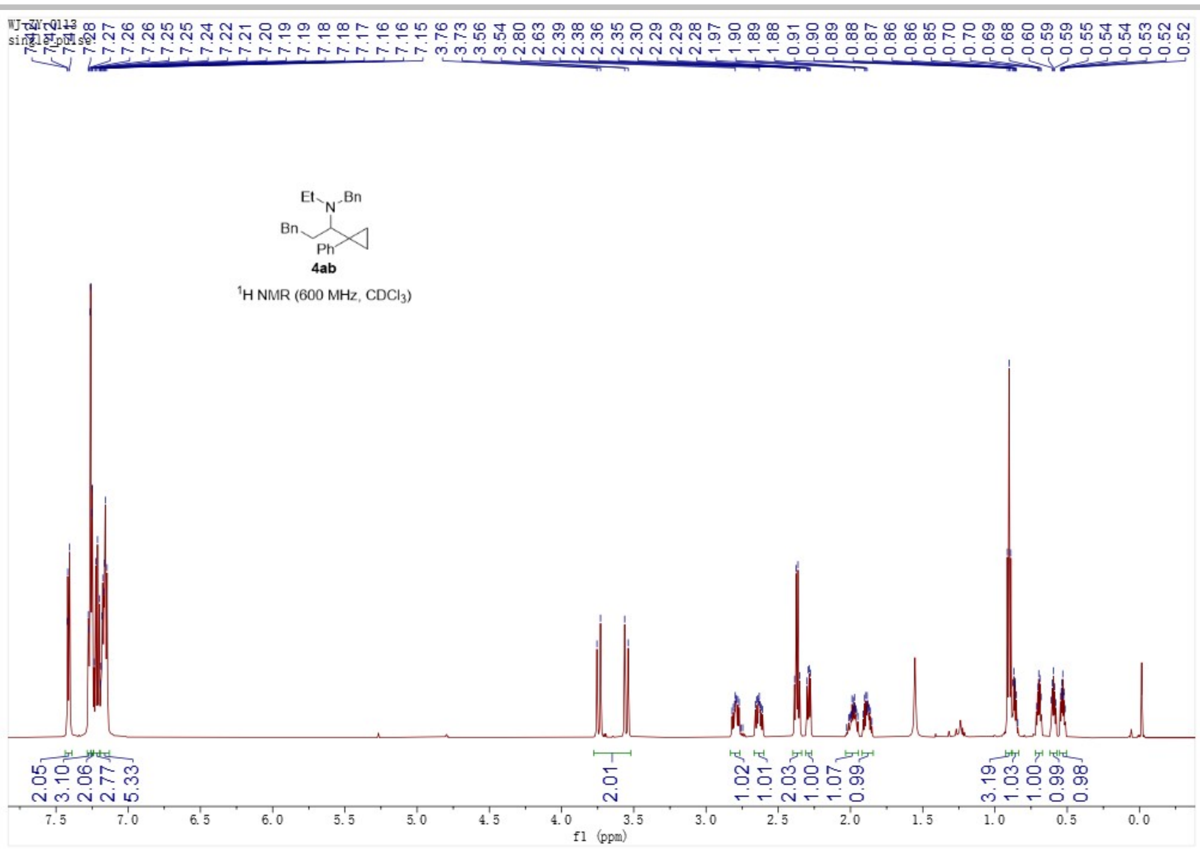

Et N Bn
Bn
Ph
4ab
^{1}H NMR (600 MHz, CDCl3)
f1 (ppm)

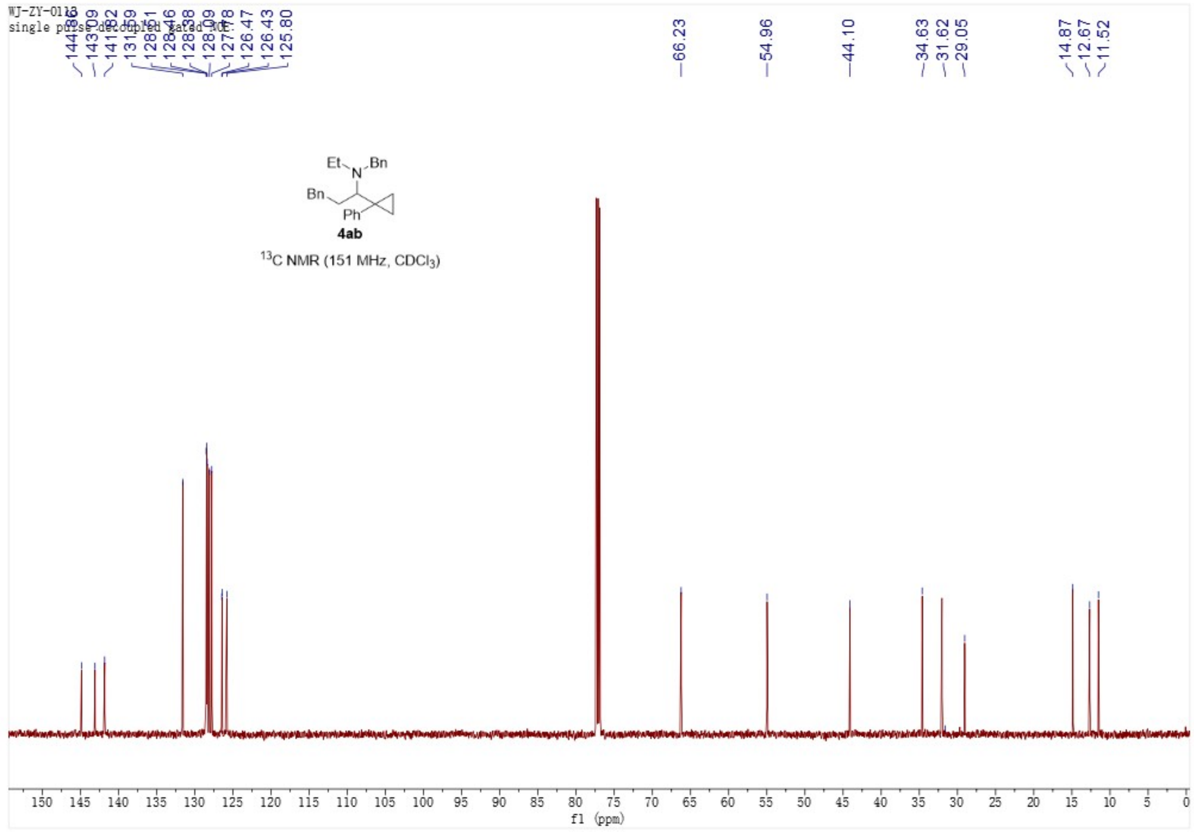

Et N Bn
Bn
Ph
4ab
^{13}C NMR (151 MHz, CDCl3)
f1 (ppm)

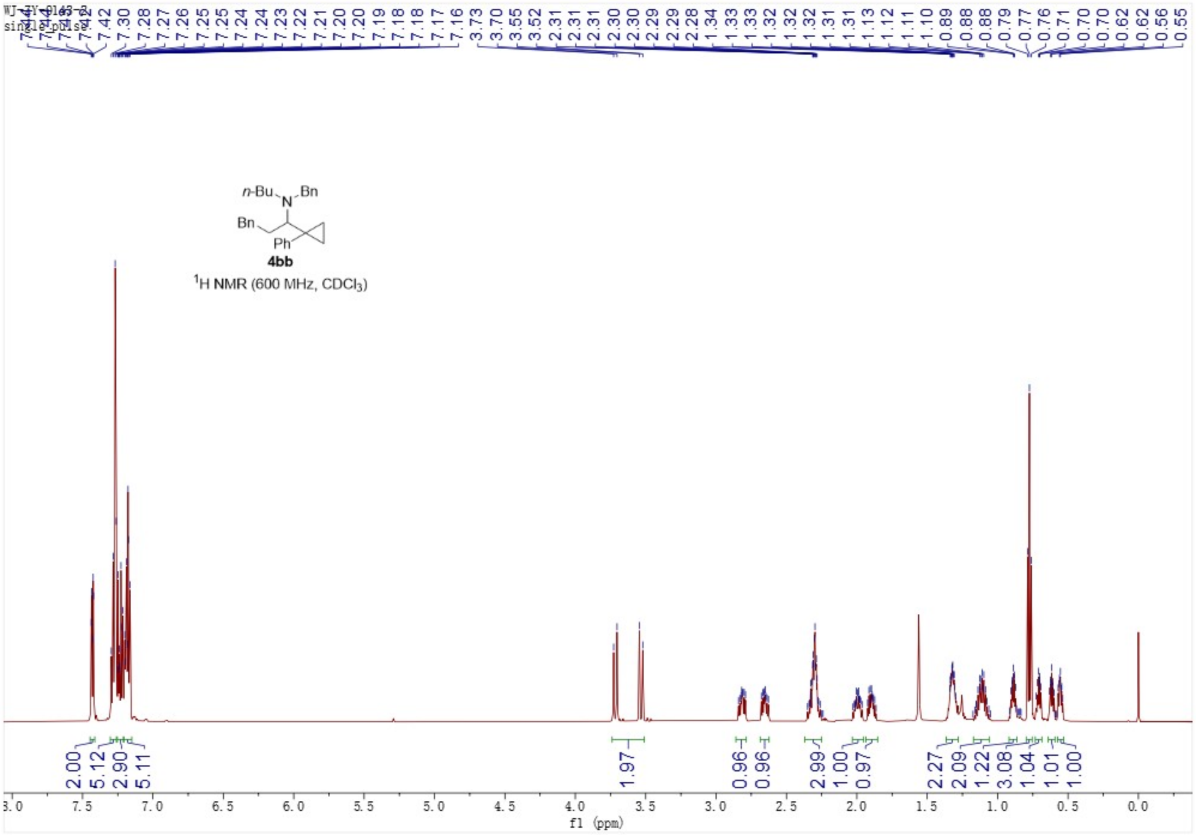

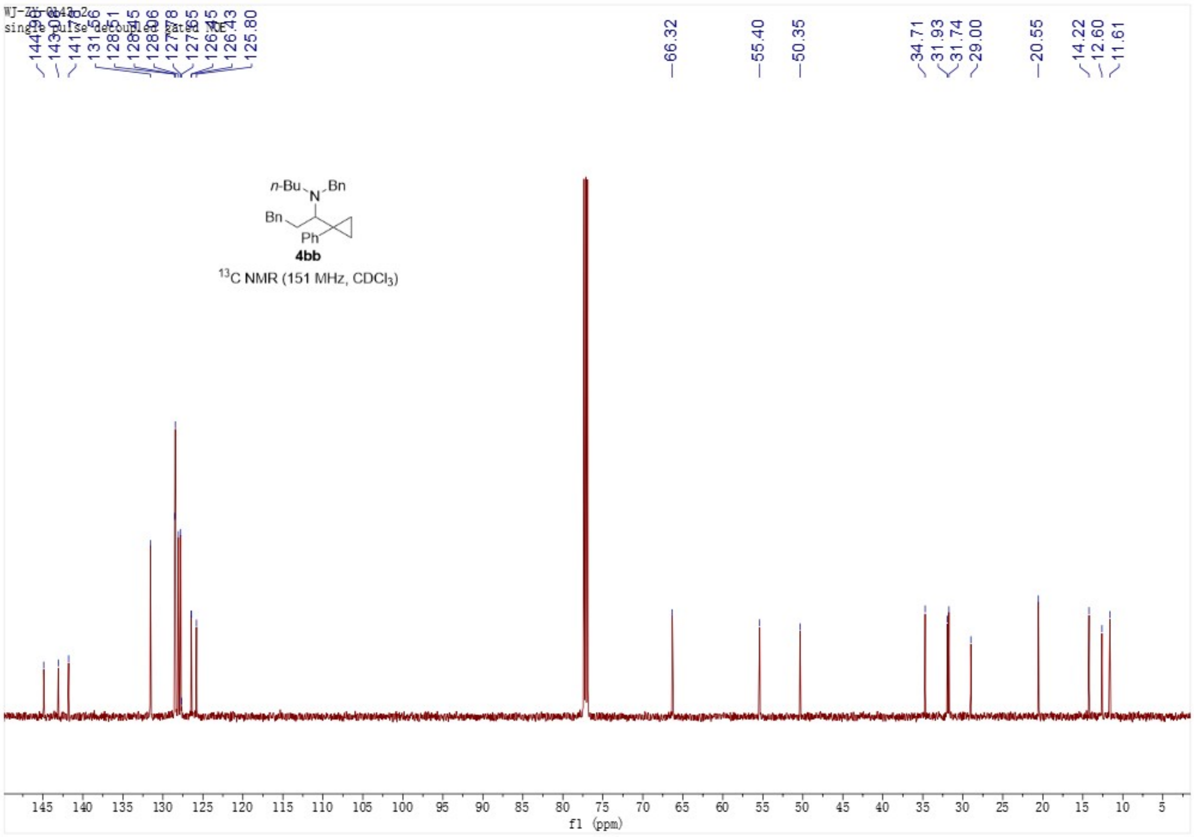

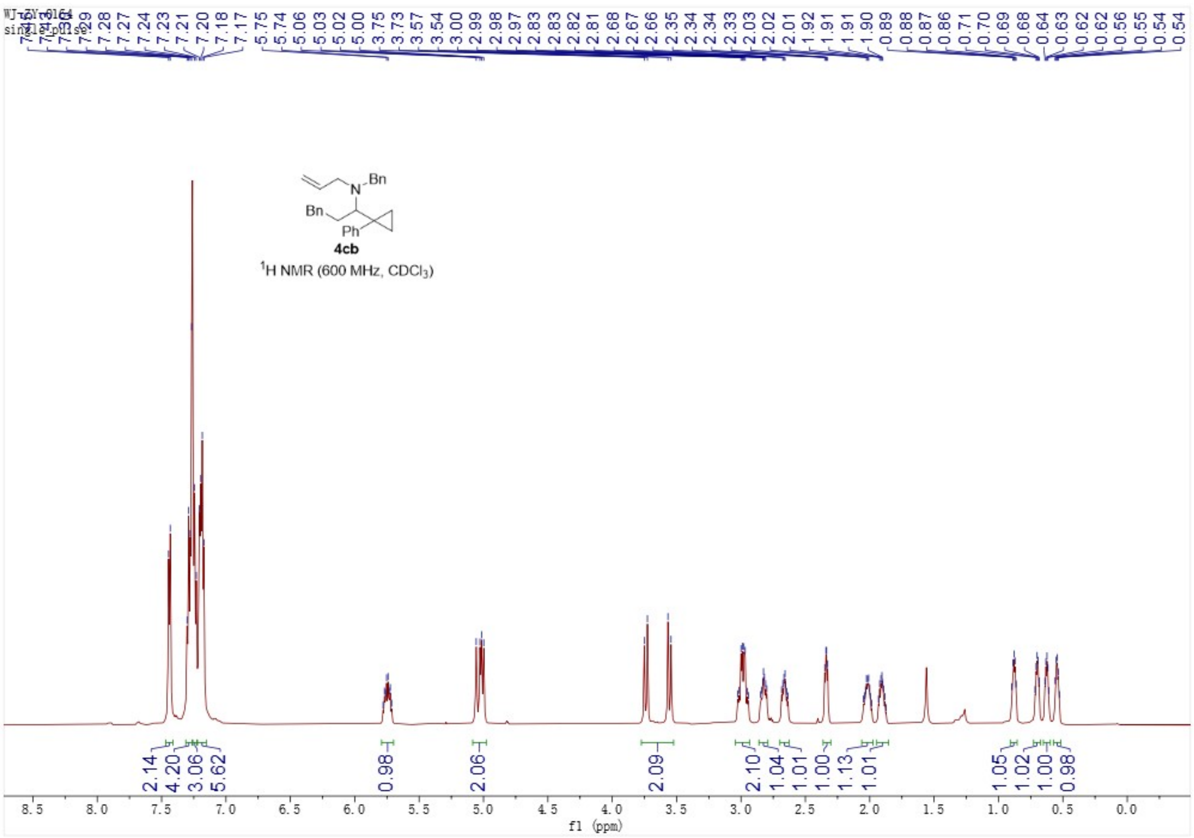

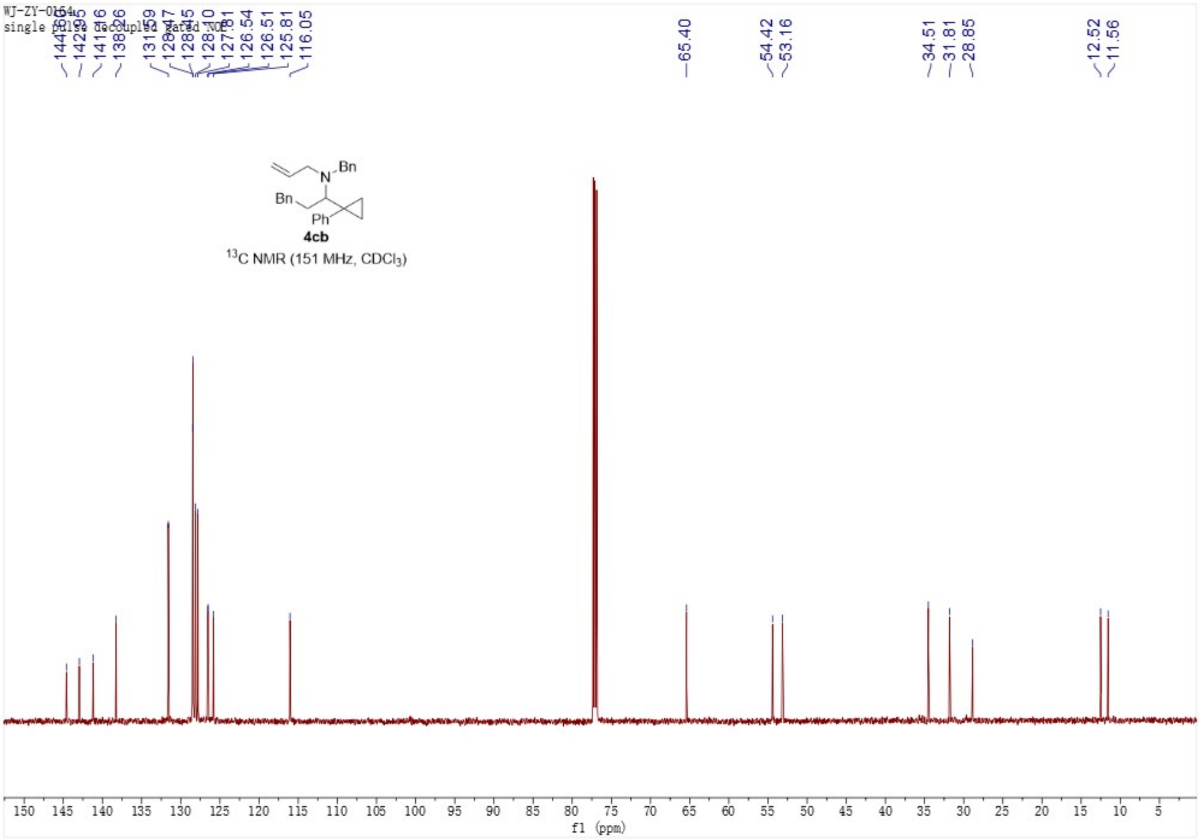

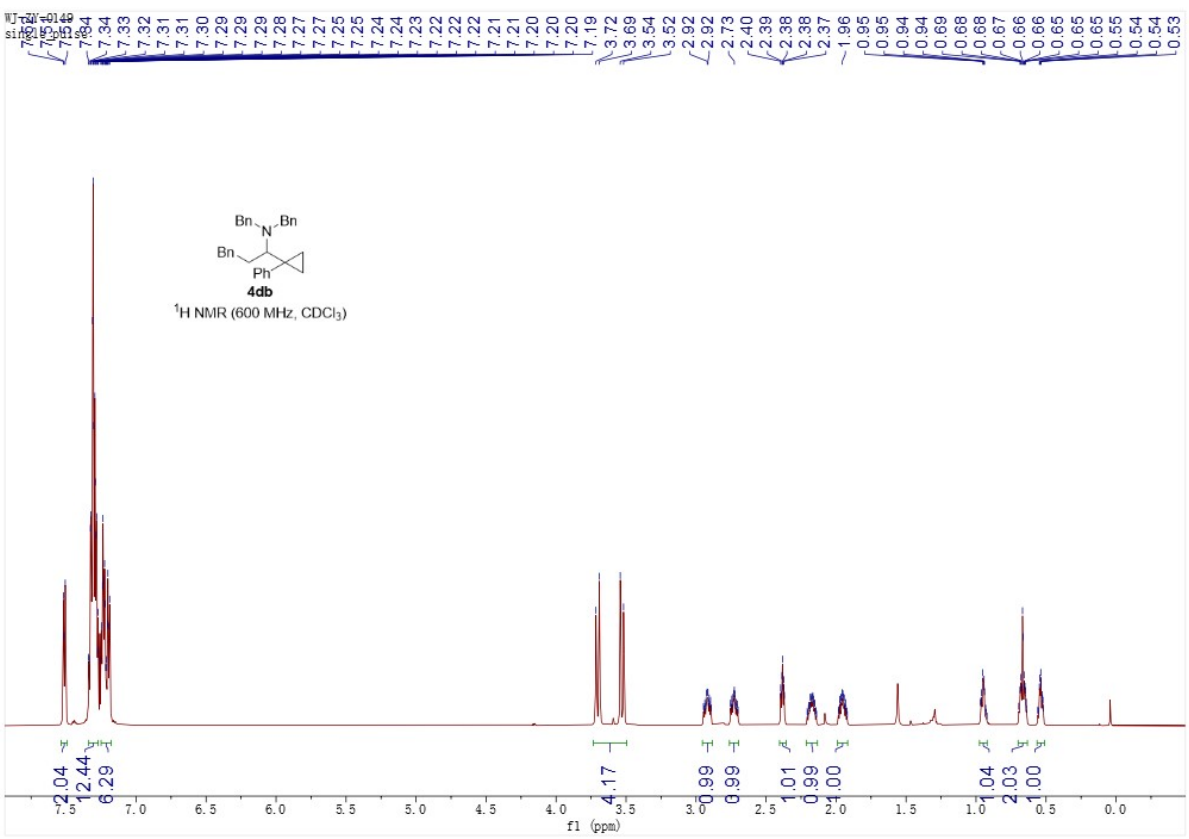
4db
^{1}H NMR (600 MHz, CDCl$_3$)

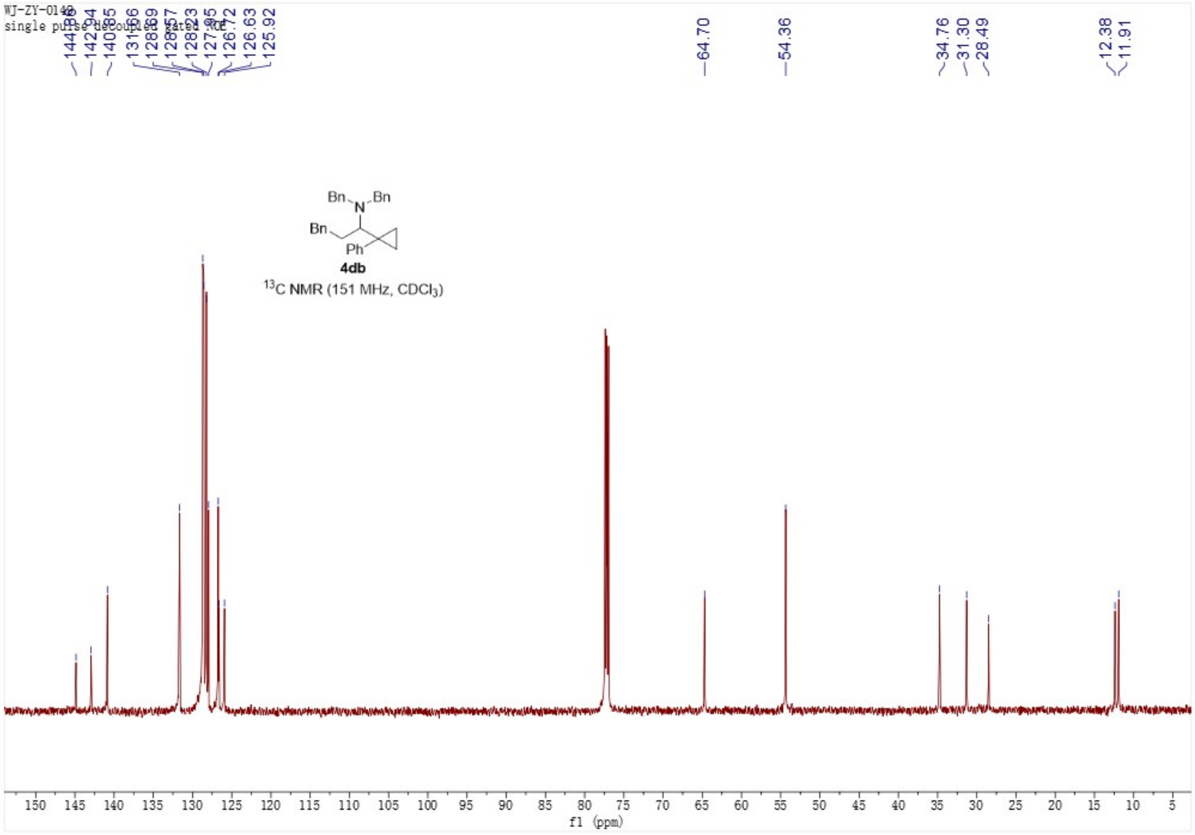
4db
^{13}C NMR (151 MHz, CDCl$_3$)

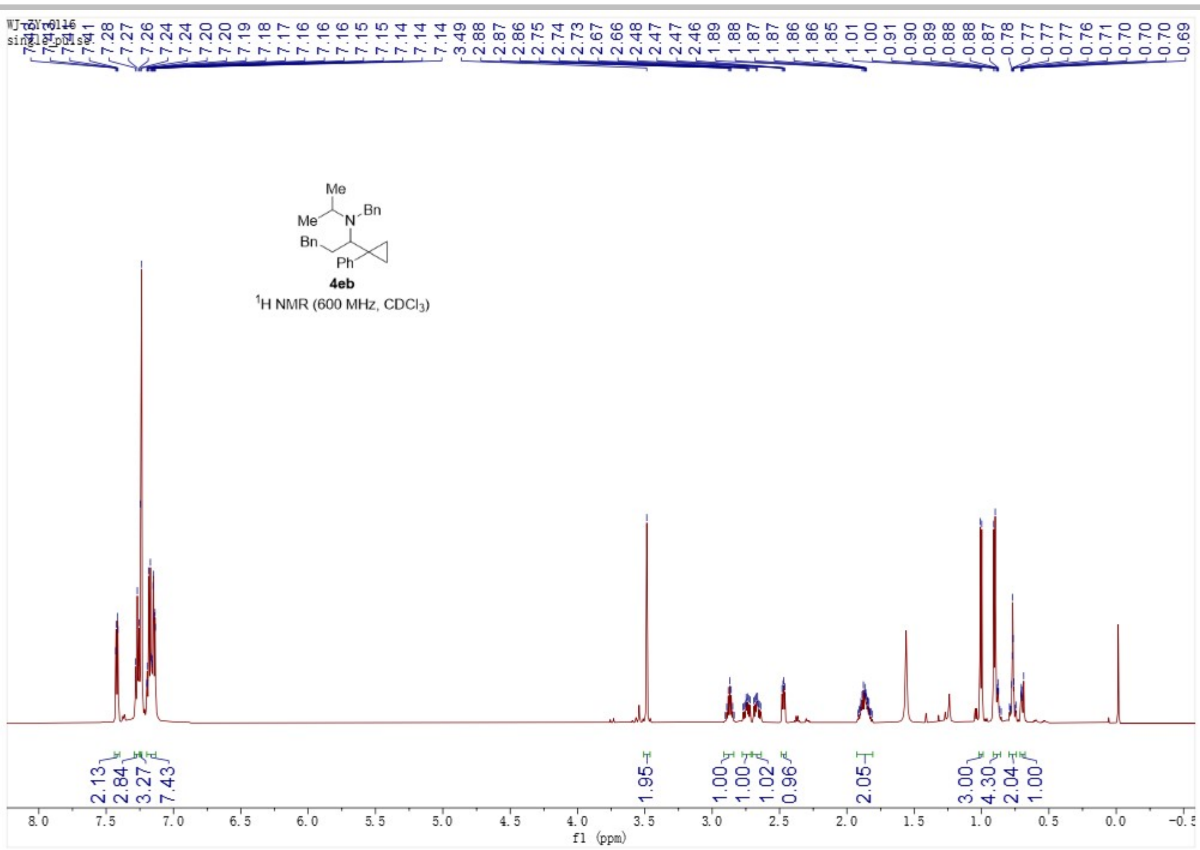
WJ-ZY-0116
single_pulse
7.28
7.27
7.26
7.24
7.24
7.20
7.20
7.19
7.18
7.17
7.16
7.16
7.16
7.15
7.15
7.14
7.14
7.14
3.49
2.88
2.87
2.86
2.75
2.74
2.73
2.67
2.66
2.48
2.47
2.47
2.46
1.89
1.88
1.87
1.87
1.86
1.86
1.85
1.01
1.00
0.91
0.90
0.89
0.88
0.88
0.87
0.78
0.77
0.77
0.77
0.76
0.71
0.70
0.70
0.70
0.69
Me
Me
N
Bn
Bn
Ph
4eb
^{1}H NMR (600 MHz, CDCl$_3$)
2.13
2.84
3.27
7.43
1.95
1.00
1.00
1.02
0.96
2.05
3.00
4.30
2.04
1.00
f1 (ppm)

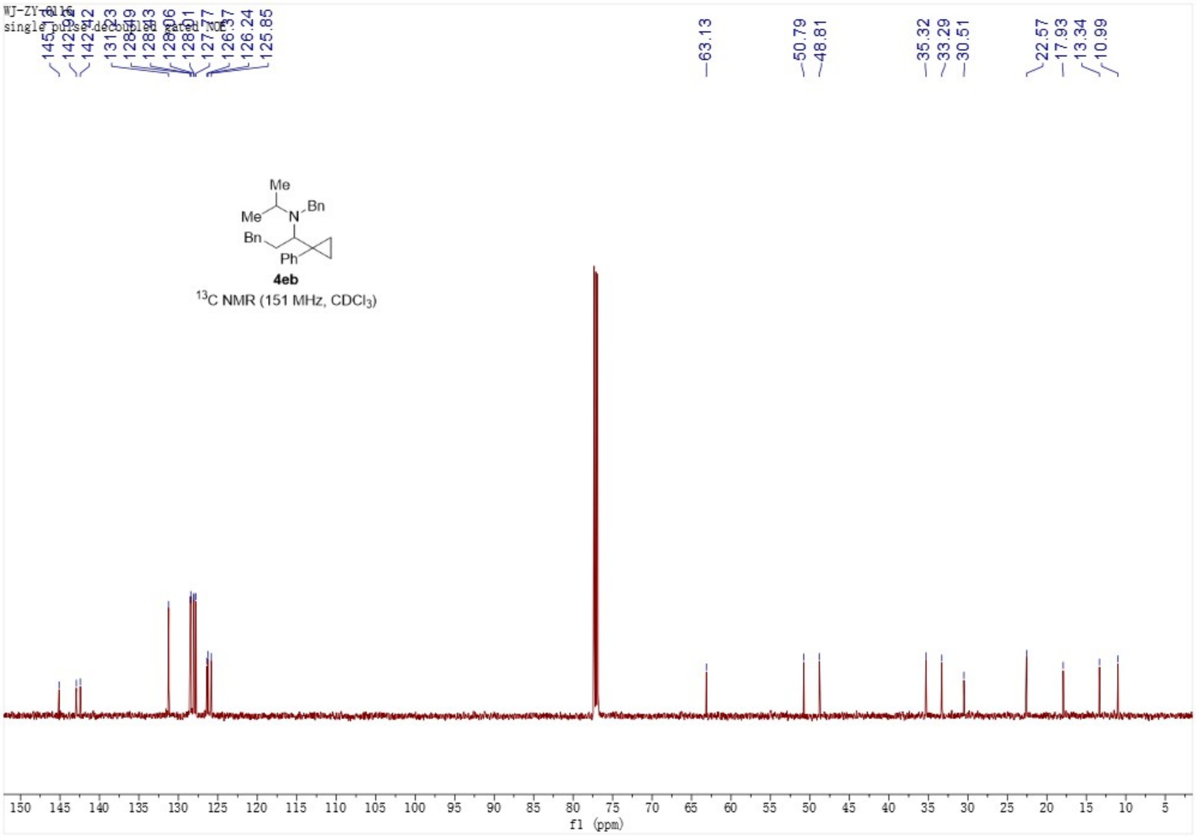
WJ-ZY-0116
single pulse decoupled gated NOE
131.23
128.13
127.77
126.37
126.24
125.85
63.13
50.79
48.81
35.32
33.29
30.51
22.57
17.93
13.34
10.99
Me
Me
N
Bn
Bn
Ph
4eb
^{13}C NMR (151 MHz, CDCl$_3$)
f1 (ppm)

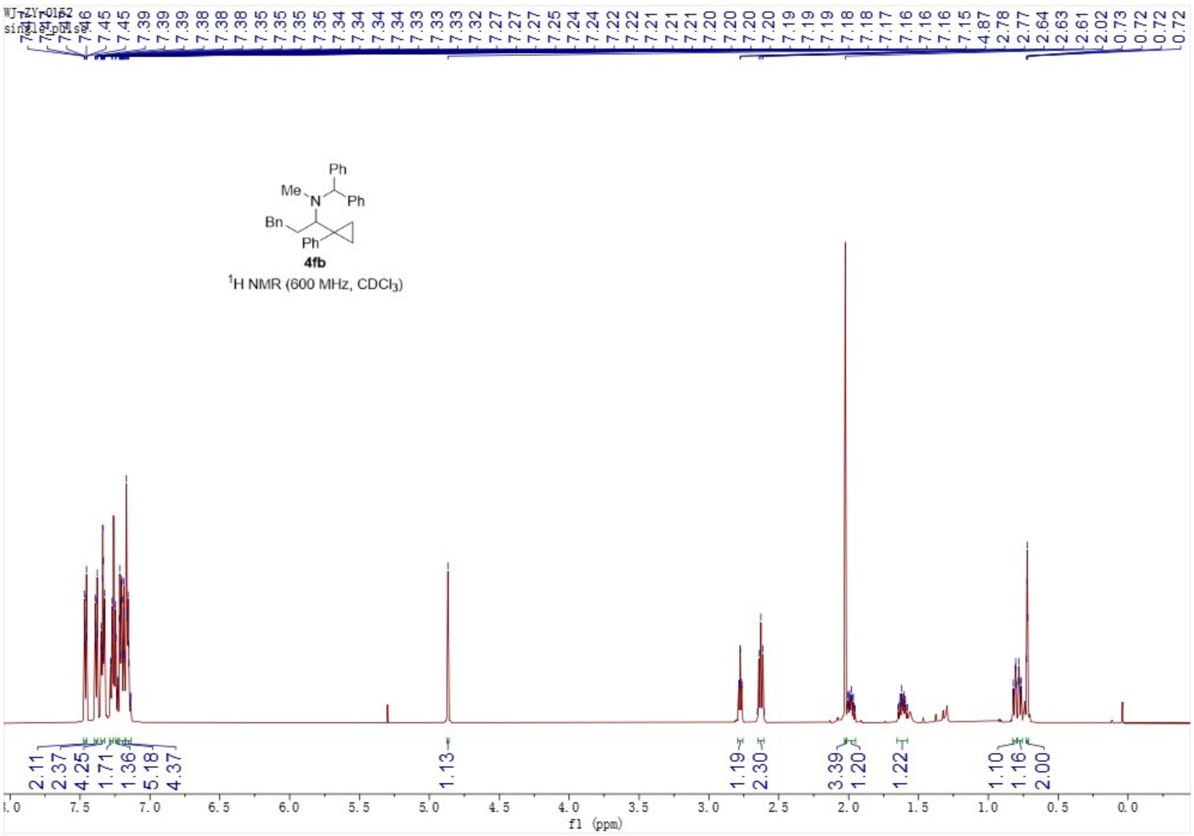
Me
N
Ph
Ph
Bn
Ph
4fb
^{1}H NMR (600 MHz, CDCl$_3$)
f1 (ppm)

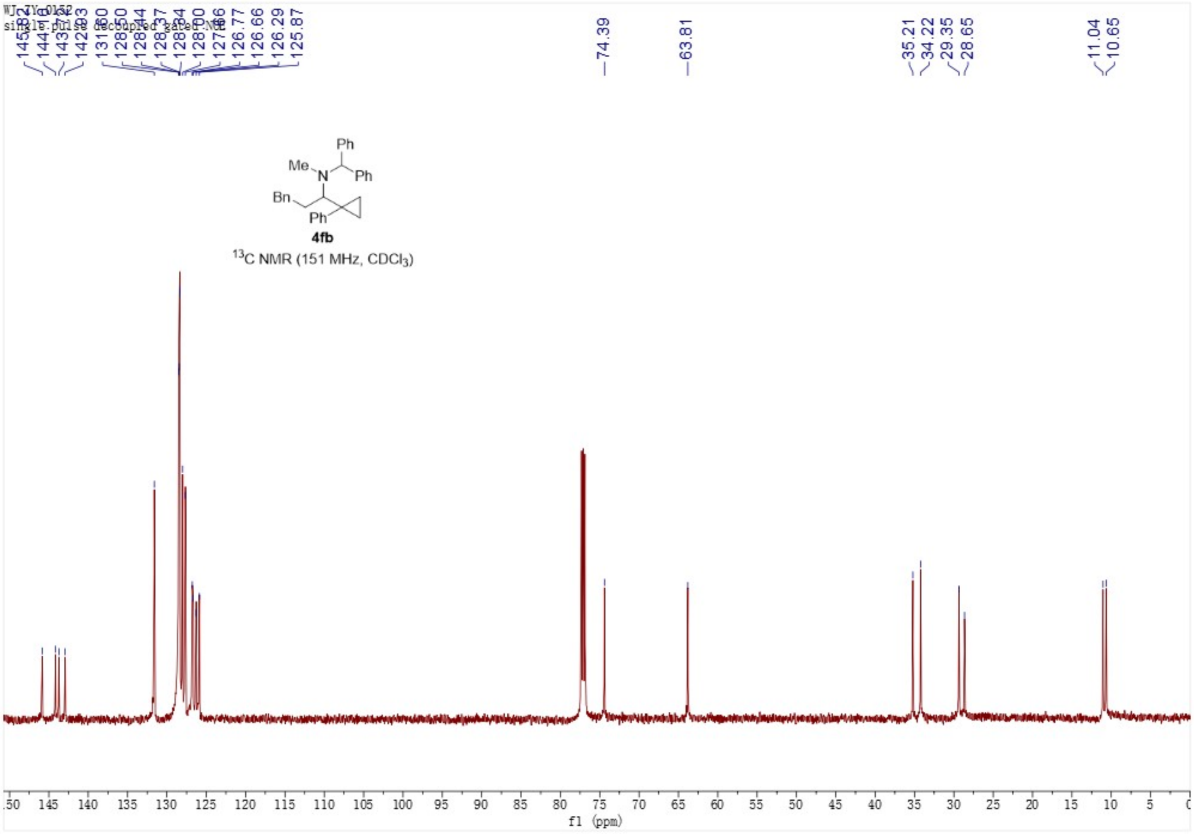
74.39
63.81
35.21
34.22
29.35
28.65
11.04
10.65
Me
N
Ph
Ph
Bn
Ph
4fb
^{13}C NMR (151 MHz, CDCl$_3$)
f1 (ppm)

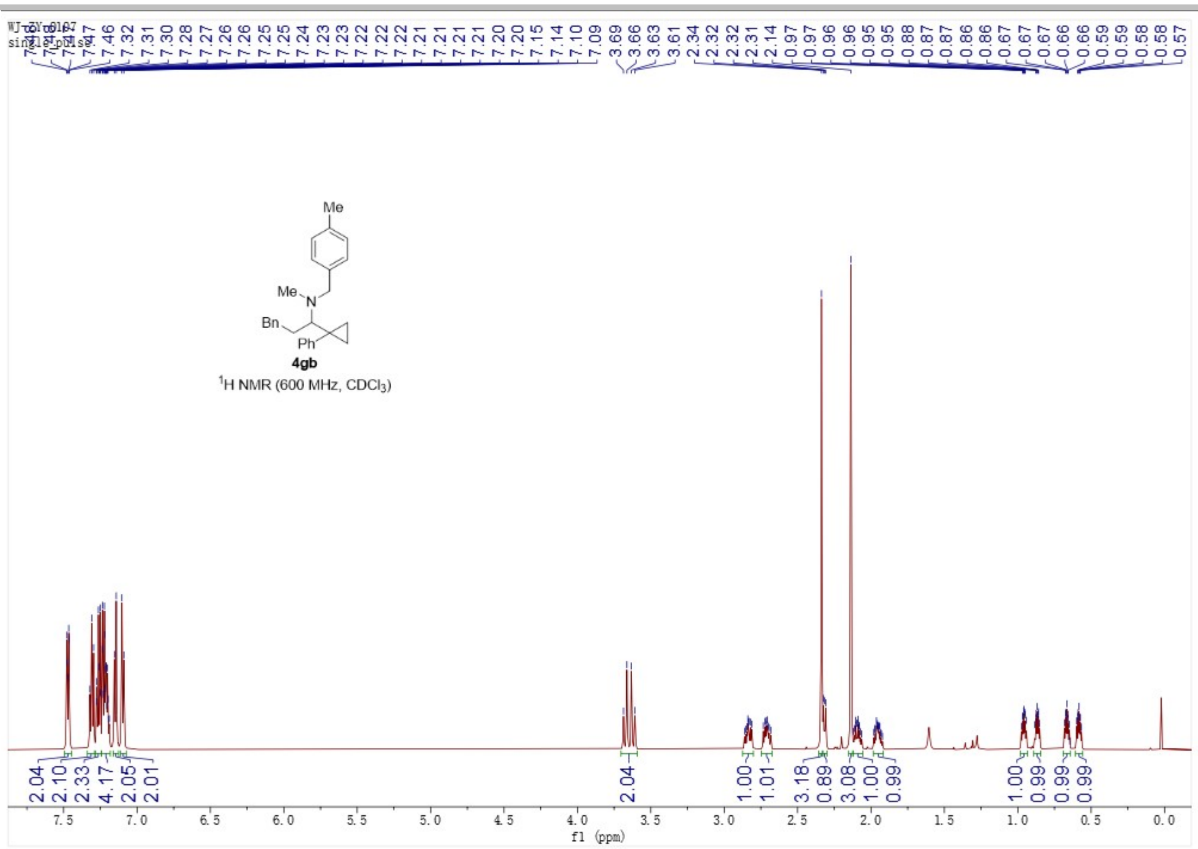
4gb
^{1}H NMR (600 MHz, CDCl$_3$)

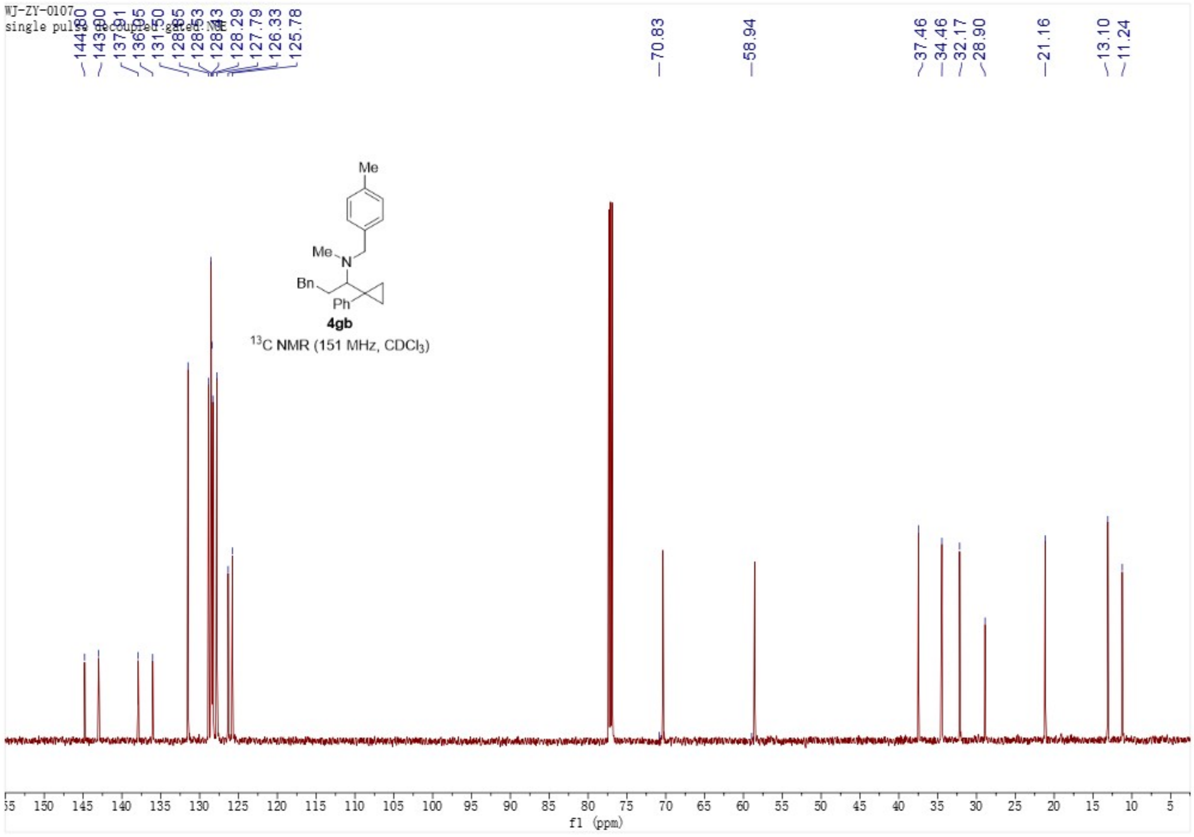
4gb
^{13}C NMR (151 MHz, CDCl$_3$)

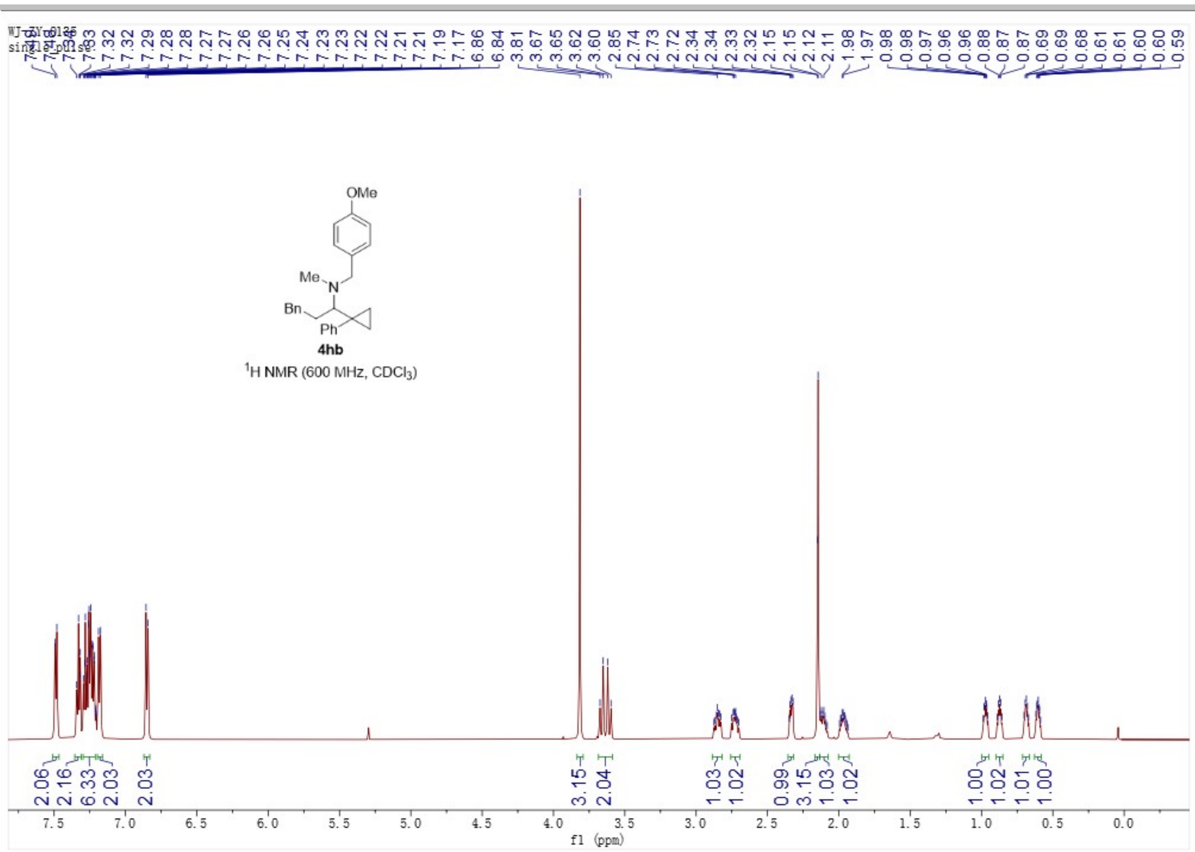
OMe
Me
N
Bn
Ph
4hb
^{1}H NMR (600 MHz, CDCl$_3$)
f1 (ppm)

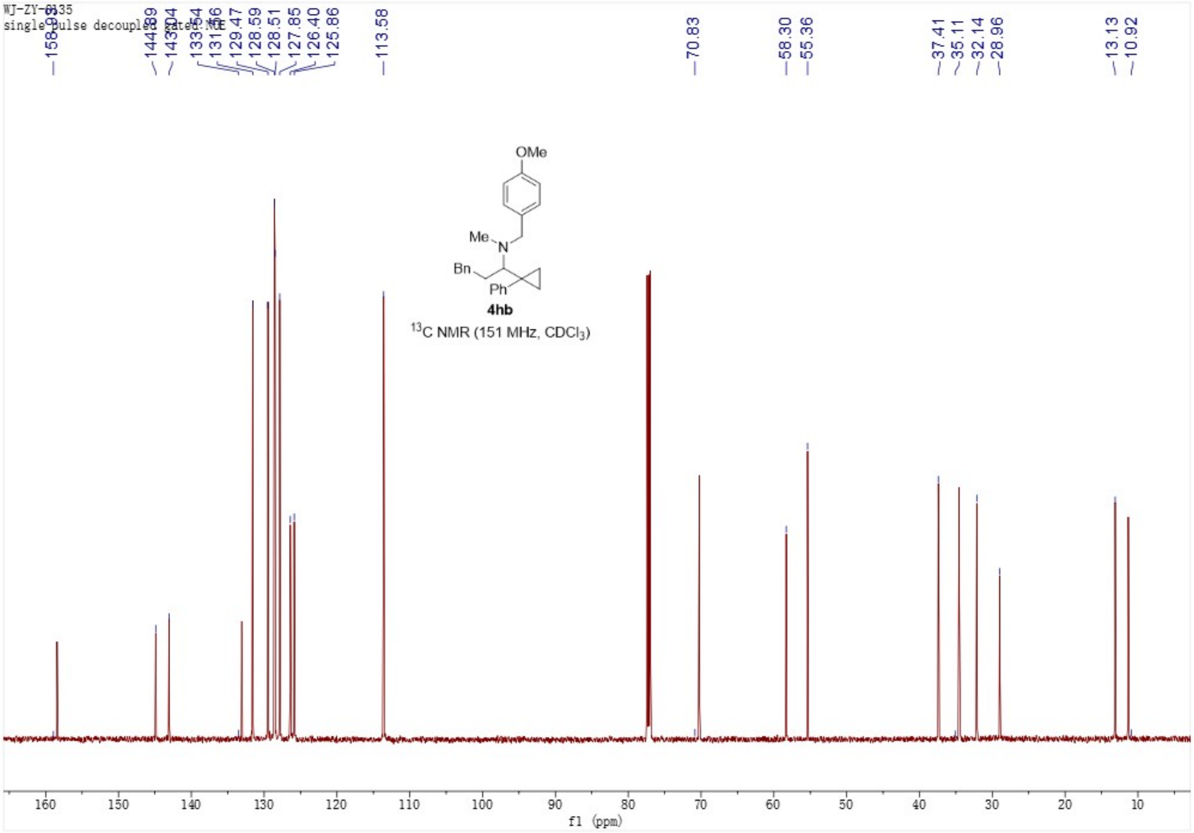
OMe
Me
N
Bn
Ph
4hb
^{13}C NMR (151 MHz, CDCl$_3$)
f1 (ppm)

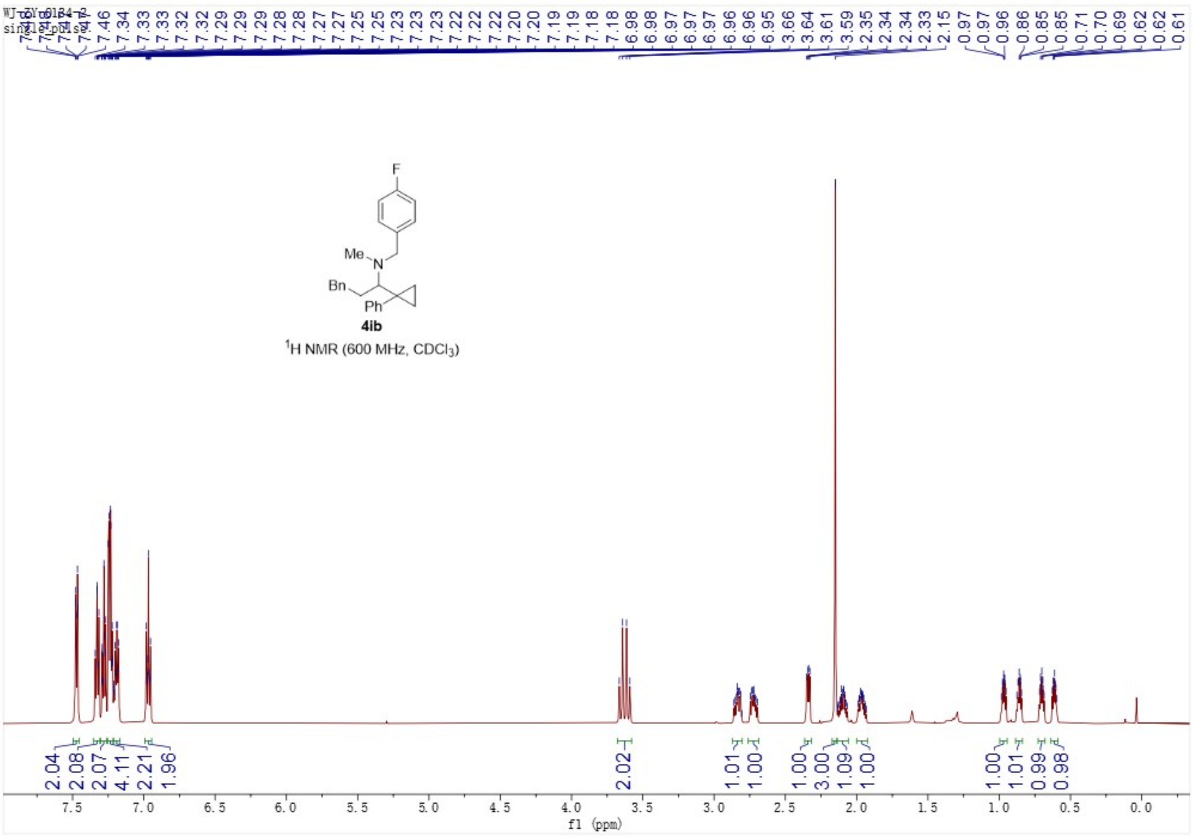
F
Me
N
Bn
Ph
4ib
1H NMR (600 MHz, CDCl3)
f1 (ppm)

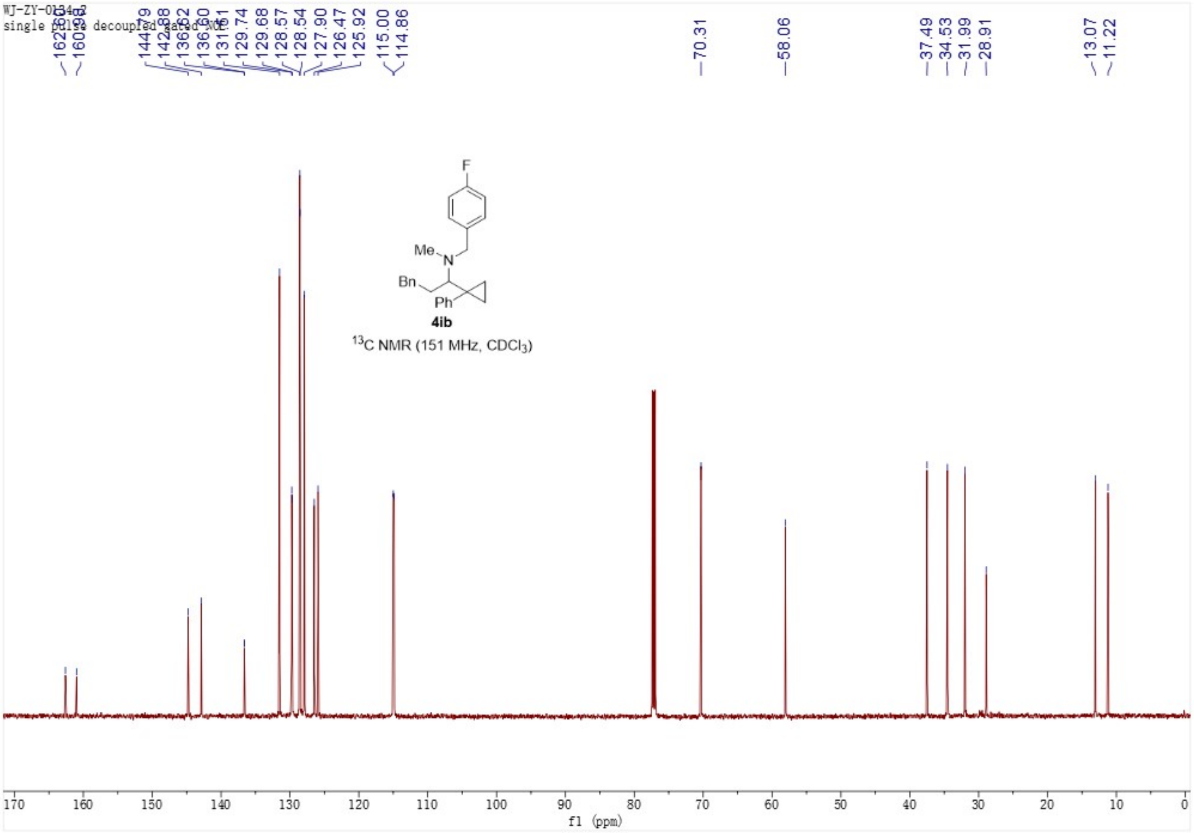
F
Me
N
Bn
Ph
4ib
13C NMR (151 MHz, CDCl3)
f1 (ppm)

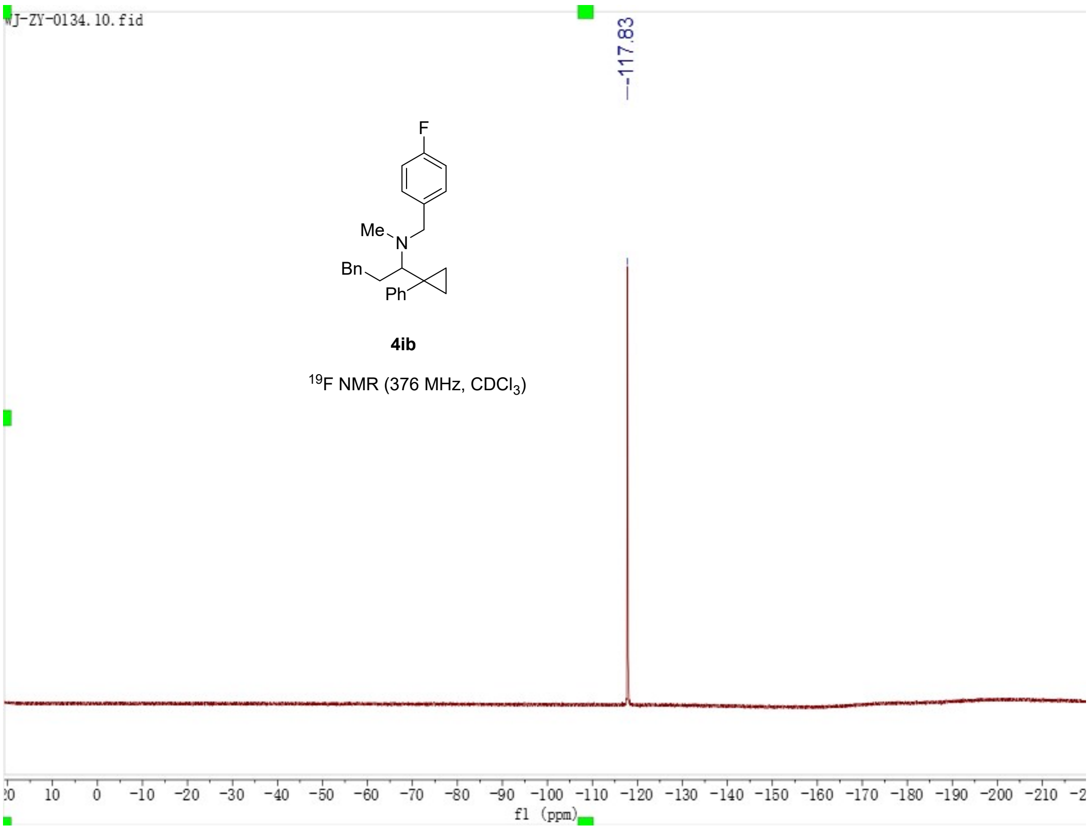

WJ-ZY-0134.10.fid
-117.83
F
Me
N
Bn
Ph
4ib
19F NMR (376 MHz, CDCl3)
20 10 0 -10 -20 -30 -40 -50 -60 -70 -80 -90 -100 -110 -120 -130 -140 -150 -160 -170 -180 -190 -200 -210
f1 (ppm)

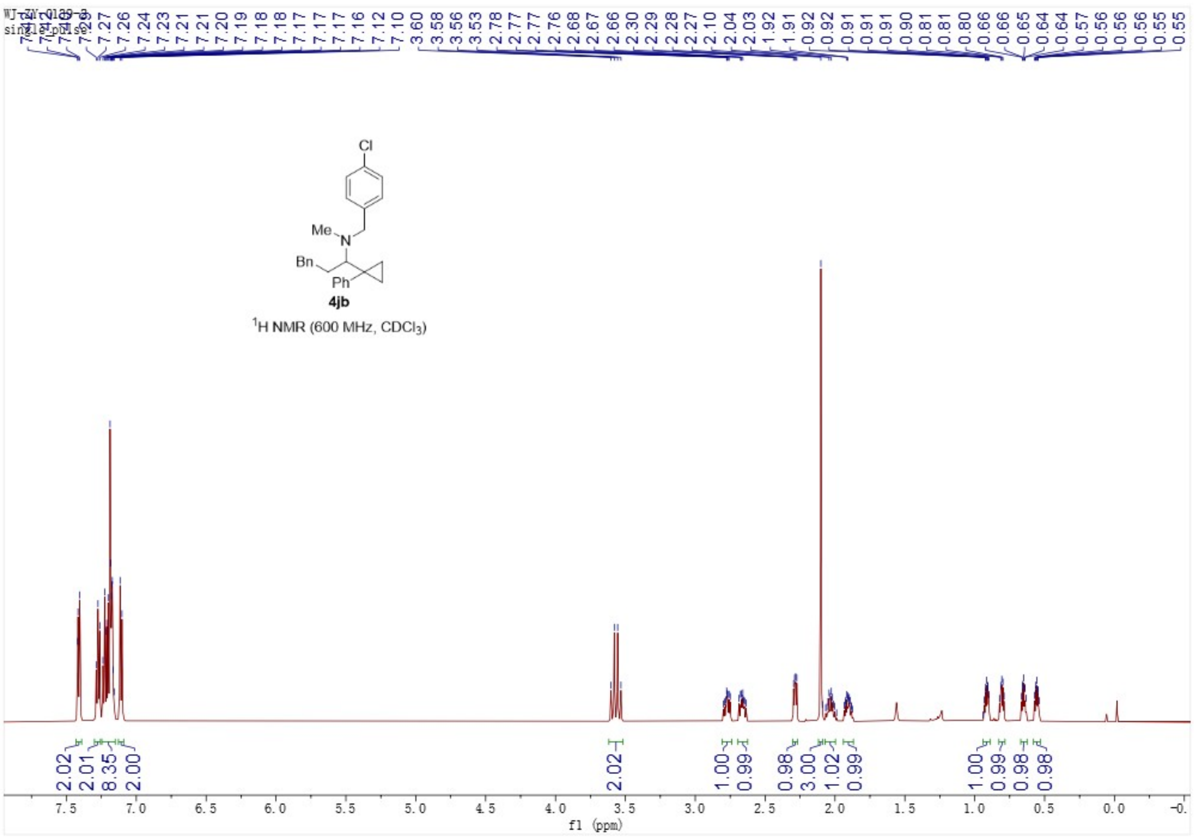

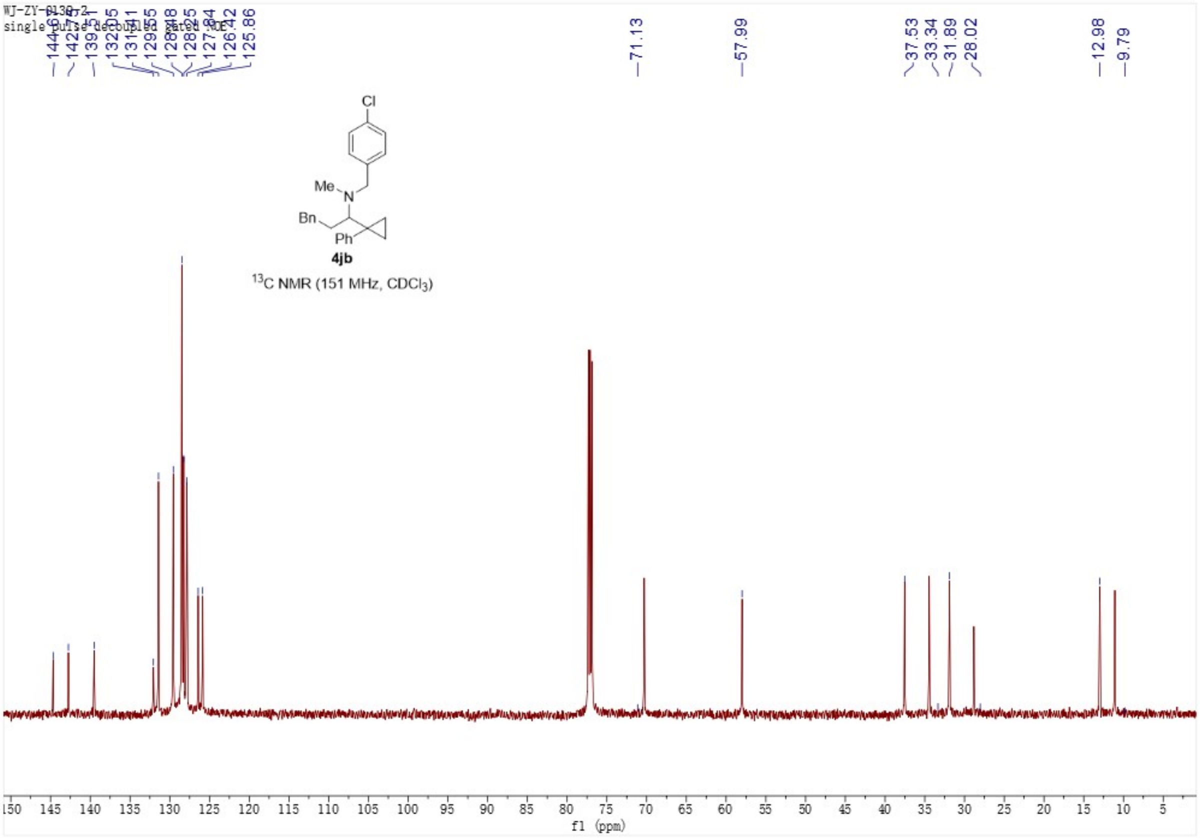

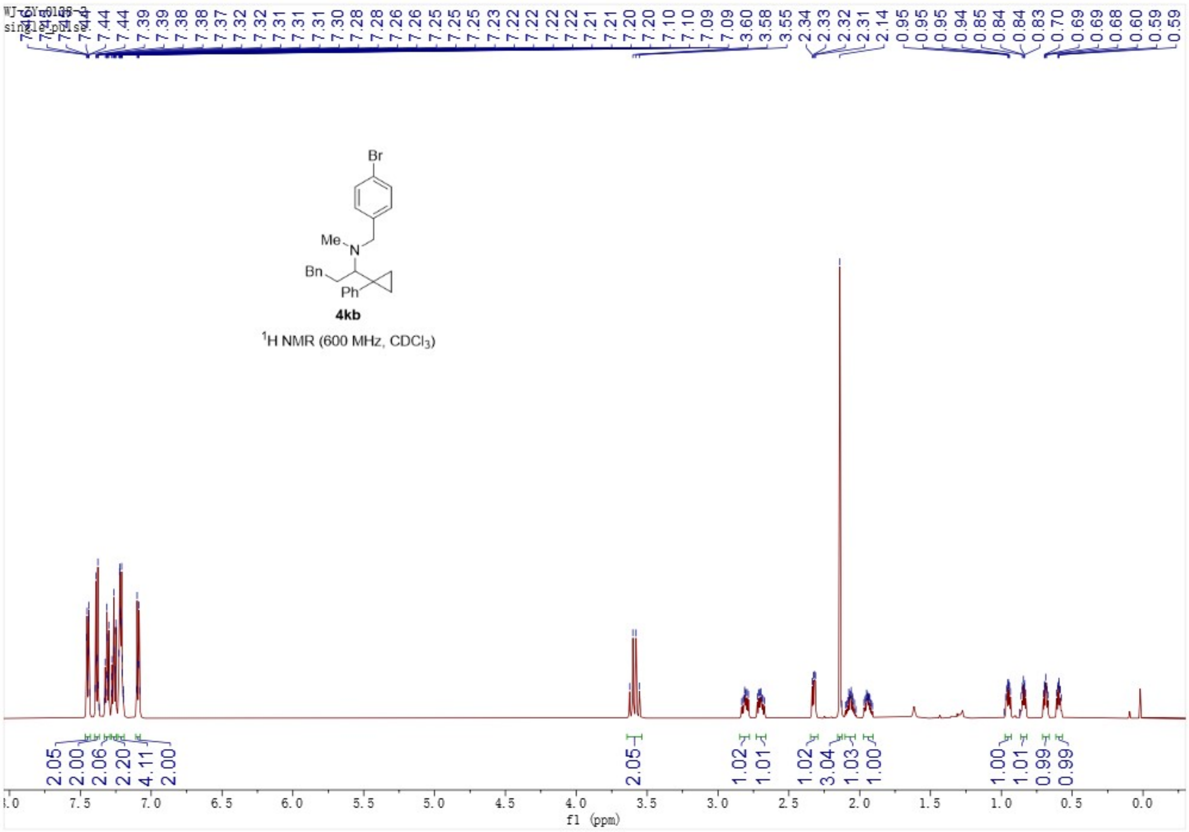

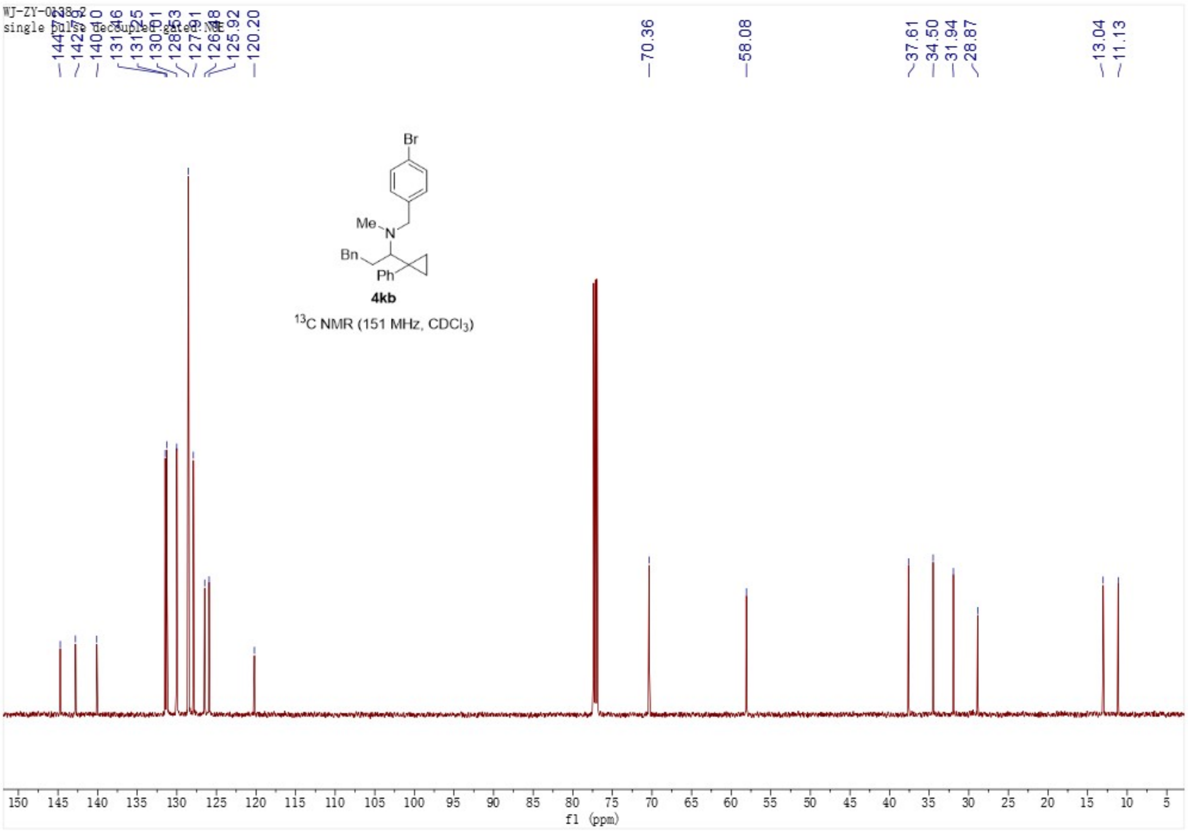

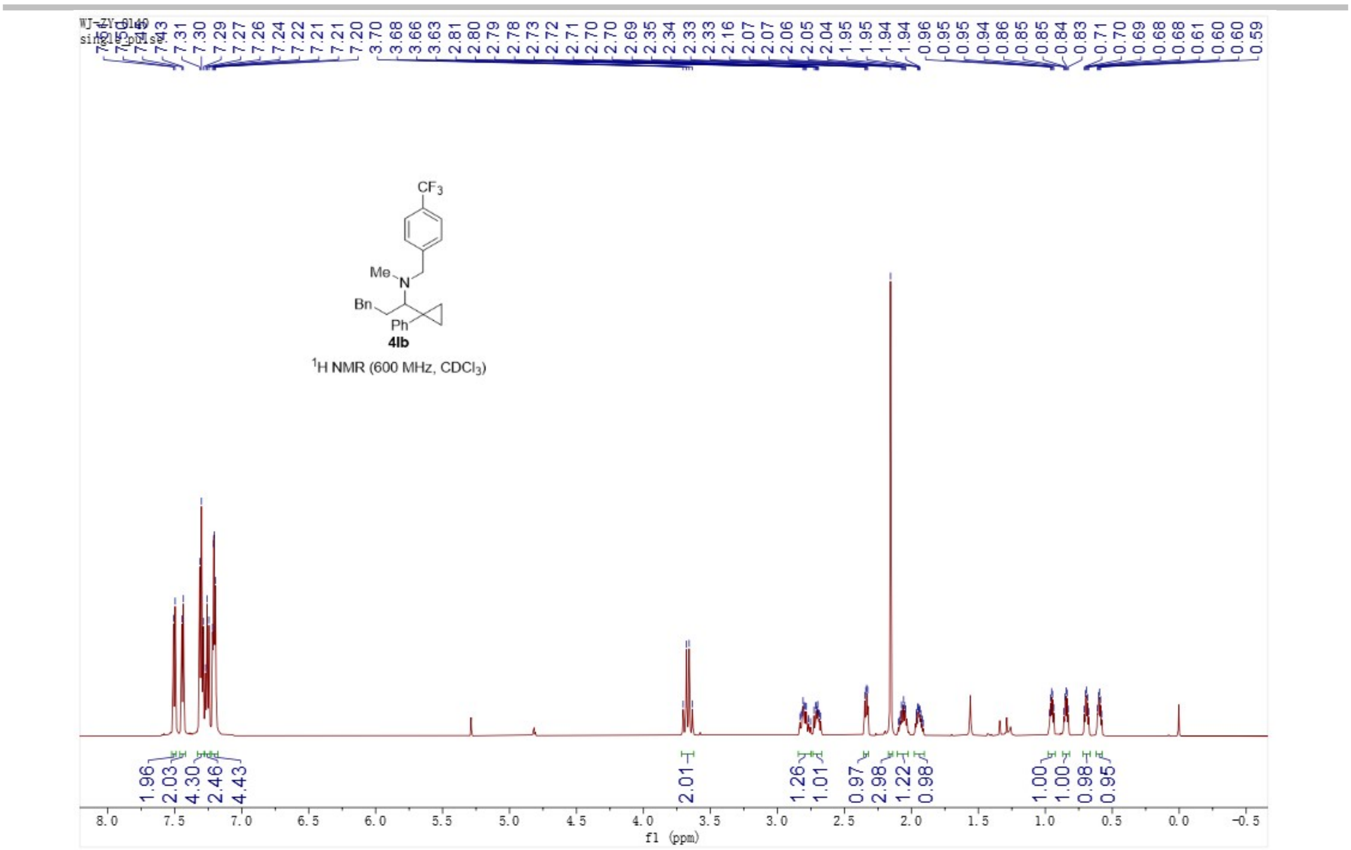
4lb
1H NMR (600 MHz, CDCl3)

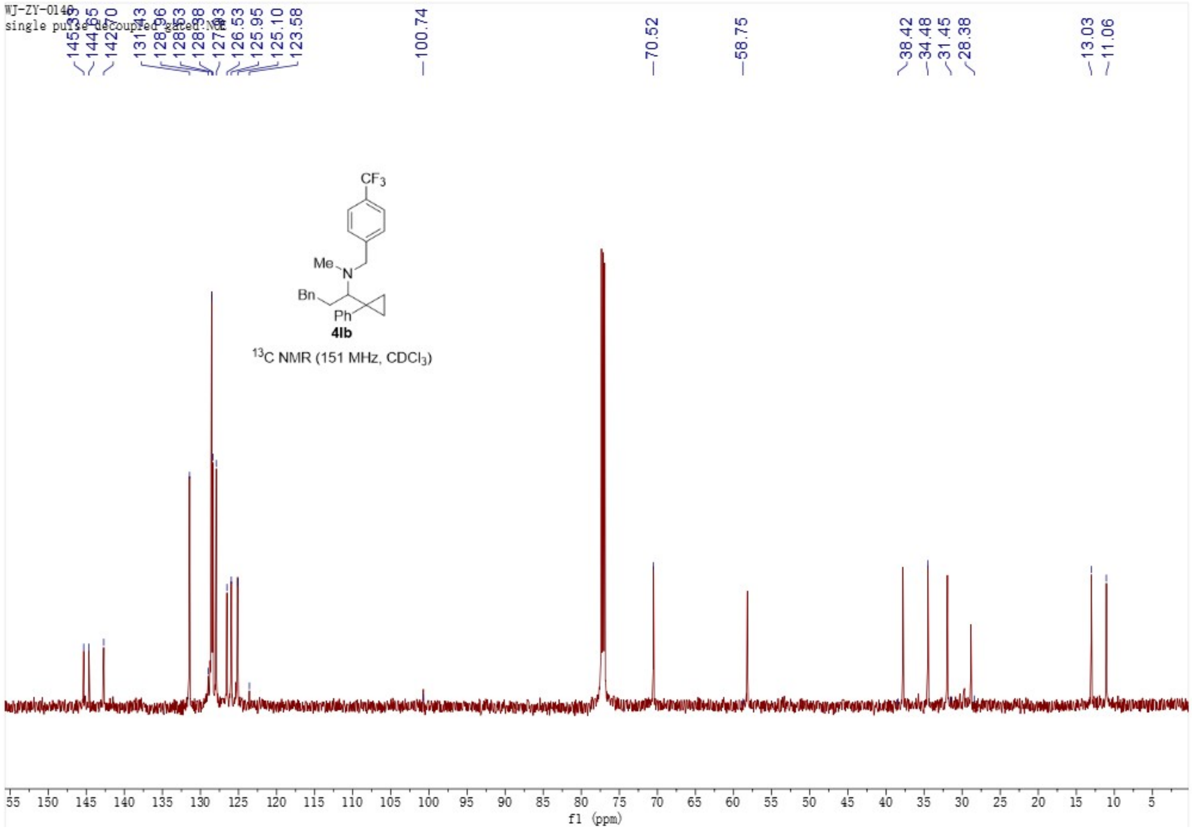
4lb
13C NMR (151 MHz, CDCl3)

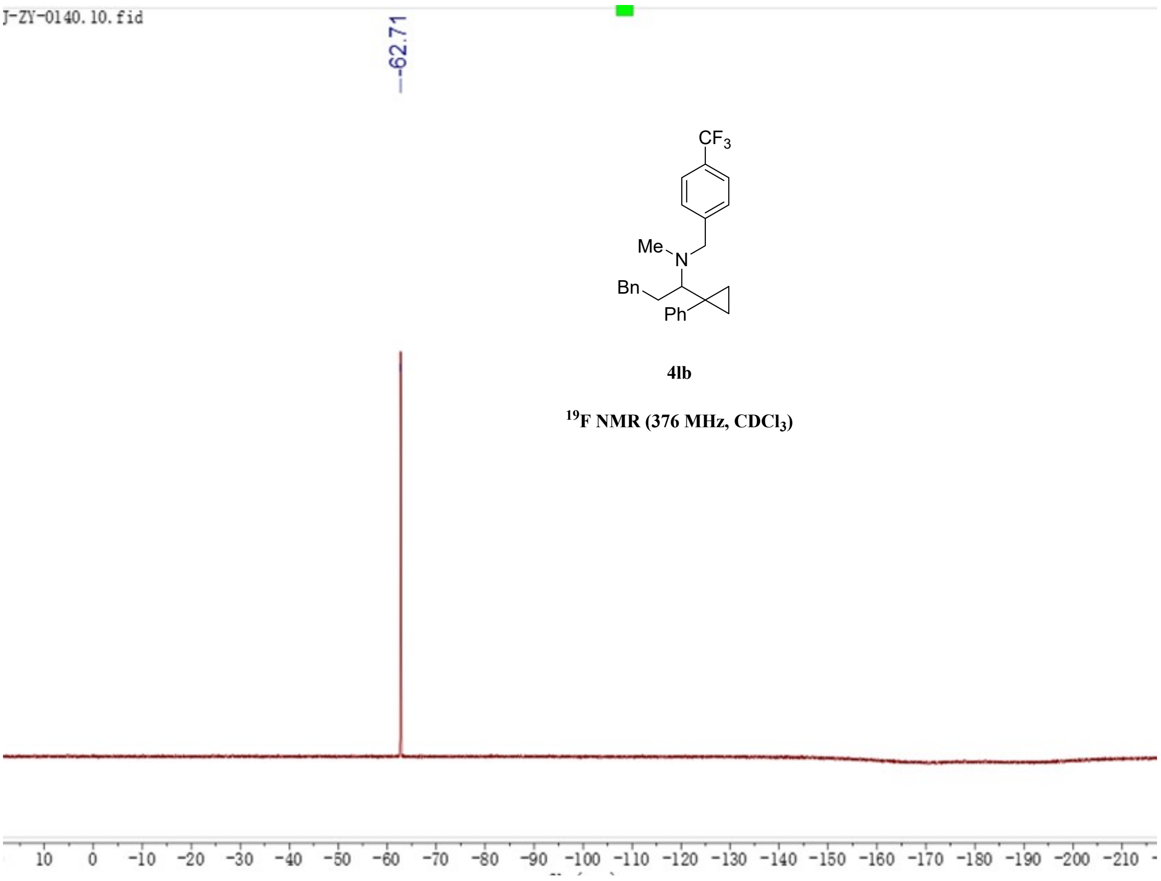

4lb

^{19}F NMR (376 MHz, $CDCl_3$)

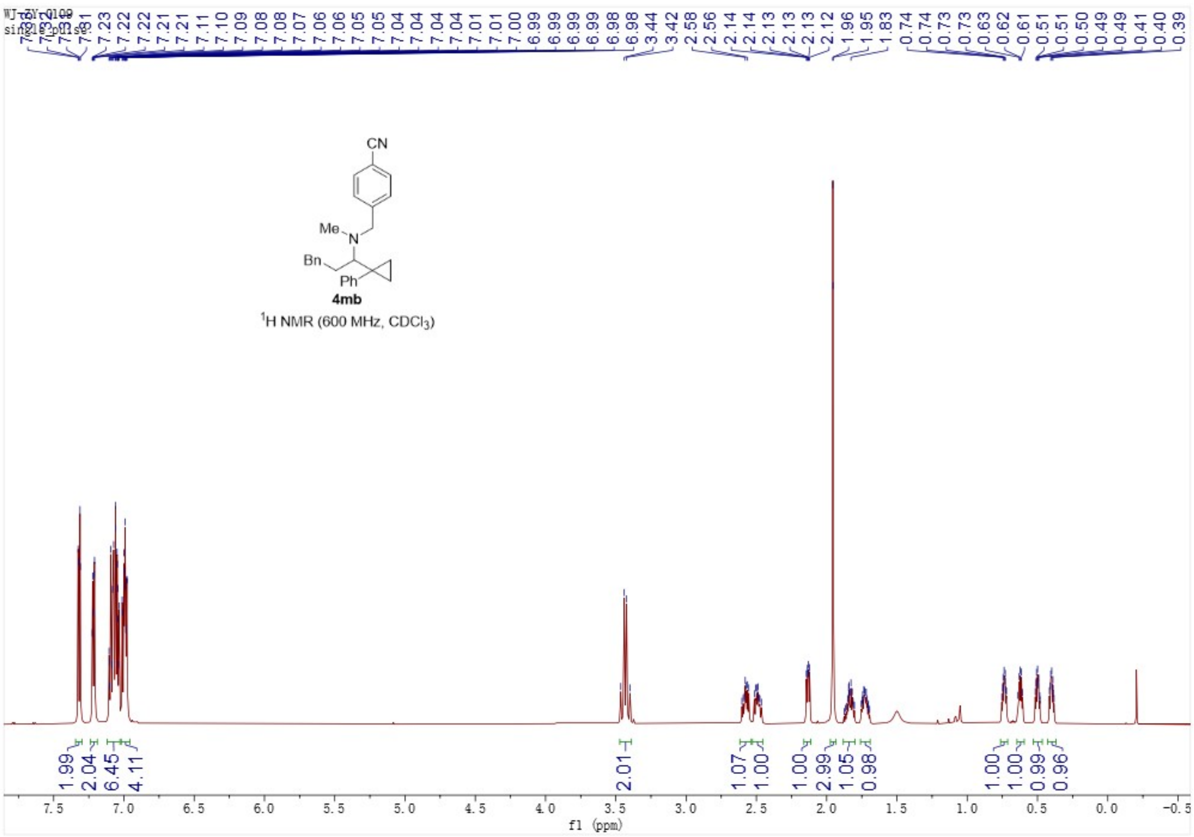
WJ-ZY-0109
single pulse
CN
Me
N
Bn
Ph
4mb
1H NMR (600 MHz, CDCl3)
f1 (ppm)

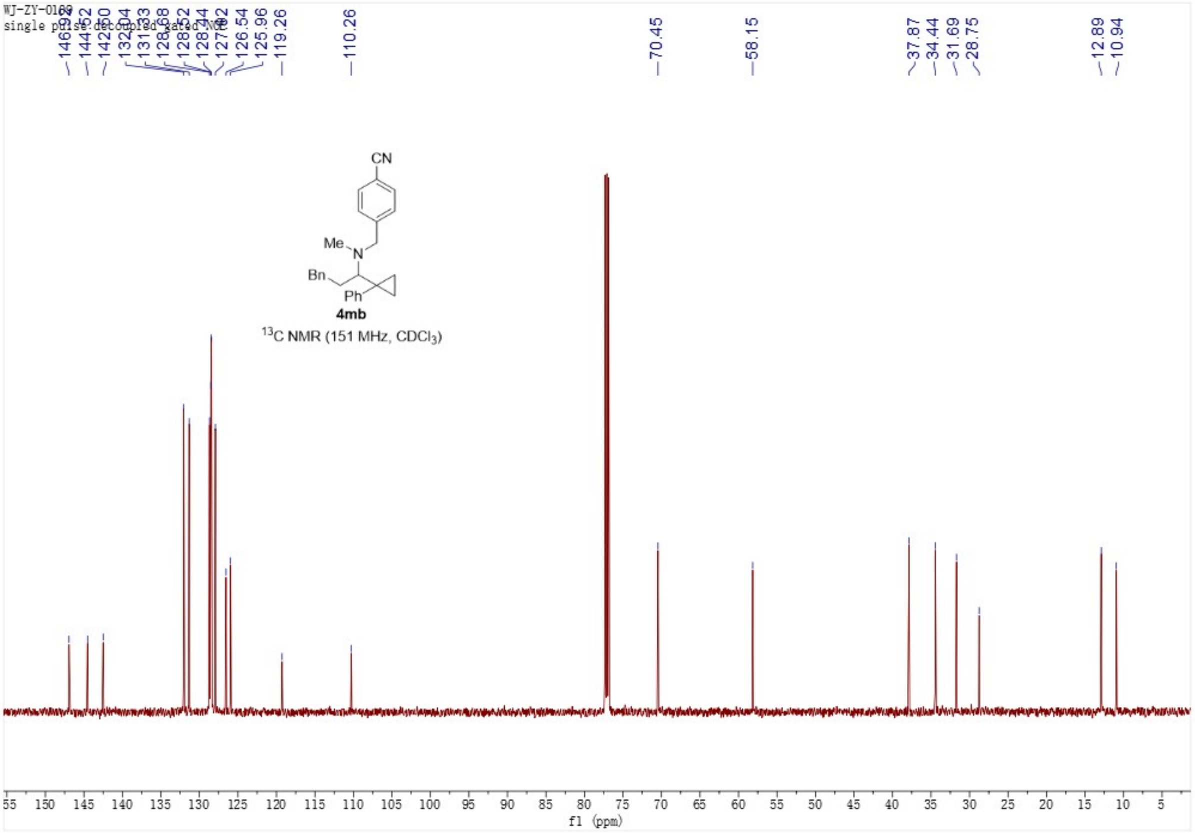
WJ-ZY-0109
single pulse decoupled gated NOE
CN
Me
N
Bn
Ph
4mb
13C NMR (151 MHz, CDCl3)
f1 (ppm)

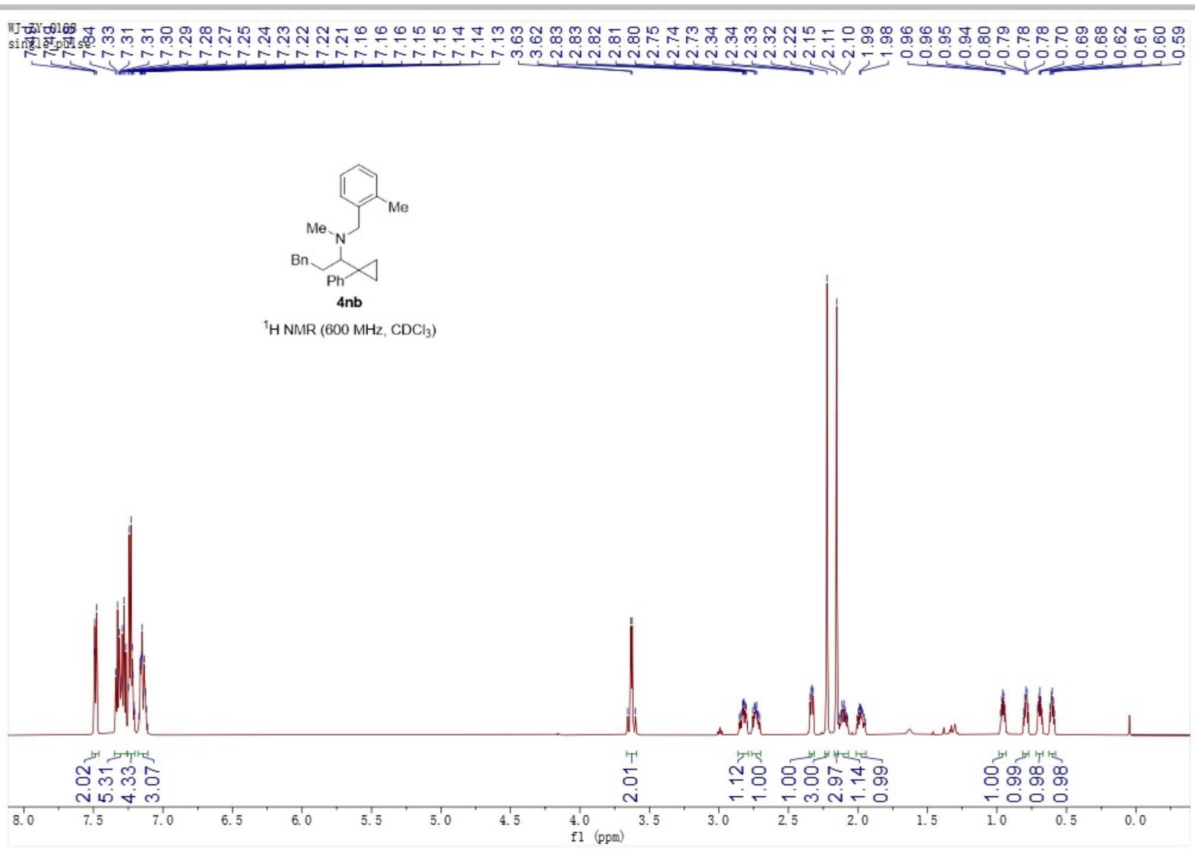

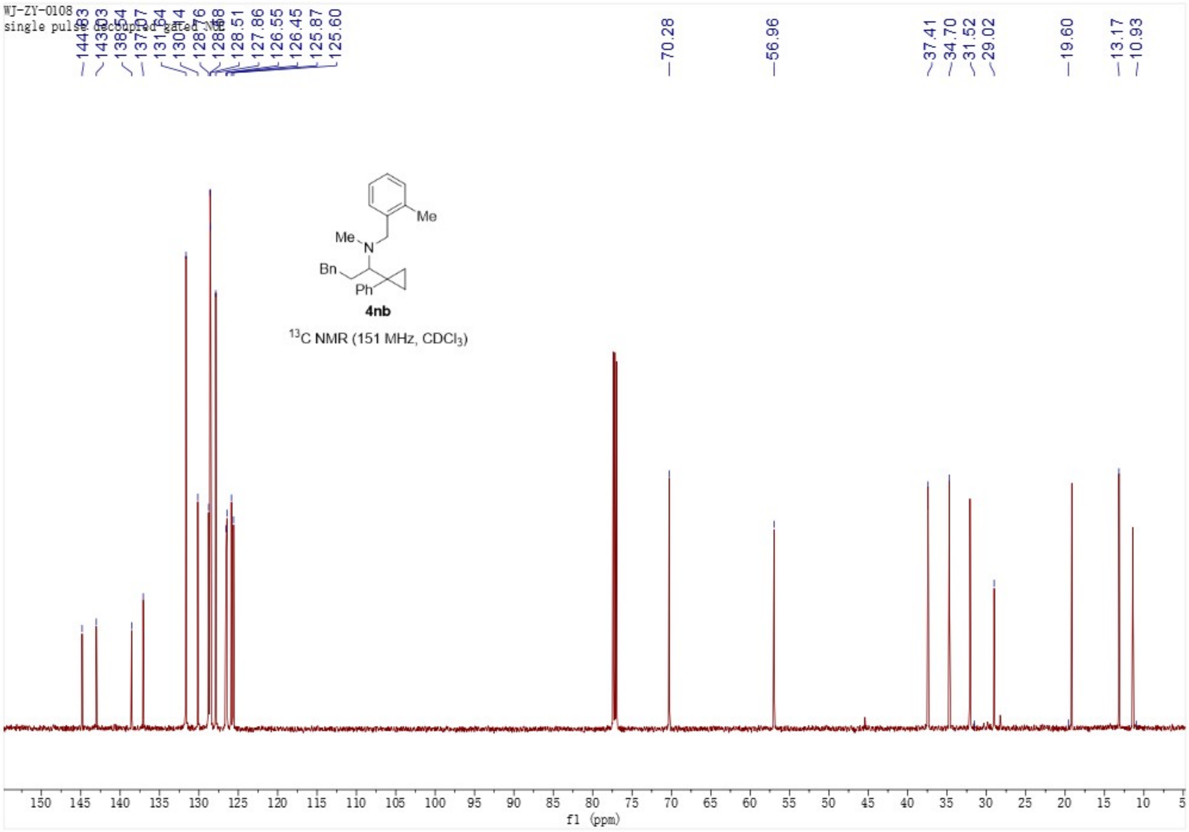

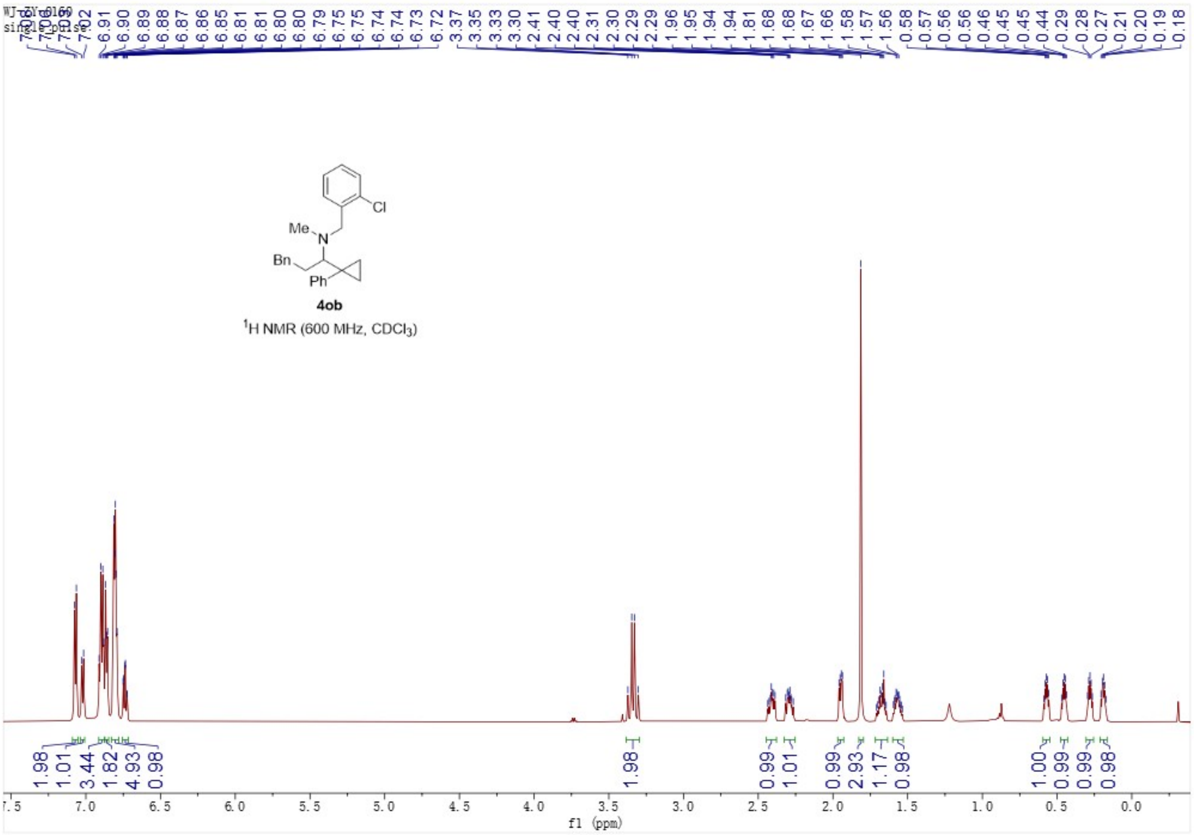

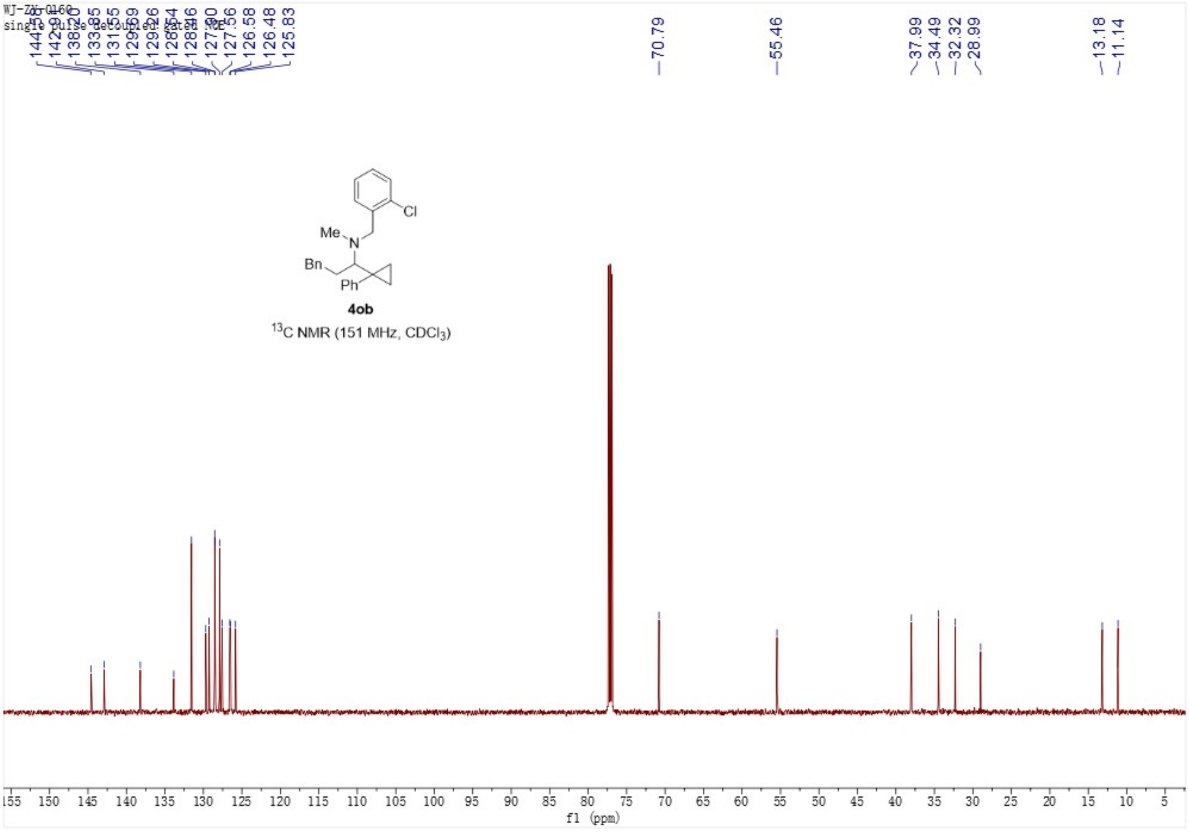

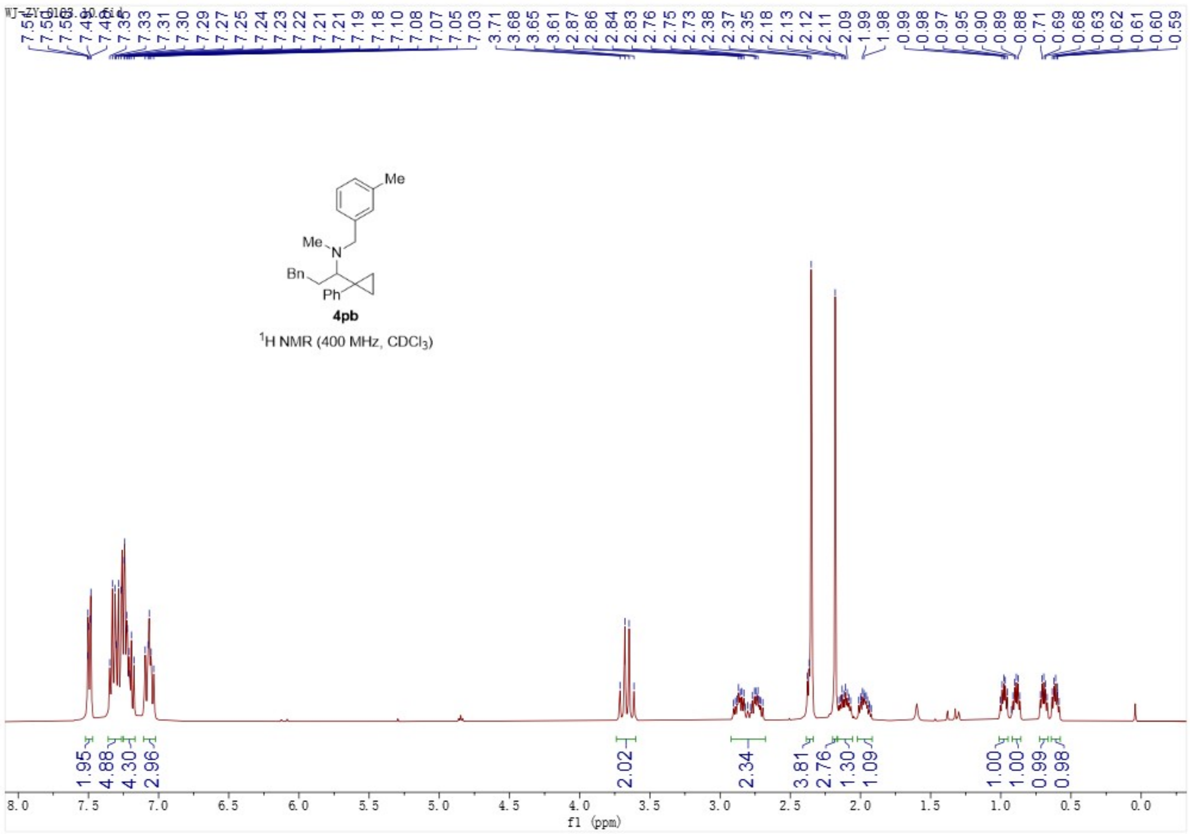

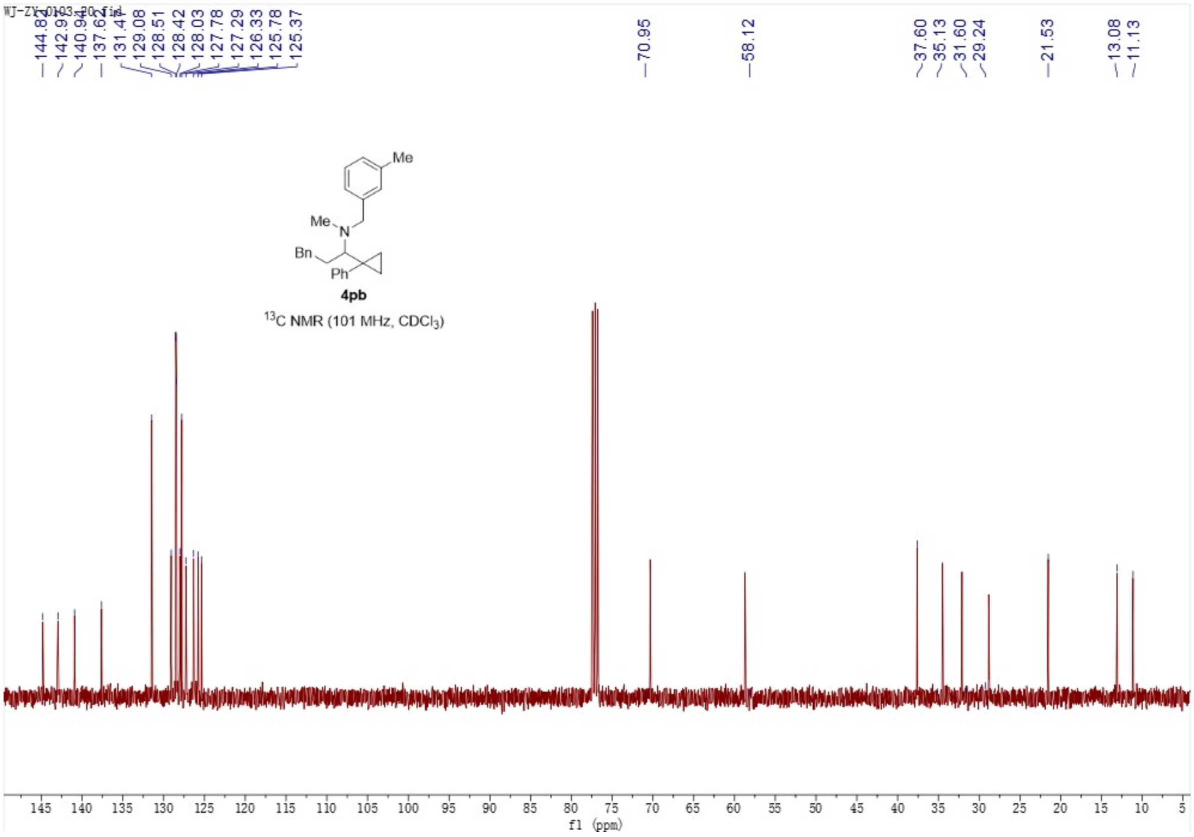

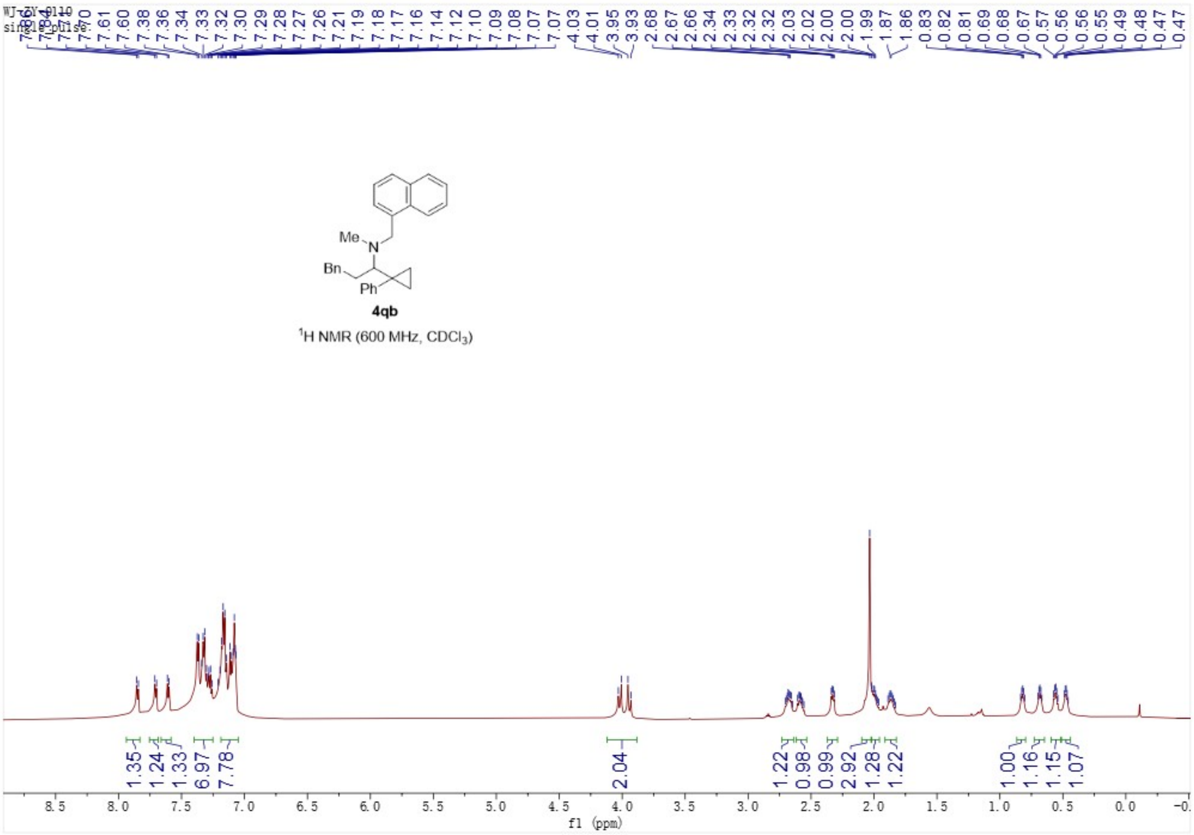
WJ-ZY-0110
7.61 7.60 7.38 7.36 7.34 7.33 7.32 7.30 7.29 7.28 7.27 7.26 7.21 7.19 7.18 7.17 7.16 7.14 7.12 7.10 7.09 7.08 7.07 7.07 4.03 4.01 3.95 3.93 2.68 2.67 2.66 2.34 2.33 2.32 2.32 2.03 2.02 2.00 2.00 1.99 1.87 1.86 0.83 0.82 0.81 0.69 0.68 0.67 0.57 0.56 0.55 0.49 0.48 0.47 0.47
Me
N
Bn
Ph
4qb
^{1}H NMR (600 MHz, CDCl$_3$)
1.35 1.24 1.33 6.97 7.78 2.04 1.22 0.98 0.99 2.92 1.28 1.22 1.00 1.16 1.15 1.07
f1 (ppm)

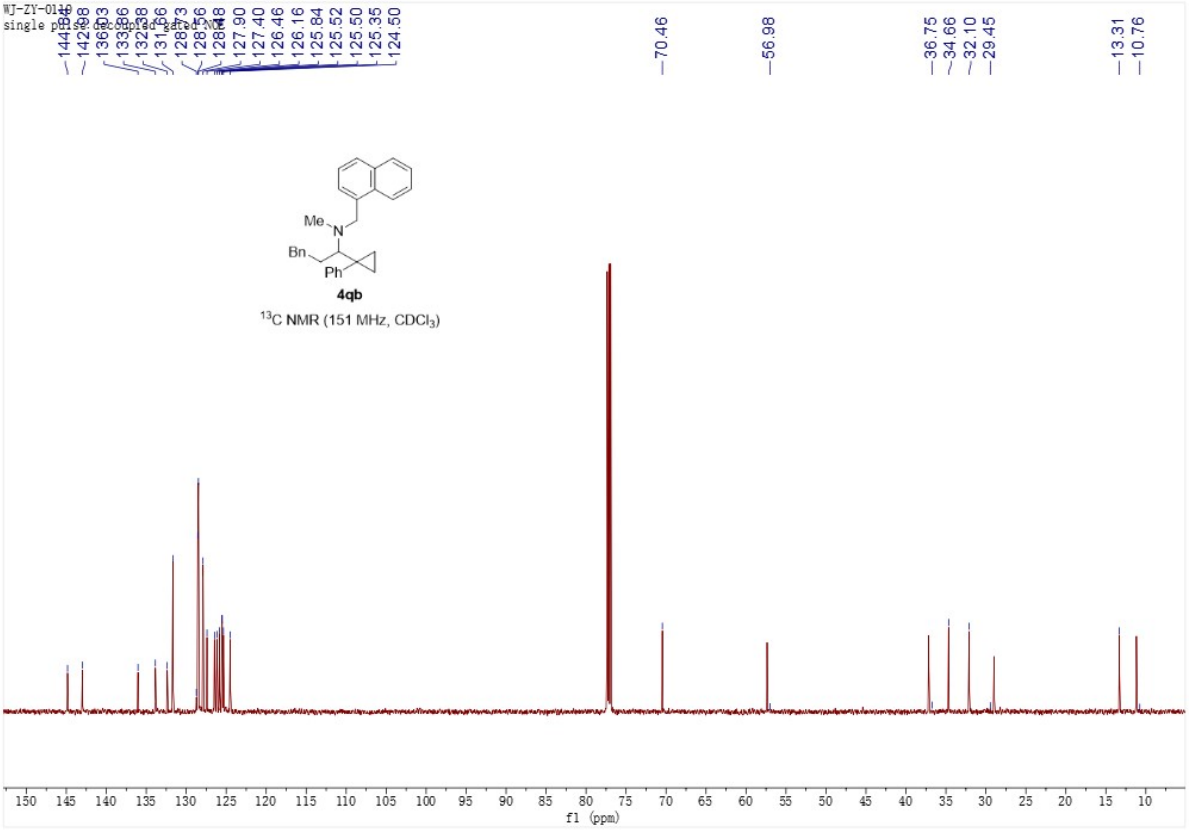
WJ-ZY-0110
single pulse decoupled gated NOE
136.03 133.86 132.38 131.66 127.90 127.40 126.46 126.16 125.84 125.52 125.50 125.35 124.50
70.46
56.98
36.75 34.66 32.10 29.45
13.31 10.76
Me
N
Bn
Ph
4qb
^{13}C NMR (151 MHz, CDCl$_3$)
f1 (ppm)

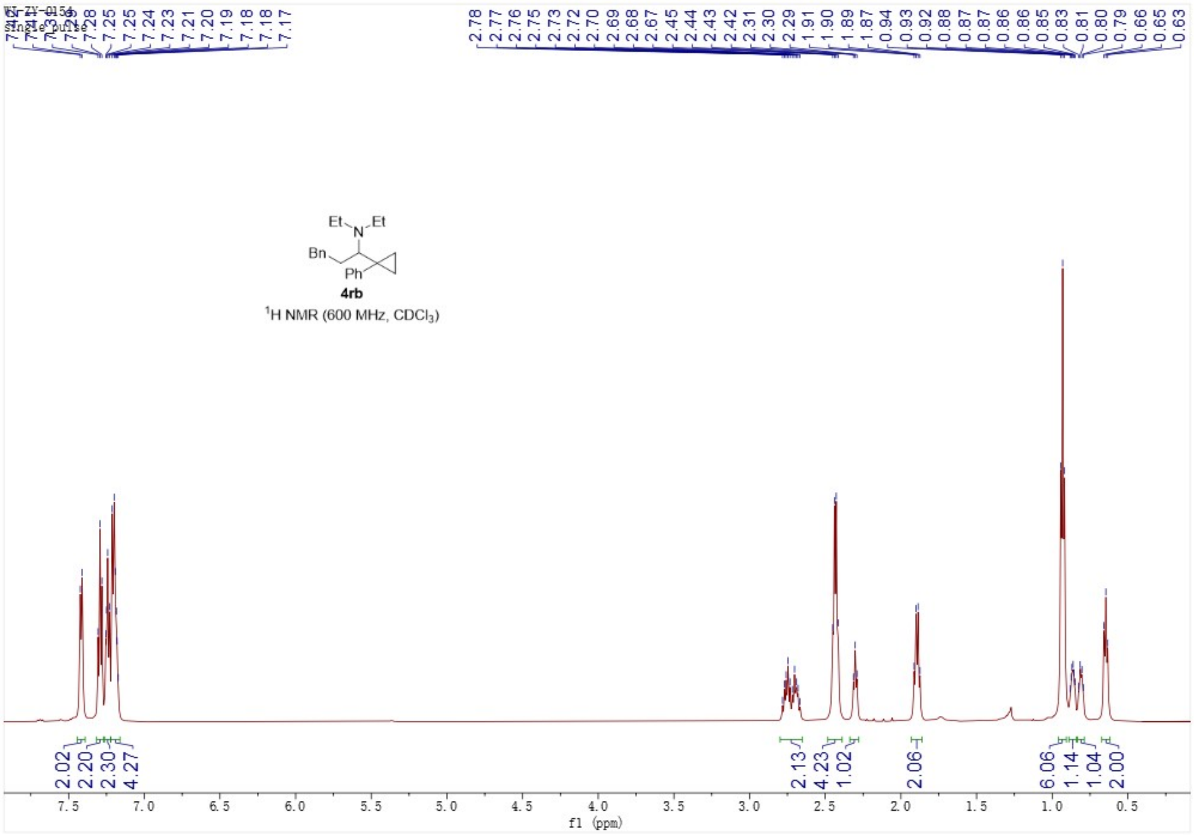

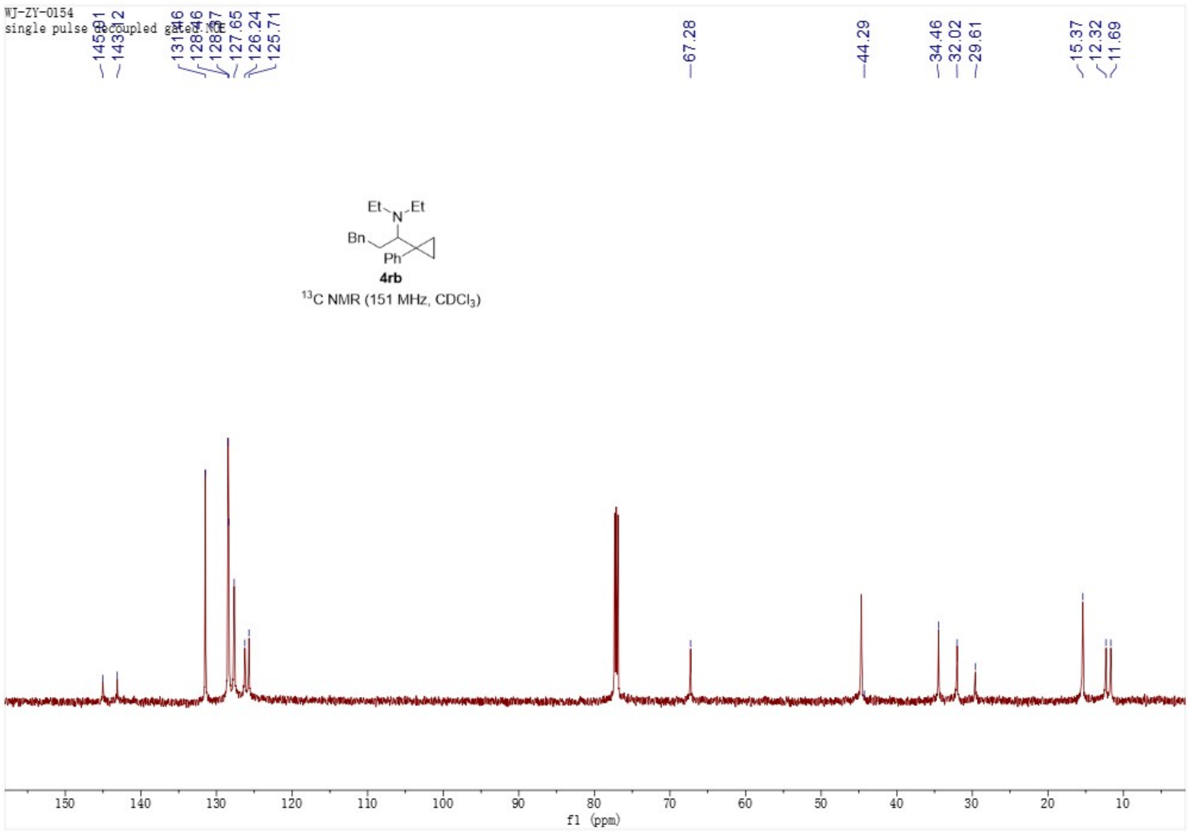

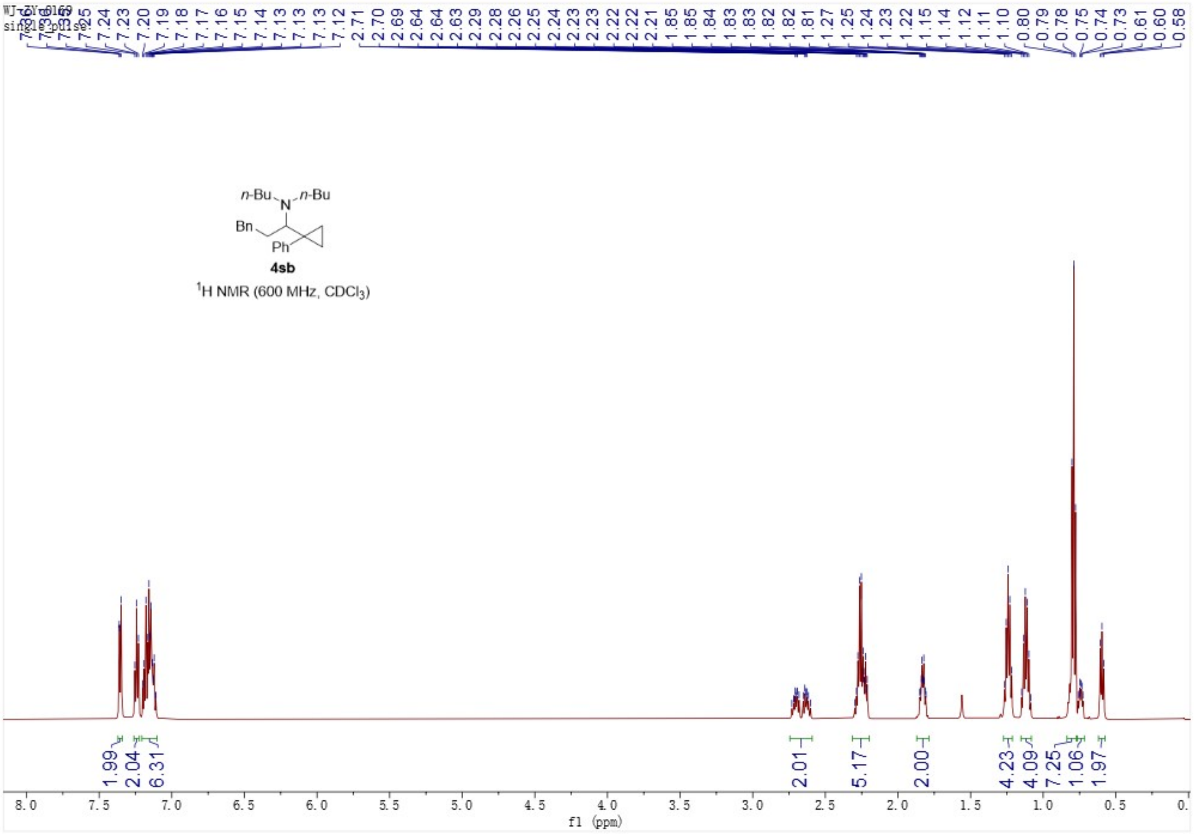
n-Bu
N
n-Bu
Bn
Ph
4sb
^{1}H NMR (600 MHz, CDCl$_3$)
f1 (ppm)

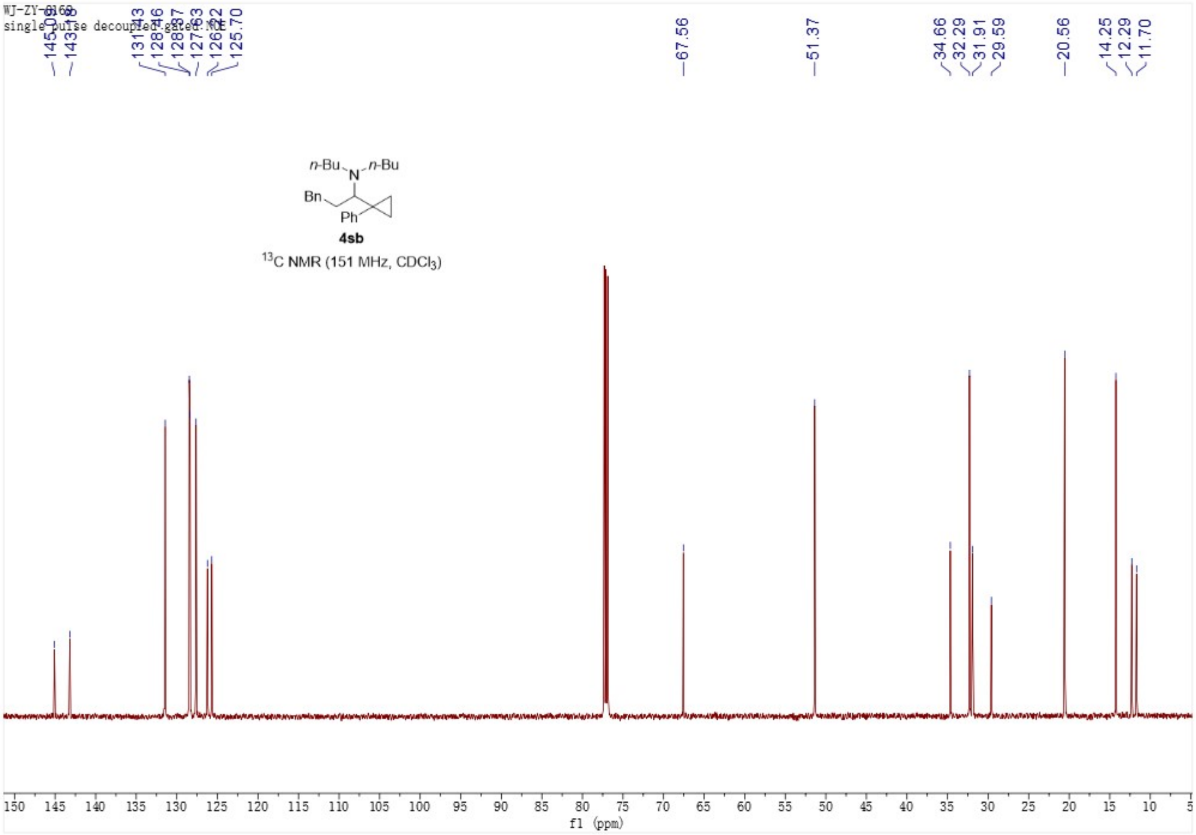
n-Bu
N
n-Bu
Bn
Ph
4sb
^{13}C NMR (151 MHz, CDCl$_3$)
67.56
51.37
34.66
32.29
31.91
29.59
20.56
14.25
12.29
11.70
f1 (ppm)

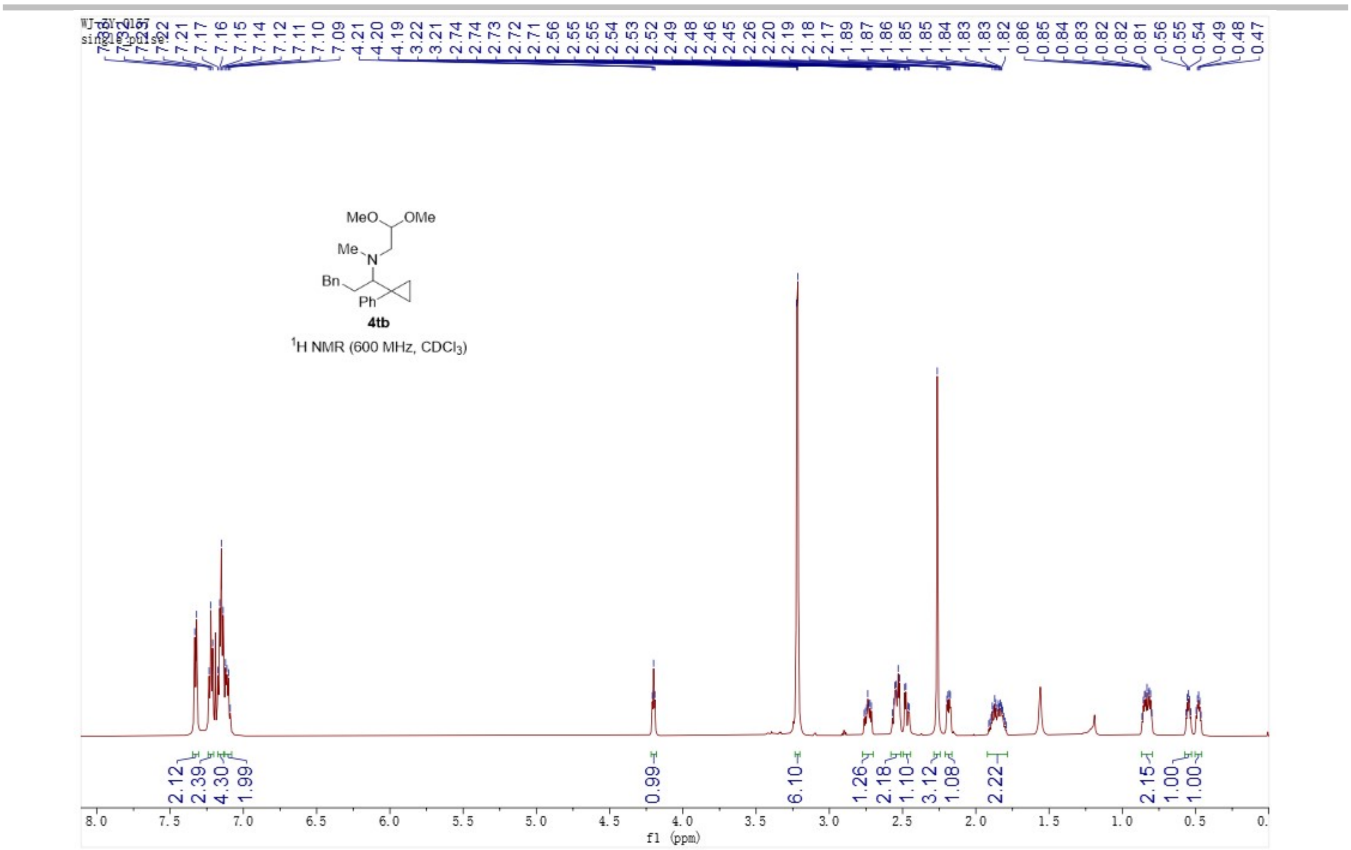

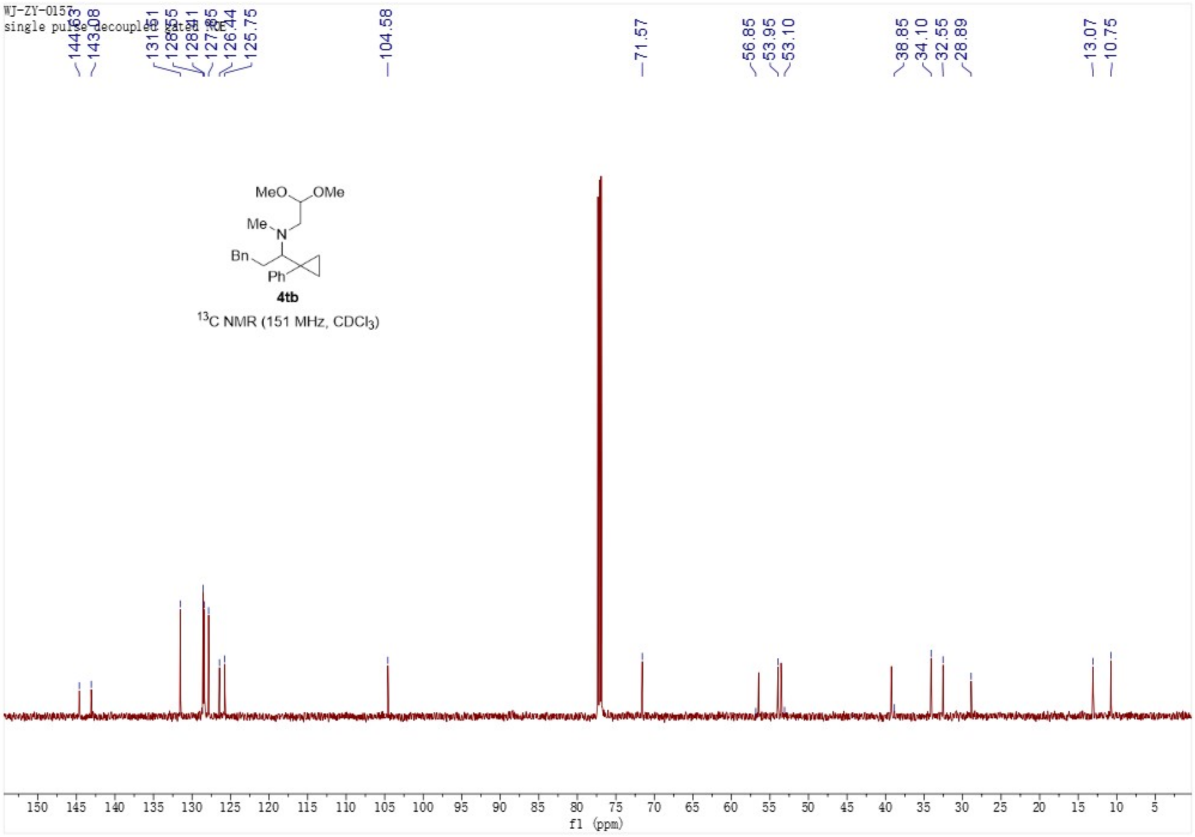

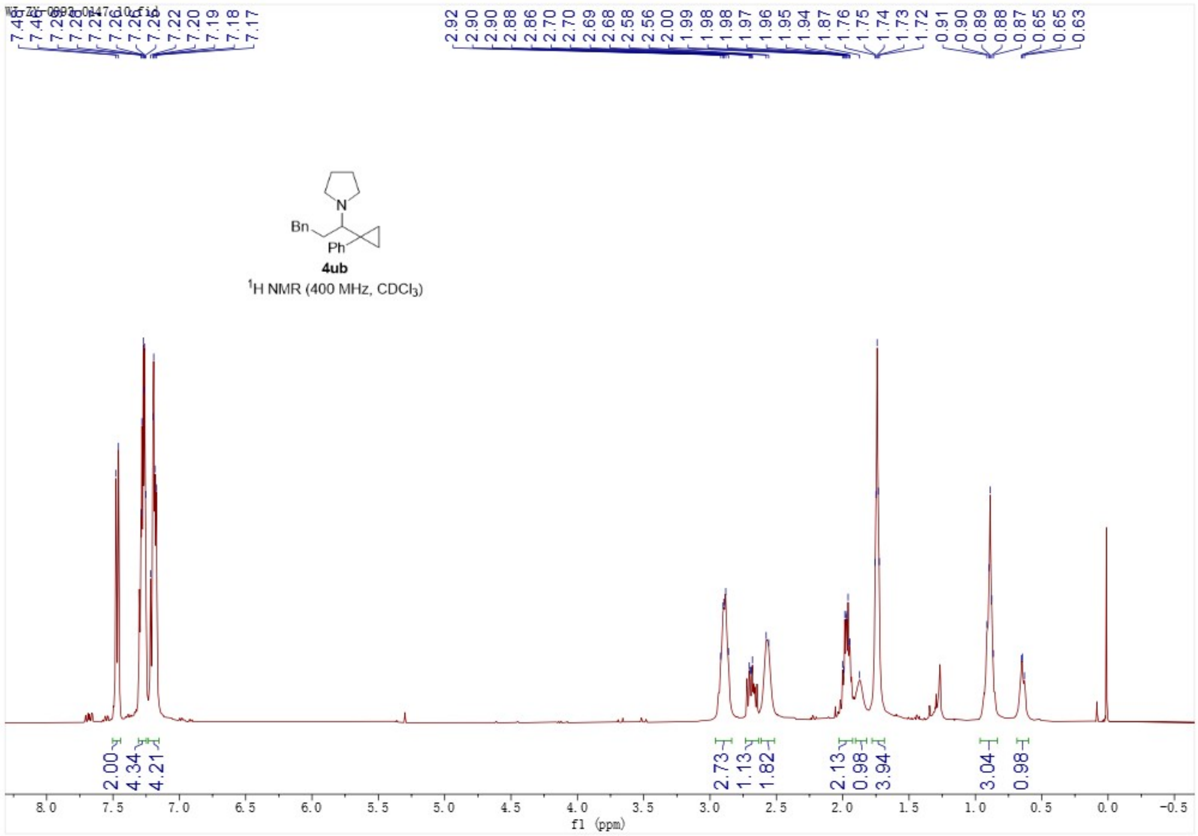

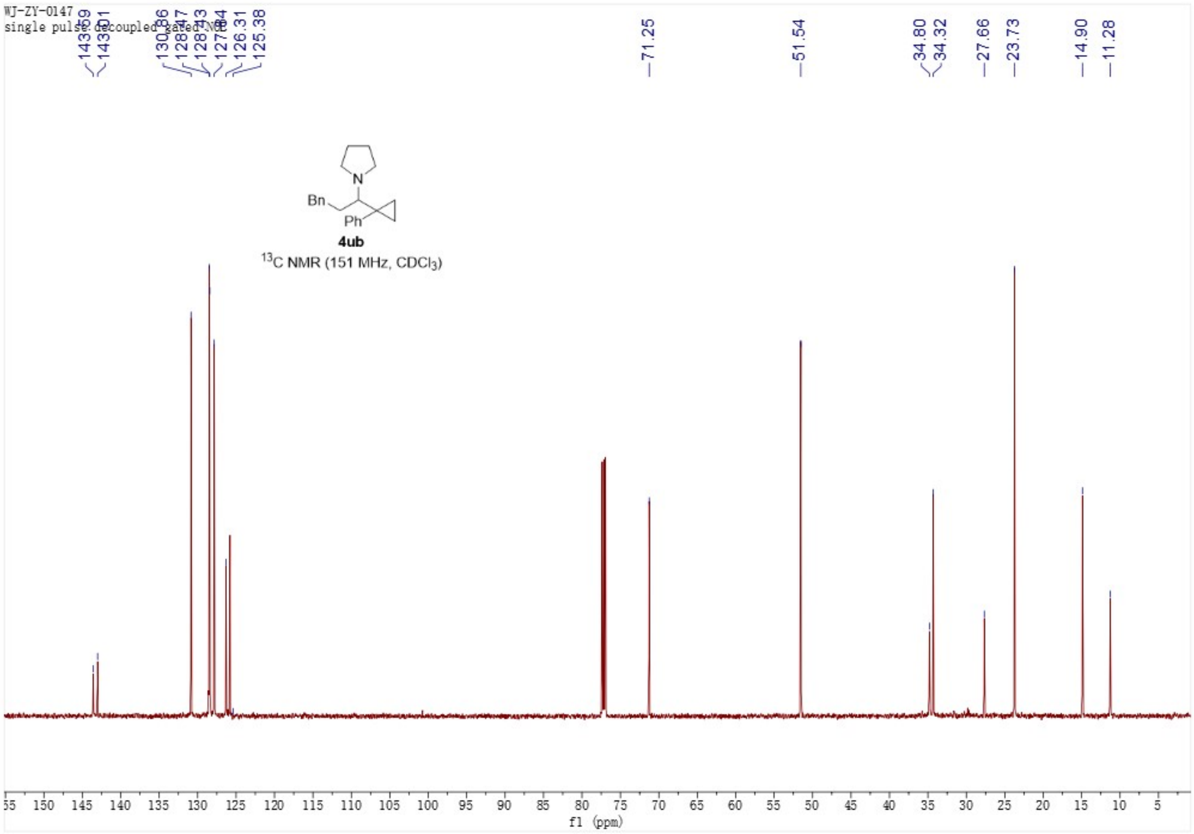

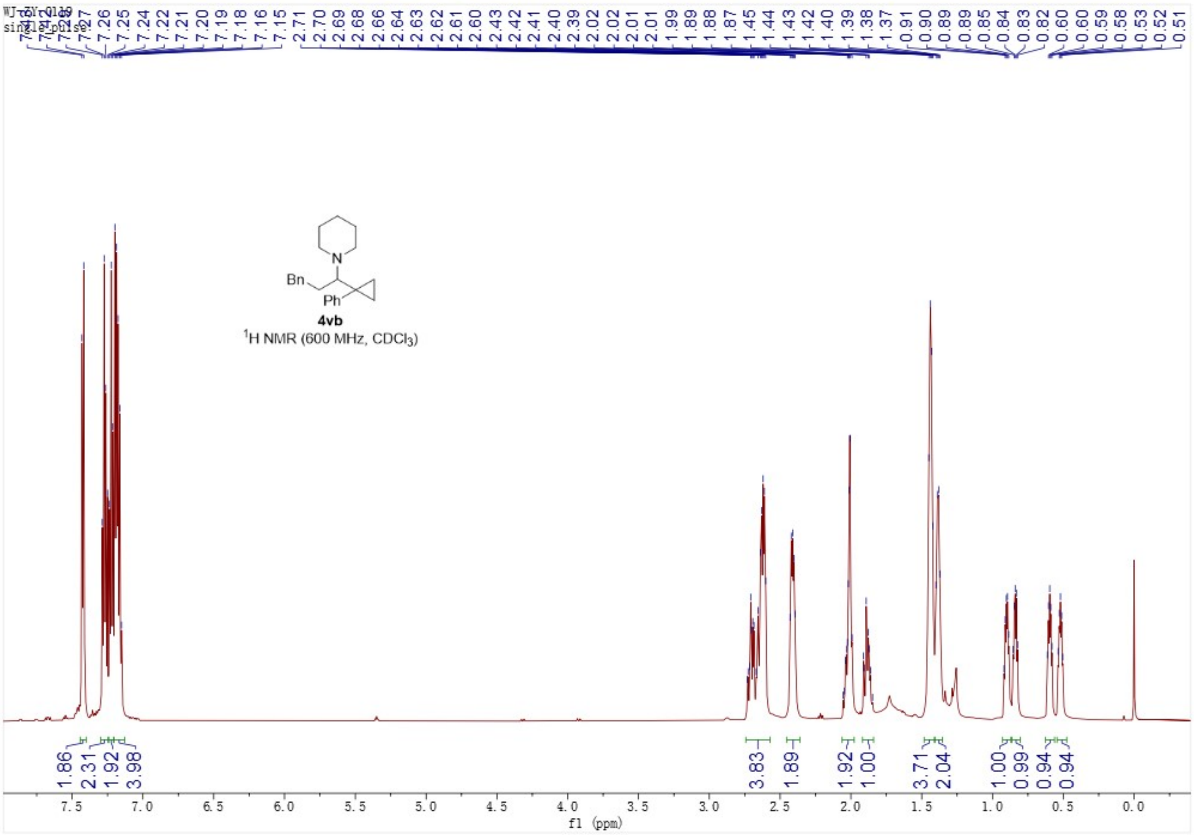

4vb
^{1}H NMR (600 MHz, CDCl$_3$)
f1 (ppm)

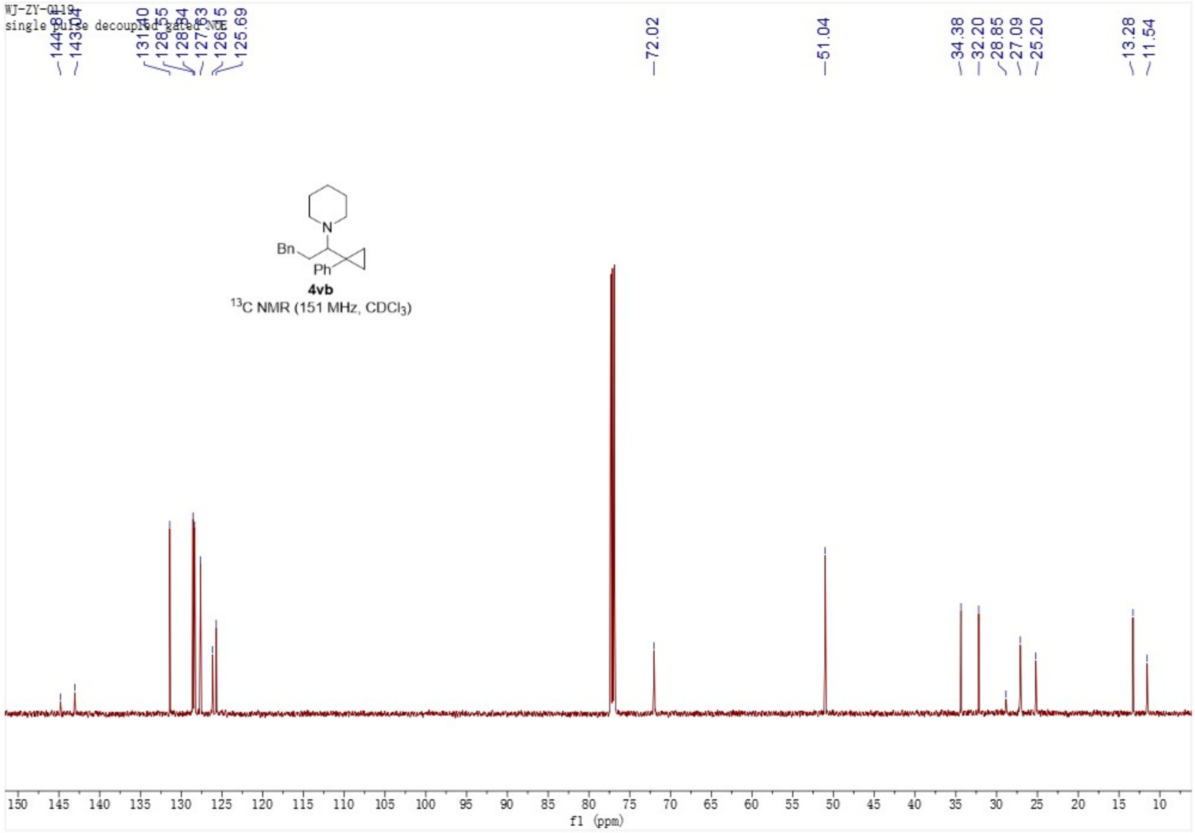

4vb
^{13}C NMR (151 MHz, CDCl$_3$)
f1 (ppm)

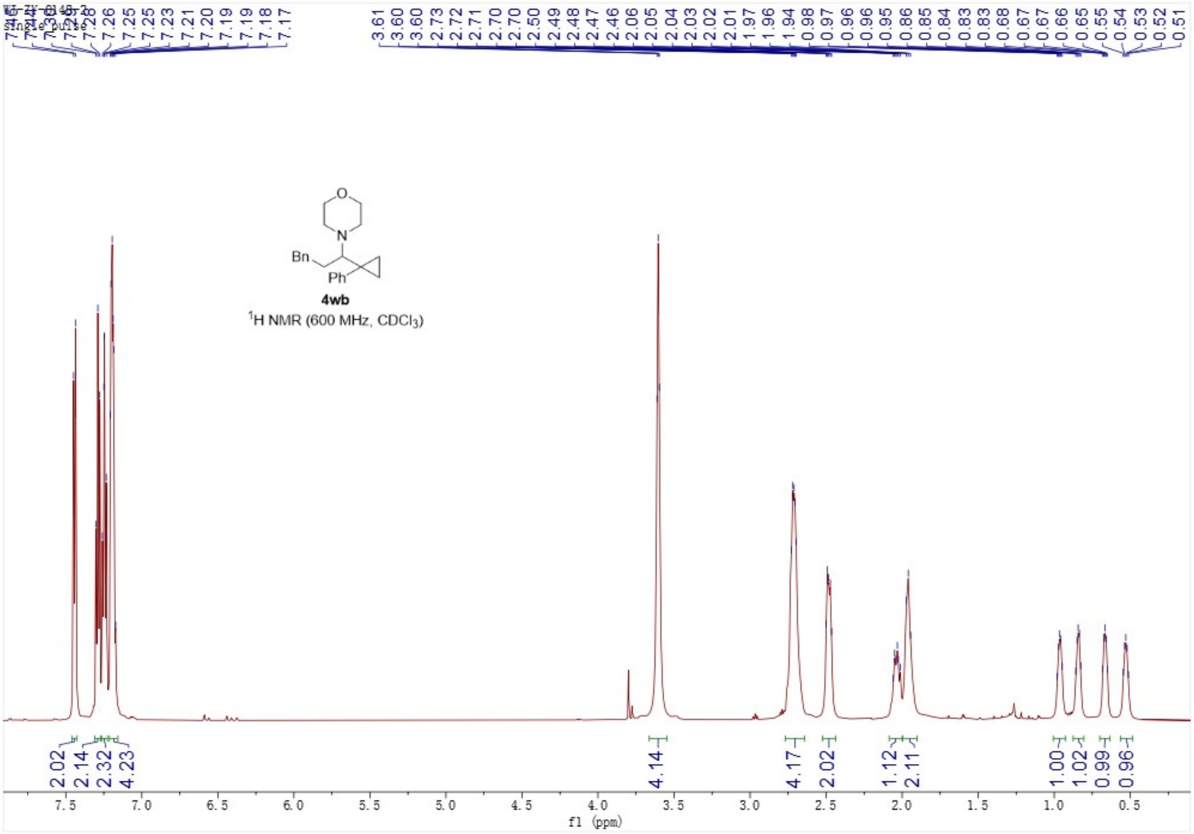

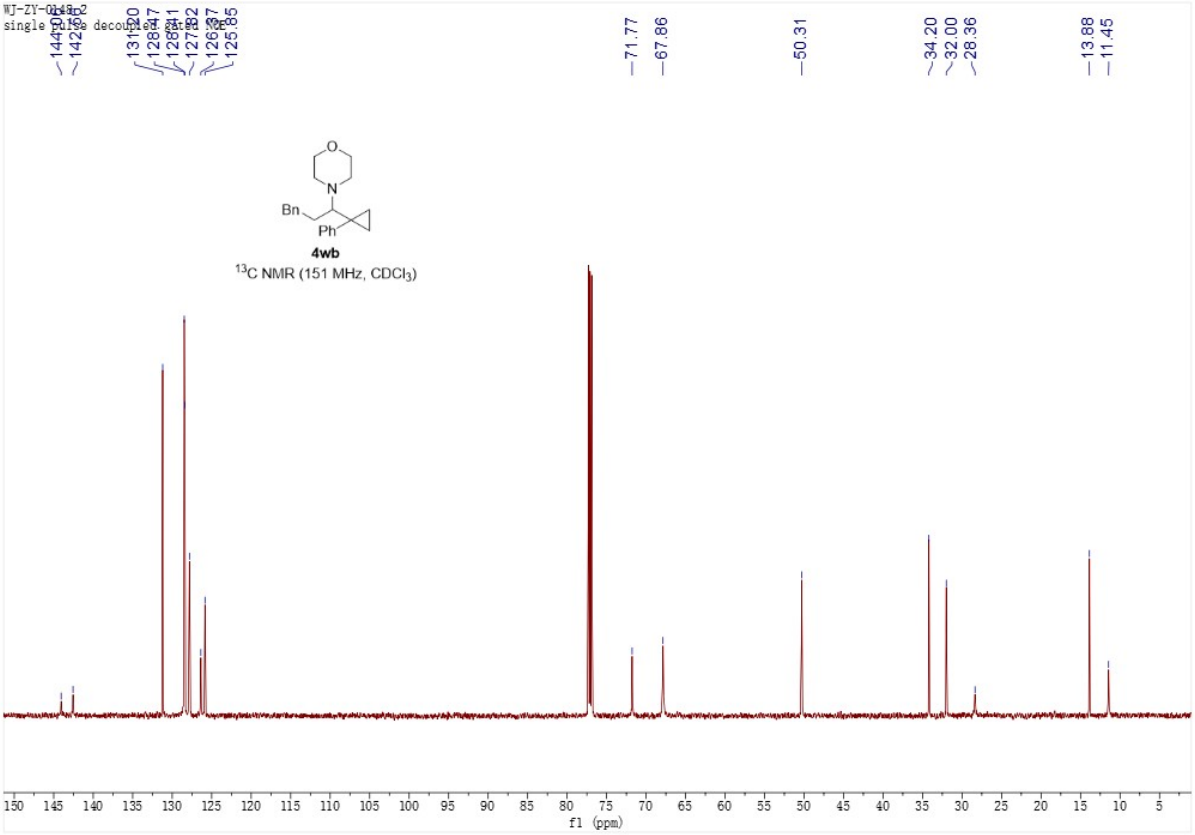

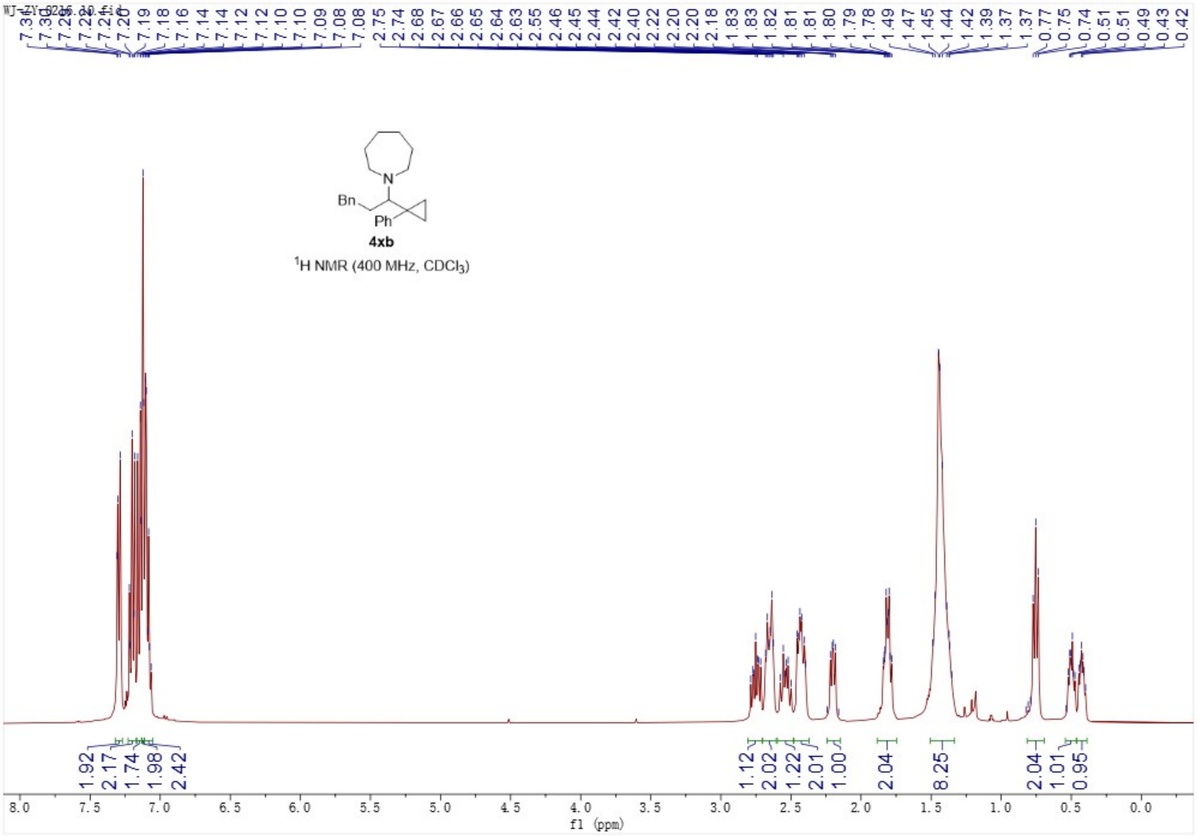
Bn
Ph
N
4xb
^{1}H NMR (400 MHz, $CDCl_3$)
f1 (ppm)

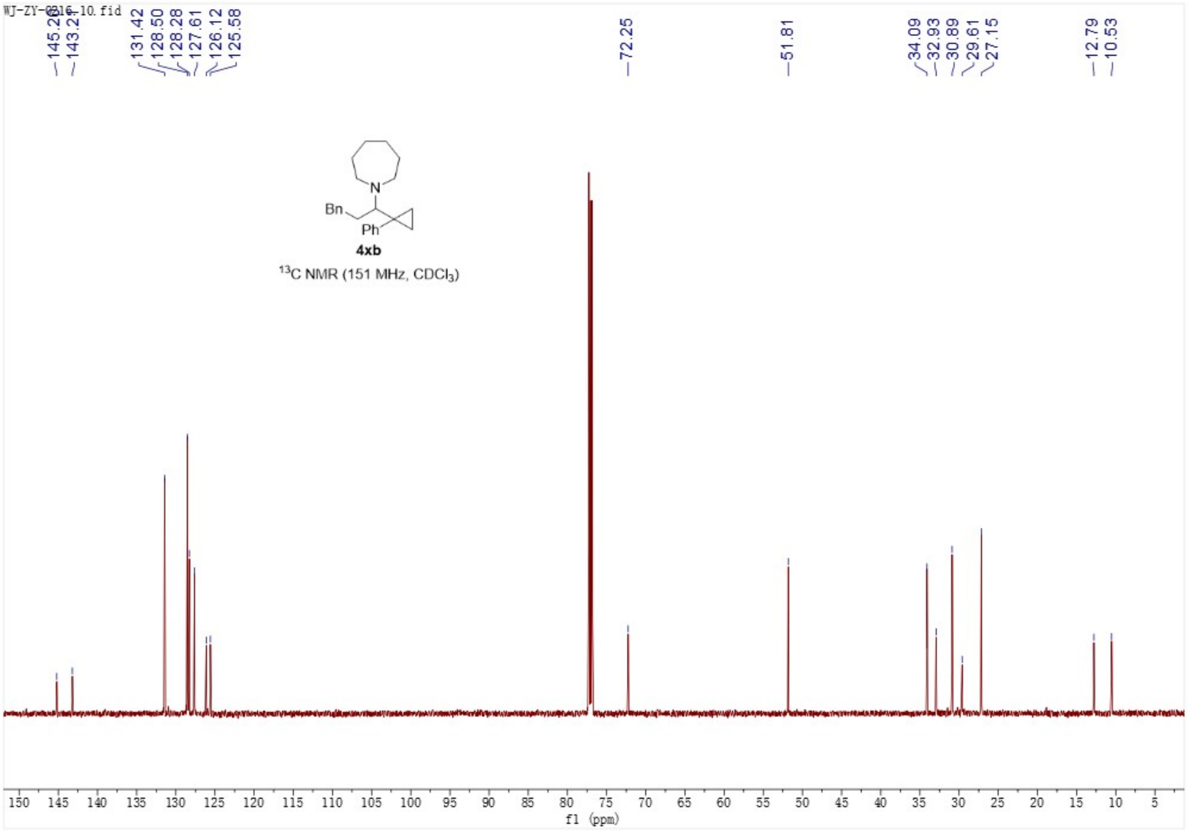
Bn
Ph
N
4xb
^{13}C NMR (151 MHz, $CDCl_3$)
145.26
143.2
131.42
128.50
128.28
127.61
126.12
125.58
72.25
51.81
34.09
32.93
30.89
29.61
27.15
12.79
10.53
f1 (ppm)

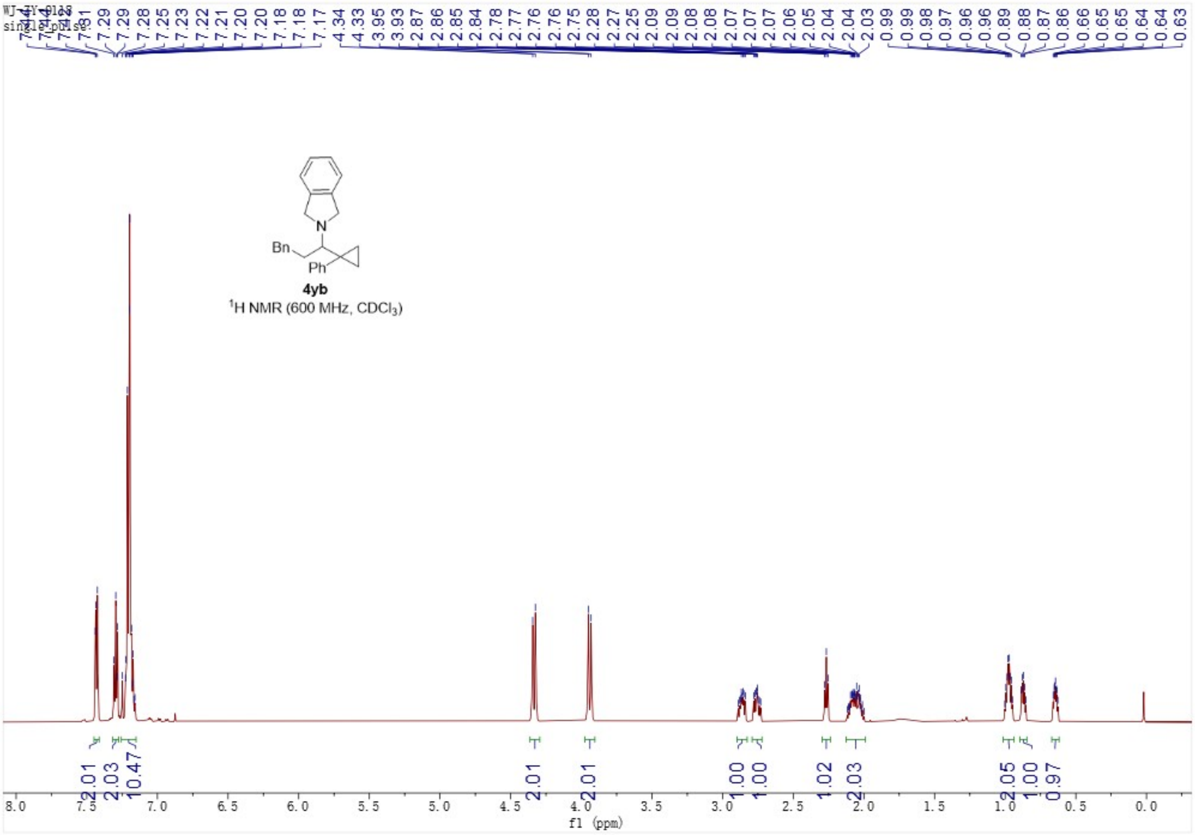

Bn
Ph
4yb
^{1}H NMR (600 MHz, CDCl$_3$)
2.01
2.03
10.47
2.01
2.01
1.00
1.00
1.02
2.03
2.05
1.00
0.97
f1 (ppm)

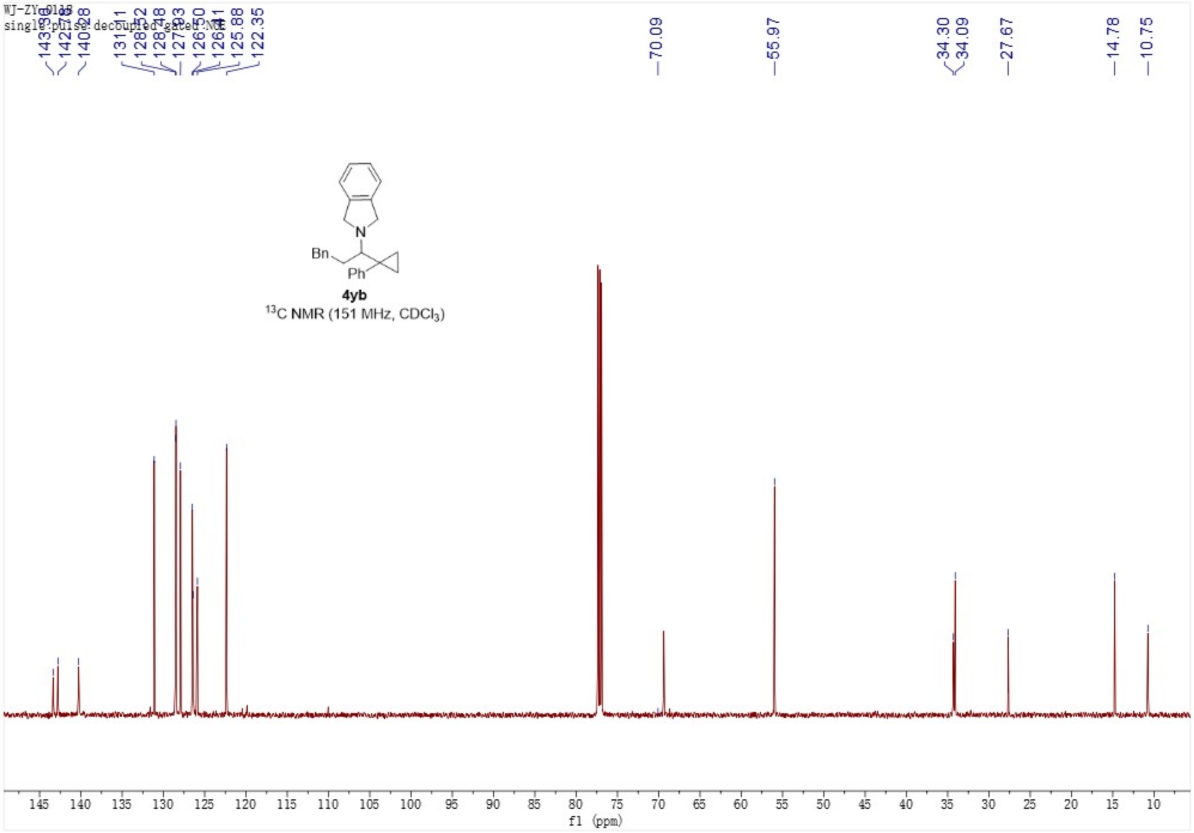

70.09
55.97
34.30
34.09
27.67
14.78
10.75
122.35
Bn
Ph
4yb
^{13}C NMR (151 MHz, CDCl$_3$)
f1 (ppm)

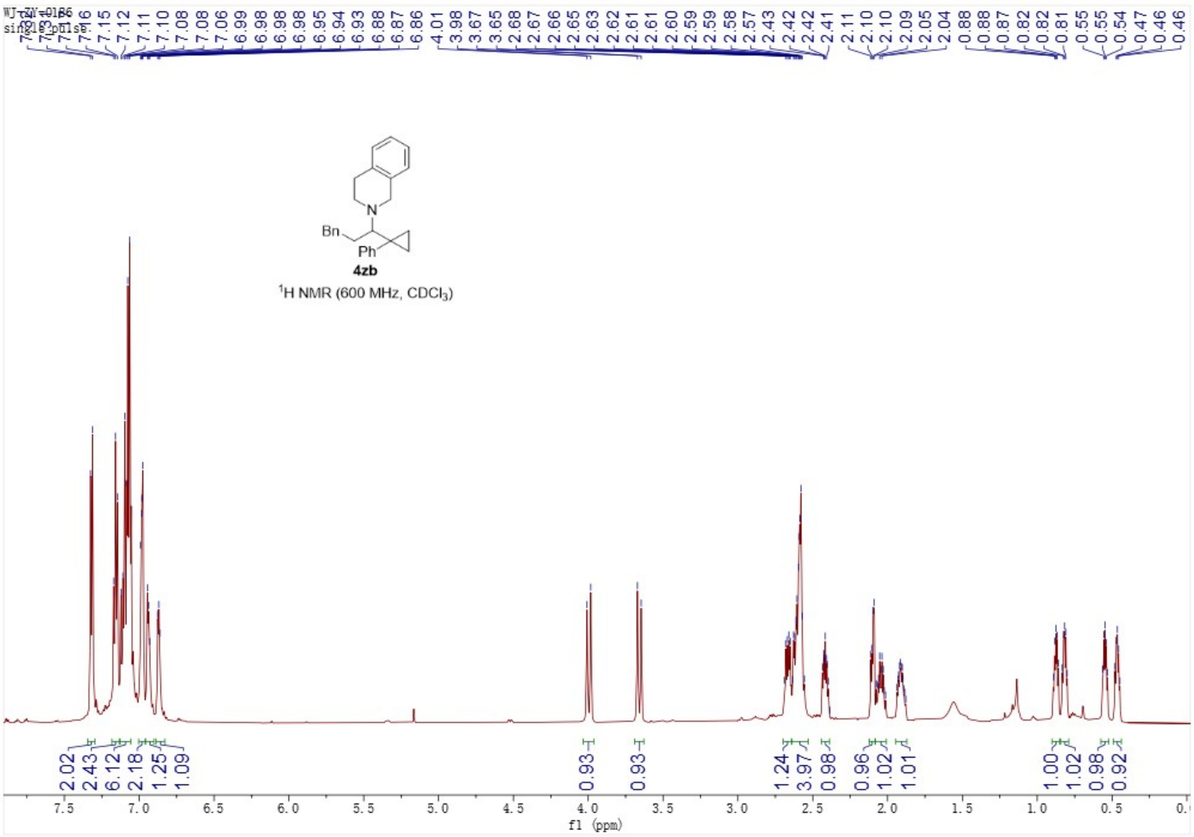
4zb
^{1}H NMR (600 MHz, CDCl$_3$)
f1 (ppm)

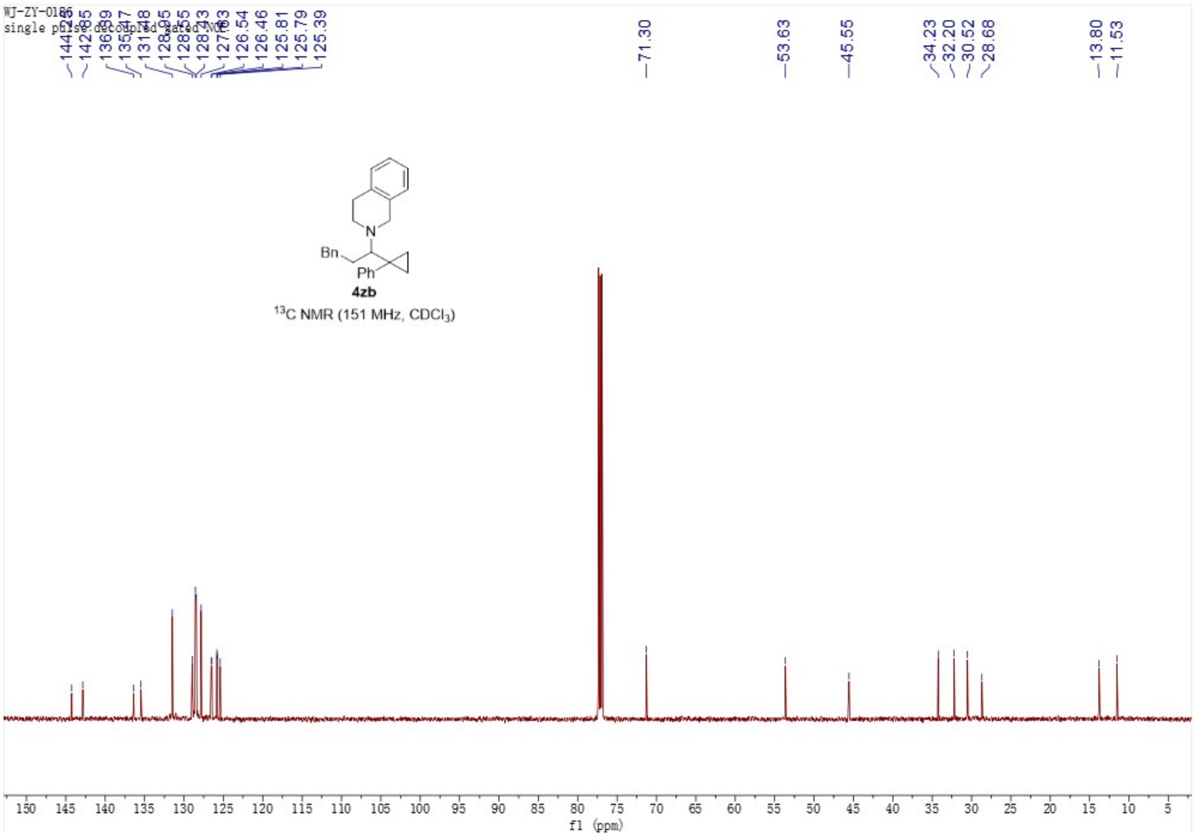
4zb
^{13}C NMR (151 MHz, CDCl$_3$)
f1 (ppm)

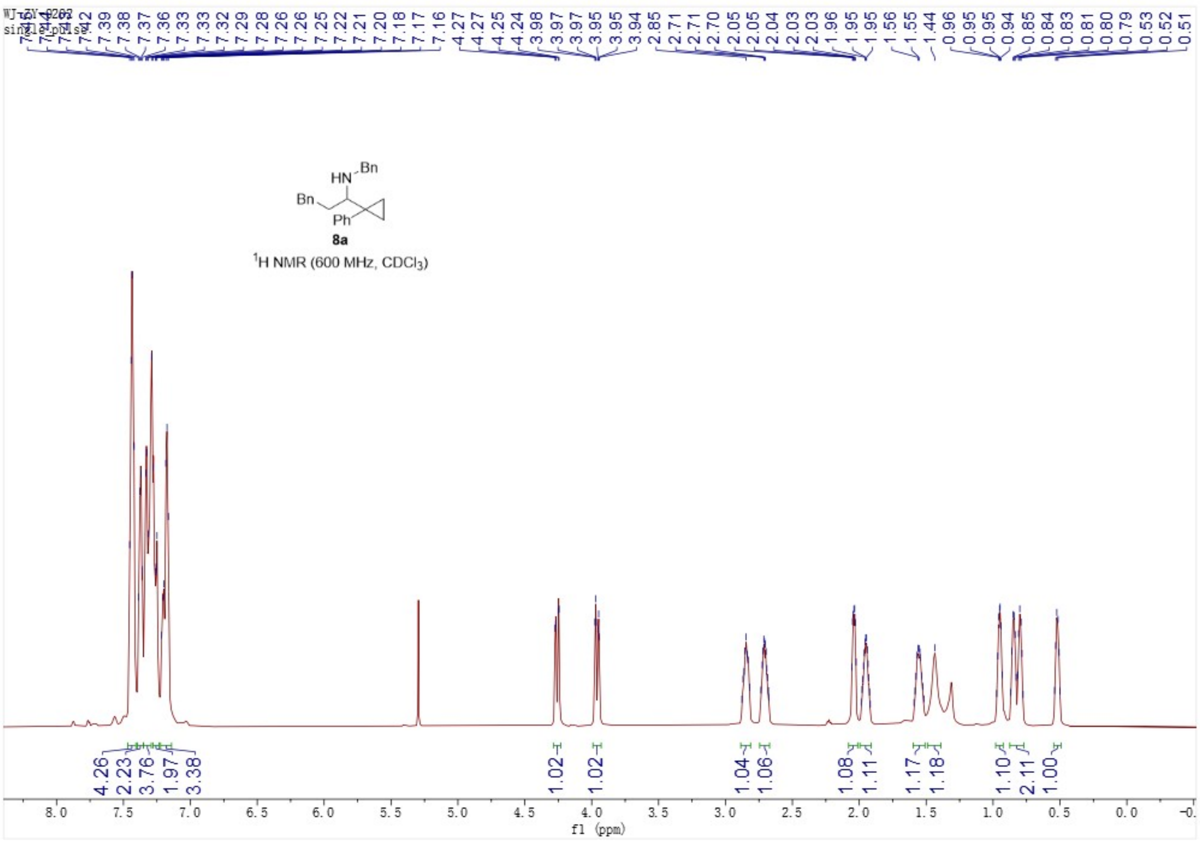
HN
Bn
Bn
Ph
8a
^1H NMR (600 MHz, $CDCl_3$)
f1 (ppm)

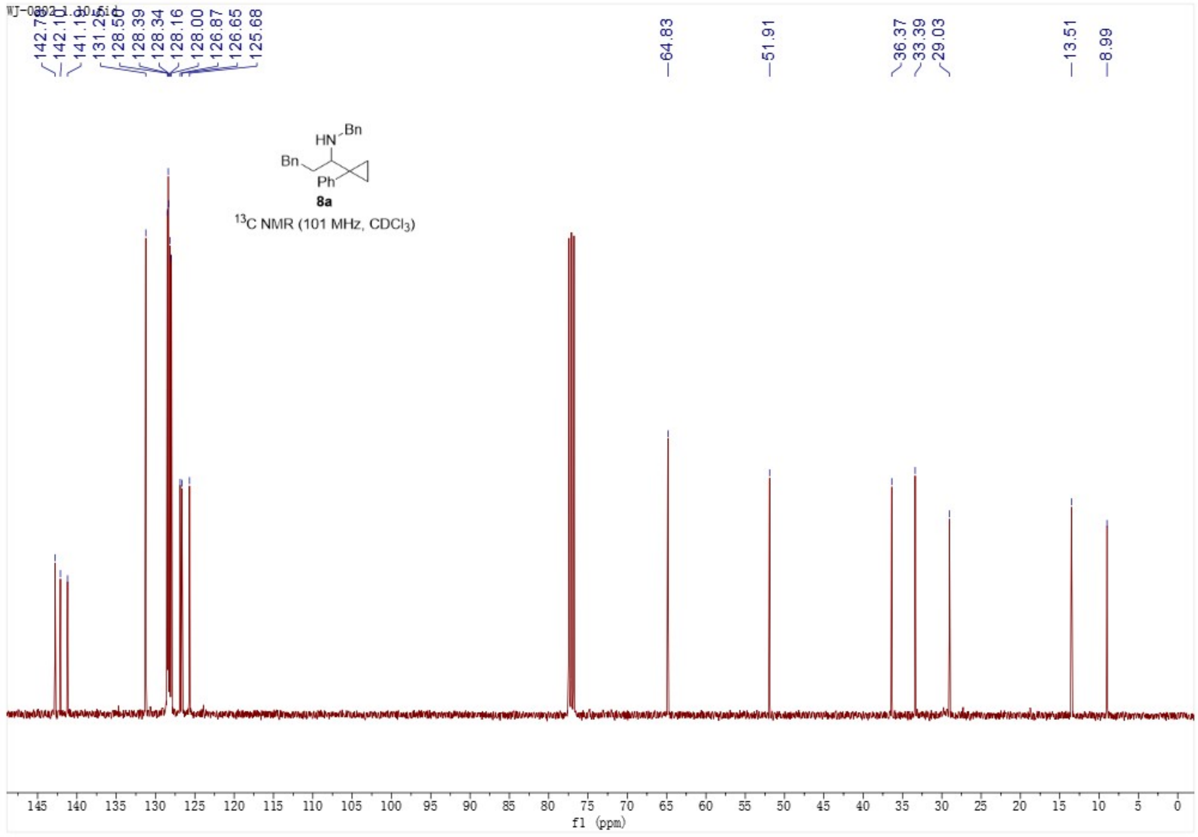
HN
Bn
Bn
Ph
8a
^{13}C NMR (101 MHz, $CDCl_3$)
f1 (ppm)

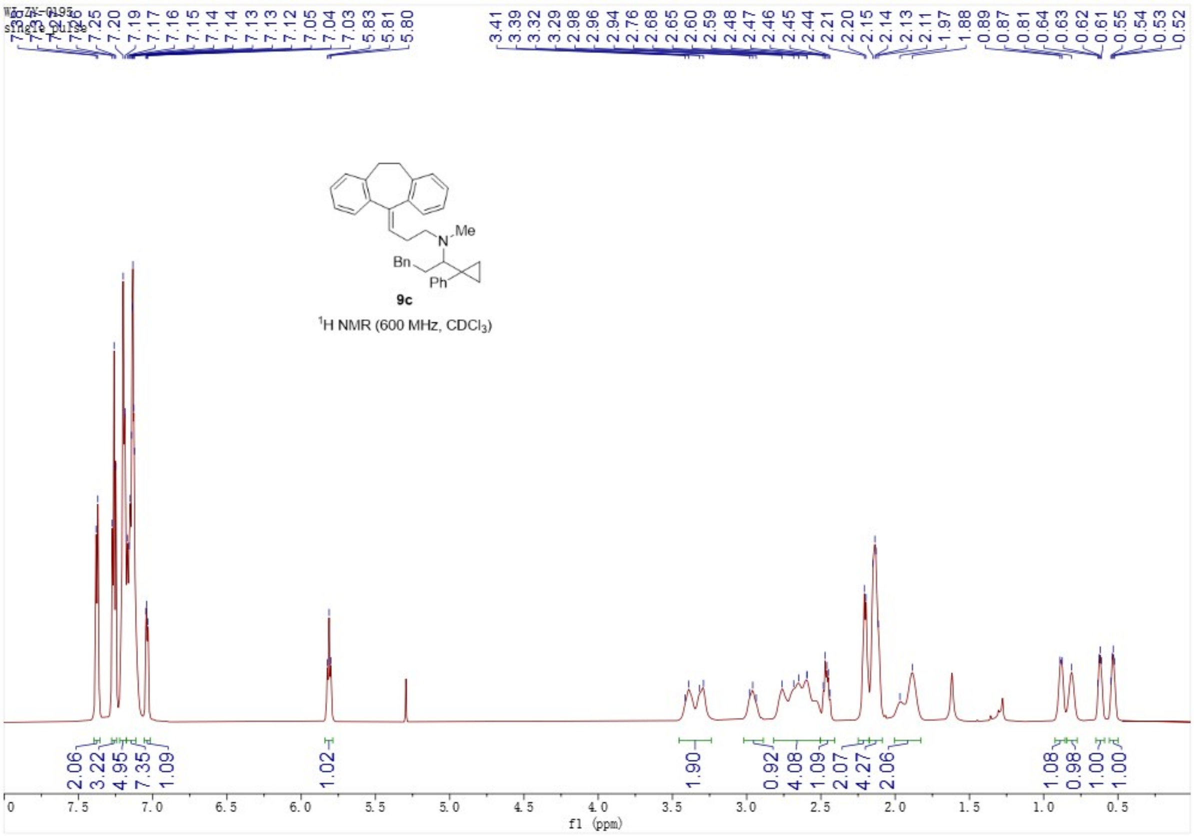
N
Me
Bn
Ph
9c
^{1}H NMR (600 MHz, CDCl$_3$)
7.25 7.20 7.19 7.17 7.16 7.15 7.14 7.13 7.13 7.12 7.05 7.04 7.03 5.83 5.81 5.80
3.41 3.39 3.32 3.29 2.98 2.96 2.94 2.76 2.68 2.65 2.60 2.59 2.48 2.47 2.46 2.45 2.44 2.21 2.20 2.15 2.14 2.13 2.11 1.97 1.88 0.89 0.87 0.81 0.64 0.63 0.62 0.61 0.55 0.54 0.53 0.52
2.06 3.22 4.95 7.35 1.09 1.02 1.90 0.92 4.08 1.09 2.07 4.27 2.06 1.08 0.98 1.00 1.00
f1 (ppm)

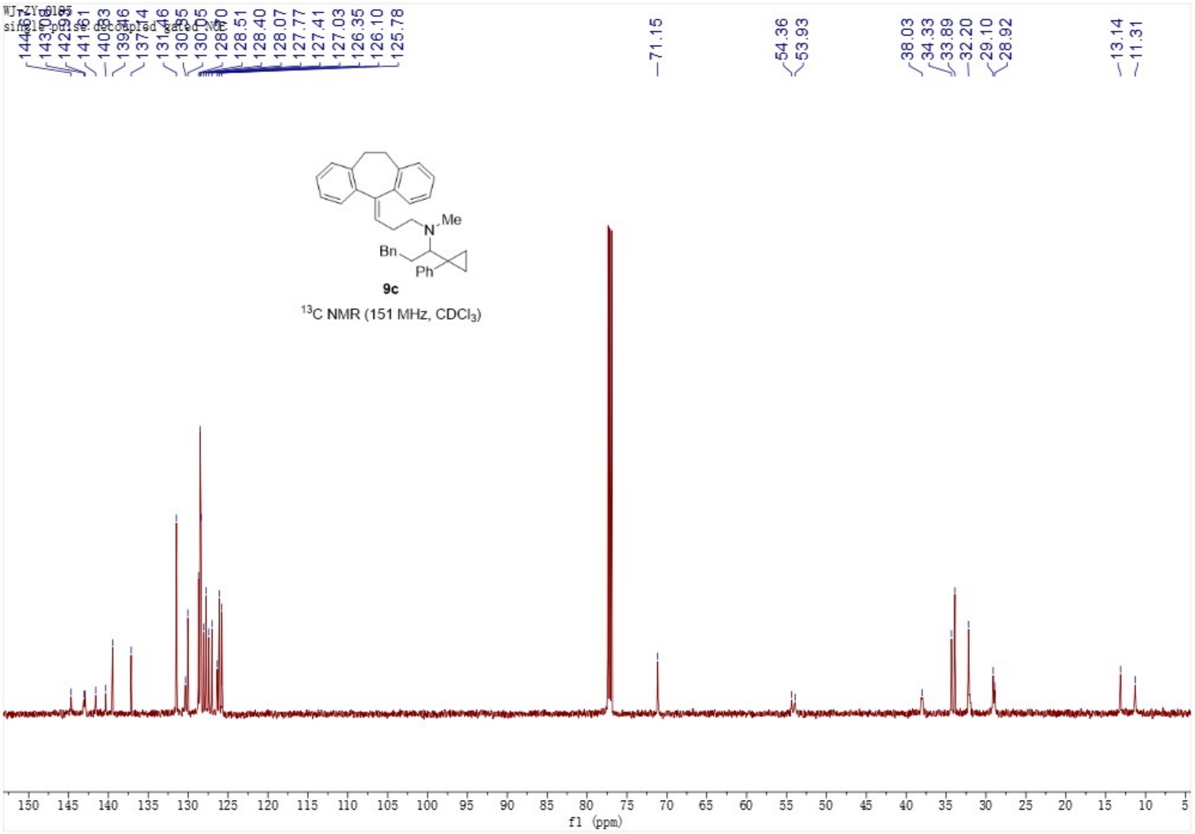
N
Me
Bn
Ph
9c
^{13}C NMR (151 MHz, CDCl$_3$)
128.51 128.40 128.07 127.77 127.41 127.03 126.35 126.10 125.78
71.15
54.36 53.93
38.03 34.33 33.89 32.20 29.10 28.92
13.14 11.31
f1 (ppm)

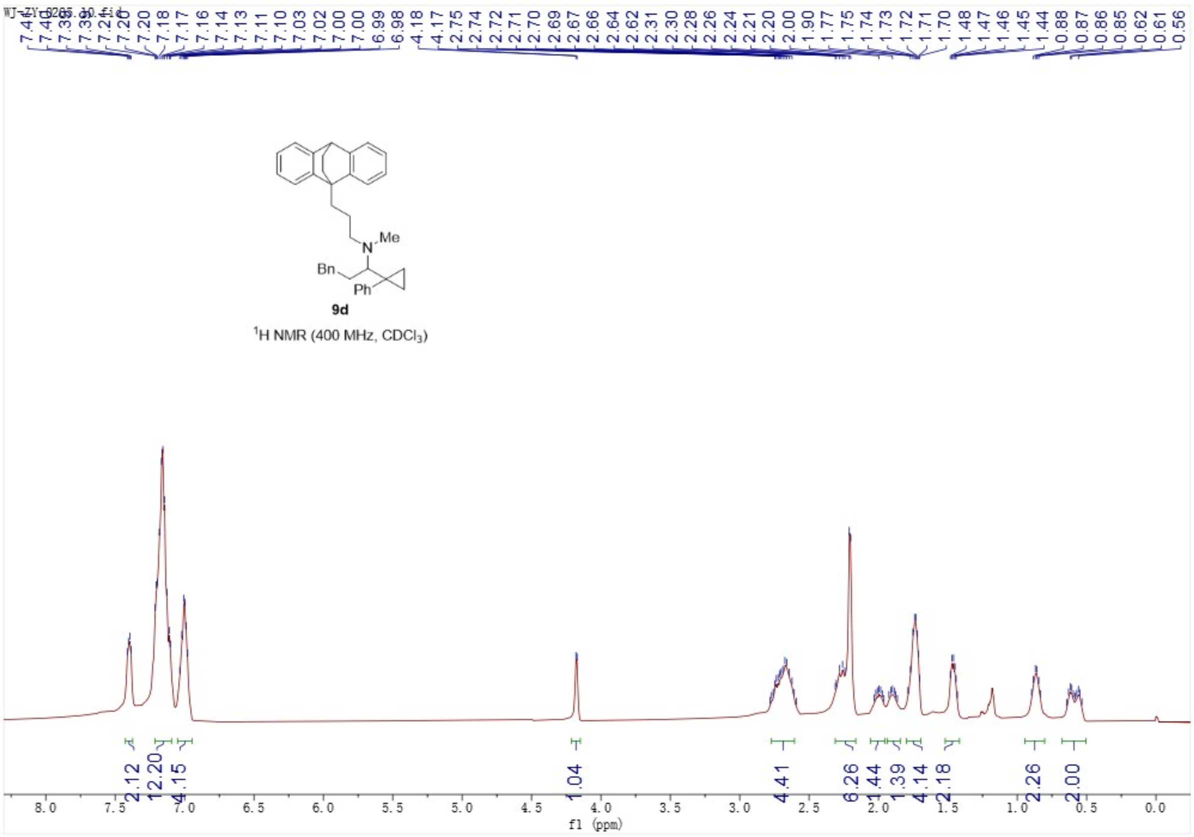

Me
N
Bn
Ph
9d
^{1}H NMR (400 MHz, CDCl$_3$)
f1 (ppm)

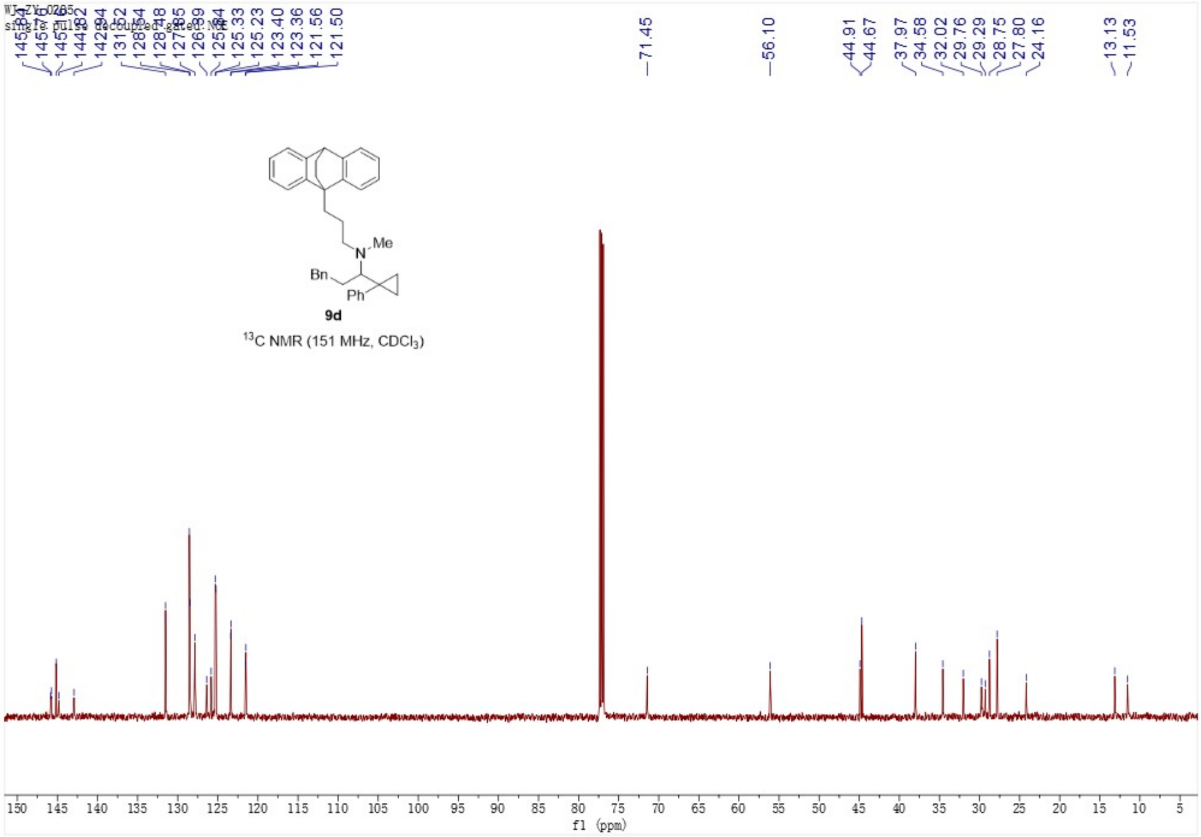

Me
N
Bn
Ph
9d
^{13}C NMR (151 MHz, CDCl$_3$)
f1 (ppm)

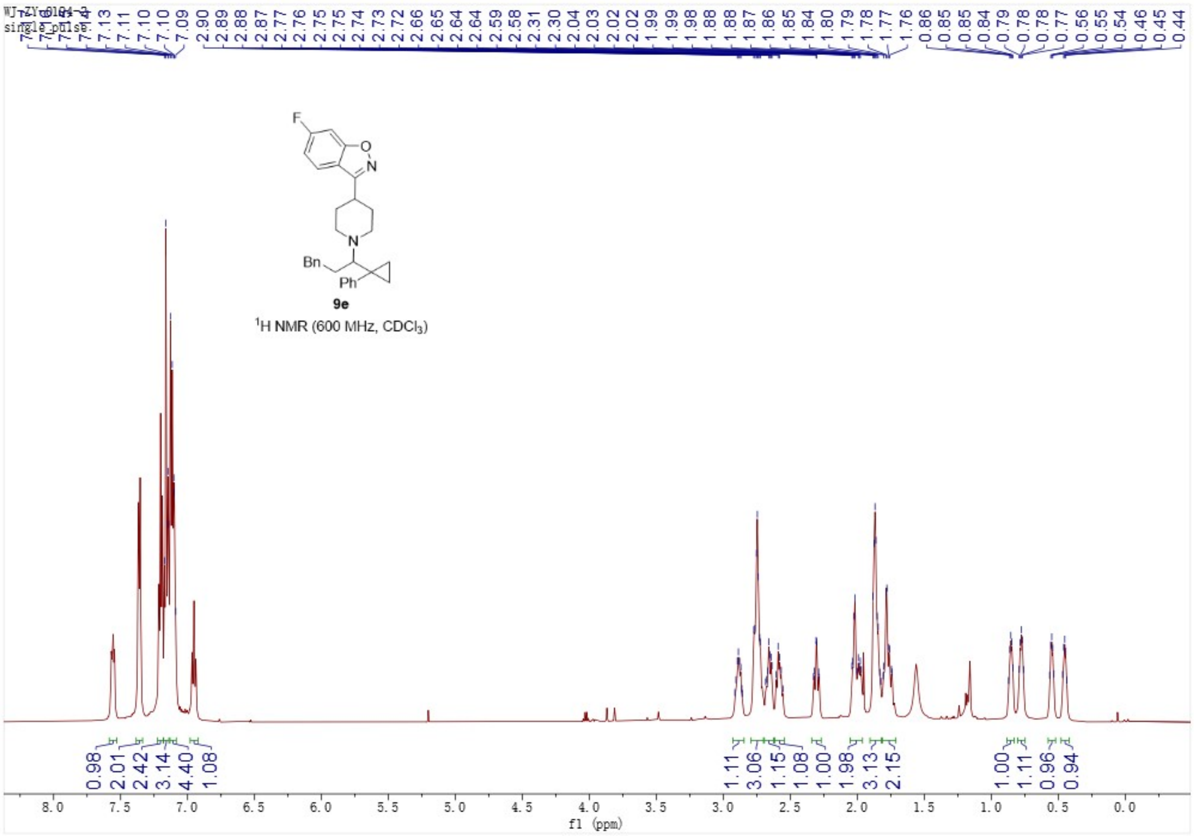

9e

^{1}H NMR (600 MHz, $CDCl_3$)

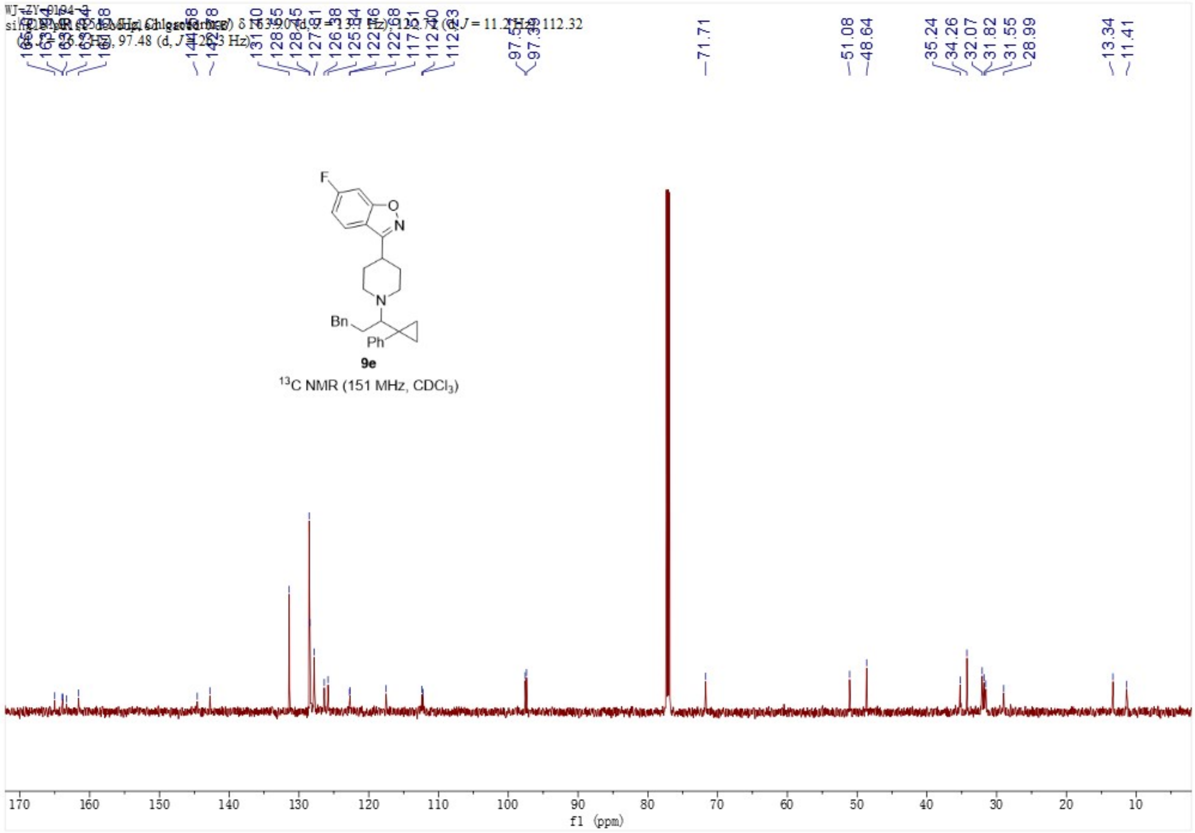

9e

^{13}C NMR (151 MHz, $CDCl_3$)

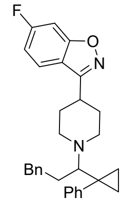

9e

^{19}F NMR (376 MHz, $CDCl_3$)

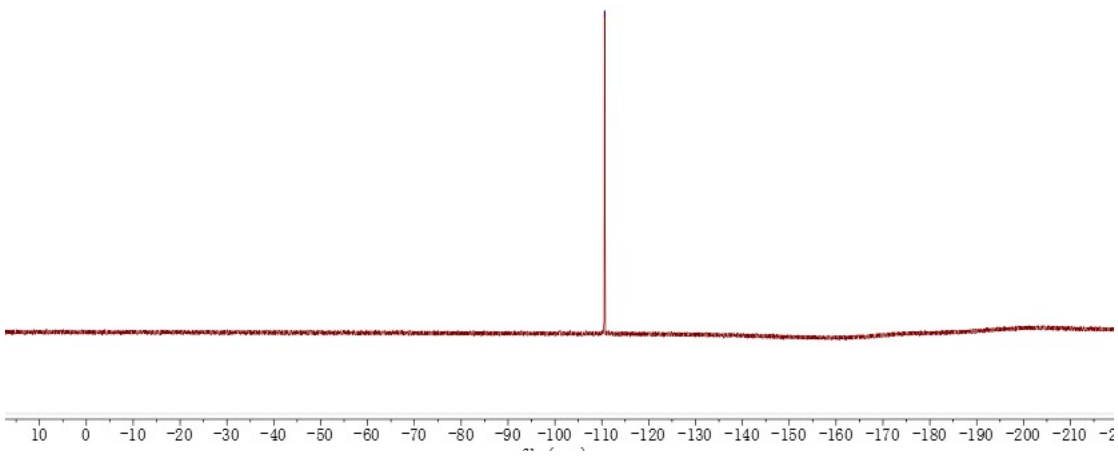

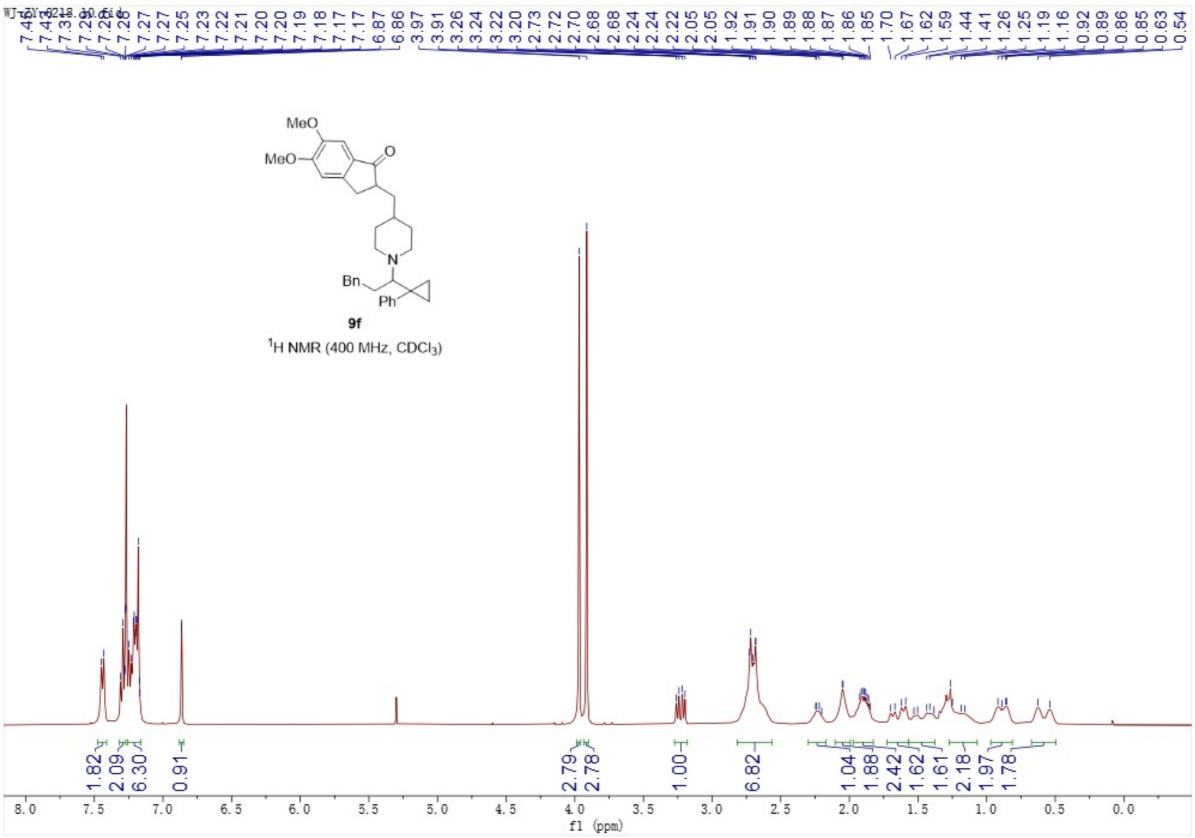

1H NMR (400 MHz, CDCl3)
9f

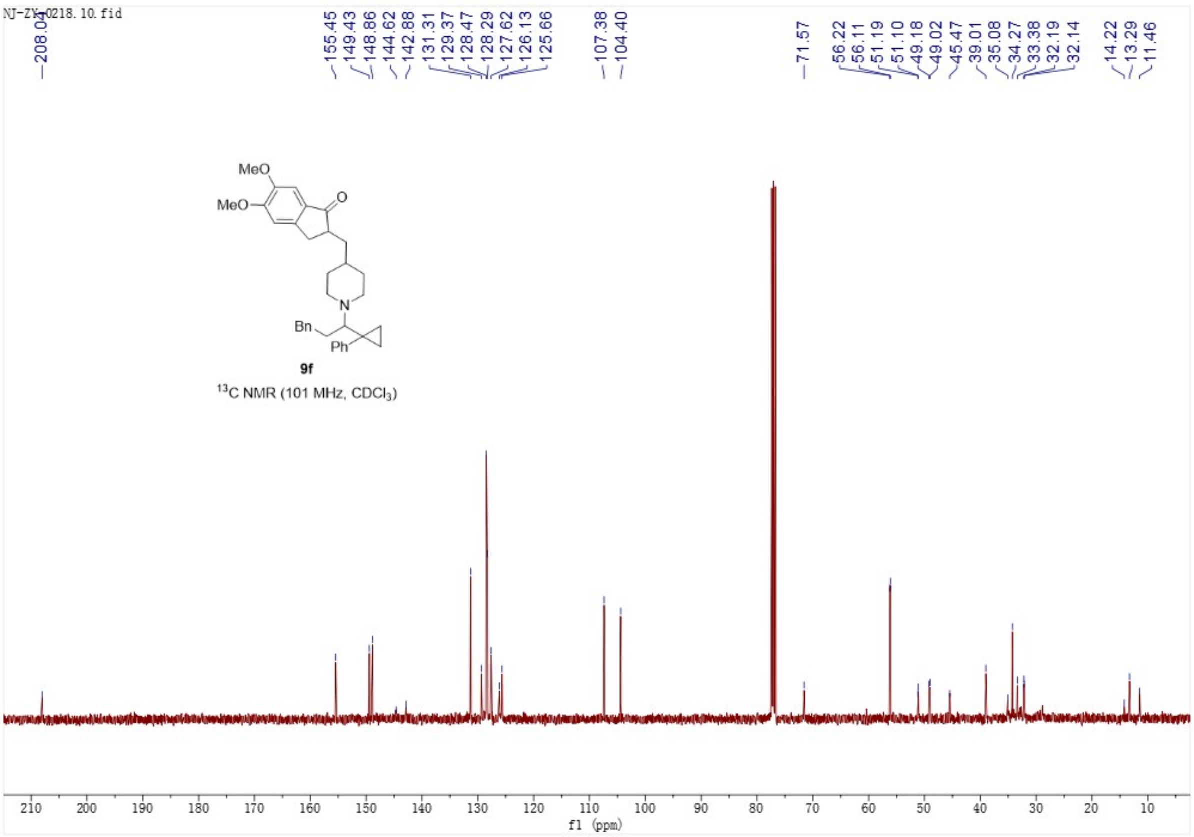

13C NMR (101 MHz, CDCl3)
9f

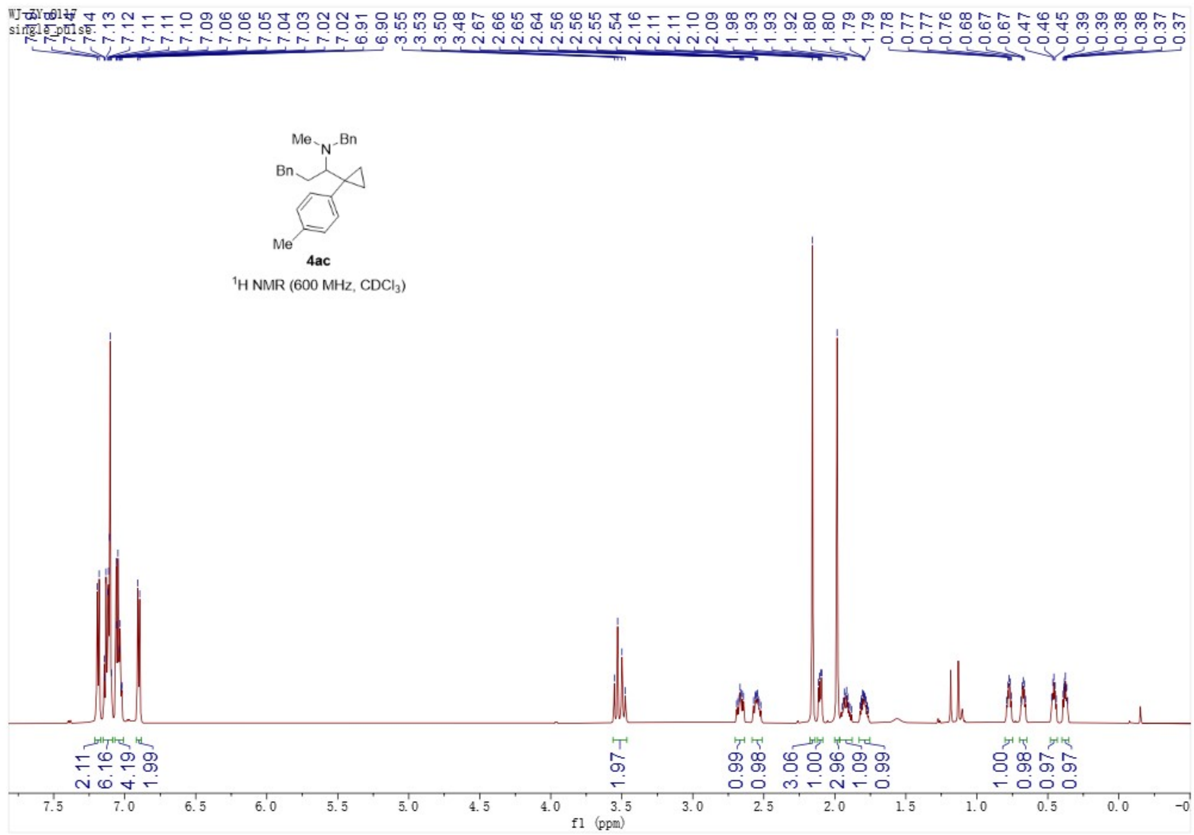
WJ-ZY-0117
single_pulse
Me
N
Bn
Bn
Me
4ac
1H NMR (600 MHz, CDCl3)
f1 (ppm)

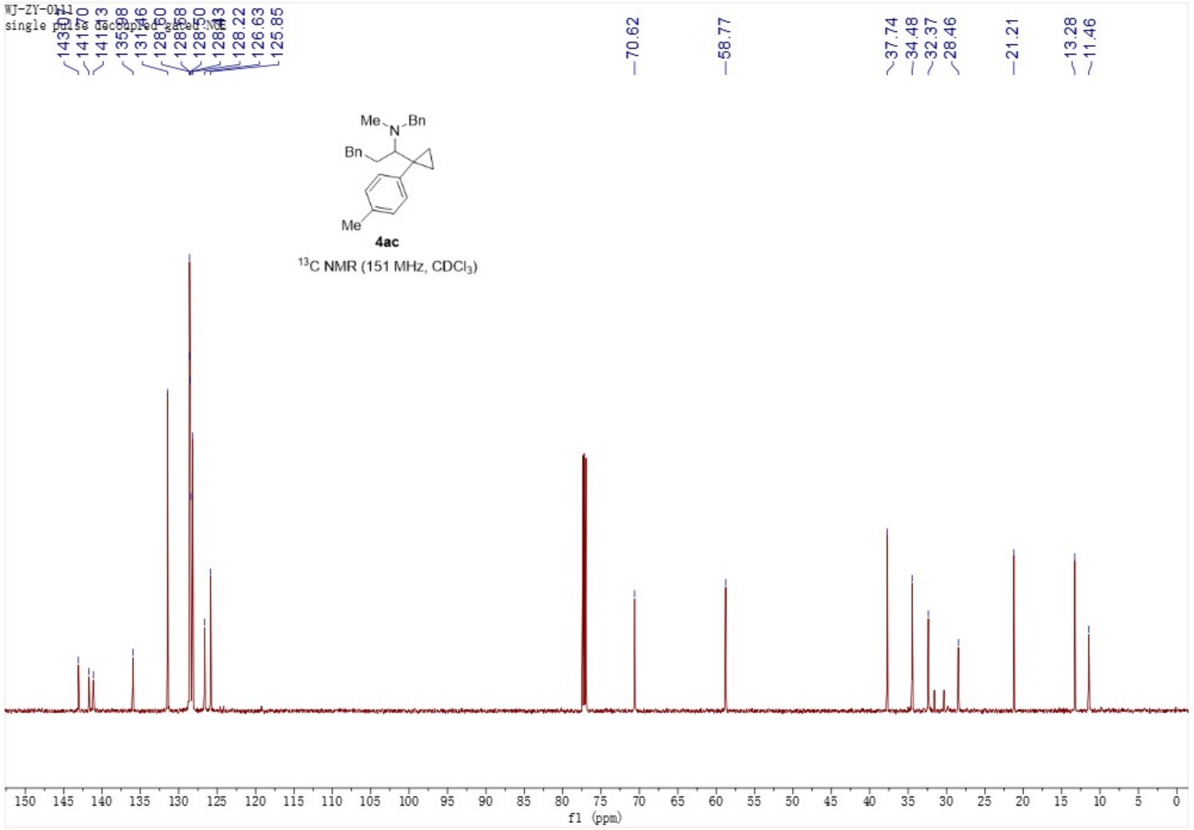
WJ-ZY-0111
single pulse decoupled gated NOE
70.62
58.77
37.74
34.48
32.37
28.46
21.21
13.28
11.46
Me
N
Bn
Bn
Me
4ac
13C NMR (151 MHz, CDCl3)
f1 (ppm)

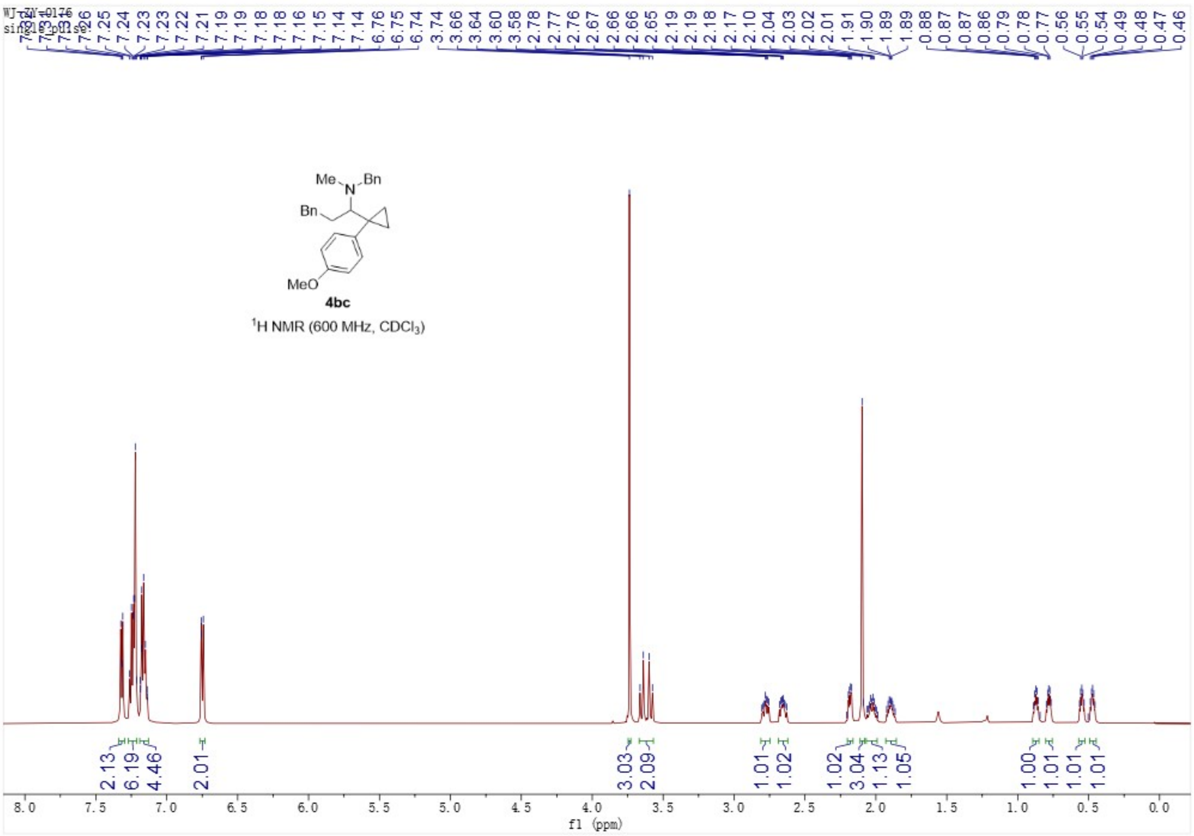
4bc
^{1}H NMR (600 MHz, CDCl$_3$)

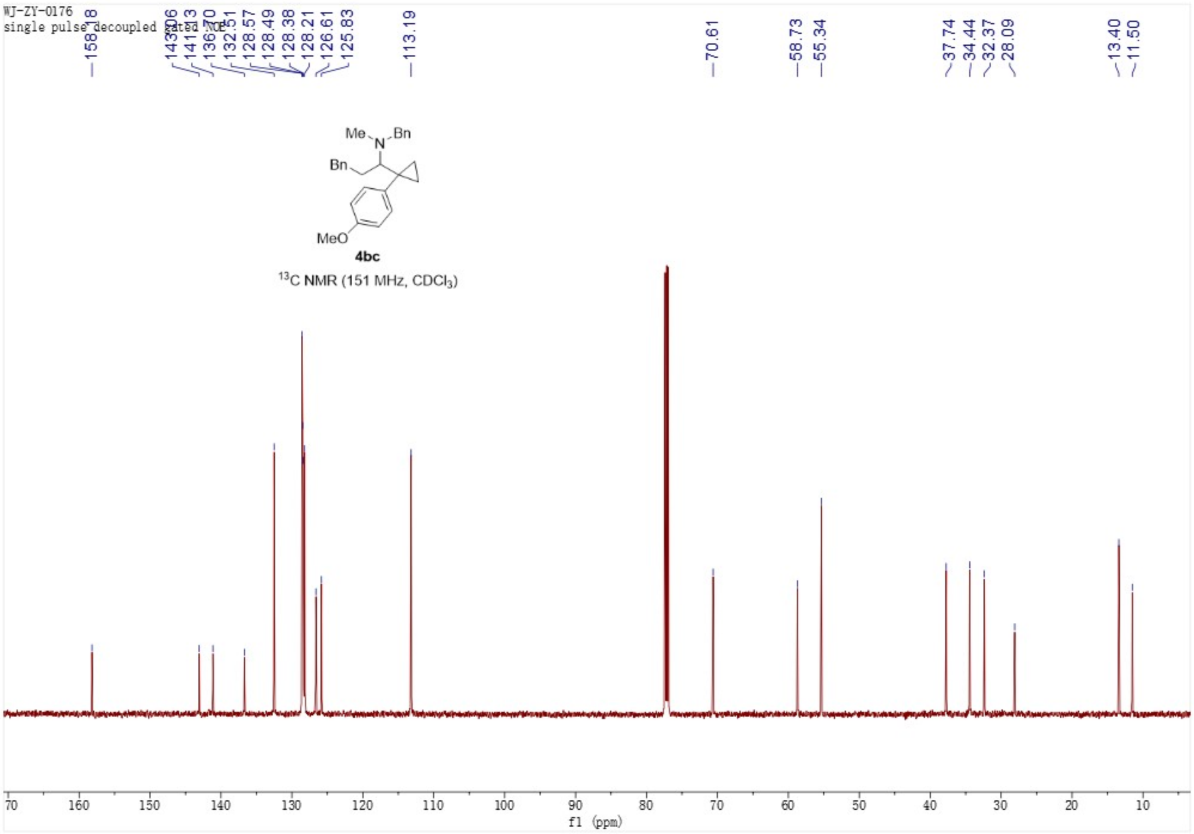
4bc
^{13}C NMR (151 MHz, CDCl$_3$)

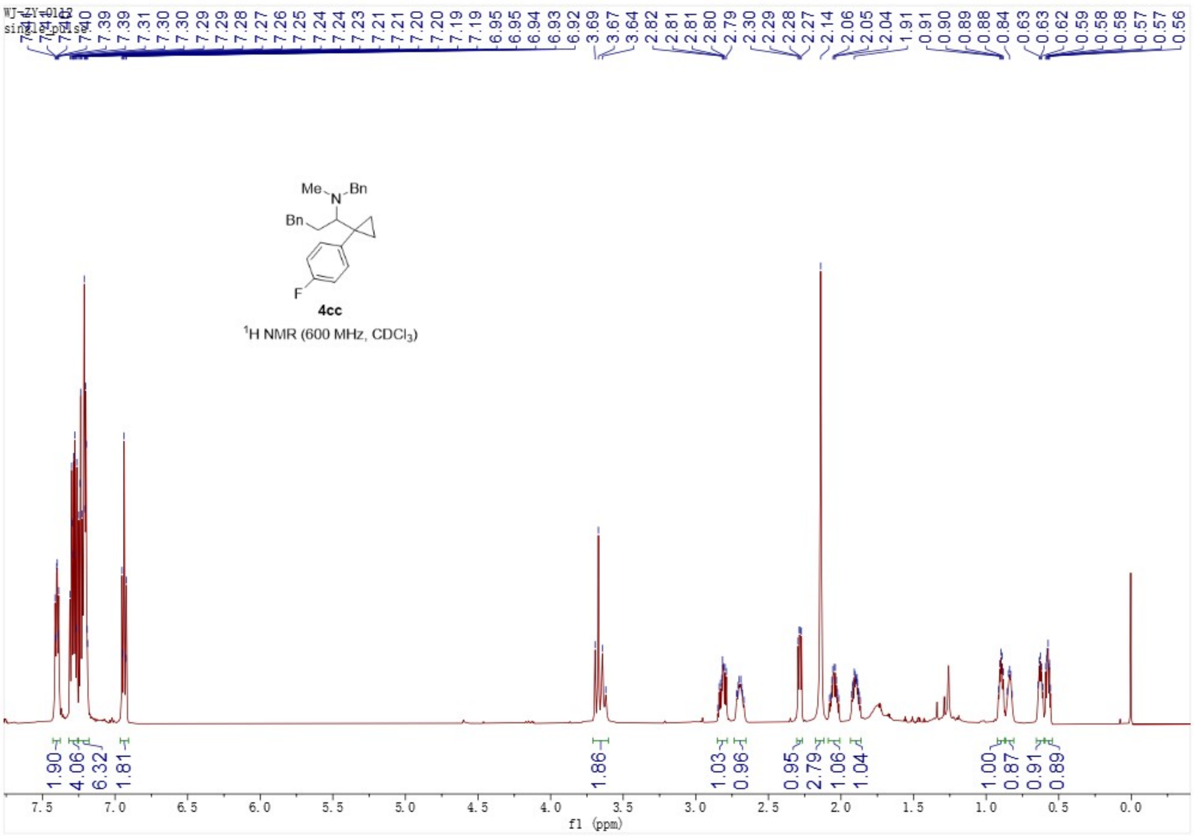

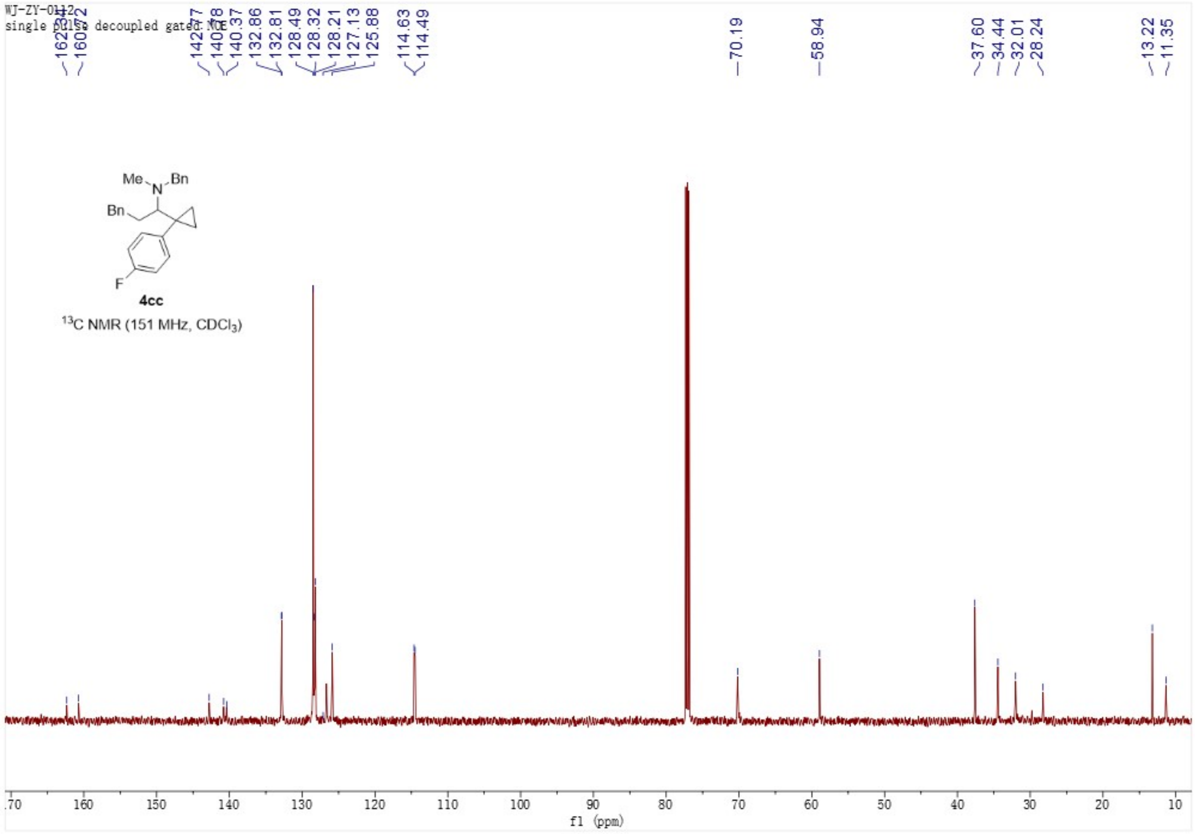

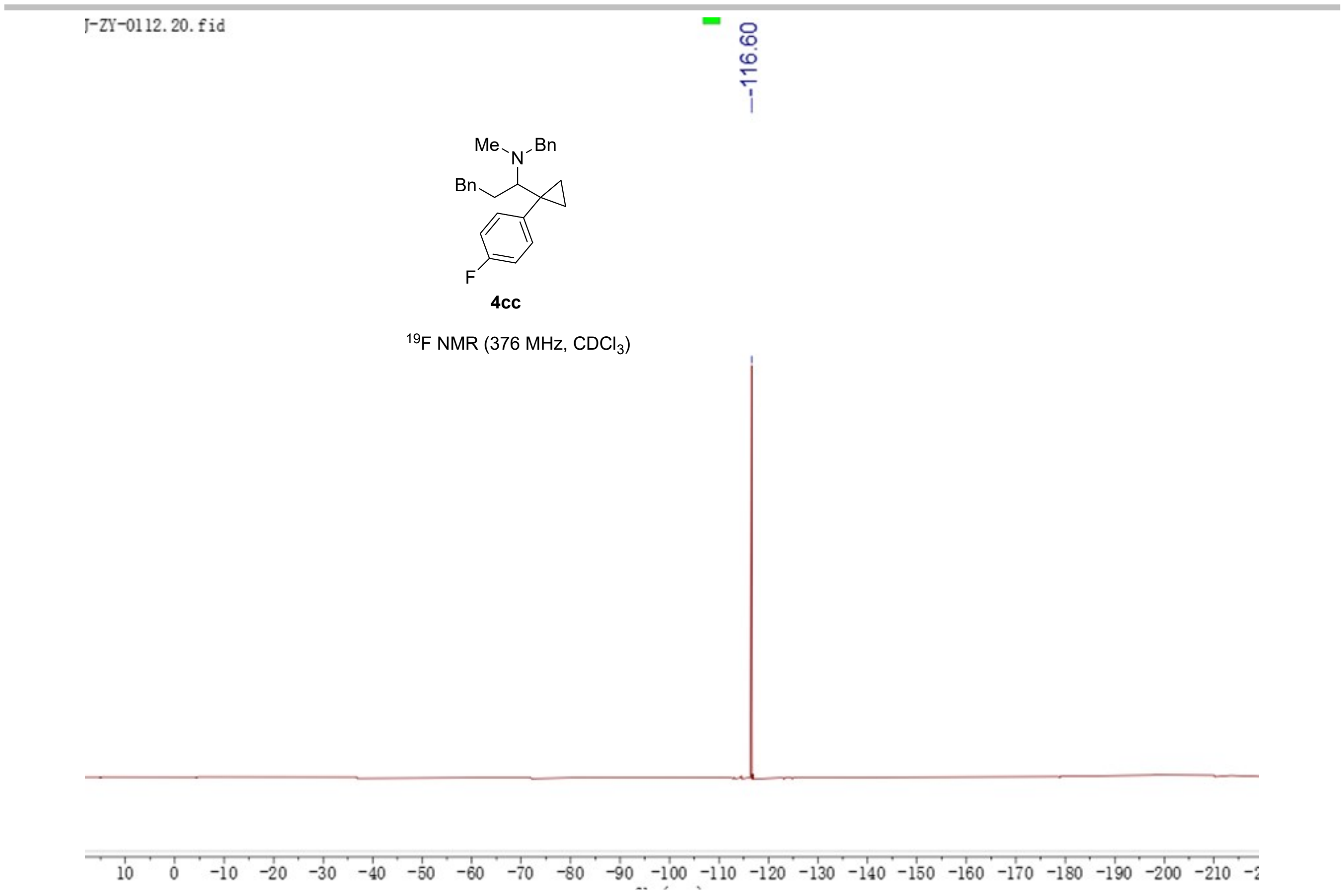

4cc

^{19}F NMR (376 MHz, $CDCl_3$)

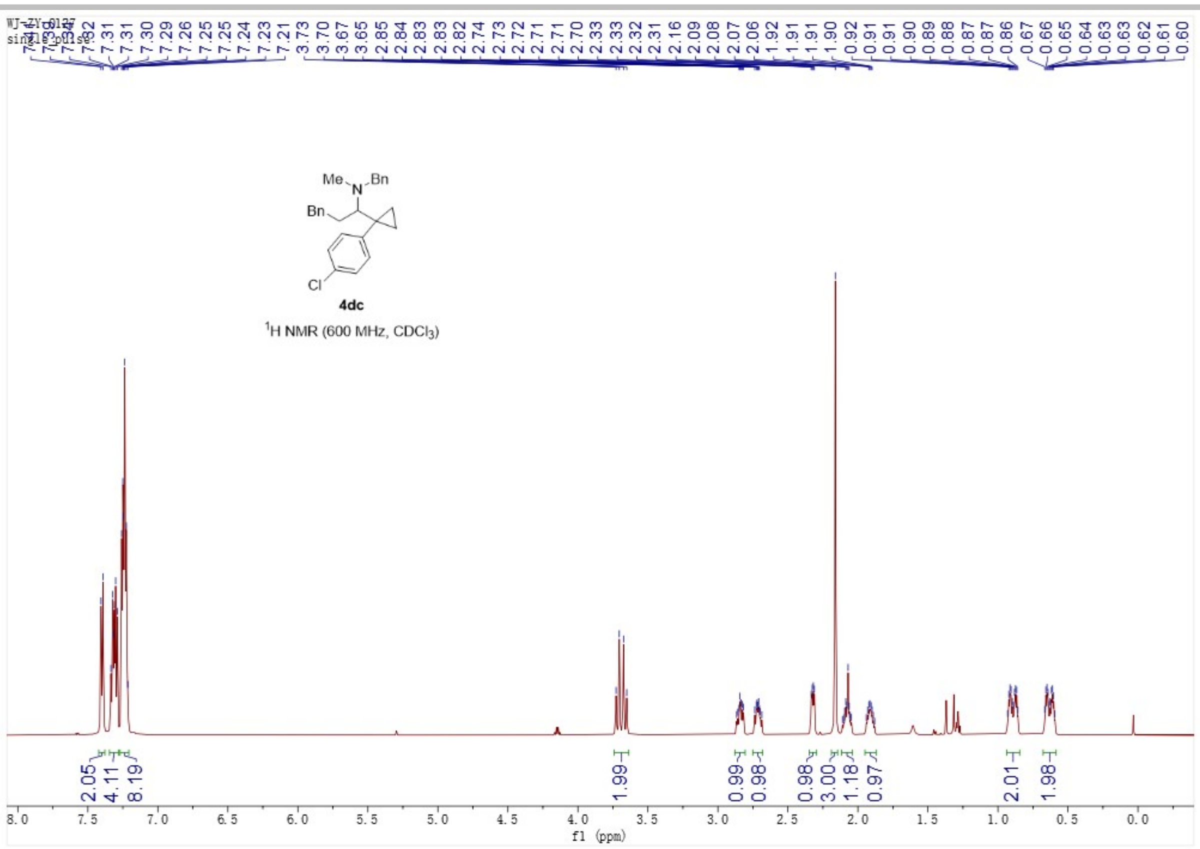
Me
N
Bn
Bn
Cl
4dc
^{1}H NMR (600 MHz, CDCl$_3$)
f1 (ppm)

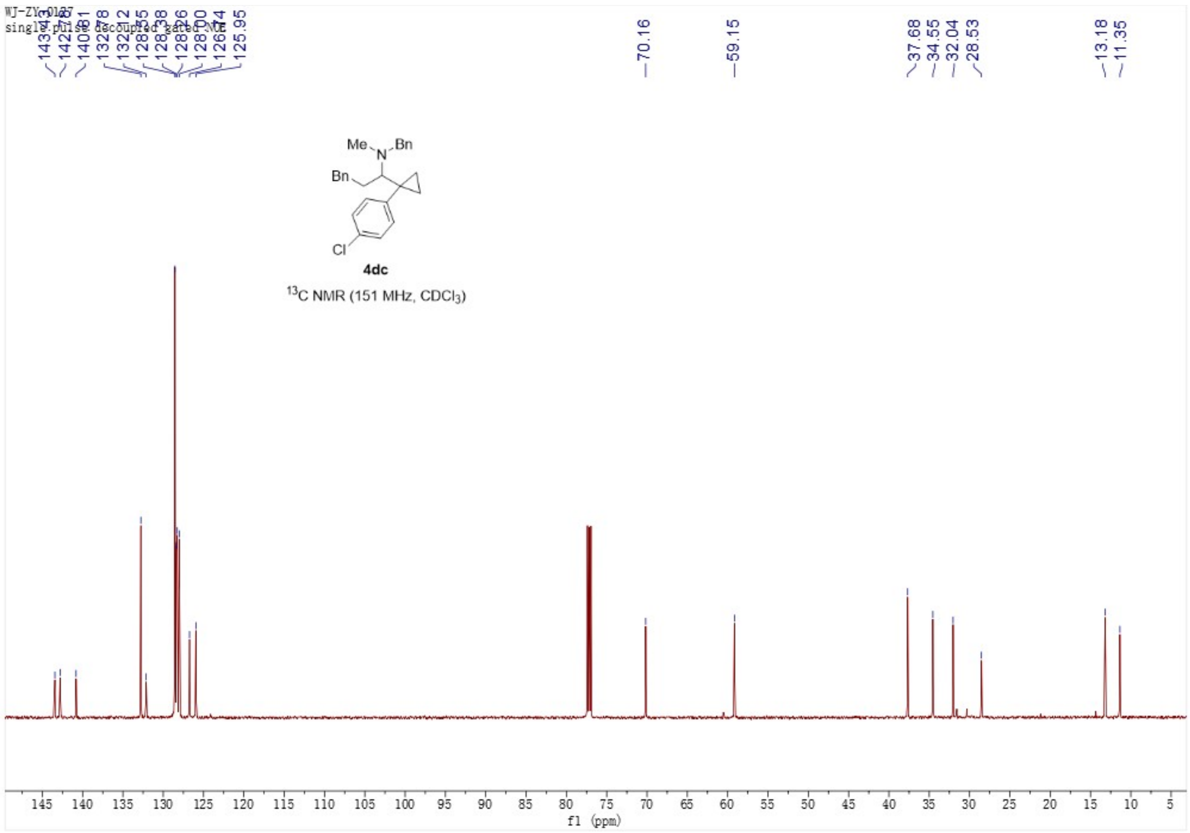
Me
N
Bn
Bn
Cl
4dc
^{13}C NMR (151 MHz, CDCl$_3$)
70.16
59.15
37.68
34.55
32.04
28.53
13.18
11.35
125.95
f1 (ppm)

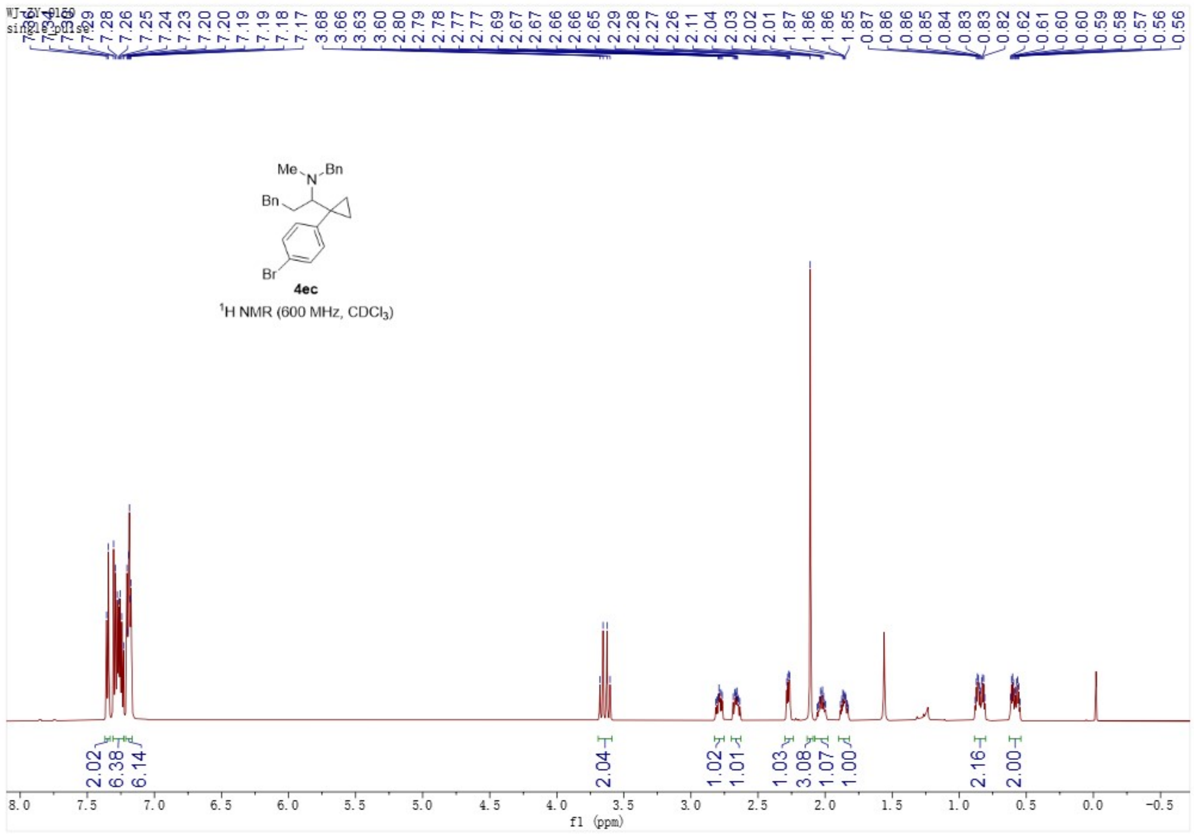
4ec
^{1}H NMR (600 MHz, CDCl$_3$)

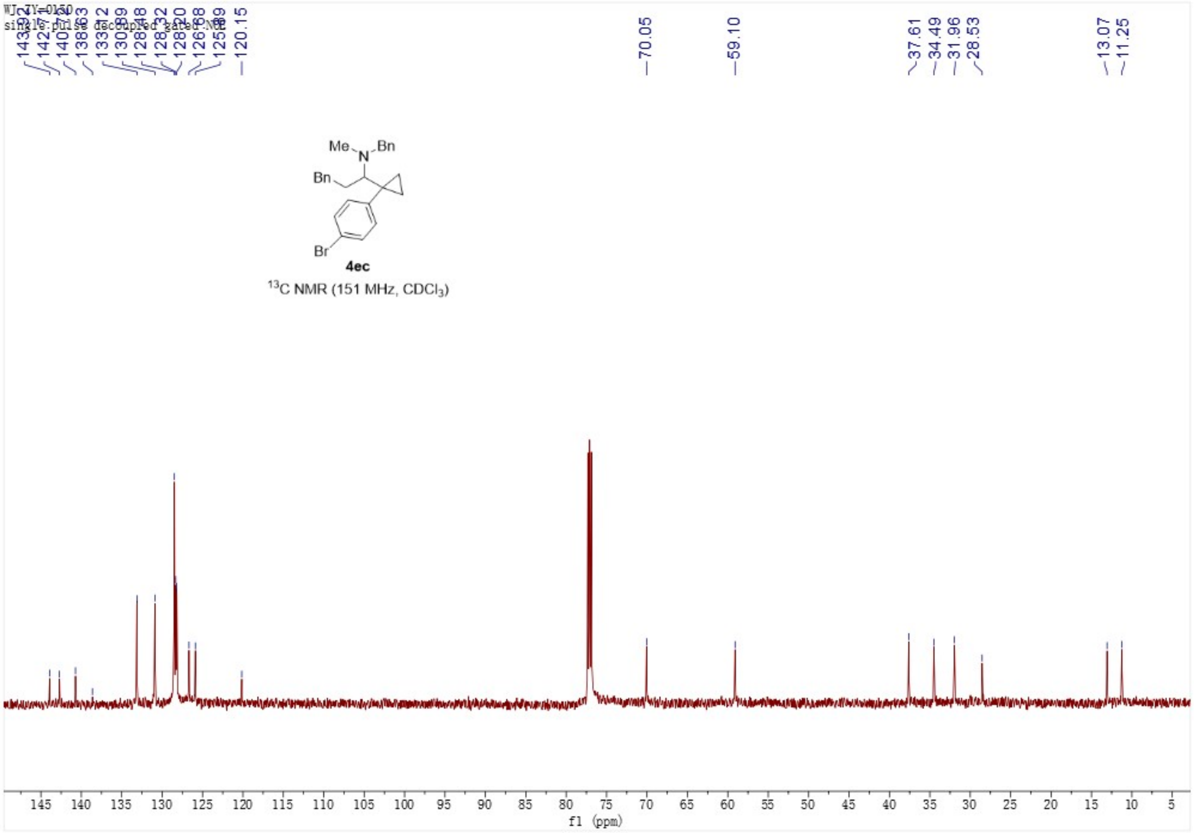
4ec
^{13}C NMR (151 MHz, CDCl$_3$)

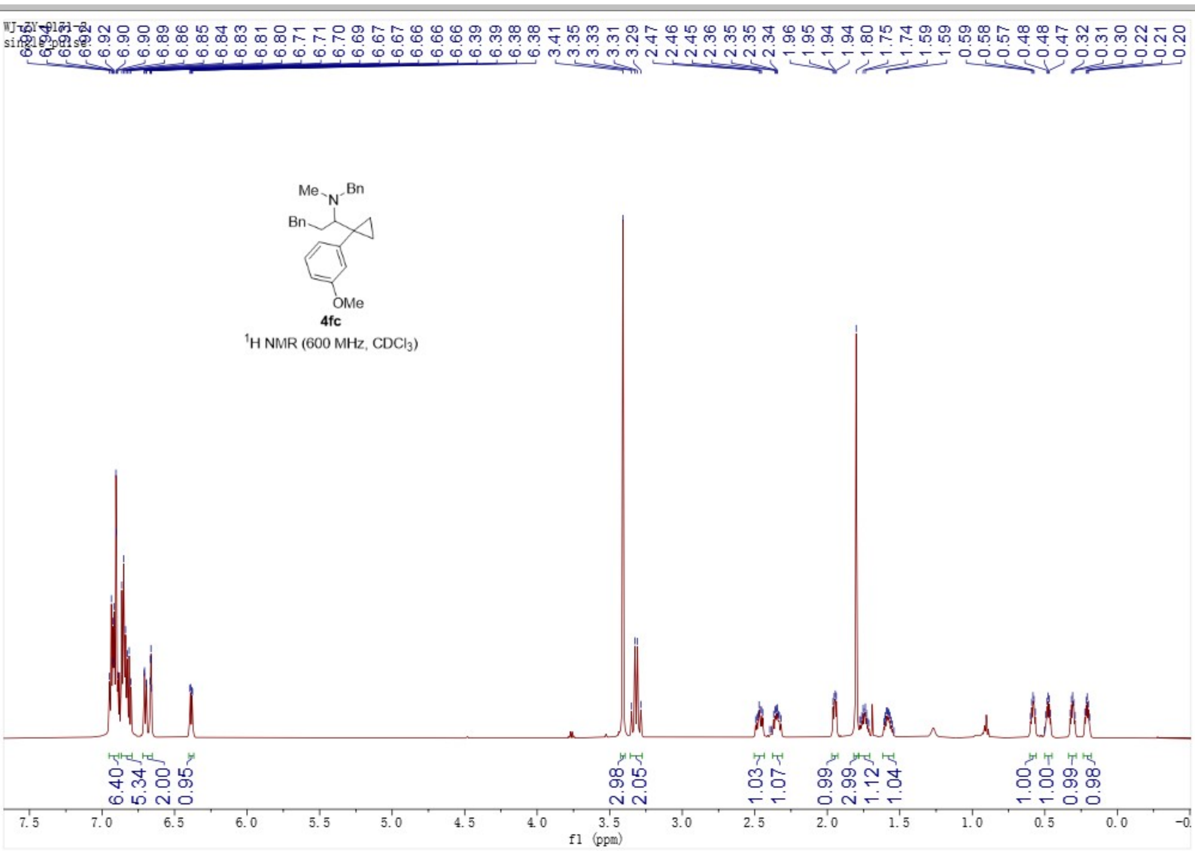
WJ-ZY-01[illegible]
single pulse
6.92
6.90
6.89
6.86
6.85
6.84
6.83
6.81
6.80
6.71
6.70
6.69
6.67
6.66
6.66
6.66
6.39
6.39
6.38
6.38
3.41
3.35
3.33
3.31
3.29
2.47
2.46
2.45
2.36
2.35
2.35
2.34
1.96
1.95
1.94
1.94
1.80
1.75
1.74
1.59
1.59
0.59
0.58
0.57
0.48
0.48
0.47
0.32
0.31
0.30
0.22
0.21
0.20
Me
N
Bn
Bn
OMe
4fc
1H NMR (600 MHz, CDCl3)
6.40
5.34
2.00
0.95
2.98
2.05
1.03
1.07
0.99
2.99
1.12
1.04
1.00
1.00
0.99
0.98
7.5
7.0
6.5
6.0
5.5
5.0
4.5
4.0
3.5
3.0
2.5
2.0
1.5
1.0
0.5
0.0
f1 (ppm)

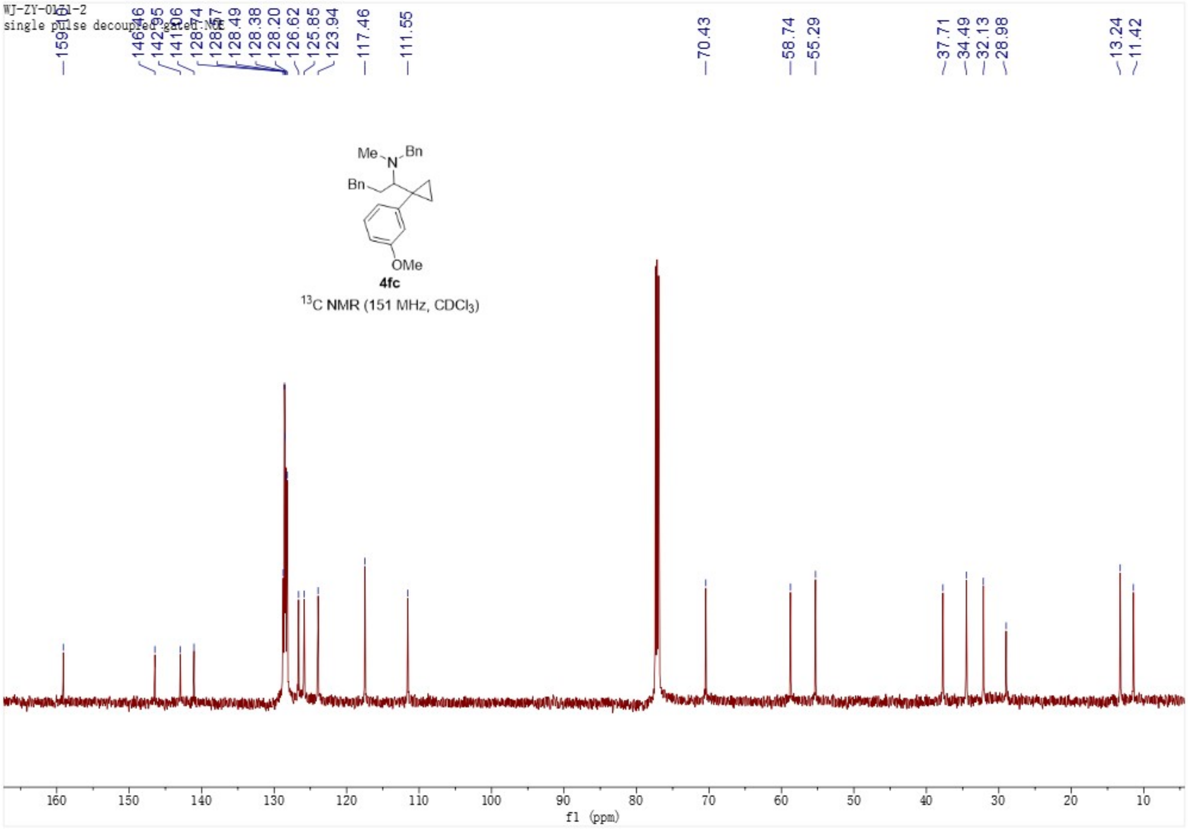
WJ-ZY-01[illegible]-2
single pulse decoupled gated NOE
142.95
141.06
128.57
128.49
128.38
128.20
126.62
125.85
123.94
117.46
111.55
70.43
58.74
55.29
37.71
34.49
32.13
28.98
13.24
11.42
Me
N
Bn
Bn
OMe
4fc
13C NMR (151 MHz, CDCl3)
160
150
140
130
120
110
100
90
80
70
60
50
40
30
20
10
f1 (ppm)

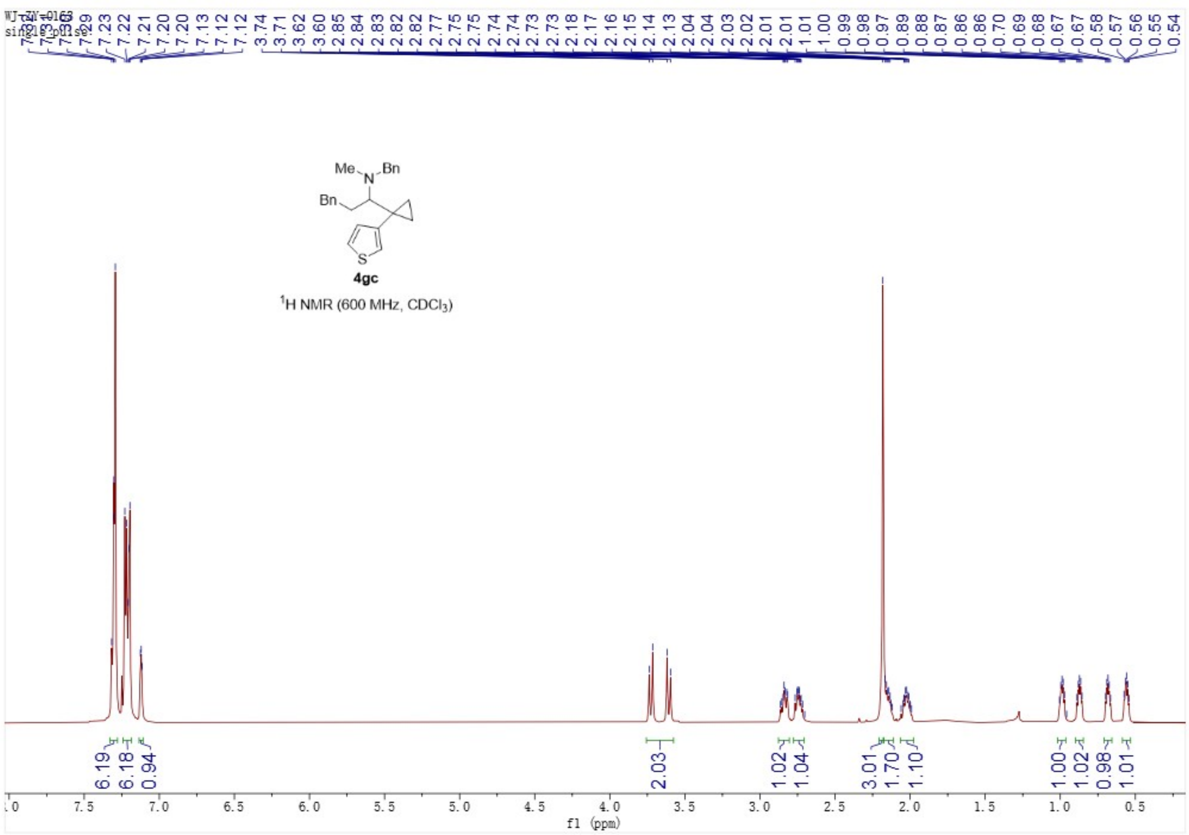
4gc
^{1}H NMR (600 MHz, CDCl$_3$)
f1 (ppm)

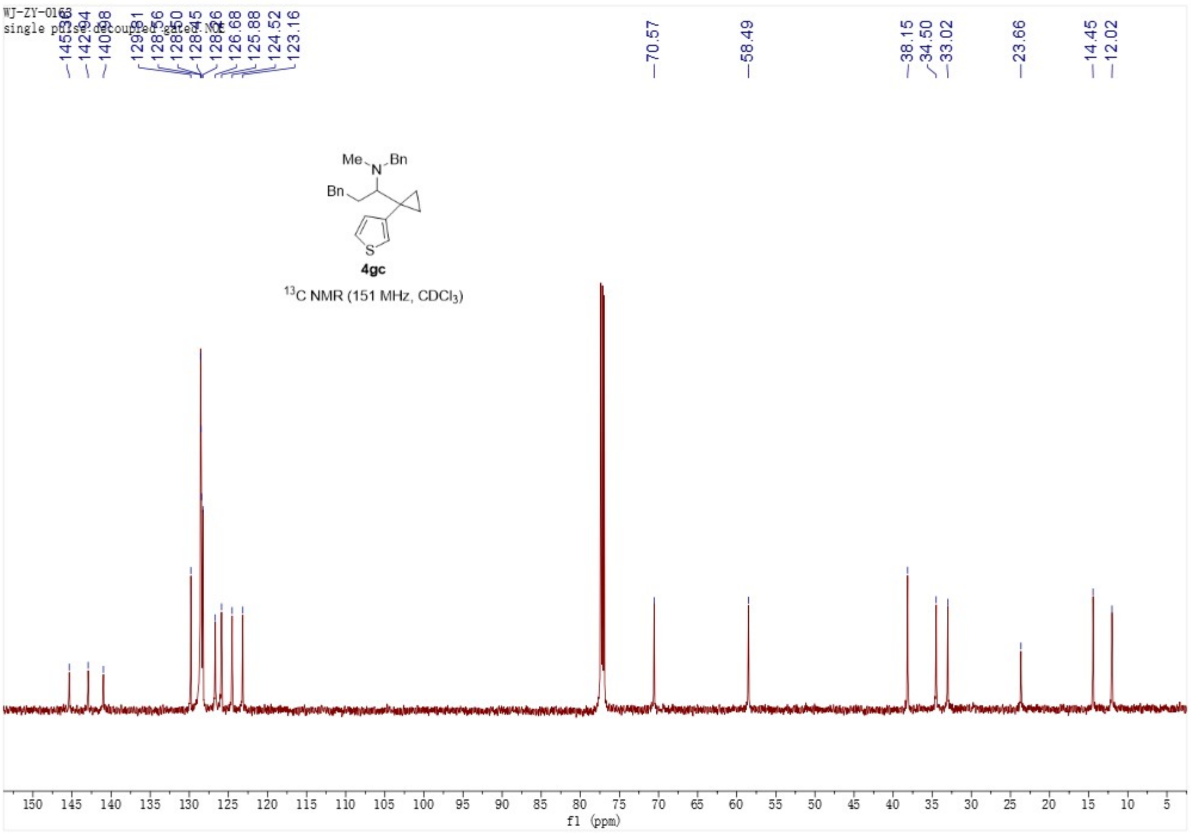
4gc
^{13}C NMR (151 MHz, CDCl$_3$)
f1 (ppm)

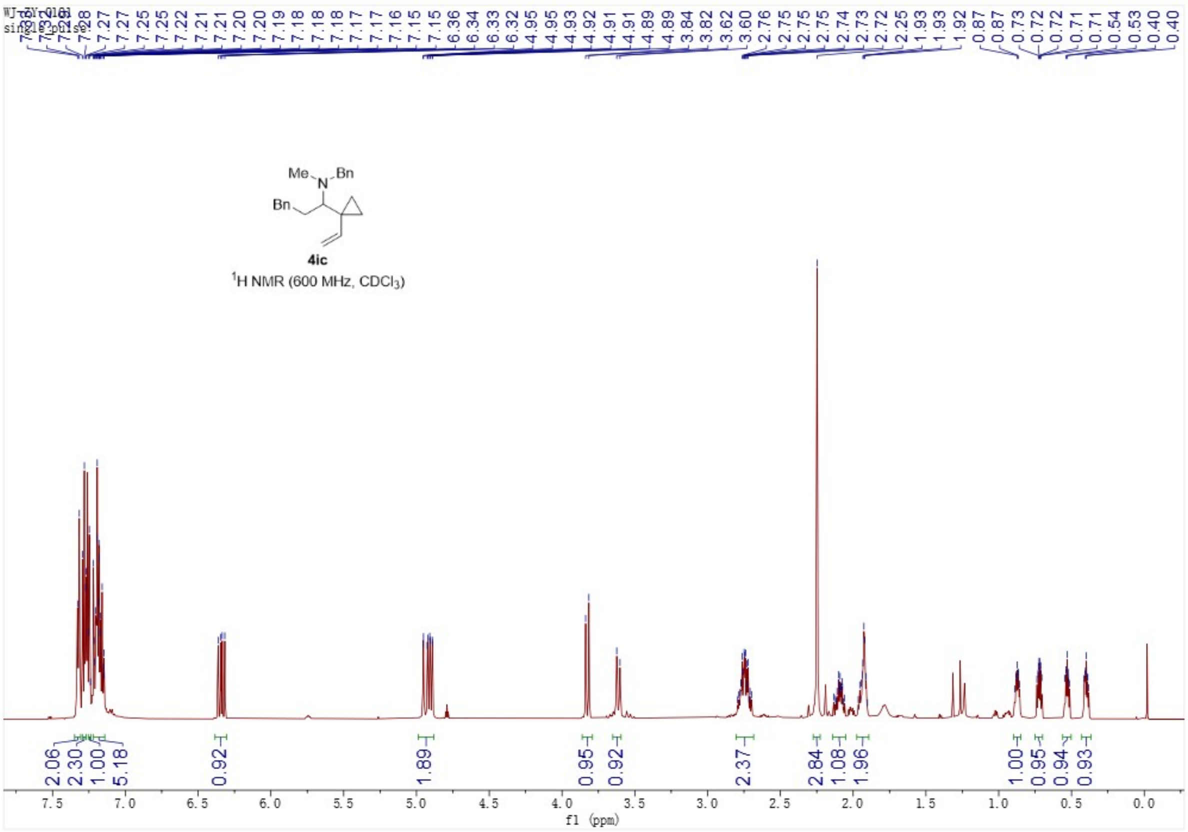

^{1}H NMR (600 MHz, $CDCl_3$)

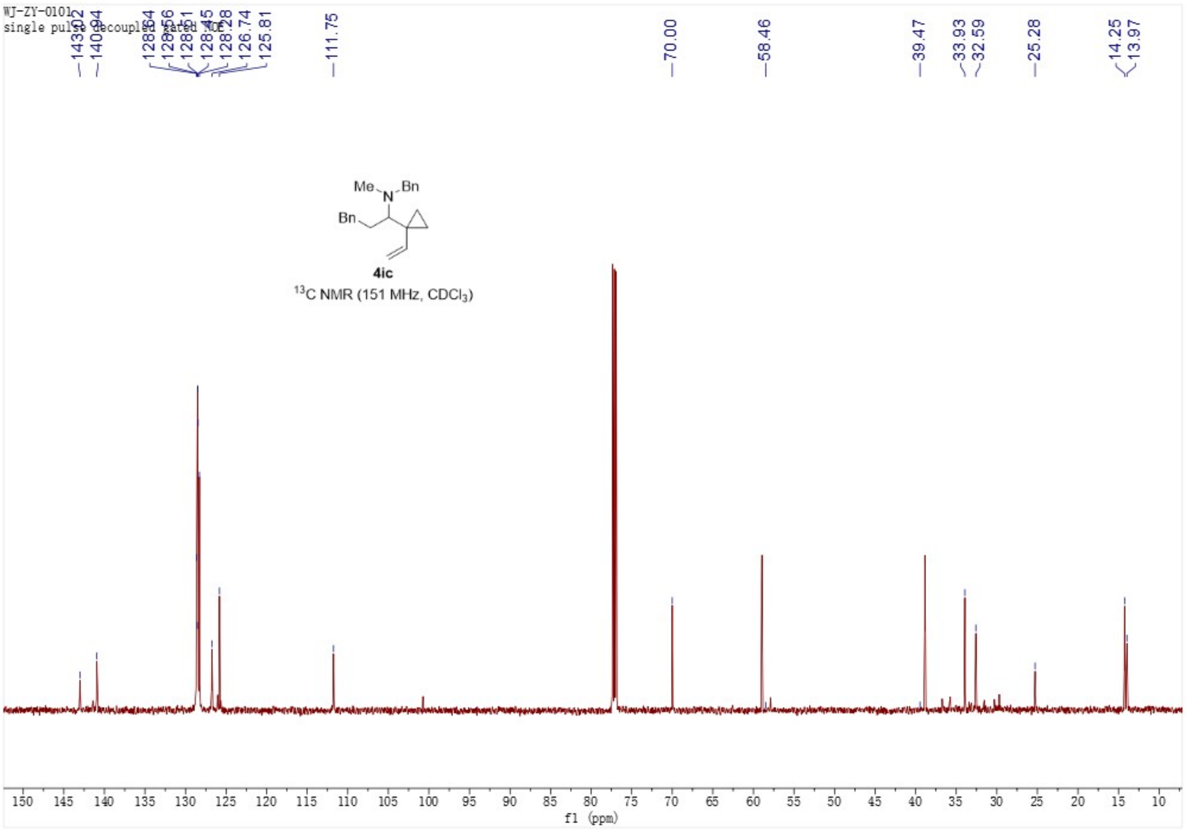

^{13}C NMR (151 MHz, $CDCl_3$)

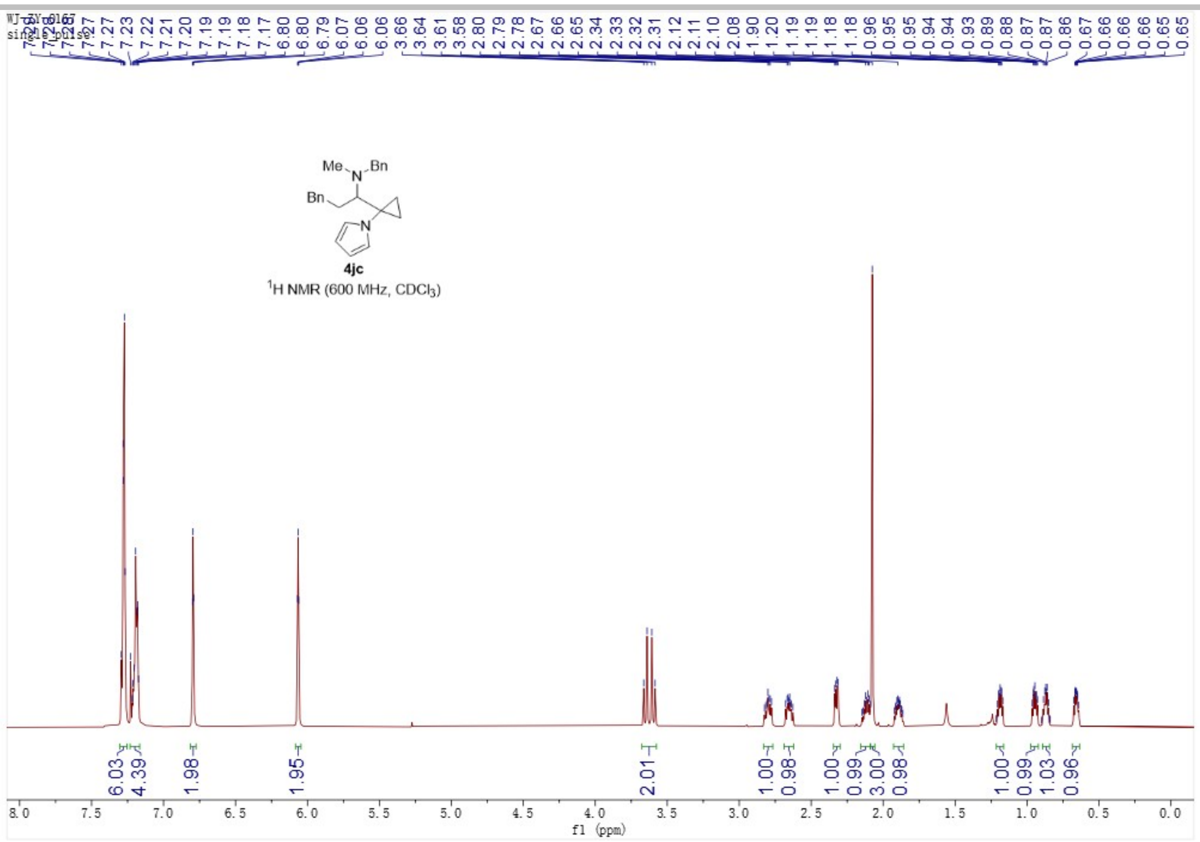
Me
N
Bn
Bn
N
4jc
^{1}H NMR (600 MHz, CDCl$_3$)
f1 (ppm)

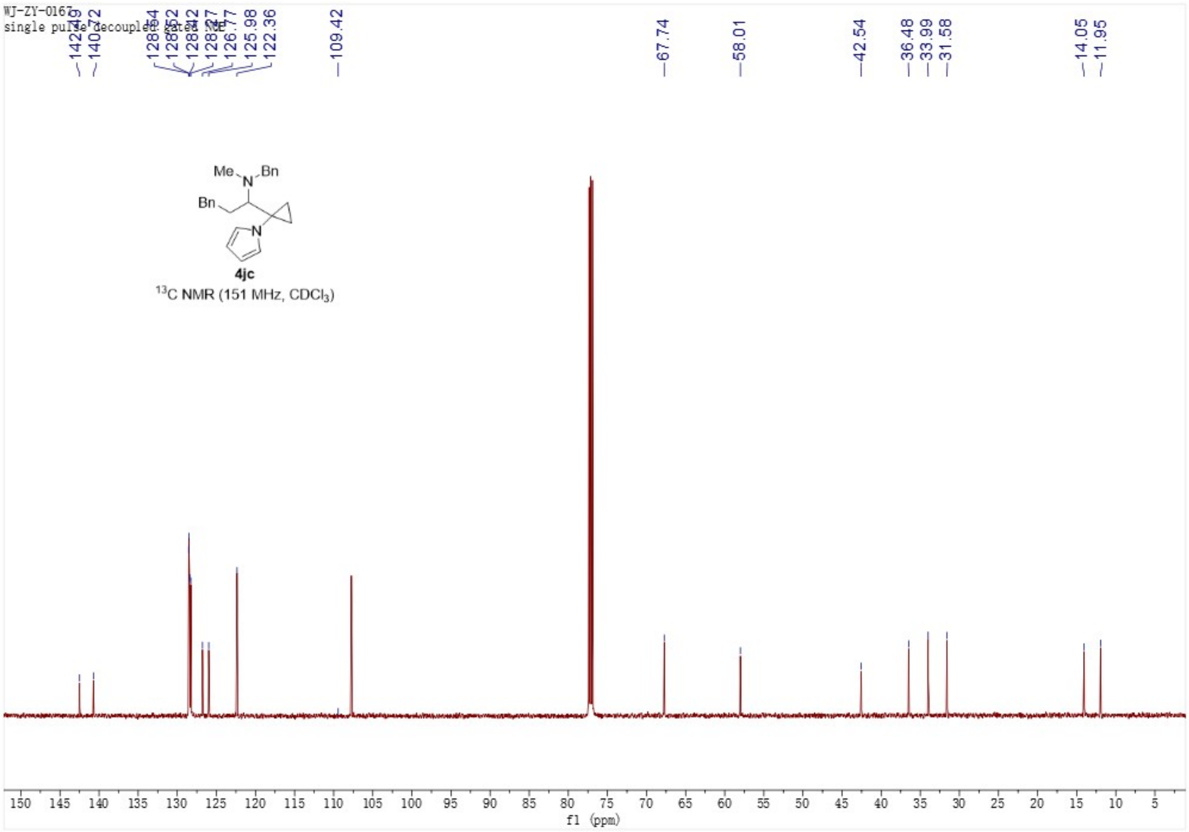
Me
N
Bn
Bn
N
4jc
^{13}C NMR (151 MHz, CDCl$_3$)
f1 (ppm)

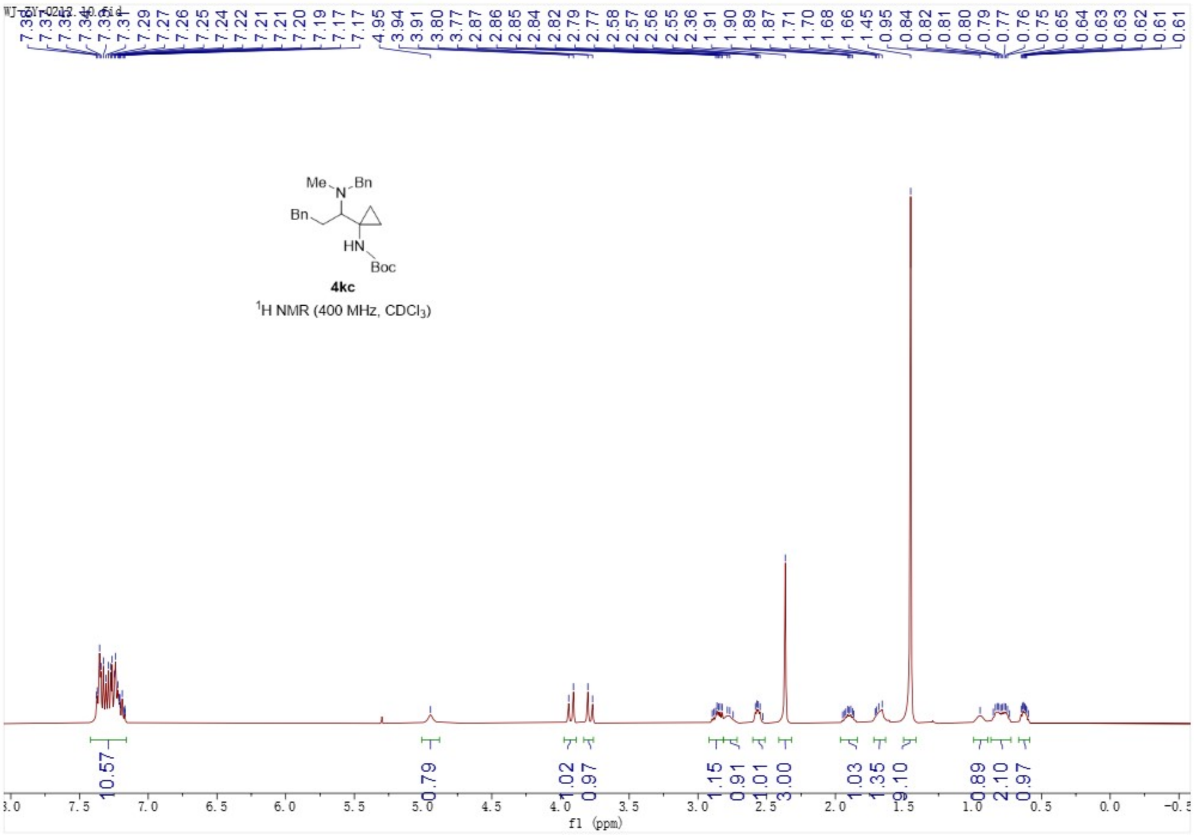

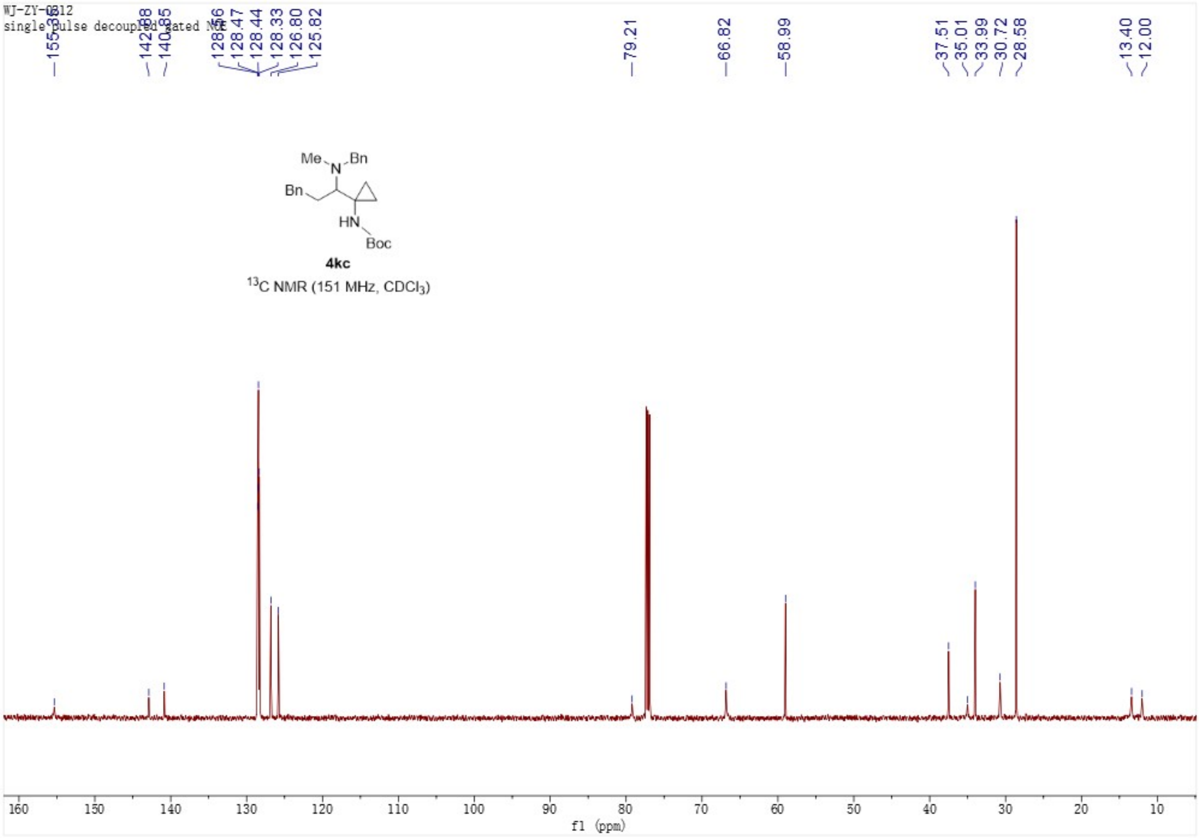

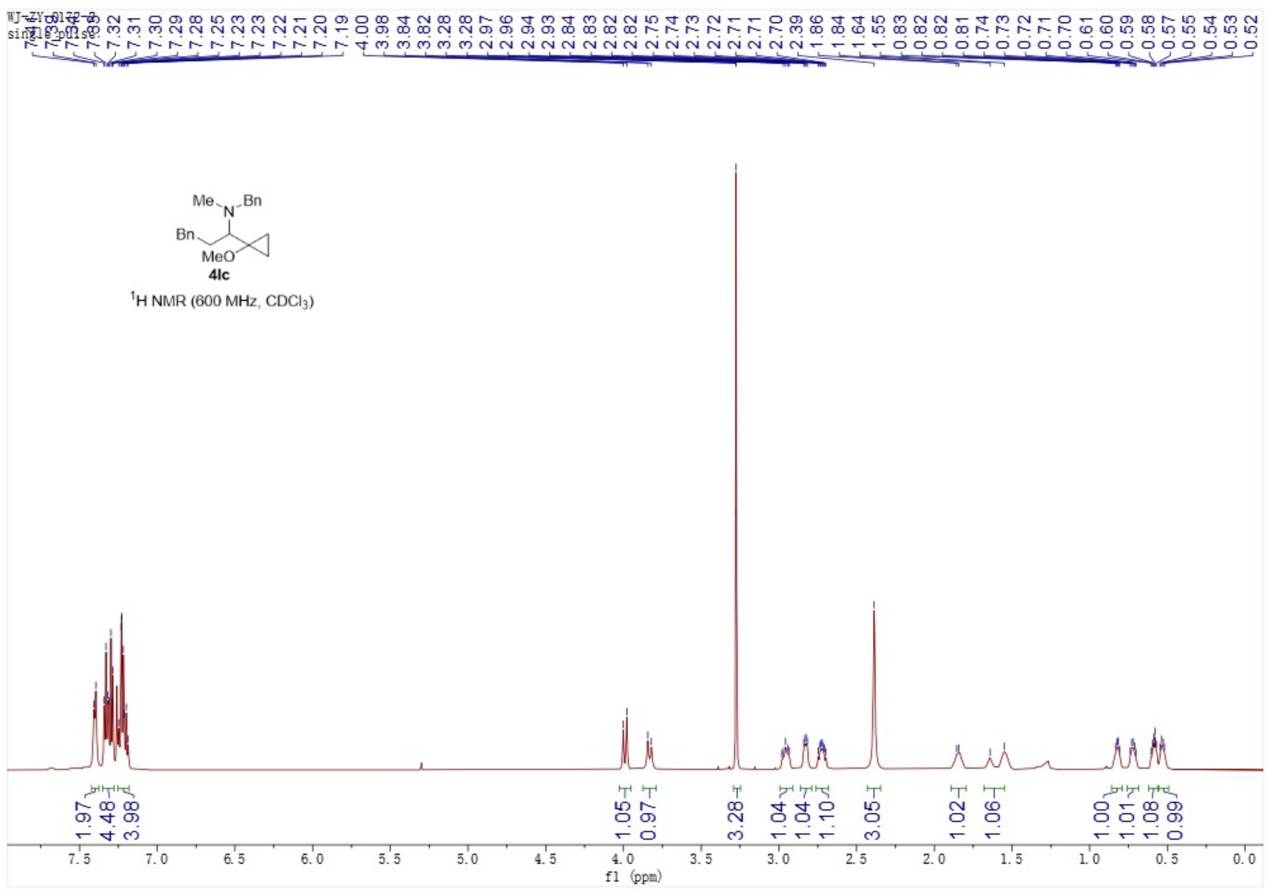

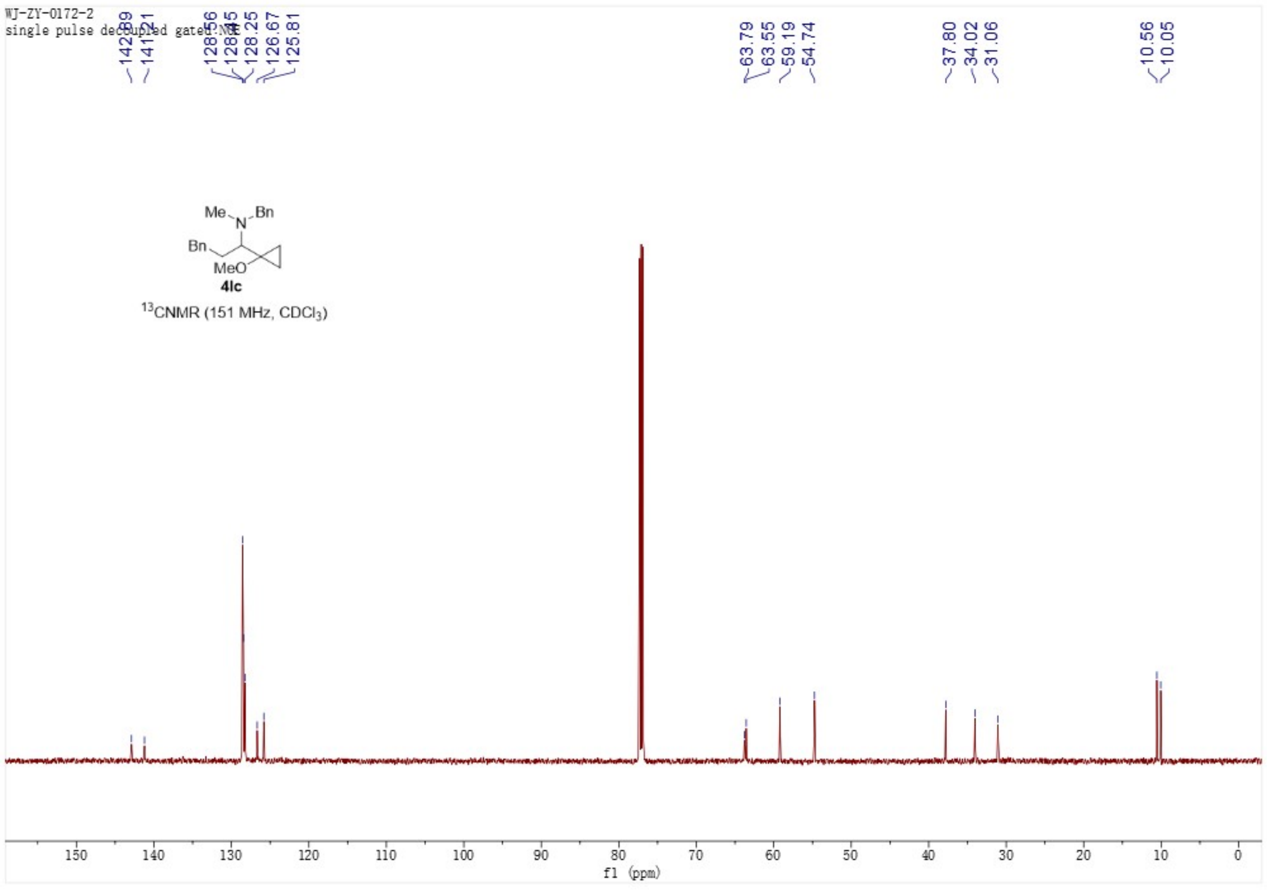

12. References:

[1] D. C. Salgueiro, B. K. Chi, I. A. Guzei, P. Garcia-Reynaga, D. J. Weix, *Angew. Chem. Int. Ed.* **2022**, *61*, e202205673
[2] S. Yang, W. T. Jiang, B. Xiao, *Chem. Comm.* **2021**, *57*, 8143-8146.
[3] S. Bonciolini, A. Pulcinella, M. Leone, D. Schiroli, A. L. Ruiz, A. Sorato, M. A. J. Dubois, R. Gopalakrishnan, G. Masson, N. Della Ca, S. Protti, M. Fagnoni, E. Zysman-Colman, M. Johansson, T. Noel, *Nat. Comm.* **2024**, *15*, 1509-1517.
[4] J. M. Phelps, R. Kumar, J. D. Robinson, J. C. K. Chu, N. J. Flodén, S. Beaton, M. J. Gaunt, *J. Am. Chem. Soc.* **2024**, *146*, 9045-9062.
[5] L. Zhou, R. Huang, S. Lu, B. Liu, M. Gao, B. Xu, *Org. Lett.* **2023**, *25*, 1415-1419.
